# Deep learning-based classifier for carcinoma of unknown primary using methylation quantitative trait loci

**DOI:** 10.1093/jnen/nlae123

**Published:** 2024-11-28

**Authors:** Adam Walker, Camila S Fang, Chanel Schroff, Jonathan Serrano, Varshini Vasudevaraja, Yiying Yang, Sarra Belakhoua, Arline Faustin, Christopher M William, David Zagzag, Sarah Chiang, Andres Martin Acosta, Misha Movahed-Ezazi, Kyung Park, Andre L Moreira, Farbod Darvishian, Kristyn Galbraith, Matija Snuderl

**Affiliations:** Department of Pathology, NYU Langone Health and NYU Grossman School of Medicine, New York, NY, United States; Department of Pathology, NYU Langone Health and NYU Grossman School of Medicine, New York, NY, United States; Brain and Spine Tumor Center, Laura and Isaac Perlmutter Cancer Center, NYU Langone Health, New York, NY, United States; Department of Pathology, NYU Langone Health and NYU Grossman School of Medicine, New York, NY, United States; Department of Pathology, NYU Langone Health and NYU Grossman School of Medicine, New York, NY, United States; Department of Pathology, NYU Langone Health and NYU Grossman School of Medicine, New York, NY, United States; Department of Pathology, NYU Langone Health and NYU Grossman School of Medicine, New York, NY, United States; Department of Pathology, NYU Langone Health and NYU Grossman School of Medicine, New York, NY, United States; Department of Pathology, NYU Langone Health and NYU Grossman School of Medicine, New York, NY, United States; Department of Pathology, NYU Langone Health and NYU Grossman School of Medicine, New York, NY, United States; Department of Pathology, NYU Langone Health and NYU Grossman School of Medicine, New York, NY, United States; Department of Pathology and Laboratory Medicine, Memorial Sloan Kettering Cancer Center, New York, NY, United States; Department of Pathology, Indiana University, Indianapolis, IN, United States; Department of Pathology, NYU Langone Health and NYU Grossman School of Medicine, New York, NY, United States; Department of Pathology, NYU Langone Health and NYU Grossman School of Medicine, New York, NY, United States; Department of Pathology, NYU Langone Health and NYU Grossman School of Medicine, New York, NY, United States; Department of Pathology, NYU Langone Health and NYU Grossman School of Medicine, New York, NY, United States; Department of Pathology, NYU Langone Health and NYU Grossman School of Medicine, New York, NY, United States; Department of Pathology, NYU Langone Health and NYU Grossman School of Medicine, New York, NY, United States; Brain and Spine Tumor Center, Laura and Isaac Perlmutter Cancer Center, NYU Langone Health, New York, NY, United States

**Keywords:** cancer of unknown primary, classifier, deep learning, DNA methylation, EPIC array, metastasis, molecular pathology

## Abstract

Cancer of unknown primary (CUP) constitutes between 2% and 5% of human malignancies and is among the most common causes of cancer death in the United States. Brain metastases are often the first clinical presentation of CUP; despite extensive pathological and imaging studies, 20%-45% of CUP are never assigned a primary site. DNA methylation array profiling is a reliable method for tumor classification but tumor-type-specific classifier development requires many reference samples. This is difficult to accomplish for CUP as many cases are never assigned a specific diagnosis. Recent studies identified subsets of methylation quantitative trait loci (mQTLs) unique to specific organs, which could help increase classifier accuracy while requiring fewer samples. We performed a retrospective genome-wide methylation analysis of 759 carcinoma samples from formalin-fixed paraffin-embedded tissue samples using Illumina EPIC array. Utilizing mQTL specific for breast, lung, ovarian/gynecologic, colon, kidney, or testis (BLOCKT) (185k total probes), we developed a deep learning-based methylation classifier that achieved 93.12% average accuracy and 93.04% average F1-score across a 10-fold validation for BLOCKT organs. Our findings indicate that our organ-based DNA methylation classifier can assist pathologists in identifying the site of origin, providing oncologists insight on a diagnosis to administer appropriate therapy, improving patient outcomes.

## INTRODUCTION

Cancer of unknown primary (CUP) is one of the top 10 most frequent cancers worldwide and constitutes between 2% and 5% of all human malignancies.^1–4^ CUPs are a heterogeneous group of metastatic tumors that have an annual incidence rate of 4.1 per 100 000 in the United States.^1^^,^^5^ Although this incidence rate has decreased steadily over time as cancer analysis tools have progressed, CUP remains a frequent cause of cancer-related death.^5–8^ Due to their heterogeneous nature, there is no standard treatment for CUP tumors, as most cancer treatment protocols are based on the organ of origin. Thus, the survival rate for CUP is often low, with studies such as Dyrvig et al.^9^ reporting only 12% overall survival at 4 years in a cohort of 542 patients.

CUP is also associated with a long diagnostic turnaround time and high cost as multiple tests and extensive hospital resources are used to attempt to determine the source of the metastasis. Yet, even after extensive testing and even autopsy, approximately 20%-45% of CUP are never assigned a primary site,^4^^,^^10^^,^^11^ resulting in an increased financial and mental burden for patients.^12^ Carcinoma of unknown primary encompasses a significant subset of cancers with an unknown primary site,^2^^,^^13^ and the source often cannot be determined despite immunohistochemical analysis and/or sequencing assessment.^14–16^

DNA methylation is an evolutionarily conserved regulator of gene expression and programs of cell differentiation and remains preserved even when other phenotypes, ie, gene or protein expression, are lost. DNA methylation has been known to be a major driver of oncogenesis for several decades.^17^ In 2002, Siegmund and Laird^18^ showed that methylation profile analysis could tie methylation changes to specific cell populations or genetic changes, and Adorján et al.^19^ demonstrated the ability for methylation to be used as a basis for a cancer classifier. DNA methylation assays continued to expand over the next decade and hit another milestone in 2012^20^; the creation of the Illumina HumanMethylation450 Bead Chip (450K array) allowed for the investigation of 450 000 CpG islands along the human genome and was largely focused on gene promoters.^21^ In 2016, Illumina released their updated EPIC array that analyzed approximately 850 000 methylation loci (850K array) and had significantly enriched enhancer coverage.^22^ Due to probe discrepancies between the two arrays, it is often difficult to utilize the two array types within the same study, and the combination of 450k and EPIC array data ultimately results in utilizing only a small set of probes common between both arrays, limiting the analytical accuracy of a diagnostic classifier.

Array-derived DNA methylation data formed the basis of the machine learning classifier developed by Capper et al.^23^ and is currently used as a standard of care for primary CNS tumor diagnosis. The random forest-based classifier focuses on identifying primary CNS tumors and a small subset of non-CNS tumors such as sarcomas occurring in the CNS, primary CNS lymphoma, and melanoma. This was soon followed by sarcoma^24^ and sinonasal tumor classifiers.^25^ In addition to the random forest algorithm, the sinonasal classifier also utilizes a support vector machine (SVM). Better performance of the SVM compared to the random forest algorithm indicated that some cancers may require a combination of different classifiers and machine learning methods for the most accurate diagnosis. Both random forest and SVM require a high number of reference tumors for each unique tumor type. However, the major clinical utility of CUP analysis is determining the organ of origin of the tumor; we hypothesized that classification based on organ-specific DNA methylation loci might be sufficient to enable clinically actionable classification, requiring significantly fewer reference samples.

Quantitative trait loci (QTLs) are loci in the genome that are significantly associated with specific phenotypes or traits within the given organism.^26^ There are many different subtypes of QTLs based on the various forms of molecular analysis, such as expression QTLs (eQTLs) and methylation QTLs (mQTLs). Recent studies have attempted to identify normal background methylation signatures for organs within the human body.^27–29^ It has also been shown that those unique methylation signatures remain preserved during carcinogenesis, serving as an epigenetic developmental fingerprint.^28^

**Figure 1.**
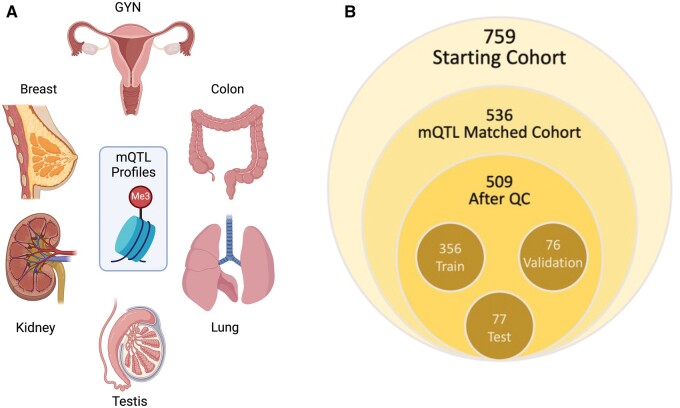
(A) Tissue types involved in this study. All of these have matching methylation quantitative trait loci profiles. Created with BioRender.com. (B) Cohort breakdown indicating samples used for processing and model creation.

Oliva et al.^30^ sought to identify QTLs within sections of the mammalian methylome that correspond to preserved background methylation patterns for major organs and identified mQTLs corresponding to probe subsets on the Illumina EPIC 85f0k arrays.^31^ Using this approach, CUP origin could be determined using a subset of organ-specific mQTL probes (Figure 1A).

In this study, we developed a DNA methylation classifier specifically targeted at identifying the organ of origin for carcinoma of unknown primary by using a deep learning neural network and limiting the inputs to the organ-specific methylation probes identified within the organ-specific mQTLs. The methylation assay and organ of origin-based classifier can be used as one of the first tests to either outright diagnose the CUP or provide a roadmap for further imaging and immunohistochemical or molecular analyses.

## METHODS

### Patients and neuropathology analysis

We retrospectively analyzed DNA methylation data of 759 primary and/or metastatic carcinomas profiled at NYU Langone Health (NYULH) between 2018 and 2023. Pathology diagnosis was retrieved from medical records. This study was performed in accordance with the approval of the NYU Langone Institutional Review Board (IRB) and in accordance with its policy and guidelines, IRB No.: i14-00948.

### DNA methylation profiling and analyses

DNA methylation profiling analysis was performed using a clinically validated DNA methylation profiling. Briefly, DNA extraction from formalin-fixed, paraffin-embedded tissue scrolls was performed using the automated Maxwell nucleic acid purification platform (Promega, Madison, WI, United States). Genome-wide DNA methylation profiling was performed using the Infinium Methylation EPIC (EPIC) BeadChip array (Illumina) according to the manufacturer’s instructions as previously described.^32^ CNS metastases were analyzed using clinically validated CNS tumor classifier as previously described.^33^

For the development of the CUP classifier, all samples underwent processing steps for Illumina methylation arrays as previously described.^32^ The resulting intensity data (idat) files were processed using the minfi Bioconductor package in R. Standard preprocessing and initial quality control were performed to exclude samples with low quality, removing individual probes that were not sufficiently expressed in the samples. Sex chromosome probes, common SNPs, and poorly performing probes were also removed as described previously.^34–36^

Tumor samples were processed over several years and, as a consequence, samples were run on multiple batches of Illumina bead chips over that time. Therefore, samples were batch corrected using the sample processing year as the index for the batching and samples were normalized with the use of the “funnorm” normalization, which is one of the most common normalization methods for Illumina EPIC methylation data processing. Further quality checks were performed using dimensionality reduction and clustering utilizing t-sne and UMAP. The development of the classifier was completed in python 3.8 mainly utilizing pytorch (2.0.0) and sklearn (1.2.2). A full list of packages used in methylation processing and classifier development is in Supplementary Data. Additionally, we utilized Neptune (Neptune.ai) to assist with logging our run metadata and better-assessing model fitness.

### Classifier development

**Figure 2.**
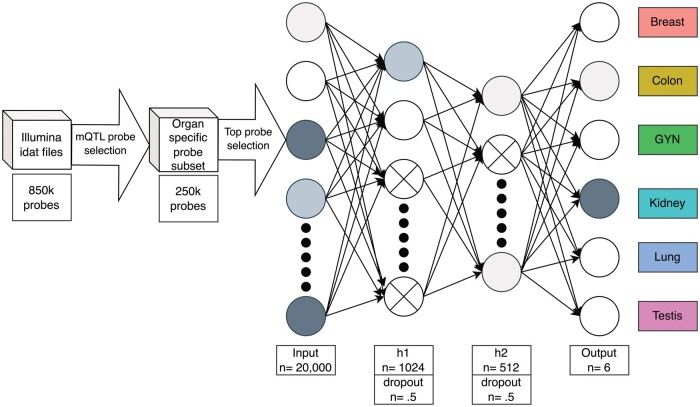
Methylation array processing pipeline steps and presentation of deep learning model architecture. Created with drawio.com.

mQTLs were extracted from Oliva et al.^30^ The model architecture is shown in Figure 2. The input layer had 20 000 nodes and each subsequent layer decreased to 1024, 512, and finally 6 for the output. ReLU (rectified linear unit) layers and 0.5 dropout layers are featured between each of the fully connected layers to assist model stability and prevent overfitting. A batch size of 16 and an epoch count of 50 were selected for training. To assess the robustness of our model, we performed a 10-fold cross-validation. The code for processing input 850k methylation files as well as creating the classifier is available on github: https://github.com/a-dev-walker/CUP_Methylation_Classifier.

## RESULTS

Our initial cohort was composed of 759 carcinoma samples (Figure 1B). The samples were a mix of metastatic tumors with known origins as well as primary tumors. Oliva et al.^30^ previously identified organ-specific mQTLs and after cross-referencing with our cohort we limited our analysis to 536 samples with confirmed pathologic diagnoses of breast, lung, ovarian/gynecologic, colon, kidney, or testis (BLOCKT, Figure 1A). Upon QC and removing of samples not passing criteria, our final cohort was composed of breast (143), gynecologic (91), testicular (86), lung (85), kidney (84), and colon (20) for a total of 509 tumors (Figure 1B). These classes corresponded to 6 out of 9 classes described by Oliva et al.^30^ Patient demographics are summarized in Table 1. The final cohort was composed of 82 brain metastases (16% of the cohort) with confirmed organ of origin and 427 (84%) primary tumors. Subsets of this cohort were previously published, notably lung,^34^ gynecological,^37^ breast,^38^ and testicular^39–41^ tumors.

**Table 1.** Cohort demographics.

Age: median (range)	62 (18-92)
Male:female	46%:54%
Primary vs CNS metastatic samples (%)	84:16
Organ of origin *N*: 509 total	
Breast (breast)	143 (28%)
Gynecologic (cervix, ovary, fallopian tube, uterus)	91 (18%)
Testicular (testis)	86 (17%)
Lung (lung)	85 (17%)
Kidney (kidney)	84 (16%)
Colon (anal, appendix, colon)	20 (4%)

Abbreviation: CNS, central nervous system.

Breakdown of tumor samples included in final classifier; organs of origin are noted for classes including multiple organ types.

**Figure 3.**
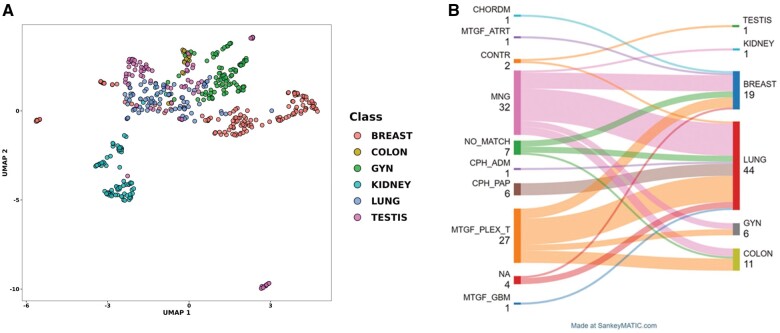
(A) Uniform Manifold Approximation and Projection (UMAP) of top 20 000 methylation probes. This UMAP is created irrespective of the following classification algorithm and was used to help identify outlier cases for reevaluation. This unsupervised clustering is somewhat able to separate out distinct organ groups; more sophisticated machine learning models are needed for proper classification. (B) Comparison of 82 carcinomas metastatic to the central nervous system classified by the CNS brain tumor classifier (left) and confirmed organs of origin (right). With the exception of metastases, which classified as craniopharyngioma and were all of lung origin, other metastatic tumors did not show predisposition for particular CNS methylation class. Notably, none of the metastatic tumors was classified with a high calibrated score that could lead to misdiagnosis by the CNS tumor classifier.

Brain metastases were initially analyzed using a CNS classifier to rule out a primary CNS tumor, we first analyzed methylation classes and calibrated scores of all brain metastasis using CNS classifier. Metastatic tumors predominantly showed classes of meningioma group (39%) and choroid plexus tumors (28%), followed by No Match and craniopharyngioma groups. Interestingly, in 5 tumors, there was a discrepancy between a methylation class and subclass. Most importantly, none of the metastatic tumors showed a high calibrated score (>0.9), confirming the analytical specificity of the CNS classifier and a low risk of misclassification of metastatic carcinomas as primary CNS neoplasms (Table S1). When we reviewed CNS metastatic samples and compared them with pathology and clinical diagnosis, we observed that samples identified as meningioma or plexus tumors by CNS classifier were distributed relatively evenly among lung, breast, colon, and GYN groups. Interestingly, all metastases that classified poorly as craniopharyngioma on the CNS classifier were metastatic lung carcinoma, perhaps due to the developmental similarities of craniopharyngioma epithelium to the upper respiratory tract (Figure 3B).

Next, we identified a subset of 185k loci related to the BLOCKT organs. These QTLs correspond to loci in the genome where DNA methylation has previously been correlated with a phenotype. The following analysis was limited to those 185k probes.

We then performed dimensionality reduction and clustering utilizing t-SNE and UMAP to ensure that our samples clustered with their assumed organ groups as well as pathological diagnoses. Based on these attempts, we further refined the dataset by reviewing the outliers and confirming pathological diagnoses. The final UMAP of the final 509 sample cohort is shown in Figure 3A. For model training efficiency, we further limited our probes to the top 20 000 most variable probes (Table S2), and the outputs of the model were scores for the 6 BLOCKT organs of origin. Samples were split into non-overlapping Training (*N* = 356), Validation (*N* = 76), and Testing (*N* = 77) cohorts (Figure 1B). The training set was used to iteratively train the machine learning model to learn how to identify the methylation profiles of each cancer type. The validation set was used to iteratively validate how well the model learned over the training process. The test set was a holdout group of samples used as the ultimate blinded benchmark of how well the model learned the methylation profiles of each cancer type.

After the Training and Validation cohorts, the Test cohort cases were processed, and the results were compared to pathology diagnosis. Overall, our model achieved a 10-fold cross-validation accuracy score of 93.12% thereby outperforming the goal of 90%. As shown in Table 2, on 10-fold cross-validation, our model also achieved a top2 average accuracy of 96.10% and an average F1 score of 93.04%. These results indicate that our model is robust to data perturbations and our architecture is solid for future dataset expansion.

**Table 2.** Results for 10-fold cross-validation of the model for robustness.

10-fold cross-validation	
Average accuracy	93.12%
Average top2 accuracy	96.10%
Average F1 score	93.04%

F1 score—Secondary accuracy metric evaluated using precision and recall.

**Figure 4.**
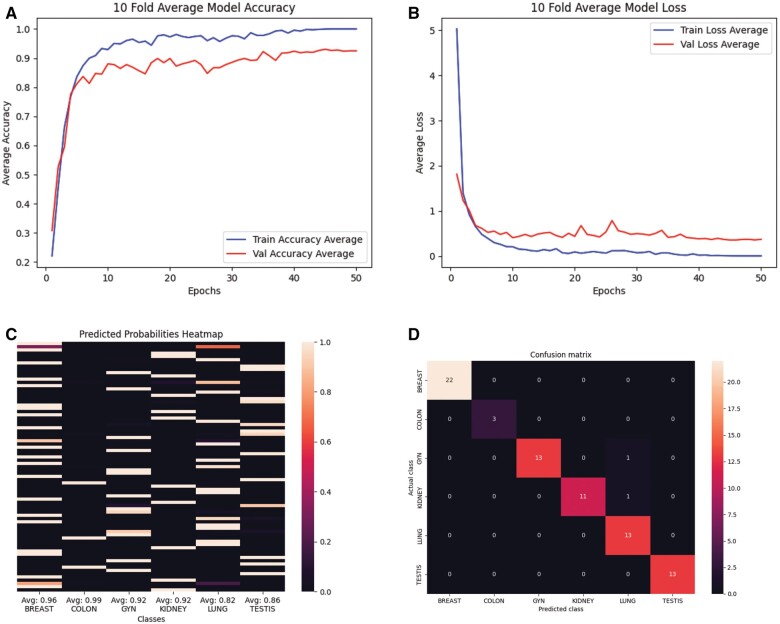
(A) 10-Fold average model training and validation accuracy over 50 epochs. (B) 10-Fold average model training and validation loss over 50 epochs. (C) Test set prediction probability scores for the best performing k-fold (based on lowest validation loss) indicating how confident the model was about its guesses. Some samples were predicted in multiple classes with low probability. (D) Test set confusion matrix for the same k-fold run indicating instances of correct and incorrect predictions.

Using a 10-fold cross-validation, the model achieved an average training loss of 0.003 and an average validation loss of 0.371 after 50 epochs (Figure 4B). We also investigated how confident the model was in each of its predictions for the k-fold run achieving the lowest validation loss given train/validation/test split (Figure 4C). For the same run, we created a confusion matrix to determine whether classifier errors occurred in tumors from a specific organ of origin (Figure 4D). The data indicate that the classifier had the most difficulty with differentiating between kidney/gynecologic and lung origins. The classifier had the highest confidence and accuracy with correctly predicting colon as the organ of origin, albeit that there were only 3 colon samples in the given test set.

Finally, we tested our CUP classifier on 11 cases sent to us for CNS DNA methylation classifier analysis, which were inconclusive (negative) using CNS classifier and were subsequently analyzed using our CUP classifier. Two of the CUP samples failed our initial QC, and we proceeded with the other 9 samples. For this processing, we used the best-performing weights from our cross-validation, and we utilized the same most variable probes from our initial cohort. When correlated with the clinical follow up, three cases were lost to clinical follow up, one of them, with a high score for Lung origin showed KRAS G12D mutation, which is compatible with a lung primary, albeit not exclusive. Four tumors classifying as lung primary showed lung lesions on imaging, and in one patient, who classified as breast cancer, a new breast lesion was confirmed. Lastly, in one patient, who classified as a colon primary, there was a remote history of colon cancer (Table S3). This suggests that when tumors do not classify with the CNS classifier, the CUP classifier may provide valuable information for management.

## DISCUSSION

CUP brain metastasis represents an unmet medical need in neurooncology as knowledge of the organ of origin is required to administer appropriate systemic therapy. While the current brain tumor classifier can detect melanoma and lymphoma, (and the sarcoma classifier can identify mesenchymal tumors), it is not designed to differentiate among different sources of metastatic disease, particularly carcinoma. DNA methylation mQTLs enable cell of origin-based classification of cancers and can be used as a diagnostic tool. Here, we developed a deep learning-based methylation classifier utilizing organ-specific mQTL probes that achieved over 90% accuracy.

In 2016, Moran et al.^42^ proposed a methylation classifier to identify CUP. Their study included over 10 000 retrospective tumor samples of known origin and their testing set of 216 cases, utilizing a random forest classifier. Their classifier (EPICUP) correctly predicted the tissue of origin in 87% of their testing set. By focusing on an organ of origin rather than specific tumor entities and using developmentally conserved mQTL probes, our model was able to achieve a slightly higher accuracy while using only a fraction of cases for training and testing.

Zheng and Xu^43^ created a deep neural network CUP methylation classifier utilizing tumor data of 7339 patients from TCGA corresponding to 18 different cancer origins and using the Illumina 450k methylation array. Most of their tumor populations were carcinomas of various origins throughout the human body. The network itself utilized an input layer of size 10 360 and only 2 hidden layers; they were able to demonstrate specificity of 99.91% and a sensitivity of 93.43%. This model is very similar to the one that we devised through the process of various parameter tuning. Since neither group of authors made their code publicly available, we could not perform a head-to-head comparison on our cohort.

We developed our model utilizing Illumina EPIC 850k methylation arrays, which contain larger genomic content. Therefore, our model is not limited to the overlap of probes present on both 450k and EPIC array. Due to the cryptic nature of CUP, it is difficult to establish overall success rates and costs for identifying the source of CUP. Typically, the process is completed in collaboration with several physicians working together through many imaging and pathology testing modalities. Therefore, our baseline accuracy rate of 93% using a single molecular assay that is already implemented in many molecular laboratories for primary CNS tumor classification can be considered highly beneficial and cost effective.

In attempting to understand the flaws of the model, we created the predicted probabilities heatmap and confusion matrix (Figure 4C and D). The predicted probabilities heatmap indicates that the model had a high predictive index with respect to the classifications rendered. This predictive index will be useful in future use of this model to provide physicians with information regarding the certainty of a given prediction. The confusion matrix indicates which classes were properly called and which had a higher prevalence of being incorrectly called, as well as what they were incorrectly called as. Our data suggest that the model had the most errors in classifying some kidney and gynecologic samples as lung samples. This could either indicate that the model was incorrectly calling these samples or that the ground truths themselves were mistaken. The ground truths in this dataset were diagnosed by pathologists and unfortunately, CUP is a notoriously difficult entity to classify. Thus, some errors could have been made in the creation of the ground truth labels. For further work, these samples could be reevaluated by expanding the cohort and including rigorous genomic orthogonal studies confirming the organ of origin.

The significant limitation of our study is the sample size. Because we based our study on data from Illumina EPIC 850k arrays, we could not utilize large publicly available datasets such as TCGA which utilized 450k arrays. With routine use of DNA methylation for tumor classification, more carcinoma samples profiled using the EPIC arrays become available, our datasets will grow to encompass more samples with more detailed epigenetic information. As DNA methylation profiling becomes more frequent, CUP cases can be profiled together with primary CNS tumors to increase the volume and decrease turnaround time.

Lastly, we were limited to only six organ classes for which mQTL profiles have already been identified. Future studies may follow the same protocol as Oliva et al.^30^ to create mQTL profiles for additional organs of interest, expanding the CUP classifier to other organs. Future work toward creating newer versions of CUP classifiers may also require comparison of neural networks and simpler machine-learning models such as Random Forest for methylation classifiers.

In conclusion, CUP brain metastases represent a significant clinical and diagnostic challenge in neuropathology and oncology and are associated with prolonged time to diagnosis, high costs, and poor outcomes. Here, we developed a deep learning-based methylation classifier utilizing mQTL probes that achieved 93% accuracy. This model can be implemented into present clinical DNA methylation profiling workflows for CNS tumors to assist pathologists in rapidly and accurately diagnosing future cancers of unknown primary.

## Supplementary Material

nlae123_Supplementary_Data
