# Supplementary material for: Deep learning-based classifier for carcinoma of unknown primary using methylation quantitative trait loci
Source: J Neuropathol Exp Neurol. 2024 Nov 28;84(2):147–54. doi: 10.1093/jnen/nlae123 (PMC11747144; doi:10.1093/jnen/nlae123)
Supplement: nlae123_Supplementary_Data [file nlae123_supplementary_data.zip › nlae123_Supplementary_Data/Supplemental Table 3.docx]

**Supplemental Table 3**

| **Case** | **Clinical history at submission** | **Highest CUP Class** | **Confidence score** | **Follow-up** |
| --- | --- | --- | --- | --- |
| 1 | Poorly differentiated neoplasm: CNS tumor subtype | LUNG | 0.97 | Lung mass on imaging |
| 2 | Brain tumor | COLON | 0.51 | History of colon cancer |
| 3 | Primary vs metastatic | BREAST | 0.53 | New breast lesion, biopsy carcinoma |
| 4 | Glioblastoma | LUNG | 0.56 | Lung mass on imaging |
| 5 | Metastatic primary of unknown origin, h/o leukemia | LUNG | 0.6 | Lung mass on imaging |
| 6 | Malignant brain tumor | LUNG | 0.99 | Lung mass on imaging |
| 7  8  9 | Malignant brain tumor  Malignant brain tumor  Malignant brain tumor | LUNG  KIDNEY  GYN | 0.94  0.73  0.65 | No clinical follow up available, KRAS G12D mutated  Lost to follow-up  Lost to follow-up |
