# Supplementary material for: Deep learning-based classifier for carcinoma of unknown primary using methylation quantitative trait loci
Source: J Neuropathol Exp Neurol. 2024 Nov 28;84(2):147–54. doi: 10.1093/jnen/nlae123 (PMC11747144; doi:10.1093/jnen/nlae123)
Supplement: nlae123_Supplementary_Data [file nlae123_supplementary_data.zip › nlae123_Supplementary_Data/Walker_et_al_JNEN_Supplemental_Table_1.docx]

**Supplemental Table 1:** Results of brain DNA methylation classifier for metastatic brain tumors

| **Group** | **Subgroup** | **Count** | **Average Group Score** | **Average Subgroup Score** |
| --- | --- | --- | --- | --- |
| CHORDM | CHORDM | 1 | 0.158 | 0.158 |
| CONTR | CONTR | 2 | 0.547 | 0.547 |
| CPH_ADM | CPH_ADM | 1 | 0.118 | 0.118 |
| CPH_PAP | CPH_PAP | 6 | 0.260 | 0.260 |
| MNG | MNG | 32 | 0.177 | 0.177 |
| MTGF_ATRT | ATRT_MYC | 1 | 0.274 | 0.213 |
| MTGF_PLEX_T | PLEX_PED_B | 23 | 0.410 | 0.397 |
| NO_MATCH | NO_MATCH | 7 | 0.000 | 0.000 |
| NA | NA | 4 | 0.000 | 0.000 |
| Mismatched Group and Subgroup | | | | |
| MTGF_PLEX_T | ETMR | 1 | 0.063 | 0.053 |
|  | HGNET_MN1 | 1 | 0.079 | 0.056 |
|  | MB_G3 | 1 | 0.060 | 0.043 |
|  | MNG | 1 | 0.087 | 0.065 |
| MTGF_GBM | MNG | 1 | 0.087 | 0.057 |

CHORDM – Chordoma

CONTR – Control tissue-inflammatory tumor

CPH_ADM – Adamantinous craniopharyngioma

CHP_PAP – Papillary craniopharyngioma

MNG – Meningioma

MTGF_ATRT – Methylation group family atypical teratoid/rhabdoid tumor

ATRT_MYC – Atypical teratoid/rhabdoid tumor MYC subtype

MTFG_PLEX_T – Methylation group family plexus tumor

PLEX_PED_B – Plexus tumor subtype pediatric B

ETMR – Embryonal tumor with multilayered rosettes

HGNET_MN1 – High-grade neuroepithelial tumor with MN1 alteration

MB_G3 – medulloblastoma subgroup 3

MTGF_GBM - Methylation group family Glioblastoma multiforme
