# Supplementary material for: Deep learning-based classifier for carcinoma of unknown primary using methylation quantitative trait loci
Source: J Neuropathol Exp Neurol. 2024 Nov 28;84(2):147–54. doi: 10.1093/jnen/nlae123 (PMC11747144; doi:10.1093/jnen/nlae123)
Supplement: nlae123_Supplementary_Data [file nlae123_supplementary_data.zip › nlae123_Supplementary_Data/Walker_et_al_JNEN_Supplemental_Table_2.docx]

**Supplemental Table 2:** Final list of Illumina EPIC probes used for the classifier**.**

|  | Probe ID |
| --- | --- |
| 1 | cg11738485 |
| 2 | cg13961503 |
| 3 | cg05404698 |
| 4 | cg06193597 |
| 5 | cg26212328 |
| 6 | cg08190858 |
| 7 | cg19200589 |
| 8 | cg21334513 |
| 9 | cg06766860 |
| 10 | cg07517893 |
| 11 | cg12436377 |
| 12 | cg20744163 |
| 13 | cg01510609 |
| 14 | cg16283362 |
| 15 | cg10715092 |
| 16 | cg08104202 |
| 17 | cg06378142 |
| 18 | cg24980653 |
| 19 | cg09250933 |
| 20 | cg11528328 |
| 21 | cg22399133 |
| 22 | cg18698788 |
| 23 | cg11688437 |
| 24 | cg24884703 |
| 25 | cg03352106 |
| 26 | cg07160932 |
| 27 | cg04131969 |
| 28 | cg13526469 |
| 29 | cg25017223 |
| 30 | cg13634242 |
| 31 | cg14968543 |
| 32 | cg04224064 |
| 33 | cg09248054 |
| 34 | cg23079252 |
| 35 | cg25888561 |
| 36 | cg09556826 |
| 37 | cg19058865 |
| 38 | cg19598567 |
| 39 | cg01141459 |
| 40 | cg10932018 |
| 41 | cg13947167 |
| 42 | cg13997680 |
| 43 | cg20272979 |
| 44 | cg10881128 |
| 45 | cg24368848 |
| 46 | cg07382347 |
| 47 | cg27049766 |
| 48 | cg09982773 |
| 49 | cg16269733 |
| 50 | cg20395967 |
| 51 | cg27064692 |
| 52 | cg19711530 |
| 53 | cg16672562 |
| 54 | cg11435506 |
| 55 | cg09597070 |
| 56 | cg03140412 |
| 57 | cg08495825 |
| 58 | cg10327440 |
| 59 | cg00292073 |
| 60 | cg13507084 |
| 61 | cg27282900 |
| 62 | cg15517113 |
| 63 | cg01104724 |
| 64 | cg04777726 |
| 65 | cg11146691 |
| 66 | cg18031307 |
| 67 | cg24545961 |
| 68 | cg22169990 |
| 69 | cg03048372 |
| 70 | cg23244913 |
| 71 | cg08417692 |
| 72 | cg06610484 |
| 73 | cg08445802 |
| 74 | cg14841828 |
| 75 | cg22108469 |
| 76 | cg13005202 |
| 77 | cg23478547 |
| 78 | cg06139006 |
| 79 | cg23689428 |
| 80 | cg09899914 |
| 81 | cg06405186 |
| 82 | cg20399616 |
| 83 | cg13899718 |
| 84 | cg25508679 |
| 85 | cg06471596 |
| 86 | cg12712184 |
| 87 | cg15689410 |
| 88 | cg11487526 |
| 89 | cg19535649 |
| 90 | cg18582260 |
| 91 | cg23238315 |
| 92 | cg06246357 |
| 93 | cg10863922 |
| 94 | cg01414185 |
| 95 | cg18395917 |
| 96 | cg02311374 |
| 97 | cg23279522 |
| 98 | cg06843889 |
| 99 | cg18320766 |
| 100 | cg02533339 |
| 101 | cg07873041 |
| 102 | cg03738707 |
| 103 | cg23049458 |
| 104 | cg00949442 |
| 105 | cg19170881 |
| 106 | cg03890691 |
| 107 | cg25999637 |
| 108 | cg22784187 |
| 109 | cg15775406 |
| 110 | cg06918474 |
| 111 | cg17469359 |
| 112 | cg21163347 |
| 113 | cg17642369 |
| 114 | cg26725201 |
| 115 | cg13185413 |
| 116 | cg04798314 |
| 117 | cg27102141 |
| 118 | cg18391209 |
| 119 | cg07095230 |
| 120 | cg19056926 |
| 121 | cg11610925 |
| 122 | cg27528510 |
| 123 | cg15691862 |
| 124 | cg00683332 |
| 125 | cg02486855 |
| 126 | cg14371731 |
| 127 | cg14564616 |
| 128 | cg07528692 |
| 129 | cg11213690 |
| 130 | cg12262617 |
| 131 | cg24393673 |
| 132 | cg17736443 |
| 133 | cg09716640 |
| 134 | cg26000619 |
| 135 | cg24702147 |
| 136 | cg11916478 |
| 137 | cg00164949 |
| 138 | cg09787089 |
| 139 | cg19729116 |
| 140 | cg08986950 |
| 141 | cg25702335 |
| 142 | cg23166865 |
| 143 | cg21899596 |
| 144 | cg25528916 |
| 145 | cg05477457 |
| 146 | cg14159672 |
| 147 | cg17629929 |
| 148 | cg05702092 |
| 149 | cg08222513 |
| 150 | cg09143801 |
| 151 | cg05004142 |
| 152 | cg27586797 |
| 153 | cg21762788 |
| 154 | cg12193833 |
| 155 | cg16966315 |
| 156 | cg06916001 |
| 157 | cg08013331 |
| 158 | cg21980043 |
| 159 | cg09544728 |
| 160 | cg07029873 |
| 161 | cg17192454 |
| 162 | cg05841700 |
| 163 | cg25066665 |
| 164 | cg16374999 |
| 165 | cg08416194 |
| 166 | cg02973171 |
| 167 | cg03735012 |
| 168 | cg02590401 |
| 169 | cg04418091 |
| 170 | cg03486485 |
| 171 | cg24886257 |
| 172 | cg14451382 |
| 173 | cg05745631 |
| 174 | cg08394412 |
| 175 | cg23302570 |
| 176 | cg06693983 |
| 177 | cg08813944 |
| 178 | cg13958426 |
| 179 | cg08517040 |
| 180 | cg06245037 |
| 181 | cg09469554 |
| 182 | cg12798157 |
| 183 | cg07124687 |
| 184 | cg06460587 |
| 185 | cg23445461 |
| 186 | cg06710082 |
| 187 | cg07365127 |
| 188 | cg23687466 |
| 189 | cg00097146 |
| 190 | cg03260566 |
| 191 | cg12484489 |
| 192 | cg07039423 |
| 193 | cg17470251 |
| 194 | cg14010720 |
| 195 | cg16601494 |
| 196 | cg04518342 |
| 197 | cg17770910 |
| 198 | cg21974923 |
| 199 | cg02741836 |
| 200 | cg01263942 |
| 201 | cg21866259 |
| 202 | cg27541454 |
| 203 | cg01364755 |
| 204 | cg27364741 |
| 205 | cg18623980 |
| 206 | cg15949044 |
| 207 | cg08668790 |
| 208 | cg05480894 |
| 209 | cg04557057 |
| 210 | cg12463578 |
| 211 | cg04623837 |
| 212 | cg02032966 |
| 213 | cg04122873 |
| 214 | cg14904662 |
| 215 | cg27345924 |
| 216 | cg04117801 |
| 217 | cg16078649 |
| 218 | cg23018755 |
| 219 | cg01629716 |
| 220 | cg16394018 |
| 221 | cg20318143 |
| 222 | cg03323696 |
| 223 | cg26089877 |
| 224 | cg18131141 |
| 225 | cg17577862 |
| 226 | cg22352772 |
| 227 | cg18862481 |
| 228 | cg09998430 |
| 229 | cg21809927 |
| 230 | cg20673407 |
| 231 | cg23525438 |
| 232 | cg21545862 |
| 233 | cg20168806 |
| 234 | cg23671221 |
| 235 | cg26065488 |
| 236 | cg27188703 |
| 237 | cg04015962 |
| 238 | cg26107890 |
| 239 | cg08781140 |
| 240 | cg02447304 |
| 241 | cg07241660 |
| 242 | cg11379205 |
| 243 | cg24962381 |
| 244 | cg15384598 |
| 245 | cg06675248 |
| 246 | cg14780632 |
| 247 | cg03692651 |
| 248 | cg16202331 |
| 249 | cg21540621 |
| 250 | cg25243455 |
| 251 | cg16677019 |
| 252 | cg24676071 |
| 253 | cg23250574 |
| 254 | cg05856321 |
| 255 | cg14227325 |
| 256 | cg25538415 |
| 257 | cg17589883 |
| 258 | cg10682299 |
| 259 | cg07333715 |
| 260 | cg13990351 |
| 261 | cg07635227 |
| 262 | cg09979641 |
| 263 | cg20418529 |
| 264 | cg15751452 |
| 265 | cg13080565 |
| 266 | cg18213661 |
| 267 | cg20065768 |
| 268 | cg25145728 |
| 269 | cg00674679 |
| 270 | cg21577836 |
| 271 | cg10573232 |
| 272 | cg13877315 |
| 273 | cg01083804 |
| 274 | cg17534029 |
| 275 | cg00631329 |
| 276 | cg07455790 |
| 277 | cg13537353 |
| 278 | cg05875410 |
| 279 | cg13249146 |
| 280 | cg02286091 |
| 281 | cg07316846 |
| 282 | cg08477332 |
| 283 | cg02387803 |
| 284 | cg17024828 |
| 285 | cg02809746 |
| 286 | cg10393811 |
| 287 | cg22979783 |
| 288 | cg13912224 |
| 289 | cg05835105 |
| 290 | cg17560895 |
| 291 | cg12254845 |
| 292 | cg27594116 |
| 293 | cg08816590 |
| 294 | cg26081974 |
| 295 | cg24324815 |
| 296 | cg18476766 |
| 297 | cg09849405 |
| 298 | cg16737517 |
| 299 | cg22881914 |
| 300 | cg10372920 |
| 301 | cg13608733 |
| 302 | cg00399591 |
| 303 | cg15681626 |
| 304 | cg04787024 |
| 305 | cg24995678 |
| 306 | cg18430555 |
| 307 | cg20531550 |
| 308 | cg17415265 |
| 309 | cg02523640 |
| 310 | cg12559208 |
| 311 | cg18888655 |
| 312 | cg07110405 |
| 313 | cg09391546 |
| 314 | cg10400707 |
| 315 | cg16180556 |
| 316 | cg02519751 |
| 317 | cg04165291 |
| 318 | cg14263858 |
| 319 | cg24570841 |
| 320 | cg15219228 |
| 321 | cg01427108 |
| 322 | cg22274539 |
| 323 | cg17375267 |
| 324 | cg04552451 |
| 325 | cg24620761 |
| 326 | cg18365406 |
| 327 | cg03502002 |
| 328 | cg20261859 |
| 329 | cg15954353 |
| 330 | cg10930308 |
| 331 | cg05626376 |
| 332 | cg14680131 |
| 333 | cg16335762 |
| 334 | cg03234186 |
| 335 | cg21719957 |
| 336 | cg19006220 |
| 337 | cg06048910 |
| 338 | cg18477674 |
| 339 | cg20162381 |
| 340 | cg26580895 |
| 341 | cg00910015 |
| 342 | cg20771240 |
| 343 | cg03892838 |
| 344 | cg23971170 |
| 345 | cg23615741 |
| 346 | cg05496203 |
| 347 | cg24738311 |
| 348 | cg08543623 |
| 349 | cg00370123 |
| 350 | cg02155398 |
| 351 | cg05626536 |
| 352 | cg20497956 |
| 353 | cg25755575 |
| 354 | cg11112911 |
| 355 | cg21587238 |
| 356 | cg15788537 |
| 357 | cg06417478 |
| 358 | cg07981013 |
| 359 | cg26156687 |
| 360 | cg12506930 |
| 361 | cg05931423 |
| 362 | cg19239040 |
| 363 | cg00059870 |
| 364 | cg11431144 |
| 365 | cg22227603 |
| 366 | cg12176783 |
| 367 | cg21792155 |
| 368 | cg17022362 |
| 369 | cg16758314 |
| 370 | cg25095032 |
| 371 | cg10304637 |
| 372 | cg22941646 |
| 373 | cg27569446 |
| 374 | cg14395885 |
| 375 | cg07266404 |
| 376 | cg04180086 |
| 377 | cg10101533 |
| 378 | cg17128947 |
| 379 | cg03120555 |
| 380 | cg08867825 |
| 381 | cg01590338 |
| 382 | cg13207326 |
| 383 | cg00601042 |
| 384 | cg24685006 |
| 385 | cg26426745 |
| 386 | cg04156293 |
| 387 | cg26216513 |
| 388 | cg23278196 |
| 389 | cg09684233 |
| 390 | cg20699586 |
| 391 | cg12196045 |
| 392 | cg03188948 |
| 393 | cg12298268 |
| 394 | cg00035316 |
| 395 | cg03179291 |
| 396 | cg18530645 |
| 397 | cg21179088 |
| 398 | cg04052466 |
| 399 | cg10185505 |
| 400 | cg16823466 |
| 401 | cg01642521 |
| 402 | cg00157199 |
| 403 | cg12020396 |
| 404 | cg18179931 |
| 405 | cg16783349 |
| 406 | cg13283952 |
| 407 | cg14266287 |
| 408 | cg05638439 |
| 409 | cg17618327 |
| 410 | cg14134732 |
| 411 | cg25207771 |
| 412 | cg22727758 |
| 413 | cg15718581 |
| 414 | cg05864326 |
| 415 | cg18232235 |
| 416 | cg16210248 |
| 417 | cg19113906 |
| 418 | cg06112444 |
| 419 | cg15877520 |
| 420 | cg03947814 |
| 421 | cg01609658 |
| 422 | cg18566515 |
| 423 | cg21751684 |
| 424 | cg07709932 |
| 425 | cg18990050 |
| 426 | cg10308785 |
| 427 | cg18040901 |
| 428 | cg22400703 |
| 429 | cg13675721 |
| 430 | cg18063312 |
| 431 | cg21250296 |
| 432 | cg10020211 |
| 433 | cg15414638 |
| 434 | cg18091046 |
| 435 | cg09311683 |
| 436 | cg27453857 |
| 437 | cg11554566 |
| 438 | cg03020554 |
| 439 | cg11312408 |
| 440 | cg06093039 |
| 441 | cg06881093 |
| 442 | cg17713488 |
| 443 | cg21526750 |
| 444 | cg04987122 |
| 445 | cg00947782 |
| 446 | cg13721134 |
| 447 | cg06038358 |
| 448 | cg24122124 |
| 449 | cg05095318 |
| 450 | cg13476133 |
| 451 | cg21606928 |
| 452 | cg22710065 |
| 453 | cg14643892 |
| 454 | cg07616879 |
| 455 | cg08827579 |
| 456 | cg12620035 |
| 457 | cg13293524 |
| 458 | cg23731272 |
| 459 | cg24924502 |
| 460 | cg18735956 |
| 461 | cg16175245 |
| 462 | cg23729417 |
| 463 | cg02670123 |
| 464 | cg26492446 |
| 465 | cg18468354 |
| 466 | cg13595880 |
| 467 | cg02395917 |
| 468 | cg11940285 |
| 469 | cg14974749 |
| 470 | cg20219381 |
| 471 | cg01128482 |
| 472 | cg17627629 |
| 473 | cg11770080 |
| 474 | cg08103988 |
| 475 | cg17625381 |
| 476 | cg01295203 |
| 477 | cg24613080 |
| 478 | cg24080247 |
| 479 | cg19963856 |
| 480 | cg06346857 |
| 481 | cg08196032 |
| 482 | cg08822897 |
| 483 | cg10384245 |
| 484 | cg15690347 |
| 485 | cg00875805 |
| 486 | cg22511877 |
| 487 | cg16896868 |
| 488 | cg07300846 |
| 489 | cg26220594 |
| 490 | cg02961707 |
| 491 | cg08898155 |
| 492 | cg17446685 |
| 493 | cg16115627 |
| 494 | cg13498757 |
| 495 | cg19866866 |
| 496 | cg05663278 |
| 497 | cg16432061 |
| 498 | cg04921315 |
| 499 | cg00107982 |
| 500 | cg17754500 |
| 501 | cg07333231 |
| 502 | cg08586426 |
| 503 | cg13423262 |
| 504 | cg25556905 |
| 505 | cg22480783 |
| 506 | cg25551714 |
| 507 | cg18096722 |
| 508 | cg12270741 |
| 509 | cg14106234 |
| 510 | cg00767010 |
| 511 | cg01992382 |
| 512 | cg22679813 |
| 513 | cg06788790 |
| 514 | cg25210835 |
| 515 | cg09143673 |
| 516 | cg16940012 |
| 517 | cg02656871 |
| 518 | cg23736843 |
| 519 | cg10395685 |
| 520 | cg19059495 |
| 521 | cg17718679 |
| 522 | cg26764761 |
| 523 | cg26517663 |
| 524 | cg13744508 |
| 525 | cg24697911 |
| 526 | cg06852942 |
| 527 | cg02631468 |
| 528 | cg26479667 |
| 529 | cg25622366 |
| 530 | cg22016995 |
| 531 | cg08167951 |
| 532 | cg11375458 |
| 533 | cg22708150 |
| 534 | cg18517818 |
| 535 | cg12584100 |
| 536 | cg25461508 |
| 537 | cg13401893 |
| 538 | cg09237133 |
| 539 | cg16399136 |
| 540 | cg20737382 |
| 541 | cg18690769 |
| 542 | cg27411712 |
| 543 | cg21614638 |
| 544 | cg26031954 |
| 545 | cg07168142 |
| 546 | cg26872028 |
| 547 | cg02454488 |
| 548 | cg15613420 |
| 549 | cg19559537 |
| 550 | cg02737268 |
| 551 | cg16329658 |
| 552 | cg19923650 |
| 553 | cg05463589 |
| 554 | cg27594616 |
| 555 | cg18377161 |
| 556 | cg08141395 |
| 557 | cg14248715 |
| 558 | cg10336039 |
| 559 | cg23618477 |
| 560 | cg16318112 |
| 561 | cg20435485 |
| 562 | cg04908380 |
| 563 | cg14768245 |
| 564 | cg14121142 |
| 565 | cg19513321 |
| 566 | cg24274579 |
| 567 | cg12264626 |
| 568 | cg07532183 |
| 569 | cg23095743 |
| 570 | cg19814934 |
| 571 | cg13225830 |
| 572 | cg18402987 |
| 573 | cg06842954 |
| 574 | cg14168530 |
| 575 | cg18322510 |
| 576 | cg04534765 |
| 577 | cg25123362 |
| 578 | cg03900143 |
| 579 | cg19255477 |
| 580 | cg16440561 |
| 581 | cg01031101 |
| 582 | cg14621254 |
| 583 | cg04481096 |
| 584 | cg25487382 |
| 585 | cg09557462 |
| 586 | cg12765064 |
| 587 | cg04141796 |
| 588 | cg21757973 |
| 589 | cg02227036 |
| 590 | cg06501366 |
| 591 | cg00175573 |
| 592 | cg13446474 |
| 593 | cg11102724 |
| 594 | cg21485303 |
| 595 | cg12318501 |
| 596 | cg22747746 |
| 597 | cg13574488 |
| 598 | cg19418458 |
| 599 | cg26568372 |
| 600 | cg08291024 |
| 601 | cg19912142 |
| 602 | cg22852065 |
| 603 | cg10130564 |
| 604 | cg10092377 |
| 605 | cg01820213 |
| 606 | cg01969701 |
| 607 | cg19517686 |
| 608 | cg12964792 |
| 609 | cg10249637 |
| 610 | cg23497383 |
| 611 | cg23548969 |
| 612 | cg07774938 |
| 613 | cg10604476 |
| 614 | cg00970396 |
| 615 | cg18397450 |
| 616 | cg02511456 |
| 617 | cg27272853 |
| 618 | cg16288089 |
| 619 | cg20992181 |
| 620 | cg16497661 |
| 621 | cg24744430 |
| 622 | cg03671052 |
| 623 | cg14428146 |
| 624 | cg08034379 |
| 625 | cg01964152 |
| 626 | cg08763224 |
| 627 | cg22867893 |
| 628 | cg11608150 |
| 629 | cg10203211 |
| 630 | cg09065876 |
| 631 | cg01577475 |
| 632 | cg05345154 |
| 633 | cg02886375 |
| 634 | cg10009968 |
| 635 | cg03260530 |
| 636 | cg23332689 |
| 637 | cg07031408 |
| 638 | cg17790037 |
| 639 | cg19594218 |
| 640 | cg17280346 |
| 641 | cg05982460 |
| 642 | cg18684142 |
| 643 | cg00011616 |
| 644 | cg16214653 |
| 645 | cg21040094 |
| 646 | cg04029027 |
| 647 | cg21887193 |
| 648 | cg15401418 |
| 649 | cg10596483 |
| 650 | cg15856626 |
| 651 | cg23588462 |
| 652 | cg11946459 |
| 653 | cg11207307 |
| 654 | cg06470822 |
| 655 | cg13993183 |
| 656 | cg05732750 |
| 657 | cg19149132 |
| 658 | cg18047662 |
| 659 | cg24770181 |
| 660 | cg06632027 |
| 661 | cg20265803 |
| 662 | cg15391590 |
| 663 | cg04707519 |
| 664 | cg08274637 |
| 665 | cg15822765 |
| 666 | cg05241828 |
| 667 | cg18501555 |
| 668 | cg26860935 |
| 669 | cg12008047 |
| 670 | cg03290977 |
| 671 | cg25947619 |
| 672 | cg05060949 |
| 673 | cg13314145 |
| 674 | cg26354017 |
| 675 | cg01553231 |
| 676 | cg03711485 |
| 677 | cg13878677 |
| 678 | cg23987789 |
| 679 | cg19901523 |
| 680 | cg13699355 |
| 681 | cg04227892 |
| 682 | cg20974609 |
| 683 | cg24508426 |
| 684 | cg08500811 |
| 685 | cg01120173 |
| 686 | cg24051554 |
| 687 | cg03526459 |
| 688 | cg14506696 |
| 689 | cg05039666 |
| 690 | cg11236727 |
| 691 | cg05486035 |
| 692 | cg20408002 |
| 693 | cg11062778 |
| 694 | cg17533927 |
| 695 | cg23087153 |
| 696 | cg16393012 |
| 697 | cg22699026 |
| 698 | cg17342973 |
| 699 | cg10615591 |
| 700 | cg04729004 |
| 701 | cg20761810 |
| 702 | cg08330690 |
| 703 | cg24245418 |
| 704 | cg09052854 |
| 705 | cg10231412 |
| 706 | cg24628298 |
| 707 | cg11382963 |
| 708 | cg26019472 |
| 709 | cg15177489 |
| 710 | cg07350470 |
| 711 | cg27651243 |
| 712 | cg11868041 |
| 713 | cg16428612 |
| 714 | cg04248757 |
| 715 | cg03799405 |
| 716 | cg10863857 |
| 717 | cg20965743 |
| 718 | cg07262247 |
| 719 | cg00249511 |
| 720 | cg16585333 |
| 721 | cg23818870 |
| 722 | cg10640845 |
| 723 | cg07891531 |
| 724 | cg25561140 |
| 725 | cg26029864 |
| 726 | cg06890747 |
| 727 | cg22740796 |
| 728 | cg09048251 |
| 729 | cg01128460 |
| 730 | cg12227401 |
| 731 | cg16479539 |
| 732 | cg25457886 |
| 733 | cg04974290 |
| 734 | cg20646846 |
| 735 | cg26251325 |
| 736 | cg07366506 |
| 737 | cg05728019 |
| 738 | cg22943986 |
| 739 | cg16278107 |
| 740 | cg14005211 |
| 741 | cg07865607 |
| 742 | cg17134153 |
| 743 | cg13341668 |
| 744 | cg16546503 |
| 745 | cg01528052 |
| 746 | cg13530938 |
| 747 | cg19821361 |
| 748 | cg00934355 |
| 749 | cg08154437 |
| 750 | cg18588323 |
| 751 | cg22346476 |
| 752 | cg01419831 |
| 753 | cg20379954 |
| 754 | cg25942990 |
| 755 | cg07796782 |
| 756 | cg08880849 |
| 757 | cg22244192 |
| 758 | cg01678084 |
| 759 | cg09233651 |
| 760 | cg10608596 |
| 761 | cg08079908 |
| 762 | cg01246520 |
| 763 | cg10578777 |
| 764 | cg10374084 |
| 765 | cg10572355 |
| 766 | cg03962794 |
| 767 | cg15878670 |
| 768 | cg24186458 |
| 769 | cg13877670 |
| 770 | cg25650256 |
| 771 | cg22895981 |
| 772 | cg06437840 |
| 773 | cg12688965 |
| 774 | cg04648480 |
| 775 | cg12848808 |
| 776 | cg02428792 |
| 777 | cg08482837 |
| 778 | cg11510586 |
| 779 | cg17519645 |
| 780 | cg09628195 |
| 781 | cg26708319 |
| 782 | cg18717167 |
| 783 | cg19106932 |
| 784 | cg09788416 |
| 785 | cg12934366 |
| 786 | cg06226156 |
| 787 | cg26199445 |
| 788 | cg03993109 |
| 789 | cg26174216 |
| 790 | cg14602530 |
| 791 | cg13742526 |
| 792 | cg09040942 |
| 793 | cg05210266 |
| 794 | cg19047868 |
| 795 | cg16389705 |
| 796 | cg10705379 |
| 797 | cg02534659 |
| 798 | cg12172441 |
| 799 | cg16353006 |
| 800 | cg27137258 |
| 801 | cg02772121 |
| 802 | cg13414270 |
| 803 | cg06371502 |
| 804 | cg16664944 |
| 805 | cg10831607 |
| 806 | cg07551254 |
| 807 | cg12597389 |
| 808 | cg26493612 |
| 809 | cg22193912 |
| 810 | cg25774643 |
| 811 | cg09238332 |
| 812 | cg06527318 |
| 813 | cg00674706 |
| 814 | cg03940650 |
| 815 | cg17965945 |
| 816 | cg17173816 |
| 817 | cg14494721 |
| 818 | cg17917283 |
| 819 | cg12954512 |
| 820 | cg20574436 |
| 821 | cg12184886 |
| 822 | cg00779056 |
| 823 | cg23931487 |
| 824 | cg21143896 |
| 825 | cg03946671 |
| 826 | cg00689492 |
| 827 | cg23022851 |
| 828 | cg18023065 |
| 829 | cg23168425 |
| 830 | cg05408831 |
| 831 | cg14089267 |
| 832 | cg21784383 |
| 833 | cg06460944 |
| 834 | cg23928920 |
| 835 | cg01329005 |
| 836 | cg20415486 |
| 837 | cg07299736 |
| 838 | cg10791959 |
| 839 | cg14029489 |
| 840 | cg25191041 |
| 841 | cg12019614 |
| 842 | cg03323067 |
| 843 | cg16915828 |
| 844 | cg12333160 |
| 845 | cg05166138 |
| 846 | cg26053291 |
| 847 | cg19563510 |
| 848 | cg09706122 |
| 849 | cg02276807 |
| 850 | cg06962944 |
| 851 | cg22851944 |
| 852 | cg27655158 |
| 853 | cg01564640 |
| 854 | cg02299497 |
| 855 | cg23198707 |
| 856 | cg17495087 |
| 857 | cg20002209 |
| 858 | cg25032094 |
| 859 | cg03576469 |
| 860 | cg04037038 |
| 861 | cg25286482 |
| 862 | cg09329079 |
| 863 | cg12121643 |
| 864 | cg01214054 |
| 865 | cg15532640 |
| 866 | cg11772171 |
| 867 | cg01102073 |
| 868 | cg16236270 |
| 869 | cg23936031 |
| 870 | cg12091642 |
| 871 | cg19397693 |
| 872 | cg26550235 |
| 873 | cg13335775 |
| 874 | cg07838270 |
| 875 | cg22579265 |
| 876 | cg12479878 |
| 877 | cg06609094 |
| 878 | cg19749898 |
| 879 | cg21104965 |
| 880 | cg18850127 |
| 881 | cg14101485 |
| 882 | cg21082028 |
| 883 | cg13924715 |
| 884 | cg09451235 |
| 885 | cg22254463 |
| 886 | cg02477557 |
| 887 | cg06545761 |
| 888 | cg18235734 |
| 889 | cg12280664 |
| 890 | cg18268547 |
| 891 | cg09957864 |
| 892 | cg02119792 |
| 893 | cg00492719 |
| 894 | cg19926444 |
| 895 | cg14457782 |
| 896 | cg15555527 |
| 897 | cg03498081 |
| 898 | cg23792314 |
| 899 | cg01833699 |
| 900 | cg22817352 |
| 901 | cg21935981 |
| 902 | cg24064594 |
| 903 | cg01075271 |
| 904 | cg12898220 |
| 905 | cg18470427 |
| 906 | cg24028634 |
| 907 | cg24709951 |
| 908 | cg01509330 |
| 909 | cg09101605 |
| 910 | cg10218000 |
| 911 | cg10722799 |
| 912 | cg20302133 |
| 913 | cg13578134 |
| 914 | cg13555283 |
| 915 | cg22848598 |
| 916 | cg09026722 |
| 917 | cg02671880 |
| 918 | cg12116288 |
| 919 | cg12633154 |
| 920 | cg00422488 |
| 921 | cg15855767 |
| 922 | cg09557313 |
| 923 | cg22604218 |
| 924 | cg08812108 |
| 925 | cg08675941 |
| 926 | cg13023646 |
| 927 | cg11616651 |
| 928 | cg22344122 |
| 929 | cg26579578 |
| 930 | cg06150841 |
| 931 | cg09858108 |
| 932 | cg04069472 |
| 933 | cg05949331 |
| 934 | cg04056576 |
| 935 | cg13213009 |
| 936 | cg15845792 |
| 937 | cg12014181 |
| 938 | cg22260869 |
| 939 | cg10532489 |
| 940 | cg12648510 |
| 941 | cg02440420 |
| 942 | cg11086982 |
| 943 | cg17873048 |
| 944 | cg13568106 |
| 945 | cg04030486 |
| 946 | cg21279510 |
| 947 | cg00655552 |
| 948 | cg14008271 |
| 949 | cg12140851 |
| 950 | cg07970799 |
| 951 | cg10397389 |
| 952 | cg23549902 |
| 953 | cg04935434 |
| 954 | cg02886033 |
| 955 | cg18631330 |
| 956 | cg25506065 |
| 957 | cg17771031 |
| 958 | cg11669516 |
| 959 | cg18537730 |
| 960 | cg13446584 |
| 961 | cg26650638 |
| 962 | cg26599630 |
| 963 | cg15978561 |
| 964 | cg01051240 |
| 965 | cg02023345 |
| 966 | cg12480115 |
| 967 | cg05884870 |
| 968 | cg10453419 |
| 969 | cg00116092 |
| 970 | cg13935437 |
| 971 | cg26113809 |
| 972 | cg01189072 |
| 973 | cg23817893 |
| 974 | cg01140515 |
| 975 | cg08234689 |
| 976 | cg06800235 |
| 977 | cg27317046 |
| 978 | cg12519783 |
| 979 | cg24181266 |
| 980 | cg01176363 |
| 981 | cg11474464 |
| 982 | cg05280806 |
| 983 | cg11362010 |
| 984 | cg15672437 |
| 985 | cg23927970 |
| 986 | cg11763830 |
| 987 | cg18693673 |
| 988 | cg15232539 |
| 989 | cg05159188 |
| 990 | cg10259889 |
| 991 | cg20555674 |
| 992 | cg11173579 |
| 993 | cg24496475 |
| 994 | cg13644317 |
| 995 | cg05934682 |
| 996 | cg00044796 |
| 997 | cg21974358 |
| 998 | cg26258846 |
| 999 | cg00508283 |
| 1000 | cg22164298 |
| 1001 | cg08920174 |
| 1002 | cg24751154 |
| 1003 | cg26187313 |
| 1004 | cg08574915 |
| 1005 | cg19093370 |
| 1006 | cg26090849 |
| 1007 | cg24035245 |
| 1008 | cg16734433 |
| 1009 | cg05267394 |
| 1010 | cg18516557 |
| 1011 | cg05196969 |
| 1012 | cg17370927 |
| 1013 | cg03252770 |
| 1014 | cg21165486 |
| 1015 | cg06992285 |
| 1016 | cg14454907 |
| 1017 | cg22070991 |
| 1018 | cg11476737 |
| 1019 | cg05517610 |
| 1020 | cg05409540 |
| 1021 | cg07591442 |
| 1022 | cg15666214 |
| 1023 | cg02408153 |
| 1024 | cg08032924 |
| 1025 | cg25480785 |
| 1026 | cg00962755 |
| 1027 | cg27295595 |
| 1028 | cg25809561 |
| 1029 | cg01335367 |
| 1030 | cg15469272 |
| 1031 | cg04142864 |
| 1032 | cg20676602 |
| 1033 | cg23156509 |
| 1034 | cg01554437 |
| 1035 | cg12459718 |
| 1036 | cg26109145 |
| 1037 | cg24839386 |
| 1038 | cg18002126 |
| 1039 | cg21193415 |
| 1040 | cg26472133 |
| 1041 | cg06728252 |
| 1042 | cg15278109 |
| 1043 | cg06529528 |
| 1044 | cg13818451 |
| 1045 | cg18244358 |
| 1046 | cg24589459 |
| 1047 | cg07297896 |
| 1048 | cg09614415 |
| 1049 | cg16043651 |
| 1050 | cg27395066 |
| 1051 | cg04863758 |
| 1052 | cg08041448 |
| 1053 | cg14160419 |
| 1054 | cg15988801 |
| 1055 | cg08855938 |
| 1056 | cg21139150 |
| 1057 | cg11988036 |
| 1058 | cg03771840 |
| 1059 | cg23931360 |
| 1060 | cg20935165 |
| 1061 | cg12334869 |
| 1062 | cg25806701 |
| 1063 | cg03858673 |
| 1064 | cg01103387 |
| 1065 | cg08474521 |
| 1066 | cg10082647 |
| 1067 | cg03598731 |
| 1068 | cg27109748 |
| 1069 | cg08193579 |
| 1070 | cg14708893 |
| 1071 | cg00083790 |
| 1072 | cg07126690 |
| 1073 | cg04705669 |
| 1074 | cg02264990 |
| 1075 | cg23780110 |
| 1076 | cg24245216 |
| 1077 | cg09677945 |
| 1078 | cg25979543 |
| 1079 | cg03265671 |
| 1080 | cg21361094 |
| 1081 | cg20927731 |
| 1082 | cg19802390 |
| 1083 | cg21729992 |
| 1084 | cg25392692 |
| 1085 | cg24467349 |
| 1086 | cg13759674 |
| 1087 | cg26149875 |
| 1088 | cg16889557 |
| 1089 | cg15924868 |
| 1090 | cg20786074 |
| 1091 | cg20467929 |
| 1092 | cg04329125 |
| 1093 | cg26858540 |
| 1094 | cg10905418 |
| 1095 | cg21406271 |
| 1096 | cg06358671 |
| 1097 | cg19718882 |
| 1098 | cg25040225 |
| 1099 | cg21932416 |
| 1100 | cg26957636 |
| 1101 | cg02139965 |
| 1102 | cg16247183 |
| 1103 | cg11546385 |
| 1104 | cg01435308 |
| 1105 | cg14858993 |
| 1106 | cg15135047 |
| 1107 | cg22289831 |
| 1108 | cg21678378 |
| 1109 | cg21191514 |
| 1110 | cg00440468 |
| 1111 | cg07171527 |
| 1112 | cg25693302 |
| 1113 | cg08757862 |
| 1114 | cg07459252 |
| 1115 | cg25536739 |
| 1116 | cg01787574 |
| 1117 | cg13113121 |
| 1118 | cg01510388 |
| 1119 | cg14676825 |
| 1120 | cg10322504 |
| 1121 | cg18861197 |
| 1122 | cg02622316 |
| 1123 | cg03605610 |
| 1124 | cg13791254 |
| 1125 | cg04046669 |
| 1126 | cg24479859 |
| 1127 | cg02907049 |
| 1128 | cg18732172 |
| 1129 | cg04419557 |
| 1130 | cg08738570 |
| 1131 | cg20884887 |
| 1132 | cg13645732 |
| 1133 | cg00584026 |
| 1134 | cg26383138 |
| 1135 | cg22477343 |
| 1136 | cg22978940 |
| 1137 | cg14765731 |
| 1138 | cg25221984 |
| 1139 | cg00711072 |
| 1140 | cg26732615 |
| 1141 | cg05554189 |
| 1142 | cg15545410 |
| 1143 | cg22151446 |
| 1144 | cg06048004 |
| 1145 | cg10530883 |
| 1146 | cg23194912 |
| 1147 | cg01407254 |
| 1148 | cg22100019 |
| 1149 | cg15077193 |
| 1150 | cg13023205 |
| 1151 | cg06489993 |
| 1152 | cg26719062 |
| 1153 | cg03601886 |
| 1154 | cg17560015 |
| 1155 | cg10134833 |
| 1156 | cg14530382 |
| 1157 | cg20773915 |
| 1158 | cg03048654 |
| 1159 | cg05234121 |
| 1160 | cg05314679 |
| 1161 | cg02540781 |
| 1162 | cg24923430 |
| 1163 | cg21047695 |
| 1164 | cg13469457 |
| 1165 | cg10962762 |
| 1166 | cg18419977 |
| 1167 | cg19074709 |
| 1168 | cg20339868 |
| 1169 | cg12377220 |
| 1170 | cg17518842 |
| 1171 | cg10534455 |
| 1172 | cg00761644 |
| 1173 | cg25417842 |
| 1174 | cg23541975 |
| 1175 | cg16312002 |
| 1176 | cg14482968 |
| 1177 | cg04656042 |
| 1178 | cg13469595 |
| 1179 | cg07356283 |
| 1180 | cg03343571 |
| 1181 | cg04546413 |
| 1182 | cg08431536 |
| 1183 | cg09232478 |
| 1184 | cg07815009 |
| 1185 | cg00384539 |
| 1186 | cg13471599 |
| 1187 | cg26843227 |
| 1188 | cg16440058 |
| 1189 | cg27312173 |
| 1190 | cg08271443 |
| 1191 | cg27079118 |
| 1192 | cg08632909 |
| 1193 | cg25950112 |
| 1194 | cg05327835 |
| 1195 | cg26465391 |
| 1196 | cg17266581 |
| 1197 | cg12174341 |
| 1198 | cg09372263 |
| 1199 | cg07178969 |
| 1200 | cg01006802 |
| 1201 | cg16085144 |
| 1202 | cg16204818 |
| 1203 | cg20248748 |
| 1204 | cg16265599 |
| 1205 | cg23781022 |
| 1206 | cg08965685 |
| 1207 | cg17581065 |
| 1208 | cg16996144 |
| 1209 | cg19596110 |
| 1210 | cg16113156 |
| 1211 | cg23291280 |
| 1212 | cg01815671 |
| 1213 | cg01689438 |
| 1214 | cg01783662 |
| 1215 | cg13999701 |
| 1216 | cg12063490 |
| 1217 | cg26924440 |
| 1218 | cg14802502 |
| 1219 | cg11986813 |
| 1220 | cg12297590 |
| 1221 | cg10236239 |
| 1222 | cg04344111 |
| 1223 | cg13110775 |
| 1224 | cg02486253 |
| 1225 | cg09540471 |
| 1226 | cg06740765 |
| 1227 | cg19637330 |
| 1228 | cg24915592 |
| 1229 | cg05482942 |
| 1230 | cg15450139 |
| 1231 | cg17494781 |
| 1232 | cg05057910 |
| 1233 | cg00223681 |
| 1234 | cg18781988 |
| 1235 | cg24624353 |
| 1236 | cg25730564 |
| 1237 | cg14245034 |
| 1238 | cg06623935 |
| 1239 | cg02374745 |
| 1240 | cg26091021 |
| 1241 | cg00104845 |
| 1242 | cg01895649 |
| 1243 | cg10203610 |
| 1244 | cg22645427 |
| 1245 | cg05253110 |
| 1246 | cg15649236 |
| 1247 | cg08787837 |
| 1248 | cg03339817 |
| 1249 | cg02737619 |
| 1250 | cg08957069 |
| 1251 | cg19394642 |
| 1252 | cg04184297 |
| 1253 | cg27086497 |
| 1254 | cg12649455 |
| 1255 | cg01702055 |
| 1256 | cg08123444 |
| 1257 | cg17898329 |
| 1258 | cg06776703 |
| 1259 | cg24006505 |
| 1260 | cg08072862 |
| 1261 | cg18892244 |
| 1262 | cg07204280 |
| 1263 | cg09808639 |
| 1264 | cg06091539 |
| 1265 | cg11331445 |
| 1266 | cg18675097 |
| 1267 | cg14282632 |
| 1268 | cg17479501 |
| 1269 | cg03826642 |
| 1270 | cg12827262 |
| 1271 | cg10621924 |
| 1272 | cg20060268 |
| 1273 | cg19317211 |
| 1274 | cg19391924 |
| 1275 | cg08202754 |
| 1276 | cg23261919 |
| 1277 | cg08047457 |
| 1278 | cg18464558 |
| 1279 | cg02707854 |
| 1280 | cg02991085 |
| 1281 | cg15431103 |
| 1282 | cg25371919 |
| 1283 | cg08085267 |
| 1284 | cg10069155 |
| 1285 | cg03704250 |
| 1286 | cg15236483 |
| 1287 | cg26337312 |
| 1288 | cg02210934 |
| 1289 | cg11989256 |
| 1290 | cg20073553 |
| 1291 | cg23216398 |
| 1292 | cg10129154 |
| 1293 | cg05180208 |
| 1294 | cg25834768 |
| 1295 | cg27642470 |
| 1296 | cg09591072 |
| 1297 | cg01572696 |
| 1298 | cg11245681 |
| 1299 | cg19378036 |
| 1300 | cg06580551 |
| 1301 | cg11916065 |
| 1302 | cg26716902 |
| 1303 | cg19000885 |
| 1304 | cg15346191 |
| 1305 | cg04685170 |
| 1306 | cg23200760 |
| 1307 | cg01226963 |
| 1308 | cg13487211 |
| 1309 | cg21226965 |
| 1310 | cg03232056 |
| 1311 | cg15975865 |
| 1312 | cg04225785 |
| 1313 | cg05589489 |
| 1314 | cg11122009 |
| 1315 | cg04270401 |
| 1316 | cg26536894 |
| 1317 | cg14435644 |
| 1318 | cg17071446 |
| 1319 | cg04003850 |
| 1320 | cg25272143 |
| 1321 | cg23456304 |
| 1322 | cg09279736 |
| 1323 | cg20743236 |
| 1324 | cg17109042 |
| 1325 | cg12859802 |
| 1326 | cg19290577 |
| 1327 | cg09112081 |
| 1328 | cg03119043 |
| 1329 | cg20383948 |
| 1330 | cg03979241 |
| 1331 | cg05783139 |
| 1332 | cg15124400 |
| 1333 | cg11994863 |
| 1334 | cg19457477 |
| 1335 | cg19241679 |
| 1336 | cg16088380 |
| 1337 | cg19580967 |
| 1338 | cg20824989 |
| 1339 | cg10531725 |
| 1340 | cg24369728 |
| 1341 | cg12495829 |
| 1342 | cg24503407 |
| 1343 | cg00626856 |
| 1344 | cg17471939 |
| 1345 | cg15469871 |
| 1346 | cg17746570 |
| 1347 | cg05192138 |
| 1348 | cg24854181 |
| 1349 | cg14015441 |
| 1350 | cg23902113 |
| 1351 | cg00054210 |
| 1352 | cg11260046 |
| 1353 | cg05339114 |
| 1354 | cg09379609 |
| 1355 | cg24113818 |
| 1356 | cg07195224 |
| 1357 | cg14751182 |
| 1358 | cg05937292 |
| 1359 | cg08189638 |
| 1360 | cg12997720 |
| 1361 | cg08119647 |
| 1362 | cg08326019 |
| 1363 | cg25078649 |
| 1364 | cg24852548 |
| 1365 | cg16501237 |
| 1366 | cg14196395 |
| 1367 | cg18004701 |
| 1368 | cg15712777 |
| 1369 | cg26070874 |
| 1370 | cg06172942 |
| 1371 | cg07533224 |
| 1372 | cg00777178 |
| 1373 | cg25663755 |
| 1374 | cg18290075 |
| 1375 | cg04703620 |
| 1376 | cg18635497 |
| 1377 | cg14671384 |
| 1378 | cg23207990 |
| 1379 | cg25576011 |
| 1380 | cg14691671 |
| 1381 | cg19907915 |
| 1382 | cg13682333 |
| 1383 | cg24808280 |
| 1384 | cg02744524 |
| 1385 | cg19759129 |
| 1386 | cg13736514 |
| 1387 | cg19867917 |
| 1388 | cg15663629 |
| 1389 | cg00939682 |
| 1390 | cg25503903 |
| 1391 | cg20740434 |
| 1392 | cg23981871 |
| 1393 | cg02349468 |
| 1394 | cg15222899 |
| 1395 | cg19011590 |
| 1396 | cg03451670 |
| 1397 | cg15165122 |
| 1398 | cg10296238 |
| 1399 | cg01079515 |
| 1400 | cg21723903 |
| 1401 | cg04206484 |
| 1402 | cg12167907 |
| 1403 | cg04184019 |
| 1404 | cg25509184 |
| 1405 | cg22234712 |
| 1406 | cg18324144 |
| 1407 | cg17646820 |
| 1408 | cg16179938 |
| 1409 | cg06951626 |
| 1410 | cg22928386 |
| 1411 | cg15854847 |
| 1412 | cg06463958 |
| 1413 | cg20050477 |
| 1414 | cg24856447 |
| 1415 | cg21634283 |
| 1416 | cg06334737 |
| 1417 | cg00667789 |
| 1418 | cg15423654 |
| 1419 | cg22702070 |
| 1420 | cg03926815 |
| 1421 | cg14132888 |
| 1422 | cg03302259 |
| 1423 | cg02469909 |
| 1424 | cg14919082 |
| 1425 | cg06234051 |
| 1426 | cg12444411 |
| 1427 | cg01218619 |
| 1428 | cg16626067 |
| 1429 | cg13371260 |
| 1430 | cg07611519 |
| 1431 | cg09392827 |
| 1432 | cg02925601 |
| 1433 | cg06546677 |
| 1434 | cg18732257 |
| 1435 | cg16797735 |
| 1436 | cg00124375 |
| 1437 | cg21497990 |
| 1438 | cg27138204 |
| 1439 | cg19259203 |
| 1440 | cg21849932 |
| 1441 | cg08330950 |
| 1442 | cg14081667 |
| 1443 | cg04495491 |
| 1444 | cg05758978 |
| 1445 | cg21327194 |
| 1446 | cg04841825 |
| 1447 | cg15755348 |
| 1448 | cg17759274 |
| 1449 | cg09193477 |
| 1450 | cg20552263 |
| 1451 | cg05659097 |
| 1452 | cg16297011 |
| 1453 | cg05347673 |
| 1454 | cg14578677 |
| 1455 | cg07307078 |
| 1456 | cg19182683 |
| 1457 | cg13175778 |
| 1458 | cg18331249 |
| 1459 | cg14253448 |
| 1460 | cg23357832 |
| 1461 | cg26014185 |
| 1462 | cg10285466 |
| 1463 | cg05991492 |
| 1464 | cg19389372 |
| 1465 | cg01221209 |
| 1466 | cg18447972 |
| 1467 | cg06765219 |
| 1468 | cg14716990 |
| 1469 | cg25685519 |
| 1470 | cg10191240 |
| 1471 | cg17455155 |
| 1472 | cg04786931 |
| 1473 | cg23727983 |
| 1474 | cg26446832 |
| 1475 | cg03719155 |
| 1476 | cg22855933 |
| 1477 | cg24996981 |
| 1478 | cg04439806 |
| 1479 | cg21277995 |
| 1480 | cg24719399 |
| 1481 | cg14308215 |
| 1482 | cg19096571 |
| 1483 | cg06145435 |
| 1484 | cg19865237 |
| 1485 | cg16329197 |
| 1486 | cg26730416 |
| 1487 | cg04960065 |
| 1488 | cg10783469 |
| 1489 | cg26515501 |
| 1490 | cg12327866 |
| 1491 | cg21697381 |
| 1492 | cg24115232 |
| 1493 | cg13779009 |
| 1494 | cg19563049 |
| 1495 | cg05672146 |
| 1496 | cg09115473 |
| 1497 | cg08217716 |
| 1498 | cg08142884 |
| 1499 | cg25736251 |
| 1500 | cg10274022 |
| 1501 | cg05770238 |
| 1502 | cg03653113 |
| 1503 | cg01413582 |
| 1504 | cg17552357 |
| 1505 | cg12883629 |
| 1506 | cg03448068 |
| 1507 | cg14133557 |
| 1508 | cg02548132 |
| 1509 | cg08980987 |
| 1510 | cg05393297 |
| 1511 | cg12686441 |
| 1512 | cg04724406 |
| 1513 | cg22581896 |
| 1514 | cg00259834 |
| 1515 | cg19864808 |
| 1516 | cg11796455 |
| 1517 | cg11523350 |
| 1518 | cg19986012 |
| 1519 | cg11630242 |
| 1520 | cg12473912 |
| 1521 | cg18525352 |
| 1522 | cg15255390 |
| 1523 | cg26777729 |
| 1524 | cg25025879 |
| 1525 | cg02642017 |
| 1526 | cg03409187 |
| 1527 | cg24704344 |
| 1528 | cg18760534 |
| 1529 | cg16848712 |
| 1530 | cg21350324 |
| 1531 | cg18869404 |
| 1532 | cg01620164 |
| 1533 | cg21230493 |
| 1534 | cg03107235 |
| 1535 | cg10876767 |
| 1536 | cg10107890 |
| 1537 | cg04679040 |
| 1538 | cg03043444 |
| 1539 | cg25511667 |
| 1540 | cg02497747 |
| 1541 | cg17326555 |
| 1542 | cg02669047 |
| 1543 | cg12375870 |
| 1544 | cg09660151 |
| 1545 | cg24794228 |
| 1546 | cg13647536 |
| 1547 | cg11117438 |
| 1548 | cg22563535 |
| 1549 | cg21546184 |
| 1550 | cg15754594 |
| 1551 | cg15140721 |
| 1552 | cg08853572 |
| 1553 | cg06148736 |
| 1554 | cg08247202 |
| 1555 | cg11635421 |
| 1556 | cg25150953 |
| 1557 | cg24550112 |
| 1558 | cg05348366 |
| 1559 | cg01159194 |
| 1560 | cg01422370 |
| 1561 | cg13845094 |
| 1562 | cg13899478 |
| 1563 | cg14893161 |
| 1564 | cg25481455 |
| 1565 | cg20698421 |
| 1566 | cg17219660 |
| 1567 | cg05194426 |
| 1568 | cg12409982 |
| 1569 | cg13918754 |
| 1570 | cg17774559 |
| 1571 | cg12977384 |
| 1572 | cg24368383 |
| 1573 | cg04202892 |
| 1574 | cg04800503 |
| 1575 | cg24485957 |
| 1576 | cg06873916 |
| 1577 | cg00566297 |
| 1578 | cg22249529 |
| 1579 | cg19726408 |
| 1580 | cg06833564 |
| 1581 | cg11775488 |
| 1582 | cg10370540 |
| 1583 | cg24859722 |
| 1584 | cg05656990 |
| 1585 | cg22507154 |
| 1586 | cg23759393 |
| 1587 | cg26221801 |
| 1588 | cg11322797 |
| 1589 | cg13094191 |
| 1590 | cg07205807 |
| 1591 | cg00715929 |
| 1592 | cg16406892 |
| 1593 | cg19498960 |
| 1594 | cg23520096 |
| 1595 | cg27250032 |
| 1596 | cg27255388 |
| 1597 | cg05547778 |
| 1598 | cg08753890 |
| 1599 | cg06582782 |
| 1600 | cg03625109 |
| 1601 | cg06622408 |
| 1602 | cg08239499 |
| 1603 | cg15941241 |
| 1604 | cg06965300 |
| 1605 | cg02380585 |
| 1606 | cg08364137 |
| 1607 | cg16071993 |
| 1608 | cg10329418 |
| 1609 | cg08801479 |
| 1610 | cg04752871 |
| 1611 | cg01884681 |
| 1612 | cg02452725 |
| 1613 | cg01540102 |
| 1614 | cg14659662 |
| 1615 | cg04194947 |
| 1616 | cg03295090 |
| 1617 | cg19521279 |
| 1618 | cg21156812 |
| 1619 | cg23744638 |
| 1620 | cg11964308 |
| 1621 | cg03113878 |
| 1622 | cg20415053 |
| 1623 | cg26674943 |
| 1624 | cg05141014 |
| 1625 | cg25428177 |
| 1626 | cg15720089 |
| 1627 | cg20677205 |
| 1628 | cg18056484 |
| 1629 | cg18686665 |
| 1630 | cg06048819 |
| 1631 | cg22775138 |
| 1632 | cg03982376 |
| 1633 | cg17884327 |
| 1634 | cg11414921 |
| 1635 | cg25147604 |
| 1636 | cg19759481 |
| 1637 | cg10544205 |
| 1638 | cg26799656 |
| 1639 | cg11840968 |
| 1640 | cg23732024 |
| 1641 | cg21699330 |
| 1642 | cg01418086 |
| 1643 | cg03287198 |
| 1644 | cg01989508 |
| 1645 | cg26336642 |
| 1646 | cg26507839 |
| 1647 | cg09081233 |
| 1648 | cg25762522 |
| 1649 | cg19366458 |
| 1650 | cg02689489 |
| 1651 | cg17173975 |
| 1652 | cg23007087 |
| 1653 | cg11756095 |
| 1654 | cg08928753 |
| 1655 | cg25610515 |
| 1656 | cg15628518 |
| 1657 | cg17730961 |
| 1658 | cg04738965 |
| 1659 | cg16674248 |
| 1660 | cg07833564 |
| 1661 | cg22714290 |
| 1662 | cg01633902 |
| 1663 | cg07668501 |
| 1664 | cg19665696 |
| 1665 | cg24989962 |
| 1666 | cg27388680 |
| 1667 | cg14875327 |
| 1668 | cg17437787 |
| 1669 | cg15454820 |
| 1670 | cg09015973 |
| 1671 | cg26286839 |
| 1672 | cg25351606 |
| 1673 | cg14137381 |
| 1674 | cg09101004 |
| 1675 | cg05121006 |
| 1676 | cg25222010 |
| 1677 | cg09333785 |
| 1678 | cg08631502 |
| 1679 | cg03760457 |
| 1680 | cg16458866 |
| 1681 | cg06890619 |
| 1682 | cg20558320 |
| 1683 | cg10476773 |
| 1684 | cg13022624 |
| 1685 | cg10510935 |
| 1686 | cg04832450 |
| 1687 | cg17048103 |
| 1688 | cg14666564 |
| 1689 | cg22796922 |
| 1690 | cg12120033 |
| 1691 | cg17565627 |
| 1692 | cg15353501 |
| 1693 | cg17010100 |
| 1694 | cg06355422 |
| 1695 | cg22964184 |
| 1696 | cg26590664 |
| 1697 | cg18132363 |
| 1698 | cg09451457 |
| 1699 | cg01181227 |
| 1700 | cg23807071 |
| 1701 | cg07157398 |
| 1702 | cg12326558 |
| 1703 | cg07197785 |
| 1704 | cg10848272 |
| 1705 | cg07155223 |
| 1706 | cg21859594 |
| 1707 | cg04114897 |
| 1708 | cg14275423 |
| 1709 | cg03364108 |
| 1710 | cg22178798 |
| 1711 | cg24688309 |
| 1712 | cg20964064 |
| 1713 | cg19685627 |
| 1714 | cg07156249 |
| 1715 | cg02272993 |
| 1716 | cg06335123 |
| 1717 | cg02460251 |
| 1718 | cg00634475 |
| 1719 | cg15868302 |
| 1720 | cg12880095 |
| 1721 | cg01156747 |
| 1722 | cg14016554 |
| 1723 | cg11668813 |
| 1724 | cg05904716 |
| 1725 | cg26547443 |
| 1726 | cg16618104 |
| 1727 | cg06247406 |
| 1728 | cg24626659 |
| 1729 | cg08077337 |
| 1730 | cg22685807 |
| 1731 | cg05640189 |
| 1732 | cg20949951 |
| 1733 | cg16936421 |
| 1734 | cg14415160 |
| 1735 | cg12039583 |
| 1736 | cg18154283 |
| 1737 | cg00401797 |
| 1738 | cg20092736 |
| 1739 | cg19112186 |
| 1740 | cg16554164 |
| 1741 | cg16603012 |
| 1742 | cg06221449 |
| 1743 | cg20216069 |
| 1744 | cg21167269 |
| 1745 | cg09354556 |
| 1746 | cg17865555 |
| 1747 | cg25695610 |
| 1748 | cg02977812 |
| 1749 | cg04851639 |
| 1750 | cg09547119 |
| 1751 | cg04579398 |
| 1752 | cg01140660 |
| 1753 | cg06125825 |
| 1754 | cg10363495 |
| 1755 | cg20477461 |
| 1756 | cg11428546 |
| 1757 | cg05578840 |
| 1758 | cg27005749 |
| 1759 | cg26590744 |
| 1760 | cg16206813 |
| 1761 | cg25207147 |
| 1762 | cg20305683 |
| 1763 | cg06658404 |
| 1764 | cg06542648 |
| 1765 | cg11679455 |
| 1766 | cg24975183 |
| 1767 | cg06226630 |
| 1768 | cg10745499 |
| 1769 | cg13375589 |
| 1770 | cg10738933 |
| 1771 | cg20909234 |
| 1772 | cg07778029 |
| 1773 | cg02017926 |
| 1774 | cg16375290 |
| 1775 | cg10377582 |
| 1776 | cg18838431 |
| 1777 | cg22821981 |
| 1778 | cg10242602 |
| 1779 | cg07192718 |
| 1780 | cg13382769 |
| 1781 | cg24894107 |
| 1782 | cg09655403 |
| 1783 | cg12268637 |
| 1784 | cg07945733 |
| 1785 | cg21570209 |
| 1786 | cg12118013 |
| 1787 | cg11450149 |
| 1788 | cg24521929 |
| 1789 | cg08893839 |
| 1790 | cg08854316 |
| 1791 | cg09069900 |
| 1792 | cg05048976 |
| 1793 | cg23245007 |
| 1794 | cg06445961 |
| 1795 | cg18575532 |
| 1796 | cg11168433 |
| 1797 | cg23817981 |
| 1798 | cg13040921 |
| 1799 | cg02199580 |
| 1800 | cg09518226 |
| 1801 | cg17344516 |
| 1802 | cg06106599 |
| 1803 | cg27177554 |
| 1804 | cg03683199 |
| 1805 | cg15028458 |
| 1806 | cg25348655 |
| 1807 | cg10840277 |
| 1808 | cg11413133 |
| 1809 | cg03682719 |
| 1810 | cg27179622 |
| 1811 | cg03669782 |
| 1812 | cg00011113 |
| 1813 | cg13949829 |
| 1814 | cg25863289 |
| 1815 | cg09841842 |
| 1816 | cg13390867 |
| 1817 | cg25292550 |
| 1818 | cg10839823 |
| 1819 | cg23133355 |
| 1820 | cg09001939 |
| 1821 | cg16859884 |
| 1822 | cg15035774 |
| 1823 | cg20071868 |
| 1824 | cg16020346 |
| 1825 | cg00492979 |
| 1826 | cg07185131 |
| 1827 | cg24174020 |
| 1828 | cg14654886 |
| 1829 | cg13379325 |
| 1830 | cg18244817 |
| 1831 | cg24361201 |
| 1832 | cg11339964 |
| 1833 | cg00217080 |
| 1834 | cg23139521 |
| 1835 | cg13888593 |
| 1836 | cg15370163 |
| 1837 | cg24940967 |
| 1838 | cg00759741 |
| 1839 | cg24659037 |
| 1840 | cg13359332 |
| 1841 | cg26266427 |
| 1842 | cg00745181 |
| 1843 | cg20784768 |
| 1844 | cg24536349 |
| 1845 | cg14108567 |
| 1846 | cg21581504 |
| 1847 | cg23786580 |
| 1848 | cg17210933 |
| 1849 | cg01064683 |
| 1850 | cg07887002 |
| 1851 | cg01315092 |
| 1852 | cg25451958 |
| 1853 | cg05634428 |
| 1854 | cg09238999 |
| 1855 | cg24122498 |
| 1856 | cg07167872 |
| 1857 | cg07097725 |
| 1858 | cg17024593 |
| 1859 | cg15888472 |
| 1860 | cg22741735 |
| 1861 | cg14954838 |
| 1862 | cg19893751 |
| 1863 | cg07127888 |
| 1864 | cg01794103 |
| 1865 | cg06986667 |
| 1866 | cg02335251 |
| 1867 | cg09360770 |
| 1868 | cg21878746 |
| 1869 | cg27444375 |
| 1870 | cg17017189 |
| 1871 | cg26235205 |
| 1872 | cg04316624 |
| 1873 | cg10010176 |
| 1874 | cg15215612 |
| 1875 | cg00092383 |
| 1876 | cg16580616 |
| 1877 | cg10950924 |
| 1878 | cg09250087 |
| 1879 | cg07702089 |
| 1880 | cg25394203 |
| 1881 | cg23804921 |
| 1882 | cg16566400 |
| 1883 | cg06456717 |
| 1884 | cg14239111 |
| 1885 | cg07482257 |
| 1886 | cg27234340 |
| 1887 | cg02147637 |
| 1888 | cg14143326 |
| 1889 | cg01109574 |
| 1890 | cg16616514 |
| 1891 | cg17239057 |
| 1892 | cg01781663 |
| 1893 | cg09365002 |
| 1894 | cg20037601 |
| 1895 | cg26348487 |
| 1896 | cg09247297 |
| 1897 | cg03171770 |
| 1898 | cg24659873 |
| 1899 | cg02305765 |
| 1900 | cg25364619 |
| 1901 | cg17939889 |
| 1902 | cg23907504 |
| 1903 | cg06947913 |
| 1904 | cg16306978 |
| 1905 | cg09568691 |
| 1906 | cg17008486 |
| 1907 | cg09467436 |
| 1908 | cg21110337 |
| 1909 | cg03108070 |
| 1910 | cg25967031 |
| 1911 | cg07916654 |
| 1912 | cg10396171 |
| 1913 | cg18584424 |
| 1914 | cg19706516 |
| 1915 | cg02469161 |
| 1916 | cg26778345 |
| 1917 | cg23008279 |
| 1918 | cg08197287 |
| 1919 | cg07389710 |
| 1920 | cg10531263 |
| 1921 | cg05396350 |
| 1922 | cg00593536 |
| 1923 | cg24628744 |
| 1924 | cg24500294 |
| 1925 | cg27462488 |
| 1926 | cg05988523 |
| 1927 | cg10122865 |
| 1928 | cg09423312 |
| 1929 | cg13092806 |
| 1930 | cg15007959 |
| 1931 | cg14509133 |
| 1932 | cg09630706 |
| 1933 | cg17203352 |
| 1934 | cg19495714 |
| 1935 | cg09156426 |
| 1936 | cg00446472 |
| 1937 | cg03804083 |
| 1938 | cg03748603 |
| 1939 | cg09473359 |
| 1940 | cg00866399 |
| 1941 | cg18964375 |
| 1942 | cg12054981 |
| 1943 | cg03052794 |
| 1944 | cg00253379 |
| 1945 | cg00696973 |
| 1946 | cg20359930 |
| 1947 | cg13645221 |
| 1948 | cg05514256 |
| 1949 | cg11861562 |
| 1950 | cg00992189 |
| 1951 | cg00107039 |
| 1952 | cg13693429 |
| 1953 | cg00548552 |
| 1954 | cg12963656 |
| 1955 | cg22550277 |
| 1956 | cg02770835 |
| 1957 | cg18443359 |
| 1958 | cg22983282 |
| 1959 | cg04872593 |
| 1960 | cg25365014 |
| 1961 | cg25539976 |
| 1962 | cg14211930 |
| 1963 | cg04384626 |
| 1964 | cg20019410 |
| 1965 | cg04861869 |
| 1966 | cg23724878 |
| 1967 | cg08220930 |
| 1968 | cg25781627 |
| 1969 | cg03982833 |
| 1970 | cg24481303 |
| 1971 | cg27377353 |
| 1972 | cg24790297 |
| 1973 | cg08325191 |
| 1974 | cg17453780 |
| 1975 | cg18899777 |
| 1976 | cg16146718 |
| 1977 | cg21938435 |
| 1978 | cg25290445 |
| 1979 | cg02806715 |
| 1980 | cg01657926 |
| 1981 | cg26765263 |
| 1982 | cg15464821 |
| 1983 | cg12480053 |
| 1984 | cg27642784 |
| 1985 | cg25316901 |
| 1986 | cg03916909 |
| 1987 | cg22209723 |
| 1988 | cg23099839 |
| 1989 | cg03870002 |
| 1990 | cg09488203 |
| 1991 | cg24516106 |
| 1992 | cg11103390 |
| 1993 | cg13784312 |
| 1994 | cg19771746 |
| 1995 | cg26153054 |
| 1996 | cg18755094 |
| 1997 | cg11829253 |
| 1998 | cg03225476 |
| 1999 | cg01196788 |
| 2000 | cg21163429 |
| 2001 | cg16562730 |
| 2002 | cg02792700 |
| 2003 | cg24138691 |
| 2004 | cg12135344 |
| 2005 | cg26379448 |
| 2006 | cg04170535 |
| 2007 | cg12518166 |
| 2008 | cg06284231 |
| 2009 | cg25620901 |
| 2010 | cg22734058 |
| 2011 | cg16055026 |
| 2012 | cg11222217 |
| 2013 | cg11775521 |
| 2014 | cg26852712 |
| 2015 | cg16419441 |
| 2016 | cg03097275 |
| 2017 | cg05821000 |
| 2018 | cg06868100 |
| 2019 | cg14088142 |
| 2020 | cg25256866 |
| 2021 | cg13581422 |
| 2022 | cg09387486 |
| 2023 | cg05848650 |
| 2024 | cg19129687 |
| 2025 | cg00918181 |
| 2026 | cg18270446 |
| 2027 | cg00682734 |
| 2028 | cg01990695 |
| 2029 | cg08553601 |
| 2030 | cg21554552 |
| 2031 | cg15729404 |
| 2032 | cg16705744 |
| 2033 | cg16702083 |
| 2034 | cg06233996 |
| 2035 | cg23111342 |
| 2036 | cg24476033 |
| 2037 | cg05322916 |
| 2038 | cg09879382 |
| 2039 | cg09392136 |
| 2040 | cg09084470 |
| 2041 | cg16606320 |
| 2042 | cg11707067 |
| 2043 | cg23197862 |
| 2044 | cg01693350 |
| 2045 | cg10331073 |
| 2046 | cg14595058 |
| 2047 | cg25150021 |
| 2048 | cg19940077 |
| 2049 | cg02193032 |
| 2050 | cg22541254 |
| 2051 | cg17345870 |
| 2052 | cg19800916 |
| 2053 | cg16240480 |
| 2054 | cg18074954 |
| 2055 | cg14239655 |
| 2056 | cg11342941 |
| 2057 | cg18179663 |
| 2058 | cg06080793 |
| 2059 | cg25696857 |
| 2060 | cg11412129 |
| 2061 | cg07863022 |
| 2062 | cg05180443 |
| 2063 | cg20693607 |
| 2064 | cg06645921 |
| 2065 | cg14652227 |
| 2066 | cg19082359 |
| 2067 | cg04250512 |
| 2068 | cg14621217 |
| 2069 | cg00498146 |
| 2070 | cg18759102 |
| 2071 | cg19927678 |
| 2072 | cg11548971 |
| 2073 | cg10523198 |
| 2074 | cg00146756 |
| 2075 | cg04453367 |
| 2076 | cg13883671 |
| 2077 | cg15646741 |
| 2078 | cg02846841 |
| 2079 | cg14236662 |
| 2080 | cg04615481 |
| 2081 | cg08622757 |
| 2082 | cg20401567 |
| 2083 | cg24148817 |
| 2084 | cg04874795 |
| 2085 | cg15235999 |
| 2086 | cg25960038 |
| 2087 | cg26963277 |
| 2088 | cg25541528 |
| 2089 | cg26870460 |
| 2090 | cg13954457 |
| 2091 | cg04416414 |
| 2092 | cg03801871 |
| 2093 | cg18630178 |
| 2094 | cg14487368 |
| 2095 | cg22287064 |
| 2096 | cg24348495 |
| 2097 | cg22443212 |
| 2098 | cg27304020 |
| 2099 | cg11131384 |
| 2100 | cg22343728 |
| 2101 | cg13709982 |
| 2102 | cg18337363 |
| 2103 | cg09967633 |
| 2104 | cg06523901 |
| 2105 | cg12557810 |
| 2106 | cg26091183 |
| 2107 | cg24080793 |
| 2108 | cg14736083 |
| 2109 | cg09988837 |
| 2110 | cg17341136 |
| 2111 | cg23555120 |
| 2112 | cg20318045 |
| 2113 | cg04453180 |
| 2114 | cg24408646 |
| 2115 | cg11845292 |
| 2116 | cg03729128 |
| 2117 | cg21491092 |
| 2118 | cg00819895 |
| 2119 | cg17612920 |
| 2120 | cg14081465 |
| 2121 | cg05715492 |
| 2122 | cg00934864 |
| 2123 | cg06158111 |
| 2124 | cg11941633 |
| 2125 | cg14200170 |
| 2126 | cg04076766 |
| 2127 | cg16563255 |
| 2128 | cg20787382 |
| 2129 | cg12499211 |
| 2130 | cg14709479 |
| 2131 | cg02599498 |
| 2132 | cg21210531 |
| 2133 | cg10754395 |
| 2134 | cg27498387 |
| 2135 | cg04159903 |
| 2136 | cg18235000 |
| 2137 | cg20935363 |
| 2138 | cg05839377 |
| 2139 | cg25502144 |
| 2140 | cg04593843 |
| 2141 | cg05797854 |
| 2142 | cg11764900 |
| 2143 | cg09643312 |
| 2144 | cg04015097 |
| 2145 | cg07967362 |
| 2146 | cg10812186 |
| 2147 | cg10660256 |
| 2148 | cg02614024 |
| 2149 | cg10463299 |
| 2150 | cg26214742 |
| 2151 | cg03809548 |
| 2152 | cg03847932 |
| 2153 | cg23480730 |
| 2154 | cg22821289 |
| 2155 | cg16597406 |
| 2156 | cg24465685 |
| 2157 | cg04197086 |
| 2158 | cg26570165 |
| 2159 | cg22140920 |
| 2160 | cg07485198 |
| 2161 | cg05642546 |
| 2162 | cg22029587 |
| 2163 | cg18737081 |
| 2164 | cg07625849 |
| 2165 | cg21448625 |
| 2166 | cg16509851 |
| 2167 | cg01302656 |
| 2168 | cg11577310 |
| 2169 | cg20827128 |
| 2170 | cg20152309 |
| 2171 | cg00920348 |
| 2172 | cg14717557 |
| 2173 | cg02748089 |
| 2174 | cg09247319 |
| 2175 | cg08260413 |
| 2176 | cg25614396 |
| 2177 | cg11424828 |
| 2178 | cg00697672 |
| 2179 | cg16997101 |
| 2180 | cg00107970 |
| 2181 | cg25859835 |
| 2182 | cg11376198 |
| 2183 | cg05585149 |
| 2184 | cg06721601 |
| 2185 | cg14424070 |
| 2186 | cg13396967 |
| 2187 | cg00876757 |
| 2188 | cg20801456 |
| 2189 | cg22965432 |
| 2190 | cg22274813 |
| 2191 | cg04550052 |
| 2192 | cg16404157 |
| 2193 | cg27436118 |
| 2194 | cg13238455 |
| 2195 | cg13472192 |
| 2196 | cg02803819 |
| 2197 | cg03728296 |
| 2198 | cg25444386 |
| 2199 | cg06183338 |
| 2200 | cg08363339 |
| 2201 | cg20372886 |
| 2202 | cg26922917 |
| 2203 | cg22779708 |
| 2204 | cg04255230 |
| 2205 | cg13410764 |
| 2206 | cg12001774 |
| 2207 | cg11013726 |
| 2208 | cg05429873 |
| 2209 | cg04209913 |
| 2210 | cg00854817 |
| 2211 | cg00893471 |
| 2212 | cg14696458 |
| 2213 | cg26438679 |
| 2214 | cg16227104 |
| 2215 | cg00504705 |
| 2216 | cg00339682 |
| 2217 | cg11926754 |
| 2218 | cg07529122 |
| 2219 | cg18387478 |
| 2220 | cg03330490 |
| 2221 | cg00636079 |
| 2222 | cg04117530 |
| 2223 | cg19839825 |
| 2224 | cg10086659 |
| 2225 | cg18074297 |
| 2226 | cg14282386 |
| 2227 | cg23930334 |
| 2228 | cg14533595 |
| 2229 | cg26282792 |
| 2230 | cg02798576 |
| 2231 | cg08755784 |
| 2232 | cg11199639 |
| 2233 | cg01345354 |
| 2234 | cg23951961 |
| 2235 | cg16100355 |
| 2236 | cg16105635 |
| 2237 | cg02102533 |
| 2238 | cg10108468 |
| 2239 | cg21642108 |
| 2240 | cg18275732 |
| 2241 | cg10699857 |
| 2242 | cg06573459 |
| 2243 | cg20892260 |
| 2244 | cg03408619 |
| 2245 | cg00626702 |
| 2246 | cg05213296 |
| 2247 | cg13358636 |
| 2248 | cg01135380 |
| 2249 | cg22272803 |
| 2250 | cg21136104 |
| 2251 | cg17275781 |
| 2252 | cg17897547 |
| 2253 | cg26651148 |
| 2254 | cg09065714 |
| 2255 | cg00651359 |
| 2256 | cg07696842 |
| 2257 | cg11940040 |
| 2258 | cg21144493 |
| 2259 | cg04150495 |
| 2260 | cg02044895 |
| 2261 | cg16662846 |
| 2262 | cg25595762 |
| 2263 | cg12055072 |
| 2264 | cg21358336 |
| 2265 | cg18479299 |
| 2266 | cg03677069 |
| 2267 | cg06520296 |
| 2268 | cg20457062 |
| 2269 | cg08889009 |
| 2270 | cg18019070 |
| 2271 | cg27454064 |
| 2272 | cg00070383 |
| 2273 | cg06473925 |
| 2274 | cg14536682 |
| 2275 | cg19509311 |
| 2276 | cg03653399 |
| 2277 | cg19967668 |
| 2278 | cg10044179 |
| 2279 | cg25568066 |
| 2280 | cg14418633 |
| 2281 | cg07595776 |
| 2282 | cg08691567 |
| 2283 | cg07878407 |
| 2284 | cg04191300 |
| 2285 | cg13935009 |
| 2286 | cg09077096 |
| 2287 | cg25509871 |
| 2288 | cg26161708 |
| 2289 | cg02399249 |
| 2290 | cg17009165 |
| 2291 | cg10211058 |
| 2292 | cg16343401 |
| 2293 | cg21945930 |
| 2294 | cg15804426 |
| 2295 | cg22048246 |
| 2296 | cg18933685 |
| 2297 | cg11854392 |
| 2298 | cg10114725 |
| 2299 | cg13007502 |
| 2300 | cg20727434 |
| 2301 | cg24365896 |
| 2302 | cg11388673 |
| 2303 | cg11605903 |
| 2304 | cg09614721 |
| 2305 | cg20996682 |
| 2306 | cg14947429 |
| 2307 | cg05485137 |
| 2308 | cg18371700 |
| 2309 | cg18913586 |
| 2310 | cg20979737 |
| 2311 | cg09285851 |
| 2312 | cg21429982 |
| 2313 | cg06760973 |
| 2314 | cg06329392 |
| 2315 | cg10526374 |
| 2316 | cg13872005 |
| 2317 | cg11424776 |
| 2318 | cg24159559 |
| 2319 | cg03579904 |
| 2320 | cg15464880 |
| 2321 | cg15988970 |
| 2322 | cg16577083 |
| 2323 | cg27260867 |
| 2324 | cg17316126 |
| 2325 | cg17526887 |
| 2326 | cg14448830 |
| 2327 | cg19230680 |
| 2328 | cg06478504 |
| 2329 | cg13261971 |
| 2330 | cg09973514 |
| 2331 | cg21158163 |
| 2332 | cg20668221 |
| 2333 | cg09247692 |
| 2334 | cg08827307 |
| 2335 | cg10016788 |
| 2336 | cg17630392 |
| 2337 | cg03858102 |
| 2338 | cg25088079 |
| 2339 | cg05887493 |
| 2340 | cg18274480 |
| 2341 | cg05068206 |
| 2342 | cg02683197 |
| 2343 | cg15541635 |
| 2344 | cg25957677 |
| 2345 | cg02711397 |
| 2346 | cg27404186 |
| 2347 | cg22488256 |
| 2348 | cg14654468 |
| 2349 | cg24191935 |
| 2350 | cg09503045 |
| 2351 | cg12449325 |
| 2352 | cg17244980 |
| 2353 | cg17854454 |
| 2354 | cg04421553 |
| 2355 | cg09515205 |
| 2356 | cg16202734 |
| 2357 | cg20952901 |
| 2358 | cg02726898 |
| 2359 | cg21464303 |
| 2360 | cg17758300 |
| 2361 | cg06390536 |
| 2362 | cg12270180 |
| 2363 | cg19349372 |
| 2364 | cg23587025 |
| 2365 | cg05697835 |
| 2366 | cg23683010 |
| 2367 | cg25381908 |
| 2368 | cg08718098 |
| 2369 | cg04194840 |
| 2370 | cg17054060 |
| 2371 | cg10803577 |
| 2372 | cg02408697 |
| 2373 | cg03185552 |
| 2374 | cg10336764 |
| 2375 | cg18582824 |
| 2376 | cg11797634 |
| 2377 | cg14161454 |
| 2378 | cg22777724 |
| 2379 | cg12048965 |
| 2380 | cg21743907 |
| 2381 | cg01060950 |
| 2382 | cg02571636 |
| 2383 | cg05857967 |
| 2384 | cg16835209 |
| 2385 | cg20905214 |
| 2386 | cg09837037 |
| 2387 | cg12786570 |
| 2388 | cg24405700 |
| 2389 | cg11600734 |
| 2390 | cg12230203 |
| 2391 | cg19888481 |
| 2392 | cg09623974 |
| 2393 | cg09030546 |
| 2394 | cg05266989 |
| 2395 | cg03494634 |
| 2396 | cg14042988 |
| 2397 | cg20449685 |
| 2398 | cg04779752 |
| 2399 | cg03248767 |
| 2400 | cg01045337 |
| 2401 | cg26975184 |
| 2402 | cg12480547 |
| 2403 | cg01169021 |
| 2404 | cg20622669 |
| 2405 | cg20770857 |
| 2406 | cg21519654 |
| 2407 | cg13773247 |
| 2408 | cg14604444 |
| 2409 | cg20053110 |
| 2410 | cg13752828 |
| 2411 | cg27154487 |
| 2412 | cg15402529 |
| 2413 | cg14304765 |
| 2414 | cg27190410 |
| 2415 | cg20622019 |
| 2416 | cg22340508 |
| 2417 | cg11229513 |
| 2418 | cg01656087 |
| 2419 | cg26457160 |
| 2420 | cg02711163 |
| 2421 | cg03495084 |
| 2422 | cg24045378 |
| 2423 | cg09402243 |
| 2424 | cg02533289 |
| 2425 | cg18725573 |
| 2426 | cg27565650 |
| 2427 | cg26402660 |
| 2428 | cg18486102 |
| 2429 | cg14111334 |
| 2430 | cg13378649 |
| 2431 | cg16307866 |
| 2432 | cg12924430 |
| 2433 | cg18472448 |
| 2434 | cg09501687 |
| 2435 | cg00496939 |
| 2436 | cg03022680 |
| 2437 | cg09363735 |
| 2438 | cg23647711 |
| 2439 | cg06692785 |
| 2440 | cg16866294 |
| 2441 | cg02745419 |
| 2442 | cg09959420 |
| 2443 | cg21609154 |
| 2444 | cg19037007 |
| 2445 | cg05321225 |
| 2446 | cg25365260 |
| 2447 | cg10854819 |
| 2448 | cg05014727 |
| 2449 | cg17496661 |
| 2450 | cg18800060 |
| 2451 | cg14204499 |
| 2452 | cg00210271 |
| 2453 | cg15081698 |
| 2454 | cg14366619 |
| 2455 | cg01712159 |
| 2456 | cg18512948 |
| 2457 | cg15949714 |
| 2458 | cg12454169 |
| 2459 | cg00978634 |
| 2460 | cg22741014 |
| 2461 | cg09670128 |
| 2462 | cg00935328 |
| 2463 | cg18089426 |
| 2464 | cg13799970 |
| 2465 | cg05162523 |
| 2466 | cg09780180 |
| 2467 | cg17681527 |
| 2468 | cg05726764 |
| 2469 | cg03837909 |
| 2470 | cg23653885 |
| 2471 | cg08686553 |
| 2472 | cg02318139 |
| 2473 | cg16677191 |
| 2474 | cg03023837 |
| 2475 | cg06775523 |
| 2476 | cg07208853 |
| 2477 | cg07679948 |
| 2478 | cg03694713 |
| 2479 | cg08750459 |
| 2480 | cg15119326 |
| 2481 | cg16985288 |
| 2482 | cg13026730 |
| 2483 | cg02608002 |
| 2484 | cg11451379 |
| 2485 | cg16264966 |
| 2486 | cg06159896 |
| 2487 | cg18208842 |
| 2488 | cg23433370 |
| 2489 | cg05304815 |
| 2490 | cg05214293 |
| 2491 | cg04064644 |
| 2492 | cg26396443 |
| 2493 | cg09214398 |
| 2494 | cg10772290 |
| 2495 | cg16969189 |
| 2496 | cg24206614 |
| 2497 | cg16519100 |
| 2498 | cg00748494 |
| 2499 | cg21517258 |
| 2500 | cg16661628 |
| 2501 | cg03709475 |
| 2502 | cg01286685 |
| 2503 | cg03604073 |
| 2504 | cg14547414 |
| 2505 | cg07429623 |
| 2506 | cg17466653 |
| 2507 | cg04027043 |
| 2508 | cg11430259 |
| 2509 | cg13413286 |
| 2510 | cg05768532 |
| 2511 | cg08516222 |
| 2512 | cg03095344 |
| 2513 | cg08806779 |
| 2514 | cg18771570 |
| 2515 | cg18232861 |
| 2516 | cg15575375 |
| 2517 | cg20459687 |
| 2518 | cg10244976 |
| 2519 | cg00804179 |
| 2520 | cg20889818 |
| 2521 | cg08894487 |
| 2522 | cg13525071 |
| 2523 | cg19796981 |
| 2524 | cg03882324 |
| 2525 | cg26940676 |
| 2526 | cg02903822 |
| 2527 | cg20856378 |
| 2528 | cg25669593 |
| 2529 | cg04879750 |
| 2530 | cg00407245 |
| 2531 | cg21691166 |
| 2532 | cg03277049 |
| 2533 | cg09648727 |
| 2534 | cg09082427 |
| 2535 | cg11288686 |
| 2536 | cg01567173 |
| 2537 | cg07644368 |
| 2538 | cg00766382 |
| 2539 | cg14181111 |
| 2540 | cg17494819 |
| 2541 | cg03109053 |
| 2542 | cg08699646 |
| 2543 | cg16200879 |
| 2544 | cg17736802 |
| 2545 | cg24522254 |
| 2546 | cg14909856 |
| 2547 | cg24896021 |
| 2548 | cg17497608 |
| 2549 | cg18526607 |
| 2550 | cg09867290 |
| 2551 | cg16696952 |
| 2552 | cg18096251 |
| 2553 | cg08834902 |
| 2554 | cg14602393 |
| 2555 | cg02193187 |
| 2556 | cg19988426 |
| 2557 | cg24045348 |
| 2558 | cg17193237 |
| 2559 | cg07525299 |
| 2560 | cg18780257 |
| 2561 | cg13633361 |
| 2562 | cg05890119 |
| 2563 | cg27391627 |
| 2564 | cg04154027 |
| 2565 | cg27530576 |
| 2566 | cg13824270 |
| 2567 | cg23244761 |
| 2568 | cg12753926 |
| 2569 | cg16580762 |
| 2570 | cg11921736 |
| 2571 | cg25289604 |
| 2572 | cg20987850 |
| 2573 | cg02356288 |
| 2574 | cg26703511 |
| 2575 | cg03257743 |
| 2576 | cg13809441 |
| 2577 | cg01611115 |
| 2578 | cg03196975 |
| 2579 | cg23464743 |
| 2580 | cg11092487 |
| 2581 | cg26441055 |
| 2582 | cg10669368 |
| 2583 | cg07109115 |
| 2584 | cg15244830 |
| 2585 | cg23564780 |
| 2586 | cg18425434 |
| 2587 | cg20077028 |
| 2588 | cg19075787 |
| 2589 | cg21160290 |
| 2590 | cg16576033 |
| 2591 | cg12055993 |
| 2592 | cg26992010 |
| 2593 | cg01152019 |
| 2594 | cg06311422 |
| 2595 | cg24992816 |
| 2596 | cg26739149 |
| 2597 | cg09978860 |
| 2598 | cg14027333 |
| 2599 | cg26694136 |
| 2600 | cg03834478 |
| 2601 | cg22660074 |
| 2602 | cg09662852 |
| 2603 | cg23895495 |
| 2604 | cg05475934 |
| 2605 | cg05528899 |
| 2606 | cg09163005 |
| 2607 | cg11673734 |
| 2608 | cg02580923 |
| 2609 | cg01726202 |
| 2610 | cg09704116 |
| 2611 | cg04478861 |
| 2612 | cg17991695 |
| 2613 | cg16311536 |
| 2614 | cg27262717 |
| 2615 | cg12198813 |
| 2616 | cg25628701 |
| 2617 | cg10764013 |
| 2618 | cg17463745 |
| 2619 | cg04029159 |
| 2620 | cg00403498 |
| 2621 | cg17059658 |
| 2622 | cg24735277 |
| 2623 | cg03015498 |
| 2624 | cg09533868 |
| 2625 | cg20724257 |
| 2626 | cg07086918 |
| 2627 | cg21923482 |
| 2628 | cg05044743 |
| 2629 | cg16940506 |
| 2630 | cg18960464 |
| 2631 | cg16781264 |
| 2632 | cg07599133 |
| 2633 | cg25277809 |
| 2634 | cg05854261 |
| 2635 | cg07752120 |
| 2636 | cg03349184 |
| 2637 | cg14533068 |
| 2638 | cg21778743 |
| 2639 | cg27591957 |
| 2640 | cg16727774 |
| 2641 | cg23639196 |
| 2642 | cg08948808 |
| 2643 | cg02473540 |
| 2644 | cg18720506 |
| 2645 | cg23040290 |
| 2646 | cg06193578 |
| 2647 | cg05088892 |
| 2648 | cg10296410 |
| 2649 | cg01121022 |
| 2650 | cg26581660 |
| 2651 | cg23118561 |
| 2652 | cg25121292 |
| 2653 | cg10153714 |
| 2654 | cg08871701 |
| 2655 | cg11019430 |
| 2656 | cg23476877 |
| 2657 | cg00058576 |
| 2658 | cg03228099 |
| 2659 | cg16577356 |
| 2660 | cg17747518 |
| 2661 | cg01440309 |
| 2662 | cg18155888 |
| 2663 | cg15009484 |
| 2664 | cg25828445 |
| 2665 | cg24508005 |
| 2666 | cg17206212 |
| 2667 | cg26363759 |
| 2668 | cg18381051 |
| 2669 | cg16487577 |
| 2670 | cg08076957 |
| 2671 | cg14424313 |
| 2672 | cg10496184 |
| 2673 | cg10785622 |
| 2674 | cg11909912 |
| 2675 | cg24660009 |
| 2676 | cg02340755 |
| 2677 | cg02025583 |
| 2678 | cg09568647 |
| 2679 | cg07130266 |
| 2680 | cg26540315 |
| 2681 | cg11930554 |
| 2682 | cg17717576 |
| 2683 | cg02457680 |
| 2684 | cg08233811 |
| 2685 | cg09034874 |
| 2686 | cg17188046 |
| 2687 | cg07872945 |
| 2688 | cg16175263 |
| 2689 | cg18689332 |
| 2690 | cg24480012 |
| 2691 | cg09545579 |
| 2692 | cg06551997 |
| 2693 | cg12021814 |
| 2694 | cg08883853 |
| 2695 | cg07734975 |
| 2696 | cg23104954 |
| 2697 | cg09522018 |
| 2698 | cg05163330 |
| 2699 | cg20429172 |
| 2700 | cg24569831 |
| 2701 | cg09991306 |
| 2702 | cg05479962 |
| 2703 | cg08000754 |
| 2704 | cg16321280 |
| 2705 | cg27255534 |
| 2706 | cg06242102 |
| 2707 | cg27362525 |
| 2708 | cg19206146 |
| 2709 | cg25565412 |
| 2710 | cg13413719 |
| 2711 | cg14784953 |
| 2712 | cg08802482 |
| 2713 | cg18173106 |
| 2714 | cg17001525 |
| 2715 | cg13959371 |
| 2716 | cg07369606 |
| 2717 | cg22006640 |
| 2718 | cg06094235 |
| 2719 | cg10024583 |
| 2720 | cg13951697 |
| 2721 | cg20509903 |
| 2722 | cg26070300 |
| 2723 | cg25174438 |
| 2724 | cg21550804 |
| 2725 | cg25825177 |
| 2726 | cg04483289 |
| 2727 | cg13840445 |
| 2728 | cg02693498 |
| 2729 | cg19090784 |
| 2730 | cg20771596 |
| 2731 | cg01399826 |
| 2732 | cg01268383 |
| 2733 | cg27296119 |
| 2734 | cg15087907 |
| 2735 | cg19489503 |
| 2736 | cg21723559 |
| 2737 | cg06502892 |
| 2738 | cg27014380 |
| 2739 | cg21679391 |
| 2740 | cg02328793 |
| 2741 | cg04138112 |
| 2742 | cg27599376 |
| 2743 | cg27662921 |
| 2744 | cg08505032 |
| 2745 | cg00955254 |
| 2746 | cg24112000 |
| 2747 | cg14504259 |
| 2748 | cg23218559 |
| 2749 | cg17928459 |
| 2750 | cg09851545 |
| 2751 | cg08555653 |
| 2752 | cg14453174 |
| 2753 | cg21760990 |
| 2754 | cg03392386 |
| 2755 | cg15229836 |
| 2756 | cg22158992 |
| 2757 | cg22489321 |
| 2758 | cg04701034 |
| 2759 | cg08721338 |
| 2760 | cg25247859 |
| 2761 | cg07700843 |
| 2762 | cg25883146 |
| 2763 | cg13455717 |
| 2764 | cg08793894 |
| 2765 | cg22900415 |
| 2766 | cg22571664 |
| 2767 | cg13374432 |
| 2768 | cg06294698 |
| 2769 | cg09652652 |
| 2770 | cg07786675 |
| 2771 | cg15662844 |
| 2772 | cg09976984 |
| 2773 | cg13757081 |
| 2774 | cg23249922 |
| 2775 | cg17939286 |
| 2776 | cg14491582 |
| 2777 | cg07891457 |
| 2778 | cg21282630 |
| 2779 | cg05863502 |
| 2780 | cg10329345 |
| 2781 | cg12158516 |
| 2782 | cg20852226 |
| 2783 | cg17519949 |
| 2784 | cg07773582 |
| 2785 | cg02320862 |
| 2786 | cg08141615 |
| 2787 | cg22976218 |
| 2788 | cg07675477 |
| 2789 | cg10622643 |
| 2790 | cg07213780 |
| 2791 | cg15829088 |
| 2792 | cg04376887 |
| 2793 | cg25896644 |
| 2794 | cg04757389 |
| 2795 | cg11036833 |
| 2796 | cg14496753 |
| 2797 | cg17861863 |
| 2798 | cg20684865 |
| 2799 | cg01073837 |
| 2800 | cg18955749 |
| 2801 | cg10159630 |
| 2802 | cg25282976 |
| 2803 | cg18358098 |
| 2804 | cg09368188 |
| 2805 | cg14654171 |
| 2806 | cg08124030 |
| 2807 | cg12827351 |
| 2808 | cg12065138 |
| 2809 | cg20264482 |
| 2810 | cg22320696 |
| 2811 | cg11704068 |
| 2812 | cg14710465 |
| 2813 | cg08474859 |
| 2814 | cg07052787 |
| 2815 | cg00270625 |
| 2816 | cg14940165 |
| 2817 | cg04497116 |
| 2818 | cg20801007 |
| 2819 | cg00088183 |
| 2820 | cg08438435 |
| 2821 | cg11931211 |
| 2822 | cg26391634 |
| 2823 | cg09678193 |
| 2824 | cg17473898 |
| 2825 | cg18020955 |
| 2826 | cg27665978 |
| 2827 | cg11508406 |
| 2828 | cg24604692 |
| 2829 | cg17733447 |
| 2830 | cg13351249 |
| 2831 | cg23754052 |
| 2832 | cg24161501 |
| 2833 | cg27434509 |
| 2834 | cg00204465 |
| 2835 | cg23440816 |
| 2836 | cg18832142 |
| 2837 | cg08214423 |
| 2838 | cg09213533 |
| 2839 | cg22654157 |
| 2840 | cg26186239 |
| 2841 | cg23759448 |
| 2842 | cg18089519 |
| 2843 | cg27259271 |
| 2844 | cg07787301 |
| 2845 | cg13819877 |
| 2846 | cg09096947 |
| 2847 | cg11774624 |
| 2848 | cg26398921 |
| 2849 | cg08858649 |
| 2850 | cg25970575 |
| 2851 | cg22318514 |
| 2852 | cg20137991 |
| 2853 | cg13132858 |
| 2854 | cg12955789 |
| 2855 | cg18045515 |
| 2856 | cg23891273 |
| 2857 | cg26753302 |
| 2858 | cg14696311 |
| 2859 | cg23778422 |
| 2860 | cg11248896 |
| 2861 | cg04526750 |
| 2862 | cg06997767 |
| 2863 | cg09682051 |
| 2864 | cg02897230 |
| 2865 | cg09393254 |
| 2866 | cg07141605 |
| 2867 | cg24069444 |
| 2868 | cg02873954 |
| 2869 | cg04131829 |
| 2870 | cg11586570 |
| 2871 | cg07504200 |
| 2872 | cg06376520 |
| 2873 | cg19690984 |
| 2874 | cg12855906 |
| 2875 | cg06274007 |
| 2876 | cg24845595 |
| 2877 | cg19816811 |
| 2878 | cg04743876 |
| 2879 | cg21929564 |
| 2880 | cg08458738 |
| 2881 | cg19181528 |
| 2882 | cg22558685 |
| 2883 | cg26635219 |
| 2884 | cg04546205 |
| 2885 | cg16655240 |
| 2886 | cg08243728 |
| 2887 | cg26562263 |
| 2888 | cg20966754 |
| 2889 | cg21301614 |
| 2890 | cg04657146 |
| 2891 | cg16561657 |
| 2892 | cg06362176 |
| 2893 | cg17463145 |
| 2894 | cg21685427 |
| 2895 | cg24971189 |
| 2896 | cg20651616 |
| 2897 | cg15016701 |
| 2898 | cg25858132 |
| 2899 | cg13718418 |
| 2900 | cg12182643 |
| 2901 | cg26047066 |
| 2902 | cg06141694 |
| 2903 | cg04364629 |
| 2904 | cg02528959 |
| 2905 | cg08433226 |
| 2906 | cg19119609 |
| 2907 | cg05407321 |
| 2908 | cg10999423 |
| 2909 | cg18822950 |
| 2910 | cg14076977 |
| 2911 | cg11549953 |
| 2912 | cg17096289 |
| 2913 | cg09803494 |
| 2914 | cg16903347 |
| 2915 | cg06034749 |
| 2916 | cg01280597 |
| 2917 | cg16238993 |
| 2918 | cg15809598 |
| 2919 | cg11414821 |
| 2920 | cg22486834 |
| 2921 | cg10679156 |
| 2922 | cg14006181 |
| 2923 | cg12850379 |
| 2924 | cg17807172 |
| 2925 | cg20885179 |
| 2926 | cg10465805 |
| 2927 | cg10863113 |
| 2928 | cg10978034 |
| 2929 | cg18145937 |
| 2930 | cg12472603 |
| 2931 | cg05835982 |
| 2932 | cg11277143 |
| 2933 | cg21370007 |
| 2934 | cg10508760 |
| 2935 | cg01137366 |
| 2936 | cg05750856 |
| 2937 | cg09688127 |
| 2938 | cg14509403 |
| 2939 | cg17534464 |
| 2940 | cg07084380 |
| 2941 | cg24679453 |
| 2942 | cg03396103 |
| 2943 | cg16058797 |
| 2944 | cg23940612 |
| 2945 | cg06155620 |
| 2946 | cg20507963 |
| 2947 | cg20948486 |
| 2948 | cg12281219 |
| 2949 | cg14571622 |
| 2950 | cg00415993 |
| 2951 | cg16624482 |
| 2952 | cg15243856 |
| 2953 | cg17541002 |
| 2954 | cg06300823 |
| 2955 | cg24091995 |
| 2956 | cg23035323 |
| 2957 | cg09734791 |
| 2958 | cg17953385 |
| 2959 | cg25031836 |
| 2960 | cg12761825 |
| 2961 | cg22093306 |
| 2962 | cg04409354 |
| 2963 | cg19971555 |
| 2964 | cg09492887 |
| 2965 | cg05341539 |
| 2966 | cg18928066 |
| 2967 | cg10586836 |
| 2968 | cg05669550 |
| 2969 | cg18229178 |
| 2970 | cg05983315 |
| 2971 | cg19664945 |
| 2972 | cg05769892 |
| 2973 | cg15974634 |
| 2974 | cg11896799 |
| 2975 | cg06516124 |
| 2976 | cg26082368 |
| 2977 | cg09374838 |
| 2978 | cg20982735 |
| 2979 | cg23565445 |
| 2980 | cg05429448 |
| 2981 | cg06241101 |
| 2982 | cg04257913 |
| 2983 | cg08330247 |
| 2984 | cg27162750 |
| 2985 | cg07506913 |
| 2986 | cg17415382 |
| 2987 | cg27263966 |
| 2988 | cg17330251 |
| 2989 | cg11581653 |
| 2990 | cg04554272 |
| 2991 | cg27363726 |
| 2992 | cg10087556 |
| 2993 | cg13468318 |
| 2994 | cg12567063 |
| 2995 | cg12150784 |
| 2996 | cg12557114 |
| 2997 | cg04093349 |
| 2998 | cg14038058 |
| 2999 | cg01089319 |
| 3000 | cg12284769 |
| 3001 | cg04540442 |
| 3002 | cg14243190 |
| 3003 | cg05997779 |
| 3004 | cg00063828 |
| 3005 | cg02505812 |
| 3006 | cg09937039 |
| 3007 | cg25992321 |
| 3008 | cg11294513 |
| 3009 | cg25539045 |
| 3010 | cg21708703 |
| 3011 | cg23568976 |
| 3012 | cg12018098 |
| 3013 | cg20120197 |
| 3014 | cg19224645 |
| 3015 | cg16270399 |
| 3016 | cg01558110 |
| 3017 | cg10518264 |
| 3018 | cg17716500 |
| 3019 | cg17670237 |
| 3020 | cg27526549 |
| 3021 | cg18457425 |
| 3022 | cg15613991 |
| 3023 | cg04806177 |
| 3024 | cg00165647 |
| 3025 | cg21974464 |
| 3026 | cg20249327 |
| 3027 | cg10299585 |
| 3028 | cg06997226 |
| 3029 | cg23517208 |
| 3030 | cg23918296 |
| 3031 | cg18736063 |
| 3032 | cg05387815 |
| 3033 | cg07786995 |
| 3034 | cg15787282 |
| 3035 | cg19437284 |
| 3036 | cg10485937 |
| 3037 | cg16591159 |
| 3038 | cg14789818 |
| 3039 | cg05028087 |
| 3040 | cg20630344 |
| 3041 | cg25344503 |
| 3042 | cg02286554 |
| 3043 | cg13936938 |
| 3044 | cg01639524 |
| 3045 | cg16132337 |
| 3046 | cg17408527 |
| 3047 | cg24787461 |
| 3048 | cg12614105 |
| 3049 | cg12470791 |
| 3050 | cg17301379 |
| 3051 | cg23553912 |
| 3052 | cg26132320 |
| 3053 | cg19213744 |
| 3054 | cg10636246 |
| 3055 | cg21264391 |
| 3056 | cg01308827 |
| 3057 | cg07601320 |
| 3058 | cg27303185 |
| 3059 | cg08381620 |
| 3060 | cg03077645 |
| 3061 | cg01607625 |
| 3062 | cg08701420 |
| 3063 | cg18132787 |
| 3064 | cg15628917 |
| 3065 | cg04319873 |
| 3066 | cg11574184 |
| 3067 | cg06105699 |
| 3068 | cg00424064 |
| 3069 | cg17368562 |
| 3070 | cg14708990 |
| 3071 | cg22322605 |
| 3072 | cg25304107 |
| 3073 | cg17292758 |
| 3074 | cg23010048 |
| 3075 | cg12216208 |
| 3076 | cg26714230 |
| 3077 | cg09236434 |
| 3078 | cg01974091 |
| 3079 | cg14893857 |
| 3080 | cg26246467 |
| 3081 | cg08423857 |
| 3082 | cg23272088 |
| 3083 | cg10883827 |
| 3084 | cg09016968 |
| 3085 | cg13939745 |
| 3086 | cg08428188 |
| 3087 | cg18893168 |
| 3088 | cg13638420 |
| 3089 | cg14803632 |
| 3090 | cg18273840 |
| 3091 | cg20373544 |
| 3092 | cg03192273 |
| 3093 | cg02799411 |
| 3094 | cg06906523 |
| 3095 | cg18747561 |
| 3096 | cg06629130 |
| 3097 | cg01177361 |
| 3098 | cg15060367 |
| 3099 | cg27182548 |
| 3100 | cg00647820 |
| 3101 | cg04961235 |
| 3102 | cg09546780 |
| 3103 | cg20695627 |
| 3104 | cg26648818 |
| 3105 | cg13702536 |
| 3106 | cg24541426 |
| 3107 | cg14487292 |
| 3108 | cg01405767 |
| 3109 | cg06853894 |
| 3110 | cg24475058 |
| 3111 | cg20979800 |
| 3112 | cg26963545 |
| 3113 | cg17118435 |
| 3114 | cg13973086 |
| 3115 | cg18912520 |
| 3116 | cg03368155 |
| 3117 | cg20657864 |
| 3118 | cg09527615 |
| 3119 | cg10427501 |
| 3120 | cg13997626 |
| 3121 | cg25744613 |
| 3122 | cg02805665 |
| 3123 | cg13154880 |
| 3124 | cg20891558 |
| 3125 | cg27523952 |
| 3126 | cg01037756 |
| 3127 | cg10072115 |
| 3128 | cg25449440 |
| 3129 | cg02099337 |
| 3130 | cg18174404 |
| 3131 | cg04415277 |
| 3132 | cg17632299 |
| 3133 | cg05394663 |
| 3134 | cg21701994 |
| 3135 | cg19566482 |
| 3136 | cg25685262 |
| 3137 | cg05140806 |
| 3138 | cg05032059 |
| 3139 | cg23089272 |
| 3140 | cg02420128 |
| 3141 | cg01017244 |
| 3142 | cg19344626 |
| 3143 | cg18171212 |
| 3144 | cg12025048 |
| 3145 | cg09480190 |
| 3146 | cg20286810 |
| 3147 | cg15038286 |
| 3148 | cg12475142 |
| 3149 | cg20034617 |
| 3150 | cg08279186 |
| 3151 | cg24278165 |
| 3152 | cg22157099 |
| 3153 | cg19901801 |
| 3154 | cg10089801 |
| 3155 | cg07119434 |
| 3156 | cg12301347 |
| 3157 | cg08224569 |
| 3158 | cg24570302 |
| 3159 | cg19519747 |
| 3160 | cg23494847 |
| 3161 | cg00793719 |
| 3162 | cg22920700 |
| 3163 | cg10934129 |
| 3164 | cg26913058 |
| 3165 | cg02216731 |
| 3166 | cg18645081 |
| 3167 | cg16945633 |
| 3168 | cg22185879 |
| 3169 | cg16602316 |
| 3170 | cg06703222 |
| 3171 | cg01847718 |
| 3172 | cg14133945 |
| 3173 | cg20309629 |
| 3174 | cg08398691 |
| 3175 | cg16922937 |
| 3176 | cg11759172 |
| 3177 | cg14228592 |
| 3178 | cg22457769 |
| 3179 | cg15145296 |
| 3180 | cg10124710 |
| 3181 | cg14567917 |
| 3182 | cg10854092 |
| 3183 | cg01257685 |
| 3184 | cg21665744 |
| 3185 | cg25362652 |
| 3186 | cg21127079 |
| 3187 | cg12744859 |
| 3188 | cg23936766 |
| 3189 | cg21202716 |
| 3190 | cg12058390 |
| 3191 | cg11309462 |
| 3192 | cg02559101 |
| 3193 | cg02613338 |
| 3194 | cg14482313 |
| 3195 | cg00169725 |
| 3196 | cg12241243 |
| 3197 | cg07161242 |
| 3198 | cg20333059 |
| 3199 | cg00918522 |
| 3200 | cg03668598 |
| 3201 | cg01511828 |
| 3202 | cg05475524 |
| 3203 | cg10177477 |
| 3204 | cg16735685 |
| 3205 | cg21101743 |
| 3206 | cg14035368 |
| 3207 | cg10716350 |
| 3208 | cg22993667 |
| 3209 | cg01413054 |
| 3210 | cg25143990 |
| 3211 | cg15039102 |
| 3212 | cg03640756 |
| 3213 | cg10893095 |
| 3214 | cg27307298 |
| 3215 | cg12874971 |
| 3216 | cg01612681 |
| 3217 | cg02346342 |
| 3218 | cg14204619 |
| 3219 | cg18848959 |
| 3220 | cg05146544 |
| 3221 | cg19767622 |
| 3222 | cg14815891 |
| 3223 | cg08404009 |
| 3224 | cg14097304 |
| 3225 | cg23497569 |
| 3226 | cg08359236 |
| 3227 | cg00634652 |
| 3228 | cg02049405 |
| 3229 | cg21144063 |
| 3230 | cg08933008 |
| 3231 | cg10680328 |
| 3232 | cg14684854 |
| 3233 | cg21843594 |
| 3234 | cg00183684 |
| 3235 | cg12073779 |
| 3236 | cg19978181 |
| 3237 | cg07852840 |
| 3238 | cg09672187 |
| 3239 | cg10858103 |
| 3240 | cg22407942 |
| 3241 | cg26926768 |
| 3242 | cg15909132 |
| 3243 | cg27168291 |
| 3244 | cg15095922 |
| 3245 | cg08144358 |
| 3246 | cg26619920 |
| 3247 | cg01416891 |
| 3248 | cg16492341 |
| 3249 | cg08762424 |
| 3250 | cg16612112 |
| 3251 | cg04954726 |
| 3252 | cg15172734 |
| 3253 | cg09868780 |
| 3254 | cg01056242 |
| 3255 | cg23712018 |
| 3256 | cg18843682 |
| 3257 | cg06112894 |
| 3258 | cg12905085 |
| 3259 | cg10899768 |
| 3260 | cg07885629 |
| 3261 | cg18148375 |
| 3262 | cg20057198 |
| 3263 | cg26841048 |
| 3264 | cg22070156 |
| 3265 | cg17454746 |
| 3266 | cg19764764 |
| 3267 | cg16276982 |
| 3268 | cg13930261 |
| 3269 | cg05695106 |
| 3270 | cg06832898 |
| 3271 | cg10042327 |
| 3272 | cg10854758 |
| 3273 | cg15645685 |
| 3274 | cg00140447 |
| 3275 | cg05412788 |
| 3276 | cg07462756 |
| 3277 | cg19627033 |
| 3278 | cg09737314 |
| 3279 | cg21776494 |
| 3280 | cg20522370 |
| 3281 | cg19163350 |
| 3282 | cg03926906 |
| 3283 | cg01997006 |
| 3284 | cg19744936 |
| 3285 | cg25063737 |
| 3286 | cg10321723 |
| 3287 | cg02622133 |
| 3288 | cg25044544 |
| 3289 | cg00472277 |
| 3290 | cg18085176 |
| 3291 | cg26830864 |
| 3292 | cg10815453 |
| 3293 | cg24258529 |
| 3294 | cg13550401 |
| 3295 | cg07971908 |
| 3296 | cg22686132 |
| 3297 | cg20638675 |
| 3298 | cg06518618 |
| 3299 | cg02184673 |
| 3300 | cg11282657 |
| 3301 | cg05221664 |
| 3302 | cg15621260 |
| 3303 | cg21974506 |
| 3304 | cg01081584 |
| 3305 | cg26539571 |
| 3306 | cg25343618 |
| 3307 | cg16288713 |
| 3308 | cg19777396 |
| 3309 | cg03780701 |
| 3310 | cg20038493 |
| 3311 | cg15831967 |
| 3312 | cg27105990 |
| 3313 | cg14776998 |
| 3314 | cg07962143 |
| 3315 | cg24859352 |
| 3316 | cg21171625 |
| 3317 | cg26313888 |
| 3318 | cg08779649 |
| 3319 | cg14534848 |
| 3320 | cg04909854 |
| 3321 | cg00377344 |
| 3322 | cg01887995 |
| 3323 | cg15508761 |
| 3324 | cg00876273 |
| 3325 | cg19406103 |
| 3326 | cg06894134 |
| 3327 | cg27173819 |
| 3328 | cg02297967 |
| 3329 | cg08146483 |
| 3330 | cg20299802 |
| 3331 | cg15608301 |
| 3332 | cg01715455 |
| 3333 | cg00278517 |
| 3334 | cg27451484 |
| 3335 | cg01820273 |
| 3336 | cg15731815 |
| 3337 | cg09321817 |
| 3338 | cg10763878 |
| 3339 | cg07795402 |
| 3340 | cg16399632 |
| 3341 | cg26186509 |
| 3342 | cg01864336 |
| 3343 | cg24090990 |
| 3344 | cg10243459 |
| 3345 | cg18105227 |
| 3346 | cg00610577 |
| 3347 | cg12179826 |
| 3348 | cg05771369 |
| 3349 | cg16218494 |
| 3350 | cg13019085 |
| 3351 | cg00788177 |
| 3352 | cg17836145 |
| 3353 | cg05130482 |
| 3354 | cg16218721 |
| 3355 | cg22488253 |
| 3356 | cg25942789 |
| 3357 | cg09872582 |
| 3358 | cg04940329 |
| 3359 | cg09577511 |
| 3360 | cg00343633 |
| 3361 | cg05236677 |
| 3362 | cg20333383 |
| 3363 | cg24330456 |
| 3364 | cg03687070 |
| 3365 | cg00947599 |
| 3366 | cg15496956 |
| 3367 | cg23336797 |
| 3368 | cg14253655 |
| 3369 | cg13114921 |
| 3370 | cg12935023 |
| 3371 | cg25726549 |
| 3372 | cg23357995 |
| 3373 | cg10506844 |
| 3374 | cg08253266 |
| 3375 | cg01302593 |
| 3376 | cg22031964 |
| 3377 | cg22796604 |
| 3378 | cg20803293 |
| 3379 | cg23313138 |
| 3380 | cg15455643 |
| 3381 | cg10389730 |
| 3382 | cg23424933 |
| 3383 | cg01606801 |
| 3384 | cg15335334 |
| 3385 | cg19158553 |
| 3386 | cg03486986 |
| 3387 | cg04166812 |
| 3388 | cg07121856 |
| 3389 | cg06556702 |
| 3390 | cg13590223 |
| 3391 | cg14423853 |
| 3392 | cg26451062 |
| 3393 | cg24450239 |
| 3394 | cg04864659 |
| 3395 | cg10514675 |
| 3396 | cg07891271 |
| 3397 | cg06058576 |
| 3398 | cg26220673 |
| 3399 | cg15283092 |
| 3400 | cg05280501 |
| 3401 | cg15250073 |
| 3402 | cg14933163 |
| 3403 | cg01128736 |
| 3404 | cg13923648 |
| 3405 | cg15961780 |
| 3406 | cg24036791 |
| 3407 | cg18802754 |
| 3408 | cg06896987 |
| 3409 | cg06734406 |
| 3410 | cg22259285 |
| 3411 | cg21477448 |
| 3412 | cg05918002 |
| 3413 | cg25137787 |
| 3414 | cg15518950 |
| 3415 | cg00661970 |
| 3416 | cg10493961 |
| 3417 | cg21117965 |
| 3418 | cg24834854 |
| 3419 | cg04805131 |
| 3420 | cg22753416 |
| 3421 | cg05891292 |
| 3422 | cg13187827 |
| 3423 | cg01495918 |
| 3424 | cg23391372 |
| 3425 | cg22151581 |
| 3426 | cg17071045 |
| 3427 | cg03840504 |
| 3428 | cg17079405 |
| 3429 | cg14626259 |
| 3430 | cg18373855 |
| 3431 | cg23930313 |
| 3432 | cg03815621 |
| 3433 | cg06070263 |
| 3434 | cg18856581 |
| 3435 | cg08692757 |
| 3436 | cg17566517 |
| 3437 | cg23424003 |
| 3438 | cg24394856 |
| 3439 | cg25640519 |
| 3440 | cg11724135 |
| 3441 | cg21884064 |
| 3442 | cg23497683 |
| 3443 | cg11003133 |
| 3444 | cg10517357 |
| 3445 | cg19697239 |
| 3446 | cg18343139 |
| 3447 | cg06151165 |
| 3448 | cg02504820 |
| 3449 | cg16576285 |
| 3450 | cg23166590 |
| 3451 | cg18995143 |
| 3452 | cg12798845 |
| 3453 | cg24271806 |
| 3454 | cg08194989 |
| 3455 | cg06881666 |
| 3456 | cg23682210 |
| 3457 | cg12967327 |
| 3458 | cg00838259 |
| 3459 | cg24545533 |
| 3460 | cg23133335 |
| 3461 | cg08154101 |
| 3462 | cg18979916 |
| 3463 | cg01833675 |
| 3464 | cg27248297 |
| 3465 | cg26889362 |
| 3466 | cg05639508 |
| 3467 | cg09234983 |
| 3468 | cg14950747 |
| 3469 | cg02741882 |
| 3470 | cg14353573 |
| 3471 | cg16569525 |
| 3472 | cg11796442 |
| 3473 | cg08072911 |
| 3474 | cg08575537 |
| 3475 | cg02274869 |
| 3476 | cg15059143 |
| 3477 | cg00507008 |
| 3478 | cg20499141 |
| 3479 | cg25602225 |
| 3480 | cg21326590 |
| 3481 | cg17726575 |
| 3482 | cg00676711 |
| 3483 | cg14658818 |
| 3484 | cg17124387 |
| 3485 | cg07472199 |
| 3486 | cg04942251 |
| 3487 | cg15126544 |
| 3488 | cg09669275 |
| 3489 | cg17678844 |
| 3490 | cg00268840 |
| 3491 | cg11012153 |
| 3492 | cg10739015 |
| 3493 | cg11503833 |
| 3494 | cg10726445 |
| 3495 | cg13453847 |
| 3496 | cg04166294 |
| 3497 | cg18193094 |
| 3498 | cg08296680 |
| 3499 | cg00643333 |
| 3500 | cg18443437 |
| 3501 | cg16620526 |
| 3502 | cg12091936 |
| 3503 | cg25980637 |
| 3504 | cg04131898 |
| 3505 | cg00767344 |
| 3506 | cg05509609 |
| 3507 | cg24922968 |
| 3508 | cg22345877 |
| 3509 | cg14783368 |
| 3510 | cg16204357 |
| 3511 | cg16803846 |
| 3512 | cg01285926 |
| 3513 | cg25259754 |
| 3514 | cg05758861 |
| 3515 | cg25969107 |
| 3516 | cg16701167 |
| 3517 | cg06360427 |
| 3518 | cg20667684 |
| 3519 | cg26736888 |
| 3520 | cg12245236 |
| 3521 | cg19852660 |
| 3522 | cg21944224 |
| 3523 | cg14855519 |
| 3524 | cg05822888 |
| 3525 | cg00144816 |
| 3526 | cg06132400 |
| 3527 | cg14348741 |
| 3528 | cg05297315 |
| 3529 | cg00275686 |
| 3530 | cg22320071 |
| 3531 | cg23509699 |
| 3532 | cg13967521 |
| 3533 | cg20481941 |
| 3534 | cg13318911 |
| 3535 | cg13984040 |
| 3536 | cg26149924 |
| 3537 | cg26337010 |
| 3538 | cg16555417 |
| 3539 | cg18202521 |
| 3540 | cg15695738 |
| 3541 | cg12924936 |
| 3542 | cg08643646 |
| 3543 | cg13215579 |
| 3544 | cg08331733 |
| 3545 | cg07279157 |
| 3546 | cg10224345 |
| 3547 | cg21380154 |
| 3548 | cg15233992 |
| 3549 | cg22511262 |
| 3550 | cg25123566 |
| 3551 | cg19104015 |
| 3552 | cg04771669 |
| 3553 | cg18780769 |
| 3554 | cg09088153 |
| 3555 | cg07803474 |
| 3556 | cg26697125 |
| 3557 | cg26014401 |
| 3558 | cg07681579 |
| 3559 | cg13053861 |
| 3560 | cg01811815 |
| 3561 | cg02703731 |
| 3562 | cg23285459 |
| 3563 | cg17518215 |
| 3564 | cg02556649 |
| 3565 | cg14929496 |
| 3566 | cg24423468 |
| 3567 | cg18789515 |
| 3568 | cg07211212 |
| 3569 | cg19091930 |
| 3570 | cg07138399 |
| 3571 | cg09997546 |
| 3572 | cg00219816 |
| 3573 | cg15789211 |
| 3574 | cg09895325 |
| 3575 | cg16863619 |
| 3576 | cg07992457 |
| 3577 | cg21724239 |
| 3578 | cg08832227 |
| 3579 | cg12088543 |
| 3580 | cg04907257 |
| 3581 | cg25067210 |
| 3582 | cg07694621 |
| 3583 | cg08340755 |
| 3584 | cg10068883 |
| 3585 | cg10148755 |
| 3586 | cg05162306 |
| 3587 | cg06324373 |
| 3588 | cg24113610 |
| 3589 | cg05158633 |
| 3590 | cg09173768 |
| 3591 | cg03499943 |
| 3592 | cg17603132 |
| 3593 | cg11899535 |
| 3594 | cg03957481 |
| 3595 | cg02554168 |
| 3596 | cg23592288 |
| 3597 | cg08041408 |
| 3598 | cg14231297 |
| 3599 | cg07026433 |
| 3600 | cg03490406 |
| 3601 | cg16730194 |
| 3602 | cg04330597 |
| 3603 | cg06849778 |
| 3604 | cg17964510 |
| 3605 | cg11124262 |
| 3606 | cg27376707 |
| 3607 | cg10133888 |
| 3608 | cg11377086 |
| 3609 | cg10761553 |
| 3610 | cg15139637 |
| 3611 | cg27644795 |
| 3612 | cg17475883 |
| 3613 | cg03388786 |
| 3614 | cg06768361 |
| 3615 | cg00188095 |
| 3616 | cg16585554 |
| 3617 | cg17881572 |
| 3618 | cg08035822 |
| 3619 | cg19211853 |
| 3620 | cg19846991 |
| 3621 | cg07995570 |
| 3622 | cg17603537 |
| 3623 | cg05524354 |
| 3624 | cg07542748 |
| 3625 | cg02408252 |
| 3626 | cg06760904 |
| 3627 | cg21238327 |
| 3628 | cg15001131 |
| 3629 | cg25899135 |
| 3630 | cg10075506 |
| 3631 | cg27077475 |
| 3632 | cg06793974 |
| 3633 | cg14088357 |
| 3634 | cg17852290 |
| 3635 | cg10931190 |
| 3636 | cg03999434 |
| 3637 | cg26716823 |
| 3638 | cg19368440 |
| 3639 | cg04768602 |
| 3640 | cg22985016 |
| 3641 | cg08144675 |
| 3642 | cg05593641 |
| 3643 | cg22502382 |
| 3644 | cg12979234 |
| 3645 | cg06167563 |
| 3646 | cg22567464 |
| 3647 | cg10925082 |
| 3648 | cg26334826 |
| 3649 | cg01318967 |
| 3650 | cg19605788 |
| 3651 | cg02083836 |
| 3652 | cg14185777 |
| 3653 | cg10213875 |
| 3654 | cg12524488 |
| 3655 | cg12621429 |
| 3656 | cg00055747 |
| 3657 | cg05124021 |
| 3658 | cg00901687 |
| 3659 | cg21100077 |
| 3660 | cg13020870 |
| 3661 | cg08931647 |
| 3662 | cg16754081 |
| 3663 | cg19788250 |
| 3664 | cg06492744 |
| 3665 | cg25561875 |
| 3666 | cg12721835 |
| 3667 | cg20849109 |
| 3668 | cg04576672 |
| 3669 | cg04862328 |
| 3670 | cg07489959 |
| 3671 | cg14266770 |
| 3672 | cg12012404 |
| 3673 | cg19907365 |
| 3674 | cg08850922 |
| 3675 | cg11867697 |
| 3676 | cg16562275 |
| 3677 | cg12553908 |
| 3678 | cg21986657 |
| 3679 | cg04978078 |
| 3680 | cg09390241 |
| 3681 | cg25663823 |
| 3682 | cg03049691 |
| 3683 | cg05371552 |
| 3684 | cg17987982 |
| 3685 | cg11729679 |
| 3686 | cg05522011 |
| 3687 | cg16298867 |
| 3688 | cg24707354 |
| 3689 | cg22171525 |
| 3690 | cg09433910 |
| 3691 | cg05049329 |
| 3692 | cg02693988 |
| 3693 | cg17088908 |
| 3694 | cg12287358 |
| 3695 | cg01984802 |
| 3696 | cg04227922 |
| 3697 | cg06022561 |
| 3698 | cg27275023 |
| 3699 | cg02140579 |
| 3700 | cg14722290 |
| 3701 | cg20722210 |
| 3702 | cg00830401 |
| 3703 | cg17481912 |
| 3704 | cg05327736 |
| 3705 | cg11160054 |
| 3706 | cg26819590 |
| 3707 | cg03497845 |
| 3708 | cg09549987 |
| 3709 | cg23526298 |
| 3710 | cg26927190 |
| 3711 | cg09597022 |
| 3712 | cg21528927 |
| 3713 | cg22023206 |
| 3714 | cg26800883 |
| 3715 | cg09180702 |
| 3716 | cg14622790 |
| 3717 | cg16670359 |
| 3718 | cg05917460 |
| 3719 | cg15846193 |
| 3720 | cg20415947 |
| 3721 | cg07150062 |
| 3722 | cg03548857 |
| 3723 | cg17787084 |
| 3724 | cg05383947 |
| 3725 | cg10317717 |
| 3726 | cg03140929 |
| 3727 | cg09119854 |
| 3728 | cg02648943 |
| 3729 | cg14024893 |
| 3730 | cg12869305 |
| 3731 | cg06132620 |
| 3732 | cg25805675 |
| 3733 | cg02963248 |
| 3734 | cg18309927 |
| 3735 | cg25661986 |
| 3736 | cg26051413 |
| 3737 | cg19939094 |
| 3738 | cg16017420 |
| 3739 | cg05907046 |
| 3740 | cg19212949 |
| 3741 | cg07535010 |
| 3742 | cg10277044 |
| 3743 | cg01148113 |
| 3744 | cg12131894 |
| 3745 | cg13158784 |
| 3746 | cg01165853 |
| 3747 | cg00525823 |
| 3748 | cg16342115 |
| 3749 | cg24698655 |
| 3750 | cg12865888 |
| 3751 | cg07429146 |
| 3752 | cg11917067 |
| 3753 | cg00161124 |
| 3754 | cg20972027 |
| 3755 | cg23986590 |
| 3756 | cg25775667 |
| 3757 | cg07480006 |
| 3758 | cg03205252 |
| 3759 | cg12415479 |
| 3760 | cg03789891 |
| 3761 | cg01516851 |
| 3762 | cg05364567 |
| 3763 | cg11737759 |
| 3764 | cg24658517 |
| 3765 | cg02957700 |
| 3766 | cg25698564 |
| 3767 | cg04196395 |
| 3768 | cg07544232 |
| 3769 | cg09182138 |
| 3770 | cg11263372 |
| 3771 | cg17537347 |
| 3772 | cg03624899 |
| 3773 | cg21568145 |
| 3774 | cg17262810 |
| 3775 | cg23041109 |
| 3776 | cg15373911 |
| 3777 | cg00056433 |
| 3778 | cg21657758 |
| 3779 | cg22272840 |
| 3780 | cg26133399 |
| 3781 | cg11954236 |
| 3782 | cg24527098 |
| 3783 | cg05592035 |
| 3784 | cg07102210 |
| 3785 | cg04917181 |
| 3786 | cg08817540 |
| 3787 | cg13325904 |
| 3788 | cg05575544 |
| 3789 | cg09626540 |
| 3790 | cg27106643 |
| 3791 | cg10154780 |
| 3792 | cg19461383 |
| 3793 | cg23899408 |
| 3794 | cg14206182 |
| 3795 | cg27088150 |
| 3796 | cg26418900 |
| 3797 | cg18494134 |
| 3798 | cg11690979 |
| 3799 | cg05380888 |
| 3800 | cg07536144 |
| 3801 | cg11378364 |
| 3802 | cg01158680 |
| 3803 | cg03225210 |
| 3804 | cg14684434 |
| 3805 | cg07204522 |
| 3806 | cg02492205 |
| 3807 | cg17945789 |
| 3808 | cg26998537 |
| 3809 | cg18650670 |
| 3810 | cg26143452 |
| 3811 | cg06263495 |
| 3812 | cg19403132 |
| 3813 | cg16416322 |
| 3814 | cg15095288 |
| 3815 | cg22089922 |
| 3816 | cg12787836 |
| 3817 | cg08170294 |
| 3818 | cg17491368 |
| 3819 | cg24740322 |
| 3820 | cg10785505 |
| 3821 | cg11222700 |
| 3822 | cg08704934 |
| 3823 | cg27502457 |
| 3824 | cg05026881 |
| 3825 | cg12536660 |
| 3826 | cg22915945 |
| 3827 | cg24044052 |
| 3828 | cg15627089 |
| 3829 | cg02987928 |
| 3830 | cg05336808 |
| 3831 | cg00502190 |
| 3832 | cg01148860 |
| 3833 | cg18799491 |
| 3834 | cg05005343 |
| 3835 | cg06086698 |
| 3836 | cg20185461 |
| 3837 | cg14096615 |
| 3838 | cg25464921 |
| 3839 | cg03901576 |
| 3840 | cg20907669 |
| 3841 | cg21683390 |
| 3842 | cg14213590 |
| 3843 | cg22877175 |
| 3844 | cg09557387 |
| 3845 | cg00537837 |
| 3846 | cg26038971 |
| 3847 | cg24563501 |
| 3848 | cg14182517 |
| 3849 | cg11885415 |
| 3850 | cg00858840 |
| 3851 | cg12098307 |
| 3852 | cg23052560 |
| 3853 | cg00409356 |
| 3854 | cg24675442 |
| 3855 | cg17518809 |
| 3856 | cg10272901 |
| 3857 | cg24107163 |
| 3858 | cg04060356 |
| 3859 | cg18801579 |
| 3860 | cg26552886 |
| 3861 | cg07673080 |
| 3862 | cg01663148 |
| 3863 | cg09264065 |
| 3864 | cg20003494 |
| 3865 | cg16935031 |
| 3866 | cg10080732 |
| 3867 | cg12848065 |
| 3868 | cg15355400 |
| 3869 | cg16668359 |
| 3870 | cg09532694 |
| 3871 | cg07790104 |
| 3872 | cg24945222 |
| 3873 | cg05753693 |
| 3874 | cg23775883 |
| 3875 | cg15634980 |
| 3876 | cg18447419 |
| 3877 | cg24129356 |
| 3878 | cg07689252 |
| 3879 | cg17410650 |
| 3880 | cg25592314 |
| 3881 | cg14296274 |
| 3882 | cg23190089 |
| 3883 | cg21109192 |
| 3884 | cg15386366 |
| 3885 | cg18793449 |
| 3886 | cg09834794 |
| 3887 | cg07075347 |
| 3888 | cg07067577 |
| 3889 | cg11505113 |
| 3890 | cg15291243 |
| 3891 | cg11823511 |
| 3892 | cg26873457 |
| 3893 | cg13671044 |
| 3894 | cg16975587 |
| 3895 | cg00907810 |
| 3896 | cg10818657 |
| 3897 | cg25693439 |
| 3898 | cg17178900 |
| 3899 | cg05965387 |
| 3900 | cg18088723 |
| 3901 | cg03194064 |
| 3902 | cg08169827 |
| 3903 | cg19683821 |
| 3904 | cg14097568 |
| 3905 | cg12632411 |
| 3906 | cg01048962 |
| 3907 | cg04295144 |
| 3908 | cg07302580 |
| 3909 | cg11470848 |
| 3910 | cg26835826 |
| 3911 | cg05514909 |
| 3912 | cg04719903 |
| 3913 | cg19287610 |
| 3914 | cg26182406 |
| 3915 | cg12404281 |
| 3916 | cg03404134 |
| 3917 | cg05349245 |
| 3918 | cg20465980 |
| 3919 | cg15402627 |
| 3920 | cg06669374 |
| 3921 | cg11178337 |
| 3922 | cg05716270 |
| 3923 | cg26738728 |
| 3924 | cg10204945 |
| 3925 | cg27254040 |
| 3926 | cg07658449 |
| 3927 | cg14364460 |
| 3928 | cg23567719 |
| 3929 | cg06263106 |
| 3930 | cg26096887 |
| 3931 | cg15434587 |
| 3932 | cg26092675 |
| 3933 | cg15883853 |
| 3934 | cg17818471 |
| 3935 | cg04649215 |
| 3936 | cg24203758 |
| 3937 | cg25623524 |
| 3938 | cg13689699 |
| 3939 | cg11612354 |
| 3940 | cg25674613 |
| 3941 | cg23253309 |
| 3942 | cg05197760 |
| 3943 | cg20588069 |
| 3944 | cg19395056 |
| 3945 | cg07831351 |
| 3946 | cg15083799 |
| 3947 | cg20379806 |
| 3948 | cg18305394 |
| 3949 | cg00484216 |
| 3950 | cg18825101 |
| 3951 | cg18434074 |
| 3952 | cg24289452 |
| 3953 | cg21689511 |
| 3954 | cg12250142 |
| 3955 | cg05617980 |
| 3956 | cg16197717 |
| 3957 | cg10222789 |
| 3958 | cg26318645 |
| 3959 | cg20899271 |
| 3960 | cg04447643 |
| 3961 | cg07488576 |
| 3962 | cg08005697 |
| 3963 | cg25587662 |
| 3964 | cg25856220 |
| 3965 | cg25305879 |
| 3966 | cg05504925 |
| 3967 | cg25006194 |
| 3968 | cg24660631 |
| 3969 | cg16951942 |
| 3970 | cg15279308 |
| 3971 | cg05428701 |
| 3972 | cg21988950 |
| 3973 | cg07959016 |
| 3974 | cg27072996 |
| 3975 | cg26290543 |
| 3976 | cg16542356 |
| 3977 | cg00076538 |
| 3978 | cg08876274 |
| 3979 | cg08249764 |
| 3980 | cg17272620 |
| 3981 | cg23471446 |
| 3982 | cg17514766 |
| 3983 | cg08355260 |
| 3984 | cg10761315 |
| 3985 | cg18912855 |
| 3986 | cg13340765 |
| 3987 | cg09830866 |
| 3988 | cg12869217 |
| 3989 | cg07954971 |
| 3990 | cg02687418 |
| 3991 | cg20945055 |
| 3992 | cg21468671 |
| 3993 | cg24592370 |
| 3994 | cg12755421 |
| 3995 | cg18764814 |
| 3996 | cg08111960 |
| 3997 | cg12139725 |
| 3998 | cg22726407 |
| 3999 | cg09619282 |
| 4000 | cg01045113 |
| 4001 | cg16906346 |
| 4002 | cg18121434 |
| 4003 | cg00110660 |
| 4004 | cg03081478 |
| 4005 | cg07059972 |
| 4006 | cg27207756 |
| 4007 | cg15820574 |
| 4008 | cg09226986 |
| 4009 | cg07796002 |
| 4010 | cg00676294 |
| 4011 | cg26348995 |
| 4012 | cg24029091 |
| 4013 | cg06930314 |
| 4014 | cg11916257 |
| 4015 | cg23516310 |
| 4016 | cg07019450 |
| 4017 | cg19536929 |
| 4018 | cg26731143 |
| 4019 | cg16991765 |
| 4020 | cg21129181 |
| 4021 | cg07525395 |
| 4022 | cg03885198 |
| 4023 | cg15197065 |
| 4024 | cg26222247 |
| 4025 | cg15916004 |
| 4026 | cg21572599 |
| 4027 | cg17109893 |
| 4028 | cg02902946 |
| 4029 | cg17207944 |
| 4030 | cg12754571 |
| 4031 | cg08419873 |
| 4032 | cg07565505 |
| 4033 | cg08708961 |
| 4034 | cg19533760 |
| 4035 | cg22121557 |
| 4036 | cg10085750 |
| 4037 | cg04113257 |
| 4038 | cg18406033 |
| 4039 | cg20393890 |
| 4040 | cg17970299 |
| 4041 | cg01414882 |
| 4042 | cg04767772 |
| 4043 | cg19329160 |
| 4044 | cg14799482 |
| 4045 | cg20012977 |
| 4046 | cg07473340 |
| 4047 | cg03739132 |
| 4048 | cg13933734 |
| 4049 | cg06441252 |
| 4050 | cg11203293 |
| 4051 | cg24833737 |
| 4052 | cg12828896 |
| 4053 | cg03864958 |
| 4054 | cg00598772 |
| 4055 | cg04569837 |
| 4056 | cg05615449 |
| 4057 | cg06817490 |
| 4058 | cg23053506 |
| 4059 | cg22839308 |
| 4060 | cg08258526 |
| 4061 | cg05766510 |
| 4062 | cg22975913 |
| 4063 | cg07461953 |
| 4064 | cg14020846 |
| 4065 | cg00216138 |
| 4066 | cg12175038 |
| 4067 | cg27174108 |
| 4068 | cg02284273 |
| 4069 | cg23215476 |
| 4070 | cg00025197 |
| 4071 | cg19198938 |
| 4072 | cg19815010 |
| 4073 | cg21123519 |
| 4074 | cg14697761 |
| 4075 | cg01097384 |
| 4076 | cg01225942 |
| 4077 | cg01480180 |
| 4078 | cg22349363 |
| 4079 | cg02182210 |
| 4080 | cg20893203 |
| 4081 | cg00705808 |
| 4082 | cg00983948 |
| 4083 | cg19633390 |
| 4084 | cg22458973 |
| 4085 | cg06890161 |
| 4086 | cg20827174 |
| 4087 | cg12169661 |
| 4088 | cg03880642 |
| 4089 | cg05083852 |
| 4090 | cg14463995 |
| 4091 | cg24575852 |
| 4092 | cg11108890 |
| 4093 | cg00542147 |
| 4094 | cg19312667 |
| 4095 | cg14871035 |
| 4096 | cg09632271 |
| 4097 | cg00180073 |
| 4098 | cg19408207 |
| 4099 | cg03521696 |
| 4100 | cg16786640 |
| 4101 | cg16103959 |
| 4102 | cg24099331 |
| 4103 | cg00334177 |
| 4104 | cg01400004 |
| 4105 | cg09551145 |
| 4106 | cg18848606 |
| 4107 | cg27484582 |
| 4108 | cg27228433 |
| 4109 | cg08199758 |
| 4110 | cg18984452 |
| 4111 | cg04169483 |
| 4112 | cg25767345 |
| 4113 | cg05768427 |
| 4114 | cg23842170 |
| 4115 | cg25730098 |
| 4116 | cg19476376 |
| 4117 | cg08666915 |
| 4118 | cg13070763 |
| 4119 | cg14514991 |
| 4120 | cg23348723 |
| 4121 | cg05609218 |
| 4122 | cg26669984 |
| 4123 | cg00503253 |
| 4124 | cg14906976 |
| 4125 | cg08597799 |
| 4126 | cg03515494 |
| 4127 | cg21614723 |
| 4128 | cg16393530 |
| 4129 | cg23224619 |
| 4130 | cg14496909 |
| 4131 | cg27148718 |
| 4132 | cg15176213 |
| 4133 | cg01975684 |
| 4134 | cg27546066 |
| 4135 | cg26345105 |
| 4136 | cg06816685 |
| 4137 | cg03647861 |
| 4138 | cg08004377 |
| 4139 | cg16274678 |
| 4140 | cg21049093 |
| 4141 | cg14132895 |
| 4142 | cg24508249 |
| 4143 | cg04899990 |
| 4144 | cg01686178 |
| 4145 | cg07105152 |
| 4146 | cg14691529 |
| 4147 | cg04645545 |
| 4148 | cg02776659 |
| 4149 | cg12075247 |
| 4150 | cg26467753 |
| 4151 | cg00024102 |
| 4152 | cg08699196 |
| 4153 | cg23951474 |
| 4154 | cg01454698 |
| 4155 | cg26772540 |
| 4156 | cg24380001 |
| 4157 | cg25636665 |
| 4158 | cg00839584 |
| 4159 | cg11404502 |
| 4160 | cg08289130 |
| 4161 | cg03327154 |
| 4162 | cg18935575 |
| 4163 | cg26964636 |
| 4164 | cg25450929 |
| 4165 | cg12962191 |
| 4166 | cg22629626 |
| 4167 | cg21803847 |
| 4168 | cg16506815 |
| 4169 | cg22531668 |
| 4170 | cg13797425 |
| 4171 | cg01952835 |
| 4172 | cg22377389 |
| 4173 | cg12929940 |
| 4174 | cg07336045 |
| 4175 | cg27598576 |
| 4176 | cg07541823 |
| 4177 | cg01146063 |
| 4178 | cg06817454 |
| 4179 | cg23425669 |
| 4180 | cg02285920 |
| 4181 | cg13869942 |
| 4182 | cg14186145 |
| 4183 | cg12912139 |
| 4184 | cg16563470 |
| 4185 | cg19719930 |
| 4186 | cg04845065 |
| 4187 | cg09959931 |
| 4188 | cg10171448 |
| 4189 | cg00644317 |
| 4190 | cg04132540 |
| 4191 | cg11394600 |
| 4192 | cg08599096 |
| 4193 | cg15561880 |
| 4194 | cg01070987 |
| 4195 | cg07514040 |
| 4196 | cg24324837 |
| 4197 | cg02203737 |
| 4198 | cg20828424 |
| 4199 | cg27313147 |
| 4200 | cg19636519 |
| 4201 | cg08334034 |
| 4202 | cg16332610 |
| 4203 | cg06743153 |
| 4204 | cg20172627 |
| 4205 | cg11884030 |
| 4206 | cg14248883 |
| 4207 | cg13432682 |
| 4208 | cg16002234 |
| 4209 | cg15130102 |
| 4210 | cg04060128 |
| 4211 | cg02874061 |
| 4212 | cg20127859 |
| 4213 | cg03290223 |
| 4214 | cg00186701 |
| 4215 | cg26425256 |
| 4216 | cg12648074 |
| 4217 | cg11231751 |
| 4218 | cg01017409 |
| 4219 | cg08574993 |
| 4220 | cg11988765 |
| 4221 | cg08265950 |
| 4222 | cg06287548 |
| 4223 | cg07975212 |
| 4224 | cg08327690 |
| 4225 | cg15394602 |
| 4226 | cg27390009 |
| 4227 | cg27331241 |
| 4228 | cg11821245 |
| 4229 | cg22008841 |
| 4230 | cg13739417 |
| 4231 | cg12611488 |
| 4232 | cg09043518 |
| 4233 | cg12141340 |
| 4234 | cg06690318 |
| 4235 | cg03181524 |
| 4236 | cg15700197 |
| 4237 | cg08686960 |
| 4238 | cg04794430 |
| 4239 | cg08186708 |
| 4240 | cg17731776 |
| 4241 | cg18011099 |
| 4242 | cg01736784 |
| 4243 | cg23183559 |
| 4244 | cg09053081 |
| 4245 | cg02152985 |
| 4246 | cg02532824 |
| 4247 | cg09744501 |
| 4248 | cg06712127 |
| 4249 | cg21294861 |
| 4250 | cg13228355 |
| 4251 | cg15775211 |
| 4252 | cg08697310 |
| 4253 | cg21870545 |
| 4254 | cg07695771 |
| 4255 | cg10911287 |
| 4256 | cg02419849 |
| 4257 | cg03157917 |
| 4258 | cg20036996 |
| 4259 | cg01072076 |
| 4260 | cg04622200 |
| 4261 | cg06650659 |
| 4262 | cg07452510 |
| 4263 | cg13332571 |
| 4264 | cg10195901 |
| 4265 | cg02256049 |
| 4266 | cg00555816 |
| 4267 | cg06965072 |
| 4268 | cg03237606 |
| 4269 | cg18231054 |
| 4270 | cg04037970 |
| 4271 | cg01019875 |
| 4272 | cg05337681 |
| 4273 | cg16543561 |
| 4274 | cg00969162 |
| 4275 | cg08076018 |
| 4276 | cg21704050 |
| 4277 | cg14418835 |
| 4278 | cg07415496 |
| 4279 | cg20441902 |
| 4280 | cg13081009 |
| 4281 | cg12669543 |
| 4282 | cg27247092 |
| 4283 | cg12741994 |
| 4284 | cg10770023 |
| 4285 | cg06361936 |
| 4286 | cg02299441 |
| 4287 | cg18106923 |
| 4288 | cg08166750 |
| 4289 | cg03566582 |
| 4290 | cg19021961 |
| 4291 | cg11323985 |
| 4292 | cg08175609 |
| 4293 | cg17010254 |
| 4294 | cg05536998 |
| 4295 | cg07935832 |
| 4296 | cg18624930 |
| 4297 | cg11384744 |
| 4298 | cg06239982 |
| 4299 | cg01285501 |
| 4300 | cg13518883 |
| 4301 | cg09015433 |
| 4302 | cg27328965 |
| 4303 | cg22798223 |
| 4304 | cg12044337 |
| 4305 | cg08433999 |
| 4306 | cg06433467 |
| 4307 | cg12568743 |
| 4308 | cg13176243 |
| 4309 | cg23471393 |
| 4310 | cg00002033 |
| 4311 | cg05345876 |
| 4312 | cg10003213 |
| 4313 | cg09745256 |
| 4314 | cg08789978 |
| 4315 | cg25344322 |
| 4316 | cg02685896 |
| 4317 | cg01190459 |
| 4318 | cg22609785 |
| 4319 | cg18507379 |
| 4320 | cg14359824 |
| 4321 | cg10807084 |
| 4322 | cg11978884 |
| 4323 | cg21040133 |
| 4324 | cg04493700 |
| 4325 | cg27549720 |
| 4326 | cg02272576 |
| 4327 | cg07802710 |
| 4328 | cg23676302 |
| 4329 | cg06640991 |
| 4330 | cg04523558 |
| 4331 | cg06373377 |
| 4332 | cg21330711 |
| 4333 | cg14463412 |
| 4334 | cg01185626 |
| 4335 | cg10752406 |
| 4336 | cg17486396 |
| 4337 | cg03763518 |
| 4338 | cg25215230 |
| 4339 | cg17450308 |
| 4340 | cg20443946 |
| 4341 | cg25699925 |
| 4342 | cg07475394 |
| 4343 | cg26307117 |
| 4344 | cg03631656 |
| 4345 | cg03483713 |
| 4346 | cg14625947 |
| 4347 | cg03830443 |
| 4348 | cg12522875 |
| 4349 | cg24527881 |
| 4350 | cg01252526 |
| 4351 | cg15659284 |
| 4352 | cg11668053 |
| 4353 | cg12512875 |
| 4354 | cg25588787 |
| 4355 | cg11346248 |
| 4356 | cg16397176 |
| 4357 | cg04759376 |
| 4358 | cg16444607 |
| 4359 | cg03721890 |
| 4360 | cg13320632 |
| 4361 | cg00449201 |
| 4362 | cg01009664 |
| 4363 | cg13045555 |
| 4364 | cg11455582 |
| 4365 | cg14042635 |
| 4366 | cg27128545 |
| 4367 | cg03651054 |
| 4368 | cg12741420 |
| 4369 | cg08202380 |
| 4370 | cg21464454 |
| 4371 | cg05927763 |
| 4372 | cg10194536 |
| 4373 | cg24420947 |
| 4374 | cg20129035 |
| 4375 | cg27508620 |
| 4376 | cg04188920 |
| 4377 | cg06092694 |
| 4378 | cg03761670 |
| 4379 | cg18941614 |
| 4380 | cg02553516 |
| 4381 | cg06872599 |
| 4382 | cg12966367 |
| 4383 | cg23391107 |
| 4384 | cg03962421 |
| 4385 | cg15753746 |
| 4386 | cg09455118 |
| 4387 | cg27278787 |
| 4388 | cg13900763 |
| 4389 | cg25831439 |
| 4390 | cg20316883 |
| 4391 | cg05604079 |
| 4392 | cg18991240 |
| 4393 | cg08373528 |
| 4394 | cg17093995 |
| 4395 | cg13308964 |
| 4396 | cg11401794 |
| 4397 | cg23448348 |
| 4398 | cg10603275 |
| 4399 | cg05516755 |
| 4400 | cg27251219 |
| 4401 | cg10503705 |
| 4402 | cg01360333 |
| 4403 | cg24722746 |
| 4404 | cg05498905 |
| 4405 | cg17393917 |
| 4406 | cg22344727 |
| 4407 | cg06911744 |
| 4408 | cg11609571 |
| 4409 | cg10968849 |
| 4410 | cg07445742 |
| 4411 | cg13476469 |
| 4412 | cg26897989 |
| 4413 | cg25734089 |
| 4414 | cg12714890 |
| 4415 | cg17179862 |
| 4416 | cg14083146 |
| 4417 | cg06100756 |
| 4418 | cg03217587 |
| 4419 | cg04377176 |
| 4420 | cg25462303 |
| 4421 | cg12226948 |
| 4422 | cg14670938 |
| 4423 | cg21184340 |
| 4424 | cg01229061 |
| 4425 | cg06759351 |
| 4426 | cg03723716 |
| 4427 | cg02221572 |
| 4428 | cg08838158 |
| 4429 | cg22143678 |
| 4430 | cg14925246 |
| 4431 | cg01736164 |
| 4432 | cg21175823 |
| 4433 | cg04728978 |
| 4434 | cg01652514 |
| 4435 | cg05342515 |
| 4436 | cg07109453 |
| 4437 | cg21859546 |
| 4438 | cg02197629 |
| 4439 | cg15246170 |
| 4440 | cg15098809 |
| 4441 | cg04702010 |
| 4442 | cg18737356 |
| 4443 | cg15951466 |
| 4444 | cg11453061 |
| 4445 | cg16735465 |
| 4446 | cg01879420 |
| 4447 | cg09709123 |
| 4448 | cg26916621 |
| 4449 | cg13870494 |
| 4450 | cg04417677 |
| 4451 | cg16046375 |
| 4452 | cg19417168 |
| 4453 | cg15128334 |
| 4454 | cg01834022 |
| 4455 | cg06049826 |
| 4456 | cg12110659 |
| 4457 | cg26787220 |
| 4458 | cg07512361 |
| 4459 | cg19187616 |
| 4460 | cg14542120 |
| 4461 | cg04704021 |
| 4462 | cg00049440 |
| 4463 | cg09396851 |
| 4464 | cg24718516 |
| 4465 | cg10687129 |
| 4466 | cg18083933 |
| 4467 | cg26317050 |
| 4468 | cg25836915 |
| 4469 | cg08128274 |
| 4470 | cg01614237 |
| 4471 | cg27431446 |
| 4472 | cg20215112 |
| 4473 | cg00605063 |
| 4474 | cg26009192 |
| 4475 | cg01757312 |
| 4476 | cg20595752 |
| 4477 | cg07053114 |
| 4478 | cg22091609 |
| 4479 | cg12938556 |
| 4480 | cg15202804 |
| 4481 | cg15208687 |
| 4482 | cg17218064 |
| 4483 | cg19138253 |
| 4484 | cg00660643 |
| 4485 | cg06353069 |
| 4486 | cg16660891 |
| 4487 | cg00106564 |
| 4488 | cg02731032 |
| 4489 | cg04371726 |
| 4490 | cg07586008 |
| 4491 | cg20920008 |
| 4492 | cg09239524 |
| 4493 | cg22146018 |
| 4494 | cg20359042 |
| 4495 | cg21037935 |
| 4496 | cg01838004 |
| 4497 | cg17573415 |
| 4498 | cg27051129 |
| 4499 | cg14746276 |
| 4500 | cg17585615 |
| 4501 | cg03222361 |
| 4502 | cg11572717 |
| 4503 | cg09340279 |
| 4504 | cg01446627 |
| 4505 | cg26534425 |
| 4506 | cg19968789 |
| 4507 | cg13153466 |
| 4508 | cg25469923 |
| 4509 | cg18983669 |
| 4510 | cg08552718 |
| 4511 | cg15175838 |
| 4512 | cg13661519 |
| 4513 | cg22891070 |
| 4514 | cg07416664 |
| 4515 | cg11762346 |
| 4516 | cg00592643 |
| 4517 | cg15065591 |
| 4518 | cg27534083 |
| 4519 | cg12387154 |
| 4520 | cg24106790 |
| 4521 | cg09941363 |
| 4522 | cg02787917 |
| 4523 | cg13762691 |
| 4524 | cg17956145 |
| 4525 | cg15169940 |
| 4526 | cg10591607 |
| 4527 | cg00287773 |
| 4528 | cg02708705 |
| 4529 | cg02383228 |
| 4530 | cg08872590 |
| 4531 | cg01708617 |
| 4532 | cg09121047 |
| 4533 | cg17806401 |
| 4534 | cg22733626 |
| 4535 | cg22055891 |
| 4536 | cg21364077 |
| 4537 | cg00817355 |
| 4538 | cg25519619 |
| 4539 | cg12166520 |
| 4540 | cg19716643 |
| 4541 | cg11209624 |
| 4542 | cg05999983 |
| 4543 | cg05557673 |
| 4544 | cg03221652 |
| 4545 | cg24928023 |
| 4546 | cg19118951 |
| 4547 | cg05852568 |
| 4548 | cg14374754 |
| 4549 | cg16170767 |
| 4550 | cg17054650 |
| 4551 | cg05668342 |
| 4552 | cg21145524 |
| 4553 | cg02511570 |
| 4554 | cg25042939 |
| 4555 | cg14582550 |
| 4556 | cg08614290 |
| 4557 | cg07458509 |
| 4558 | cg12939447 |
| 4559 | cg08609318 |
| 4560 | cg03707169 |
| 4561 | cg13420177 |
| 4562 | cg20718640 |
| 4563 | cg08260245 |
| 4564 | cg13874780 |
| 4565 | cg14473344 |
| 4566 | cg21245816 |
| 4567 | cg02617721 |
| 4568 | cg02247178 |
| 4569 | cg17264909 |
| 4570 | cg08133631 |
| 4571 | cg23717517 |
| 4572 | cg22249789 |
| 4573 | cg20055426 |
| 4574 | cg10626548 |
| 4575 | cg17720259 |
| 4576 | cg01447112 |
| 4577 | cg26331172 |
| 4578 | cg18007837 |
| 4579 | cg06577400 |
| 4580 | cg21717724 |
| 4581 | cg08241330 |
| 4582 | cg13593391 |
| 4583 | cg14396008 |
| 4584 | cg19451167 |
| 4585 | cg13288038 |
| 4586 | cg08007485 |
| 4587 | cg21431715 |
| 4588 | cg08129583 |
| 4589 | cg24680632 |
| 4590 | cg17552979 |
| 4591 | cg00331101 |
| 4592 | cg02485963 |
| 4593 | cg09671951 |
| 4594 | cg02793451 |
| 4595 | cg03135704 |
| 4596 | cg05008972 |
| 4597 | cg14451791 |
| 4598 | cg05025860 |
| 4599 | cg02561771 |
| 4600 | cg16105461 |
| 4601 | cg16731240 |
| 4602 | cg18343437 |
| 4603 | cg16446288 |
| 4604 | cg02857760 |
| 4605 | cg03936663 |
| 4606 | cg02034689 |
| 4607 | cg16998150 |
| 4608 | cg06218079 |
| 4609 | cg12883479 |
| 4610 | cg19308222 |
| 4611 | cg00213822 |
| 4612 | cg04998590 |
| 4613 | cg02885925 |
| 4614 | cg10009482 |
| 4615 | cg07906828 |
| 4616 | cg07361047 |
| 4617 | cg03377528 |
| 4618 | cg16907566 |
| 4619 | cg27483342 |
| 4620 | cg18196829 |
| 4621 | cg06334965 |
| 4622 | cg13529733 |
| 4623 | cg11803843 |
| 4624 | cg03881854 |
| 4625 | cg26107333 |
| 4626 | cg07209071 |
| 4627 | cg16043757 |
| 4628 | cg00202266 |
| 4629 | cg05597836 |
| 4630 | cg04987431 |
| 4631 | cg05522466 |
| 4632 | cg22737448 |
| 4633 | cg07822859 |
| 4634 | cg16763089 |
| 4635 | cg03166753 |
| 4636 | cg04921068 |
| 4637 | cg24452128 |
| 4638 | cg16124670 |
| 4639 | cg21426404 |
| 4640 | cg00505093 |
| 4641 | cg05961151 |
| 4642 | cg01956293 |
| 4643 | cg00943809 |
| 4644 | cg20480274 |
| 4645 | cg21661379 |
| 4646 | cg10233674 |
| 4647 | cg21749794 |
| 4648 | cg06316886 |
| 4649 | cg23254302 |
| 4650 | cg05293861 |
| 4651 | cg22678136 |
| 4652 | cg05754643 |
| 4653 | cg09045731 |
| 4654 | cg11953749 |
| 4655 | cg15737177 |
| 4656 | cg27621721 |
| 4657 | cg14937614 |
| 4658 | cg06444400 |
| 4659 | cg26848395 |
| 4660 | cg19071879 |
| 4661 | cg11412853 |
| 4662 | cg25212090 |
| 4663 | cg14980042 |
| 4664 | cg27319662 |
| 4665 | cg06887285 |
| 4666 | cg27438152 |
| 4667 | cg11059159 |
| 4668 | cg16643902 |
| 4669 | cg06303238 |
| 4670 | cg21484940 |
| 4671 | cg24702357 |
| 4672 | cg09095356 |
| 4673 | cg24505341 |
| 4674 | cg10200298 |
| 4675 | cg11308335 |
| 4676 | cg07856071 |
| 4677 | cg14631871 |
| 4678 | cg00199007 |
| 4679 | cg04115185 |
| 4680 | cg01403243 |
| 4681 | cg07665510 |
| 4682 | cg04428163 |
| 4683 | cg03547924 |
| 4684 | cg16553731 |
| 4685 | cg02164556 |
| 4686 | cg06832339 |
| 4687 | cg17115881 |
| 4688 | cg18086678 |
| 4689 | cg13246055 |
| 4690 | cg16149927 |
| 4691 | cg08616663 |
| 4692 | cg00373061 |
| 4693 | cg08164500 |
| 4694 | cg23221052 |
| 4695 | cg00726615 |
| 4696 | cg04047221 |
| 4697 | cg10169241 |
| 4698 | cg02959108 |
| 4699 | cg19402991 |
| 4700 | cg18855674 |
| 4701 | cg21041775 |
| 4702 | cg01295093 |
| 4703 | cg20155035 |
| 4704 | cg11343211 |
| 4705 | cg04674680 |
| 4706 | cg17014757 |
| 4707 | cg15897845 |
| 4708 | cg07780543 |
| 4709 | cg03407747 |
| 4710 | cg09109520 |
| 4711 | cg19592277 |
| 4712 | cg14127947 |
| 4713 | cg13856142 |
| 4714 | cg06066088 |
| 4715 | cg09378456 |
| 4716 | cg23101649 |
| 4717 | cg22212414 |
| 4718 | cg08110170 |
| 4719 | cg11234839 |
| 4720 | cg19092981 |
| 4721 | cg14989243 |
| 4722 | cg22583148 |
| 4723 | cg02844545 |
| 4724 | cg19450111 |
| 4725 | cg18751168 |
| 4726 | cg17182270 |
| 4727 | cg19476597 |
| 4728 | cg17960517 |
| 4729 | cg15015639 |
| 4730 | cg10197383 |
| 4731 | cg26144451 |
| 4732 | cg02372723 |
| 4733 | cg19505399 |
| 4734 | cg04382470 |
| 4735 | cg14298457 |
| 4736 | cg06785806 |
| 4737 | cg10423771 |
| 4738 | cg09117448 |
| 4739 | cg14508231 |
| 4740 | cg02000275 |
| 4741 | cg25541653 |
| 4742 | cg16867086 |
| 4743 | cg20692268 |
| 4744 | cg09093142 |
| 4745 | cg01938496 |
| 4746 | cg15181598 |
| 4747 | cg19855622 |
| 4748 | cg06961024 |
| 4749 | cg12192716 |
| 4750 | cg00107682 |
| 4751 | cg05142677 |
| 4752 | cg14730102 |
| 4753 | cg11176481 |
| 4754 | cg05324194 |
| 4755 | cg09797337 |
| 4756 | cg10072269 |
| 4757 | cg11735242 |
| 4758 | cg16117273 |
| 4759 | cg25399162 |
| 4760 | cg14477868 |
| 4761 | cg15682262 |
| 4762 | cg18758433 |
| 4763 | cg04650653 |
| 4764 | cg08461752 |
| 4765 | cg26261172 |
| 4766 | cg13508175 |
| 4767 | cg08886020 |
| 4768 | cg03748376 |
| 4769 | cg27217474 |
| 4770 | cg16675758 |
| 4771 | cg07112131 |
| 4772 | cg12420683 |
| 4773 | cg23369234 |
| 4774 | cg03224396 |
| 4775 | cg24049493 |
| 4776 | cg17040309 |
| 4777 | cg06517373 |
| 4778 | cg20273931 |
| 4779 | cg27427491 |
| 4780 | cg21542223 |
| 4781 | cg05589784 |
| 4782 | cg15696900 |
| 4783 | cg01926319 |
| 4784 | cg15869743 |
| 4785 | cg16879110 |
| 4786 | cg01054402 |
| 4787 | cg16856285 |
| 4788 | cg27216368 |
| 4789 | cg09805403 |
| 4790 | cg19537111 |
| 4791 | cg18670770 |
| 4792 | cg27651480 |
| 4793 | cg06096061 |
| 4794 | cg07499899 |
| 4795 | cg20354777 |
| 4796 | cg25352836 |
| 4797 | cg00776398 |
| 4798 | cg20993403 |
| 4799 | cg24208134 |
| 4800 | cg06401976 |
| 4801 | cg00205586 |
| 4802 | cg13689252 |
| 4803 | cg17298326 |
| 4804 | cg11954332 |
| 4805 | cg16087940 |
| 4806 | cg00549876 |
| 4807 | cg14401746 |
| 4808 | cg04682087 |
| 4809 | cg26648185 |
| 4810 | cg10679688 |
| 4811 | cg07948866 |
| 4812 | cg19110943 |
| 4813 | cg13374172 |
| 4814 | cg14085177 |
| 4815 | cg25296938 |
| 4816 | cg11614941 |
| 4817 | cg14009912 |
| 4818 | cg19976205 |
| 4819 | cg07541744 |
| 4820 | cg21964148 |
| 4821 | cg09187944 |
| 4822 | cg03846571 |
| 4823 | cg10800941 |
| 4824 | cg18746831 |
| 4825 | cg05437823 |
| 4826 | cg07453773 |
| 4827 | cg16350123 |
| 4828 | cg08709360 |
| 4829 | cg17995197 |
| 4830 | cg18703066 |
| 4831 | cg15548295 |
| 4832 | cg06952307 |
| 4833 | cg12719030 |
| 4834 | cg01062116 |
| 4835 | cg14248575 |
| 4836 | cg07816074 |
| 4837 | cg10923428 |
| 4838 | cg06943590 |
| 4839 | cg12820294 |
| 4840 | cg20007410 |
| 4841 | cg26114124 |
| 4842 | cg12078775 |
| 4843 | cg04911919 |
| 4844 | cg03796140 |
| 4845 | cg01219345 |
| 4846 | cg26010877 |
| 4847 | cg01698298 |
| 4848 | cg10295858 |
| 4849 | cg06636541 |
| 4850 | cg26864950 |
| 4851 | cg22507772 |
| 4852 | cg16739441 |
| 4853 | cg12320351 |
| 4854 | cg23172664 |
| 4855 | cg05260411 |
| 4856 | cg22718636 |
| 4857 | cg24966502 |
| 4858 | cg05683630 |
| 4859 | cg11655515 |
| 4860 | cg22425745 |
| 4861 | cg23965720 |
| 4862 | cg14843013 |
| 4863 | cg04488894 |
| 4864 | cg23926743 |
| 4865 | cg13487054 |
| 4866 | cg14442890 |
| 4867 | cg03188064 |
| 4868 | cg10944297 |
| 4869 | cg07363212 |
| 4870 | cg00744433 |
| 4871 | cg12752420 |
| 4872 | cg07355507 |
| 4873 | cg03090940 |
| 4874 | cg25323137 |
| 4875 | cg24394732 |
| 4876 | cg00017842 |
| 4877 | cg19284726 |
| 4878 | cg03372423 |
| 4879 | cg03107649 |
| 4880 | cg23005885 |
| 4881 | cg27340001 |
| 4882 | cg15146462 |
| 4883 | cg12922751 |
| 4884 | cg09156097 |
| 4885 | cg12483545 |
| 4886 | cg24667437 |
| 4887 | cg04303382 |
| 4888 | cg08835103 |
| 4889 | cg14696870 |
| 4890 | cg05852395 |
| 4891 | cg13073814 |
| 4892 | cg22081832 |
| 4893 | cg04515533 |
| 4894 | cg01403596 |
| 4895 | cg21268578 |
| 4896 | cg06797880 |
| 4897 | cg19826026 |
| 4898 | cg06658697 |
| 4899 | cg23658987 |
| 4900 | cg17152177 |
| 4901 | cg26105687 |
| 4902 | cg08530838 |
| 4903 | cg15499368 |
| 4904 | cg21307466 |
| 4905 | cg07392358 |
| 4906 | cg01061391 |
| 4907 | cg20900312 |
| 4908 | cg14268695 |
| 4909 | cg09433738 |
| 4910 | cg01297721 |
| 4911 | cg11030264 |
| 4912 | cg03775802 |
| 4913 | cg14219071 |
| 4914 | cg18071489 |
| 4915 | cg17892321 |
| 4916 | cg08485649 |
| 4917 | cg16746901 |
| 4918 | cg00157012 |
| 4919 | cg26716099 |
| 4920 | cg20815683 |
| 4921 | cg19203800 |
| 4922 | cg13951664 |
| 4923 | cg02152631 |
| 4924 | cg12042952 |
| 4925 | cg20373822 |
| 4926 | cg25362339 |
| 4927 | cg15261665 |
| 4928 | cg22039909 |
| 4929 | cg22270593 |
| 4930 | cg14346074 |
| 4931 | cg21814875 |
| 4932 | cg01546563 |
| 4933 | cg17060838 |
| 4934 | cg13446906 |
| 4935 | cg09275704 |
| 4936 | cg15813051 |
| 4937 | cg00496602 |
| 4938 | cg04603130 |
| 4939 | cg26348243 |
| 4940 | cg27666046 |
| 4941 | cg26330116 |
| 4942 | cg09607488 |
| 4943 | cg01250801 |
| 4944 | cg06786372 |
| 4945 | cg12122408 |
| 4946 | cg20527831 |
| 4947 | cg13185005 |
| 4948 | cg23012310 |
| 4949 | cg07661904 |
| 4950 | cg23639072 |
| 4951 | cg07601212 |
| 4952 | cg01387450 |
| 4953 | cg01003539 |
| 4954 | cg05037556 |
| 4955 | cg12630714 |
| 4956 | cg08724920 |
| 4957 | cg14548259 |
| 4958 | cg26643967 |
| 4959 | cg14195925 |
| 4960 | cg26827328 |
| 4961 | cg12082522 |
| 4962 | cg01985330 |
| 4963 | cg04194341 |
| 4964 | cg06949629 |
| 4965 | cg16260889 |
| 4966 | cg01575590 |
| 4967 | cg08436089 |
| 4968 | cg22772930 |
| 4969 | cg08242458 |
| 4970 | cg27357306 |
| 4971 | cg03242507 |
| 4972 | cg21903286 |
| 4973 | cg19757176 |
| 4974 | cg26603656 |
| 4975 | cg26341112 |
| 4976 | cg19392699 |
| 4977 | cg01119452 |
| 4978 | cg25308354 |
| 4979 | cg12756686 |
| 4980 | cg18988435 |
| 4981 | cg00146027 |
| 4982 | cg06204040 |
| 4983 | cg17672101 |
| 4984 | cg02183671 |
| 4985 | cg11235391 |
| 4986 | cg15182493 |
| 4987 | cg22243996 |
| 4988 | cg05091658 |
| 4989 | cg10014470 |
| 4990 | cg19748086 |
| 4991 | cg21876918 |
| 4992 | cg09265718 |
| 4993 | cg20761187 |
| 4994 | cg06423355 |
| 4995 | cg14825976 |
| 4996 | cg09235562 |
| 4997 | cg08102185 |
| 4998 | cg10007680 |
| 4999 | cg13884765 |
| 5000 | cg24663971 |
| 5001 | cg27300829 |
| 5002 | cg02380802 |
| 5003 | cg21363515 |
| 5004 | cg00811436 |
| 5005 | cg01767614 |
| 5006 | cg00029256 |
| 5007 | cg18114294 |
| 5008 | cg02256631 |
| 5009 | cg19254118 |
| 5010 | cg09251078 |
| 5011 | cg20178976 |
| 5012 | cg10286153 |
| 5013 | cg16415710 |
| 5014 | cg16861968 |
| 5015 | cg00721530 |
| 5016 | cg24741066 |
| 5017 | cg13883027 |
| 5018 | cg17827650 |
| 5019 | cg07173299 |
| 5020 | cg01422797 |
| 5021 | cg22490254 |
| 5022 | cg02223801 |
| 5023 | cg26795333 |
| 5024 | cg10501093 |
| 5025 | cg21833554 |
| 5026 | cg20193363 |
| 5027 | cg25295659 |
| 5028 | cg15912800 |
| 5029 | cg24998349 |
| 5030 | cg03284879 |
| 5031 | cg07222863 |
| 5032 | cg02370667 |
| 5033 | cg17502037 |
| 5034 | cg09299133 |
| 5035 | cg08720517 |
| 5036 | cg00352543 |
| 5037 | cg07147475 |
| 5038 | cg02022733 |
| 5039 | cg11231500 |
| 5040 | cg06486618 |
| 5041 | cg05820861 |
| 5042 | cg21153620 |
| 5043 | cg16773564 |
| 5044 | cg23864180 |
| 5045 | cg20830514 |
| 5046 | cg03273803 |
| 5047 | cg06783429 |
| 5048 | cg08684511 |
| 5049 | cg00805880 |
| 5050 | cg23121156 |
| 5051 | cg15521792 |
| 5052 | cg27223728 |
| 5053 | cg02325434 |
| 5054 | cg20151221 |
| 5055 | cg11415067 |
| 5056 | cg16394702 |
| 5057 | cg19457763 |
| 5058 | cg21243944 |
| 5059 | cg03059635 |
| 5060 | cg22801992 |
| 5061 | cg10635122 |
| 5062 | cg07019828 |
| 5063 | cg00335715 |
| 5064 | cg23642130 |
| 5065 | cg00321404 |
| 5066 | cg03327829 |
| 5067 | cg22214889 |
| 5068 | cg05848337 |
| 5069 | cg20275841 |
| 5070 | cg10172783 |
| 5071 | cg07112337 |
| 5072 | cg24942922 |
| 5073 | cg02391394 |
| 5074 | cg00964997 |
| 5075 | cg00660445 |
| 5076 | cg03338298 |
| 5077 | cg13740929 |
| 5078 | cg00090674 |
| 5079 | cg02477603 |
| 5080 | cg04714994 |
| 5081 | cg04150429 |
| 5082 | cg17238766 |
| 5083 | cg09400037 |
| 5084 | cg23692401 |
| 5085 | cg19303898 |
| 5086 | cg22266001 |
| 5087 | cg18257996 |
| 5088 | cg12036817 |
| 5089 | cg24903860 |
| 5090 | cg01235000 |
| 5091 | cg07448060 |
| 5092 | cg16361302 |
| 5093 | cg24942023 |
| 5094 | cg01026256 |
| 5095 | cg27091865 |
| 5096 | cg04080282 |
| 5097 | cg17894854 |
| 5098 | cg13944494 |
| 5099 | cg01337207 |
| 5100 | cg08202226 |
| 5101 | cg13226232 |
| 5102 | cg26657404 |
| 5103 | cg13019092 |
| 5104 | cg20566840 |
| 5105 | cg23668057 |
| 5106 | cg11646124 |
| 5107 | cg13583664 |
| 5108 | cg09038944 |
| 5109 | cg10531355 |
| 5110 | cg09560586 |
| 5111 | cg00522231 |
| 5112 | cg13571460 |
| 5113 | cg14019044 |
| 5114 | cg12511331 |
| 5115 | cg10003689 |
| 5116 | cg08877188 |
| 5117 | cg03400060 |
| 5118 | cg26335937 |
| 5119 | cg26697605 |
| 5120 | cg06379435 |
| 5121 | cg03770907 |
| 5122 | cg16822666 |
| 5123 | cg07676517 |
| 5124 | cg09454156 |
| 5125 | cg06536724 |
| 5126 | cg09973548 |
| 5127 | cg11062466 |
| 5128 | cg05979457 |
| 5129 | cg02611419 |
| 5130 | cg23911285 |
| 5131 | cg25442600 |
| 5132 | cg22747501 |
| 5133 | cg04182992 |
| 5134 | cg03330558 |
| 5135 | cg18980148 |
| 5136 | cg04674201 |
| 5137 | cg25191235 |
| 5138 | cg04599341 |
| 5139 | cg09330795 |
| 5140 | cg01828742 |
| 5141 | cg12708821 |
| 5142 | cg21363233 |
| 5143 | cg17070064 |
| 5144 | cg21871952 |
| 5145 | cg02608914 |
| 5146 | cg06893273 |
| 5147 | cg08583841 |
| 5148 | cg21652471 |
| 5149 | cg00848997 |
| 5150 | cg25246421 |
| 5151 | cg25051331 |
| 5152 | cg24412699 |
| 5153 | cg02891314 |
| 5154 | cg03082589 |
| 5155 | cg24726200 |
| 5156 | cg11401293 |
| 5157 | cg13861904 |
| 5158 | cg06899985 |
| 5159 | cg17622053 |
| 5160 | cg23248424 |
| 5161 | cg27507960 |
| 5162 | cg14206684 |
| 5163 | cg01083910 |
| 5164 | cg06842071 |
| 5165 | cg07084627 |
| 5166 | cg07318204 |
| 5167 | cg18166150 |
| 5168 | cg13786863 |
| 5169 | cg26802256 |
| 5170 | cg15896939 |
| 5171 | cg15593510 |
| 5172 | cg19597227 |
| 5173 | cg08024887 |
| 5174 | cg01323777 |
| 5175 | cg00333528 |
| 5176 | cg06706310 |
| 5177 | cg10022788 |
| 5178 | cg27612638 |
| 5179 | cg15858483 |
| 5180 | cg04749615 |
| 5181 | cg15422147 |
| 5182 | cg25313930 |
| 5183 | cg12970547 |
| 5184 | cg04518186 |
| 5185 | cg19307112 |
| 5186 | cg07834574 |
| 5187 | cg27331665 |
| 5188 | cg24084719 |
| 5189 | cg24878594 |
| 5190 | cg22485693 |
| 5191 | cg13207490 |
| 5192 | cg05032870 |
| 5193 | cg03198009 |
| 5194 | cg03421195 |
| 5195 | cg13645530 |
| 5196 | cg27014538 |
| 5197 | cg05385453 |
| 5198 | cg22658846 |
| 5199 | cg10885151 |
| 5200 | cg19218509 |
| 5201 | cg13897134 |
| 5202 | cg25550323 |
| 5203 | cg02631462 |
| 5204 | cg21695771 |
| 5205 | cg24261273 |
| 5206 | cg26260558 |
| 5207 | cg19092476 |
| 5208 | cg09851300 |
| 5209 | cg20973210 |
| 5210 | cg13670288 |
| 5211 | cg13152974 |
| 5212 | cg27165836 |
| 5213 | cg01984298 |
| 5214 | cg27225975 |
| 5215 | cg23737927 |
| 5216 | cg19732889 |
| 5217 | cg23630110 |
| 5218 | cg18210893 |
| 5219 | cg16251130 |
| 5220 | cg14194263 |
| 5221 | cg21208539 |
| 5222 | cg03297163 |
| 5223 | cg05496248 |
| 5224 | cg24935217 |
| 5225 | cg19184455 |
| 5226 | cg23936361 |
| 5227 | cg26465602 |
| 5228 | cg10671668 |
| 5229 | cg08276645 |
| 5230 | cg21041888 |
| 5231 | cg02133809 |
| 5232 | cg27109043 |
| 5233 | cg03553715 |
| 5234 | cg18680969 |
| 5235 | cg23279756 |
| 5236 | cg18651072 |
| 5237 | cg16963373 |
| 5238 | cg26094554 |
| 5239 | cg15636391 |
| 5240 | cg04273289 |
| 5241 | cg09164108 |
| 5242 | cg02921273 |
| 5243 | cg04503968 |
| 5244 | cg21911021 |
| 5245 | cg07076915 |
| 5246 | cg25514833 |
| 5247 | cg01282504 |
| 5248 | cg03177551 |
| 5249 | cg25514244 |
| 5250 | cg06654118 |
| 5251 | cg07442343 |
| 5252 | cg20300343 |
| 5253 | cg09001573 |
| 5254 | cg24843609 |
| 5255 | cg00155526 |
| 5256 | cg27137285 |
| 5257 | cg04786750 |
| 5258 | cg17847560 |
| 5259 | cg17366166 |
| 5260 | cg21034095 |
| 5261 | cg20811857 |
| 5262 | cg14860676 |
| 5263 | cg23482747 |
| 5264 | cg05162545 |
| 5265 | cg27228611 |
| 5266 | cg01212326 |
| 5267 | cg24454109 |
| 5268 | cg03253704 |
| 5269 | cg10332649 |
| 5270 | cg07964754 |
| 5271 | cg06400119 |
| 5272 | cg25169679 |
| 5273 | cg08180229 |
| 5274 | cg12143028 |
| 5275 | cg18882786 |
| 5276 | cg05683379 |
| 5277 | cg21236655 |
| 5278 | cg12263124 |
| 5279 | cg07227743 |
| 5280 | cg10589396 |
| 5281 | cg15449217 |
| 5282 | cg16446486 |
| 5283 | cg01093476 |
| 5284 | cg02444433 |
| 5285 | cg27527798 |
| 5286 | cg19308375 |
| 5287 | cg09711085 |
| 5288 | cg26784201 |
| 5289 | cg16257051 |
| 5290 | cg17537073 |
| 5291 | cg01770296 |
| 5292 | cg02694058 |
| 5293 | cg03166163 |
| 5294 | cg06525670 |
| 5295 | cg22237300 |
| 5296 | cg24574963 |
| 5297 | cg23227889 |
| 5298 | cg17977250 |
| 5299 | cg27400205 |
| 5300 | cg18235100 |
| 5301 | cg06419850 |
| 5302 | cg02749784 |
| 5303 | cg15742712 |
| 5304 | cg16265717 |
| 5305 | cg01617516 |
| 5306 | cg09409956 |
| 5307 | cg13104274 |
| 5308 | cg09317884 |
| 5309 | cg24342814 |
| 5310 | cg17641649 |
| 5311 | cg27658314 |
| 5312 | cg12103742 |
| 5313 | cg03555861 |
| 5314 | cg26745220 |
| 5315 | cg08882547 |
| 5316 | cg02879554 |
| 5317 | cg07146073 |
| 5318 | cg21180726 |
| 5319 | cg00210562 |
| 5320 | cg14258623 |
| 5321 | cg17286831 |
| 5322 | cg10682957 |
| 5323 | cg02094273 |
| 5324 | cg11239407 |
| 5325 | cg19223137 |
| 5326 | cg05822532 |
| 5327 | cg05571310 |
| 5328 | cg16674866 |
| 5329 | cg15915632 |
| 5330 | cg01939675 |
| 5331 | cg16817992 |
| 5332 | cg07107916 |
| 5333 | cg23206851 |
| 5334 | cg26557696 |
| 5335 | cg07834490 |
| 5336 | cg00105060 |
| 5337 | cg00691026 |
| 5338 | cg21584831 |
| 5339 | cg05160879 |
| 5340 | cg04754315 |
| 5341 | cg20356637 |
| 5342 | cg01391631 |
| 5343 | cg11717969 |
| 5344 | cg07828472 |
| 5345 | cg20095609 |
| 5346 | cg20122518 |
| 5347 | cg00849267 |
| 5348 | cg14758937 |
| 5349 | cg16711124 |
| 5350 | cg01663665 |
| 5351 | cg19878806 |
| 5352 | cg12227036 |
| 5353 | cg09744391 |
| 5354 | cg26457248 |
| 5355 | cg24142849 |
| 5356 | cg06375949 |
| 5357 | cg01003893 |
| 5358 | cg15832560 |
| 5359 | cg02586236 |
| 5360 | cg14982276 |
| 5361 | cg09183220 |
| 5362 | cg16054907 |
| 5363 | cg20679626 |
| 5364 | cg13393830 |
| 5365 | cg00483315 |
| 5366 | cg26700215 |
| 5367 | cg26471497 |
| 5368 | cg02558625 |
| 5369 | cg02394955 |
| 5370 | cg26817371 |
| 5371 | cg16709904 |
| 5372 | cg23123017 |
| 5373 | cg17814030 |
| 5374 | cg16901197 |
| 5375 | cg21117734 |
| 5376 | cg12298429 |
| 5377 | cg08373515 |
| 5378 | cg06984903 |
| 5379 | cg15656203 |
| 5380 | cg03320508 |
| 5381 | cg22663463 |
| 5382 | cg08352755 |
| 5383 | cg07474682 |
| 5384 | cg23489072 |
| 5385 | cg25370039 |
| 5386 | cg24073122 |
| 5387 | cg18150909 |
| 5388 | cg07260778 |
| 5389 | cg06550539 |
| 5390 | cg15095952 |
| 5391 | cg09439560 |
| 5392 | cg14372281 |
| 5393 | cg23201907 |
| 5394 | cg06960164 |
| 5395 | cg02034205 |
| 5396 | cg07834841 |
| 5397 | cg08960549 |
| 5398 | cg11179284 |
| 5399 | cg25461308 |
| 5400 | cg22368204 |
| 5401 | cg01303385 |
| 5402 | cg20177995 |
| 5403 | cg03927133 |
| 5404 | cg05887508 |
| 5405 | cg24828869 |
| 5406 | cg25566442 |
| 5407 | cg21175685 |
| 5408 | cg25072336 |
| 5409 | cg06116009 |
| 5410 | cg10571588 |
| 5411 | cg05103917 |
| 5412 | cg12091498 |
| 5413 | cg13823492 |
| 5414 | cg17896987 |
| 5415 | cg08717672 |
| 5416 | cg23463206 |
| 5417 | cg23523937 |
| 5418 | cg07867517 |
| 5419 | cg11542165 |
| 5420 | cg04768479 |
| 5421 | cg11268834 |
| 5422 | cg22329875 |
| 5423 | cg25912517 |
| 5424 | cg21371012 |
| 5425 | cg01135546 |
| 5426 | cg21287890 |
| 5427 | cg04326863 |
| 5428 | cg08306218 |
| 5429 | cg27650906 |
| 5430 | cg13346442 |
| 5431 | cg25037165 |
| 5432 | cg01933308 |
| 5433 | cg01366882 |
| 5434 | cg19570321 |
| 5435 | cg12878815 |
| 5436 | cg26628164 |
| 5437 | cg16527405 |
| 5438 | cg13403455 |
| 5439 | cg02959939 |
| 5440 | cg24706275 |
| 5441 | cg02376677 |
| 5442 | cg06355129 |
| 5443 | cg18576936 |
| 5444 | cg15339596 |
| 5445 | cg01449089 |
| 5446 | cg22887597 |
| 5447 | cg13567542 |
| 5448 | cg24213719 |
| 5449 | cg05118430 |
| 5450 | cg01577414 |
| 5451 | cg07501635 |
| 5452 | cg24122814 |
| 5453 | cg26852928 |
| 5454 | cg18067932 |
| 5455 | cg02934237 |
| 5456 | cg18649451 |
| 5457 | cg12247222 |
| 5458 | cg25019274 |
| 5459 | cg18586712 |
| 5460 | cg20811236 |
| 5461 | cg18077580 |
| 5462 | cg01260325 |
| 5463 | cg13230640 |
| 5464 | cg14678774 |
| 5465 | cg26724975 |
| 5466 | cg00955911 |
| 5467 | cg27500194 |
| 5468 | cg02798614 |
| 5469 | cg12589308 |
| 5470 | cg08043592 |
| 5471 | cg23257225 |
| 5472 | cg15227982 |
| 5473 | cg21827674 |
| 5474 | cg12190768 |
| 5475 | cg15823097 |
| 5476 | cg25054666 |
| 5477 | cg12038696 |
| 5478 | cg13894136 |
| 5479 | cg15847015 |
| 5480 | cg19235198 |
| 5481 | cg00755146 |
| 5482 | cg23610545 |
| 5483 | cg19524238 |
| 5484 | cg08322008 |
| 5485 | cg26574334 |
| 5486 | cg21598506 |
| 5487 | cg03221504 |
| 5488 | cg02438517 |
| 5489 | cg15288618 |
| 5490 | cg24348581 |
| 5491 | cg12259537 |
| 5492 | cg07202054 |
| 5493 | cg00125585 |
| 5494 | cg15226219 |
| 5495 | cg26541517 |
| 5496 | cg10208416 |
| 5497 | cg12810503 |
| 5498 | cg15205409 |
| 5499 | cg12691330 |
| 5500 | cg26561212 |
| 5501 | cg23155911 |
| 5502 | cg00813378 |
| 5503 | cg02091185 |
| 5504 | cg15716241 |
| 5505 | cg24404823 |
| 5506 | cg08054209 |
| 5507 | cg23987493 |
| 5508 | cg19746897 |
| 5509 | cg13518308 |
| 5510 | cg06766960 |
| 5511 | cg16688675 |
| 5512 | cg07310500 |
| 5513 | cg14436312 |
| 5514 | cg21247398 |
| 5515 | cg13673514 |
| 5516 | cg06397347 |
| 5517 | cg09827761 |
| 5518 | cg04038844 |
| 5519 | cg03440799 |
| 5520 | cg16256243 |
| 5521 | cg19467964 |
| 5522 | cg02693157 |
| 5523 | cg24394172 |
| 5524 | cg20619100 |
| 5525 | cg19118595 |
| 5526 | cg14314770 |
| 5527 | cg09456439 |
| 5528 | cg16419027 |
| 5529 | cg12715395 |
| 5530 | cg12135269 |
| 5531 | cg09696159 |
| 5532 | cg14124465 |
| 5533 | cg10718066 |
| 5534 | cg24864161 |
| 5535 | cg23471274 |
| 5536 | cg08170824 |
| 5537 | cg24509810 |
| 5538 | cg08537688 |
| 5539 | cg27119660 |
| 5540 | cg11309659 |
| 5541 | cg01335210 |
| 5542 | cg27444828 |
| 5543 | cg08765573 |
| 5544 | cg20506745 |
| 5545 | cg14621221 |
| 5546 | cg18616525 |
| 5547 | cg27618369 |
| 5548 | cg00030047 |
| 5549 | cg22790915 |
| 5550 | cg02443306 |
| 5551 | cg06875227 |
| 5552 | cg07688463 |
| 5553 | cg08076717 |
| 5554 | cg23152525 |
| 5555 | cg16708981 |
| 5556 | cg25712005 |
| 5557 | cg02600878 |
| 5558 | cg13664888 |
| 5559 | cg03553278 |
| 5560 | cg07585069 |
| 5561 | cg19797516 |
| 5562 | cg20097520 |
| 5563 | cg25258098 |
| 5564 | cg05740763 |
| 5565 | cg26752773 |
| 5566 | cg00498816 |
| 5567 | cg20141398 |
| 5568 | cg04168853 |
| 5569 | cg09290006 |
| 5570 | cg10029594 |
| 5571 | cg11671913 |
| 5572 | cg23994112 |
| 5573 | cg12961080 |
| 5574 | cg00011740 |
| 5575 | cg04871767 |
| 5576 | cg14179389 |
| 5577 | cg18553223 |
| 5578 | cg21093945 |
| 5579 | cg18131582 |
| 5580 | cg05406088 |
| 5581 | cg02635001 |
| 5582 | cg26049648 |
| 5583 | cg24441810 |
| 5584 | cg17050472 |
| 5585 | cg15928398 |
| 5586 | cg05215063 |
| 5587 | cg11056229 |
| 5588 | cg26053697 |
| 5589 | cg26668551 |
| 5590 | cg13525276 |
| 5591 | cg14742937 |
| 5592 | cg01887834 |
| 5593 | cg24386507 |
| 5594 | cg08786003 |
| 5595 | cg09516476 |
| 5596 | cg09033333 |
| 5597 | cg00169770 |
| 5598 | cg05484710 |
| 5599 | cg12109170 |
| 5600 | cg23877117 |
| 5601 | cg23442853 |
| 5602 | cg25222362 |
| 5603 | cg09137125 |
| 5604 | cg08173915 |
| 5605 | cg06204531 |
| 5606 | cg26995244 |
| 5607 | cg00835279 |
| 5608 | cg17343088 |
| 5609 | cg10621849 |
| 5610 | cg21836586 |
| 5611 | cg24683041 |
| 5612 | cg21850149 |
| 5613 | cg15426035 |
| 5614 | cg11387774 |
| 5615 | cg21287519 |
| 5616 | cg01204210 |
| 5617 | cg13706315 |
| 5618 | cg03380809 |
| 5619 | cg11192416 |
| 5620 | cg19838884 |
| 5621 | cg18591496 |
| 5622 | cg05599160 |
| 5623 | cg02890194 |
| 5624 | cg02219601 |
| 5625 | cg11723741 |
| 5626 | cg24764622 |
| 5627 | cg01622006 |
| 5628 | cg07784872 |
| 5629 | cg17648080 |
| 5630 | cg25880537 |
| 5631 | cg27039625 |
| 5632 | cg00123762 |
| 5633 | cg10617909 |
| 5634 | cg14127013 |
| 5635 | cg16797972 |
| 5636 | cg22703759 |
| 5637 | cg13980930 |
| 5638 | cg14353148 |
| 5639 | cg09495643 |
| 5640 | cg10502118 |
| 5641 | cg10562586 |
| 5642 | cg18665594 |
| 5643 | cg04803214 |
| 5644 | cg24689460 |
| 5645 | cg02665578 |
| 5646 | cg02642943 |
| 5647 | cg20617957 |
| 5648 | cg03714110 |
| 5649 | cg02779913 |
| 5650 | cg08554554 |
| 5651 | cg14200473 |
| 5652 | cg14048158 |
| 5653 | cg19607165 |
| 5654 | cg00255573 |
| 5655 | cg26394775 |
| 5656 | cg07297242 |
| 5657 | cg03451743 |
| 5658 | cg17780731 |
| 5659 | cg18272538 |
| 5660 | cg21586152 |
| 5661 | cg14800014 |
| 5662 | cg18254481 |
| 5663 | cg03781727 |
| 5664 | cg27558693 |
| 5665 | cg16747717 |
| 5666 | cg18732899 |
| 5667 | cg24692310 |
| 5668 | cg17963840 |
| 5669 | cg22367872 |
| 5670 | cg11951019 |
| 5671 | cg04604142 |
| 5672 | cg05337761 |
| 5673 | cg17551002 |
| 5674 | cg19093687 |
| 5675 | cg18840799 |
| 5676 | cg22529990 |
| 5677 | cg10712211 |
| 5678 | cg04744624 |
| 5679 | cg22878489 |
| 5680 | cg25008474 |
| 5681 | cg12060684 |
| 5682 | cg13776248 |
| 5683 | cg06832449 |
| 5684 | cg22436429 |
| 5685 | cg17368297 |
| 5686 | cg11196437 |
| 5687 | cg04856678 |
| 5688 | cg20581874 |
| 5689 | cg01714811 |
| 5690 | cg04265797 |
| 5691 | cg08699206 |
| 5692 | cg16227731 |
| 5693 | cg08285943 |
| 5694 | cg23108931 |
| 5695 | cg00178119 |
| 5696 | cg25608130 |
| 5697 | cg12846139 |
| 5698 | cg05617317 |
| 5699 | cg15581886 |
| 5700 | cg16874812 |
| 5701 | cg17041511 |
| 5702 | cg07532353 |
| 5703 | cg03552873 |
| 5704 | cg03625287 |
| 5705 | cg16071219 |
| 5706 | cg16775542 |
| 5707 | cg27644715 |
| 5708 | cg17933583 |
| 5709 | cg00223315 |
| 5710 | cg00817464 |
| 5711 | cg09990194 |
| 5712 | cg02157552 |
| 5713 | cg12936245 |
| 5714 | cg23757461 |
| 5715 | cg02634968 |
| 5716 | cg12198334 |
| 5717 | cg04667538 |
| 5718 | cg08618362 |
| 5719 | cg02282631 |
| 5720 | cg05086699 |
| 5721 | cg20730770 |
| 5722 | cg14604066 |
| 5723 | cg08829979 |
| 5724 | cg21044139 |
| 5725 | cg14748380 |
| 5726 | cg05000860 |
| 5727 | cg25288140 |
| 5728 | cg18394340 |
| 5729 | cg04614502 |
| 5730 | cg01615861 |
| 5731 | cg09331790 |
| 5732 | cg19333758 |
| 5733 | cg20940424 |
| 5734 | cg13966241 |
| 5735 | cg09561125 |
| 5736 | cg24194661 |
| 5737 | cg14189678 |
| 5738 | cg02069962 |
| 5739 | cg13125627 |
| 5740 | cg15177211 |
| 5741 | cg23071808 |
| 5742 | cg23252259 |
| 5743 | cg18347010 |
| 5744 | cg17132305 |
| 5745 | cg00497023 |
| 5746 | cg20151476 |
| 5747 | cg05462446 |
| 5748 | cg21813377 |
| 5749 | cg26379012 |
| 5750 | cg11909137 |
| 5751 | cg21803088 |
| 5752 | cg19480554 |
| 5753 | cg12253931 |
| 5754 | cg04042468 |
| 5755 | cg07713291 |
| 5756 | cg05228777 |
| 5757 | cg15118447 |
| 5758 | cg20342079 |
| 5759 | cg09455823 |
| 5760 | cg13806741 |
| 5761 | cg03802852 |
| 5762 | cg18184129 |
| 5763 | cg26140637 |
| 5764 | cg16511229 |
| 5765 | cg01510278 |
| 5766 | cg23009254 |
| 5767 | cg13255398 |
| 5768 | cg25322720 |
| 5769 | cg26596712 |
| 5770 | cg17511968 |
| 5771 | cg15112223 |
| 5772 | cg09870910 |
| 5773 | cg03790988 |
| 5774 | cg24092655 |
| 5775 | cg08800613 |
| 5776 | cg09662920 |
| 5777 | cg09580592 |
| 5778 | cg11354603 |
| 5779 | cg06038028 |
| 5780 | cg27285599 |
| 5781 | cg10137601 |
| 5782 | cg22859492 |
| 5783 | cg16271200 |
| 5784 | cg03834411 |
| 5785 | cg16823064 |
| 5786 | cg03020503 |
| 5787 | cg02316596 |
| 5788 | cg10852875 |
| 5789 | cg27427581 |
| 5790 | cg07068761 |
| 5791 | cg22688098 |
| 5792 | cg24750391 |
| 5793 | cg26542283 |
| 5794 | cg07563363 |
| 5795 | cg08815293 |
| 5796 | cg26807616 |
| 5797 | cg18477009 |
| 5798 | cg04377609 |
| 5799 | cg05928239 |
| 5800 | cg06528267 |
| 5801 | cg18327128 |
| 5802 | cg12691004 |
| 5803 | cg26725246 |
| 5804 | cg23886165 |
| 5805 | cg14772935 |
| 5806 | cg04998634 |
| 5807 | cg27575482 |
| 5808 | cg08543028 |
| 5809 | cg06992609 |
| 5810 | cg11637968 |
| 5811 | cg13437682 |
| 5812 | cg09320190 |
| 5813 | cg06301726 |
| 5814 | cg17010273 |
| 5815 | cg08169778 |
| 5816 | cg16924010 |
| 5817 | cg09747182 |
| 5818 | cg09316169 |
| 5819 | cg04093633 |
| 5820 | cg25778857 |
| 5821 | cg12369353 |
| 5822 | cg15852352 |
| 5823 | cg22620627 |
| 5824 | cg17835109 |
| 5825 | cg10748355 |
| 5826 | cg01928646 |
| 5827 | cg25993216 |
| 5828 | cg08630045 |
| 5829 | cg26117265 |
| 5830 | cg23191354 |
| 5831 | cg03552293 |
| 5832 | cg05840264 |
| 5833 | cg08575330 |
| 5834 | cg07199764 |
| 5835 | cg14125904 |
| 5836 | cg03211193 |
| 5837 | cg26659805 |
| 5838 | cg00952516 |
| 5839 | cg11873492 |
| 5840 | cg00897144 |
| 5841 | cg23339720 |
| 5842 | cg26620710 |
| 5843 | cg00231810 |
| 5844 | cg11502375 |
| 5845 | cg26458142 |
| 5846 | cg13016732 |
| 5847 | cg14256110 |
| 5848 | cg09906991 |
| 5849 | cg13495130 |
| 5850 | cg14483291 |
| 5851 | cg02396987 |
| 5852 | cg16482761 |
| 5853 | cg08382669 |
| 5854 | cg21350115 |
| 5855 | cg16008476 |
| 5856 | cg06205476 |
| 5857 | cg27468830 |
| 5858 | cg03086300 |
| 5859 | cg08511957 |
| 5860 | cg24199400 |
| 5861 | cg23604266 |
| 5862 | cg00239353 |
| 5863 | cg10910344 |
| 5864 | cg15572489 |
| 5865 | cg09227556 |
| 5866 | cg08200507 |
| 5867 | cg24736989 |
| 5868 | cg00214140 |
| 5869 | cg10239894 |
| 5870 | cg15237264 |
| 5871 | cg23579508 |
| 5872 | cg21132352 |
| 5873 | cg01462727 |
| 5874 | cg02505534 |
| 5875 | cg06511943 |
| 5876 | cg09332484 |
| 5877 | cg16935980 |
| 5878 | cg06075311 |
| 5879 | cg24246313 |
| 5880 | cg14633398 |
| 5881 | cg03835671 |
| 5882 | cg07876823 |
| 5883 | cg21548155 |
| 5884 | cg09959490 |
| 5885 | cg20402477 |
| 5886 | cg24051473 |
| 5887 | cg18110333 |
| 5888 | cg16017889 |
| 5889 | cg04118741 |
| 5890 | cg22747480 |
| 5891 | cg01933405 |
| 5892 | cg23288059 |
| 5893 | cg02806739 |
| 5894 | cg17757980 |
| 5895 | cg14978830 |
| 5896 | cg10660980 |
| 5897 | cg24054871 |
| 5898 | cg07030884 |
| 5899 | cg04955826 |
| 5900 | cg03877706 |
| 5901 | cg14392677 |
| 5902 | cg10321236 |
| 5903 | cg06238395 |
| 5904 | cg14772079 |
| 5905 | cg19484420 |
| 5906 | cg26067760 |
| 5907 | cg04061696 |
| 5908 | cg15700537 |
| 5909 | cg08572513 |
| 5910 | cg09179845 |
| 5911 | cg22077197 |
| 5912 | cg16676373 |
| 5913 | cg01920850 |
| 5914 | cg02018277 |
| 5915 | cg02036851 |
| 5916 | cg01412419 |
| 5917 | cg02512703 |
| 5918 | cg12997777 |
| 5919 | cg13718960 |
| 5920 | cg03564727 |
| 5921 | cg10653003 |
| 5922 | cg19323241 |
| 5923 | cg22007486 |
| 5924 | cg08551989 |
| 5925 | cg10298187 |
| 5926 | cg01375160 |
| 5927 | cg12804791 |
| 5928 | cg15677087 |
| 5929 | cg08344422 |
| 5930 | cg19798881 |
| 5931 | cg04602284 |
| 5932 | cg14120750 |
| 5933 | cg03885399 |
| 5934 | cg12731799 |
| 5935 | cg10849662 |
| 5936 | cg20181270 |
| 5937 | cg09606807 |
| 5938 | cg22024589 |
| 5939 | cg18353747 |
| 5940 | cg02039404 |
| 5941 | cg04137949 |
| 5942 | cg17475619 |
| 5943 | cg16044871 |
| 5944 | cg03627646 |
| 5945 | cg00777702 |
| 5946 | cg19990651 |
| 5947 | cg18291850 |
| 5948 | cg15733882 |
| 5949 | cg00781473 |
| 5950 | cg21307723 |
| 5951 | cg23069297 |
| 5952 | cg07138944 |
| 5953 | cg11659663 |
| 5954 | cg26938379 |
| 5955 | cg01716288 |
| 5956 | cg00958775 |
| 5957 | cg25845463 |
| 5958 | cg01379456 |
| 5959 | cg20555284 |
| 5960 | cg16968985 |
| 5961 | cg15511801 |
| 5962 | cg13251913 |
| 5963 | cg17666418 |
| 5964 | cg19954357 |
| 5965 | cg17260771 |
| 5966 | cg14072016 |
| 5967 | cg22606684 |
| 5968 | cg26196886 |
| 5969 | cg24216893 |
| 5970 | cg11001844 |
| 5971 | cg14417784 |
| 5972 | cg16195673 |
| 5973 | cg20898829 |
| 5974 | cg16880583 |
| 5975 | cg03074968 |
| 5976 | cg07211259 |
| 5977 | cg25452407 |
| 5978 | cg15935723 |
| 5979 | cg05252264 |
| 5980 | cg03507641 |
| 5981 | cg18674234 |
| 5982 | cg14429056 |
| 5983 | cg15145341 |
| 5984 | cg27484857 |
| 5985 | cg16644418 |
| 5986 | cg01340093 |
| 5987 | cg17139085 |
| 5988 | cg23133460 |
| 5989 | cg09235770 |
| 5990 | cg14992520 |
| 5991 | cg07613115 |
| 5992 | cg19873763 |
| 5993 | cg03012319 |
| 5994 | cg13292942 |
| 5995 | cg15611912 |
| 5996 | cg10264012 |
| 5997 | cg25694915 |
| 5998 | cg02157894 |
| 5999 | cg15174396 |
| 6000 | cg25951288 |
| 6001 | cg15398400 |
| 6002 | cg18504708 |
| 6003 | cg15818671 |
| 6004 | cg10397527 |
| 6005 | cg25716691 |
| 6006 | cg04080963 |
| 6007 | cg14526021 |
| 6008 | cg07808966 |
| 6009 | cg05709657 |
| 6010 | cg13340126 |
| 6011 | cg21498785 |
| 6012 | cg05494467 |
| 6013 | cg09120873 |
| 6014 | cg15134787 |
| 6015 | cg14517946 |
| 6016 | cg03850986 |
| 6017 | cg24367294 |
| 6018 | cg13341082 |
| 6019 | cg09557913 |
| 6020 | cg07011913 |
| 6021 | cg03415545 |
| 6022 | cg23529405 |
| 6023 | cg14844771 |
| 6024 | cg17326104 |
| 6025 | cg03046886 |
| 6026 | cg24803719 |
| 6027 | cg16993462 |
| 6028 | cg20288565 |
| 6029 | cg21232540 |
| 6030 | cg19897071 |
| 6031 | cg00332855 |
| 6032 | cg20988238 |
| 6033 | cg13882284 |
| 6034 | cg06432648 |
| 6035 | cg16574871 |
| 6036 | cg05542646 |
| 6037 | cg09705592 |
| 6038 | cg24136671 |
| 6039 | cg18134292 |
| 6040 | cg26122286 |
| 6041 | cg11603350 |
| 6042 | cg12898512 |
| 6043 | cg27134730 |
| 6044 | cg05687686 |
| 6045 | cg01741372 |
| 6046 | cg01232511 |
| 6047 | cg14299483 |
| 6048 | cg13092315 |
| 6049 | cg17298275 |
| 6050 | cg06638935 |
| 6051 | cg06939851 |
| 6052 | cg02294028 |
| 6053 | cg06643567 |
| 6054 | cg23248910 |
| 6055 | cg26752663 |
| 6056 | cg07811864 |
| 6057 | cg00921344 |
| 6058 | cg02595219 |
| 6059 | cg02845345 |
| 6060 | cg23010118 |
| 6061 | cg24484802 |
| 6062 | cg23279355 |
| 6063 | cg13255083 |
| 6064 | cg19617160 |
| 6065 | cg16744531 |
| 6066 | cg14359501 |
| 6067 | cg03641066 |
| 6068 | cg25093813 |
| 6069 | cg09979763 |
| 6070 | cg19399219 |
| 6071 | cg03669698 |
| 6072 | cg23931049 |
| 6073 | cg23202177 |
| 6074 | cg25518170 |
| 6075 | cg18634334 |
| 6076 | cg07798610 |
| 6077 | cg25720459 |
| 6078 | cg11483169 |
| 6079 | cg20472296 |
| 6080 | cg19528051 |
| 6081 | cg19490001 |
| 6082 | cg08162783 |
| 6083 | cg26591579 |
| 6084 | cg20774026 |
| 6085 | cg07305889 |
| 6086 | cg16131272 |
| 6087 | cg16727201 |
| 6088 | cg09125127 |
| 6089 | cg07088950 |
| 6090 | cg08697117 |
| 6091 | cg13717357 |
| 6092 | cg19734190 |
| 6093 | cg25315420 |
| 6094 | cg18653451 |
| 6095 | cg24637724 |
| 6096 | cg23521666 |
| 6097 | cg21215020 |
| 6098 | cg03241244 |
| 6099 | cg24923379 |
| 6100 | cg22158212 |
| 6101 | cg25574024 |
| 6102 | cg05073325 |
| 6103 | cg09836782 |
| 6104 | cg24530489 |
| 6105 | cg12674212 |
| 6106 | cg20456258 |
| 6107 | cg20184247 |
| 6108 | cg11412466 |
| 6109 | cg02304226 |
| 6110 | cg18791991 |
| 6111 | cg15595755 |
| 6112 | cg04262860 |
| 6113 | cg01260710 |
| 6114 | cg20945232 |
| 6115 | cg03706479 |
| 6116 | cg15913725 |
| 6117 | cg06276663 |
| 6118 | cg20418394 |
| 6119 | cg08105834 |
| 6120 | cg11150585 |
| 6121 | cg18032014 |
| 6122 | cg05692746 |
| 6123 | cg07441712 |
| 6124 | cg00446839 |
| 6125 | cg01926843 |
| 6126 | cg05926640 |
| 6127 | cg01661564 |
| 6128 | cg19002297 |
| 6129 | cg06188545 |
| 6130 | cg05135549 |
| 6131 | cg18031880 |
| 6132 | cg10924277 |
| 6133 | cg02493798 |
| 6134 | cg02450064 |
| 6135 | cg06657721 |
| 6136 | cg22270364 |
| 6137 | cg08237401 |
| 6138 | cg22472290 |
| 6139 | cg05273913 |
| 6140 | cg10946295 |
| 6141 | cg22234663 |
| 6142 | cg06958829 |
| 6143 | cg18193259 |
| 6144 | cg04165845 |
| 6145 | cg11521079 |
| 6146 | cg20724476 |
| 6147 | cg00934609 |
| 6148 | cg04838249 |
| 6149 | cg08418111 |
| 6150 | cg00484421 |
| 6151 | cg18710784 |
| 6152 | cg02366878 |
| 6153 | cg08632164 |
| 6154 | cg08262907 |
| 6155 | cg12671602 |
| 6156 | cg26174004 |
| 6157 | cg06055873 |
| 6158 | cg03565563 |
| 6159 | cg00420566 |
| 6160 | cg24979039 |
| 6161 | cg05765921 |
| 6162 | cg05740045 |
| 6163 | cg08777639 |
| 6164 | cg25281674 |
| 6165 | cg27262794 |
| 6166 | cg05897122 |
| 6167 | cg05374956 |
| 6168 | cg05549818 |
| 6169 | cg10966440 |
| 6170 | cg08820956 |
| 6171 | cg18014207 |
| 6172 | cg04947907 |
| 6173 | cg15552843 |
| 6174 | cg19870512 |
| 6175 | cg22097662 |
| 6176 | cg08836861 |
| 6177 | cg07839926 |
| 6178 | cg13777717 |
| 6179 | cg21933078 |
| 6180 | cg07166171 |
| 6181 | cg22563987 |
| 6182 | cg17976812 |
| 6183 | cg02797195 |
| 6184 | cg12385021 |
| 6185 | cg06635975 |
| 6186 | cg07481398 |
| 6187 | cg10716444 |
| 6188 | cg17931394 |
| 6189 | cg12358061 |
| 6190 | cg17507529 |
| 6191 | cg10742917 |
| 6192 | cg25693597 |
| 6193 | cg25259564 |
| 6194 | cg21603744 |
| 6195 | cg26721651 |
| 6196 | cg11118147 |
| 6197 | cg06768993 |
| 6198 | cg06288788 |
| 6199 | cg10622019 |
| 6200 | cg06873345 |
| 6201 | cg09161656 |
| 6202 | cg07491324 |
| 6203 | cg07102320 |
| 6204 | cg08870893 |
| 6205 | cg14398247 |
| 6206 | cg15769565 |
| 6207 | cg16415457 |
| 6208 | cg15494558 |
| 6209 | cg10588622 |
| 6210 | cg07812827 |
| 6211 | cg22859289 |
| 6212 | cg07451370 |
| 6213 | cg19044242 |
| 6214 | cg01214815 |
| 6215 | cg13541696 |
| 6216 | cg00863893 |
| 6217 | cg23506391 |
| 6218 | cg04315771 |
| 6219 | cg22009488 |
| 6220 | cg12039611 |
| 6221 | cg11500952 |
| 6222 | cg26444116 |
| 6223 | cg11991735 |
| 6224 | cg24440941 |
| 6225 | cg20517911 |
| 6226 | cg18261050 |
| 6227 | cg17496939 |
| 6228 | cg12760508 |
| 6229 | cg24930749 |
| 6230 | cg25092712 |
| 6231 | cg26944755 |
| 6232 | cg00470505 |
| 6233 | cg12769599 |
| 6234 | cg00956549 |
| 6235 | cg05810177 |
| 6236 | cg00424367 |
| 6237 | cg19029904 |
| 6238 | cg17638248 |
| 6239 | cg22732361 |
| 6240 | cg01030624 |
| 6241 | cg06067885 |
| 6242 | cg26561526 |
| 6243 | cg12251779 |
| 6244 | cg13375571 |
| 6245 | cg08010651 |
| 6246 | cg13474332 |
| 6247 | cg25356393 |
| 6248 | cg22488367 |
| 6249 | cg01738022 |
| 6250 | cg17971113 |
| 6251 | cg02566259 |
| 6252 | cg15473017 |
| 6253 | cg08437466 |
| 6254 | cg16885600 |
| 6255 | cg25417675 |
| 6256 | cg09482628 |
| 6257 | cg10777887 |
| 6258 | cg19188855 |
| 6259 | cg17953716 |
| 6260 | cg09973405 |
| 6261 | cg22894896 |
| 6262 | cg11002258 |
| 6263 | cg05764155 |
| 6264 | cg07421015 |
| 6265 | cg25904263 |
| 6266 | cg24460976 |
| 6267 | cg07489413 |
| 6268 | cg06432655 |
| 6269 | cg16008979 |
| 6270 | cg15030167 |
| 6271 | cg09767344 |
| 6272 | cg16254267 |
| 6273 | cg02930419 |
| 6274 | cg05898452 |
| 6275 | cg25241559 |
| 6276 | cg16603087 |
| 6277 | cg23943268 |
| 6278 | cg07329184 |
| 6279 | cg08463186 |
| 6280 | cg16458589 |
| 6281 | cg26002687 |
| 6282 | cg16653138 |
| 6283 | cg06846279 |
| 6284 | cg24011260 |
| 6285 | cg05205314 |
| 6286 | cg23581456 |
| 6287 | cg01035261 |
| 6288 | cg22411549 |
| 6289 | cg23625660 |
| 6290 | cg05351204 |
| 6291 | cg05568492 |
| 6292 | cg13716506 |
| 6293 | cg07718478 |
| 6294 | cg18803104 |
| 6295 | cg00738934 |
| 6296 | cg03097846 |
| 6297 | cg18843739 |
| 6298 | cg09784948 |
| 6299 | cg11598403 |
| 6300 | cg08512365 |
| 6301 | cg18989615 |
| 6302 | cg12971487 |
| 6303 | cg05924540 |
| 6304 | cg25053567 |
| 6305 | cg14682556 |
| 6306 | cg16845623 |
| 6307 | cg06640593 |
| 6308 | cg04362945 |
| 6309 | cg03649288 |
| 6310 | cg19391247 |
| 6311 | cg21649089 |
| 6312 | cg01914652 |
| 6313 | cg22987116 |
| 6314 | cg12696398 |
| 6315 | cg27350193 |
| 6316 | cg03557384 |
| 6317 | cg08847431 |
| 6318 | cg03964111 |
| 6319 | cg23303369 |
| 6320 | cg00667751 |
| 6321 | cg12778716 |
| 6322 | cg05588228 |
| 6323 | cg21228500 |
| 6324 | cg06230615 |
| 6325 | cg15493618 |
| 6326 | cg00809164 |
| 6327 | cg19512473 |
| 6328 | cg20583679 |
| 6329 | cg16998223 |
| 6330 | cg08310581 |
| 6331 | cg25735051 |
| 6332 | cg16663891 |
| 6333 | cg18794321 |
| 6334 | cg20077343 |
| 6335 | cg26116950 |
| 6336 | cg02525423 |
| 6337 | cg00449000 |
| 6338 | cg14148690 |
| 6339 | cg16585167 |
| 6340 | cg13422047 |
| 6341 | cg10381249 |
| 6342 | cg02466788 |
| 6343 | cg15268101 |
| 6344 | cg05651265 |
| 6345 | cg19391456 |
| 6346 | cg02604758 |
| 6347 | cg26560969 |
| 6348 | cg15096563 |
| 6349 | cg22095263 |
| 6350 | cg25708328 |
| 6351 | cg05485060 |
| 6352 | cg16448636 |
| 6353 | cg02008416 |
| 6354 | cg01464849 |
| 6355 | cg06611524 |
| 6356 | cg22800743 |
| 6357 | cg18409999 |
| 6358 | cg01951916 |
| 6359 | cg12511580 |
| 6360 | cg16483840 |
| 6361 | cg11537355 |
| 6362 | cg03710412 |
| 6363 | cg17023203 |
| 6364 | cg13128937 |
| 6365 | cg09136052 |
| 6366 | cg16135989 |
| 6367 | cg00767581 |
| 6368 | cg27324541 |
| 6369 | cg13667389 |
| 6370 | cg17816357 |
| 6371 | cg05790630 |
| 6372 | cg11497410 |
| 6373 | cg16937902 |
| 6374 | cg12509665 |
| 6375 | cg27066925 |
| 6376 | cg08479476 |
| 6377 | cg05906443 |
| 6378 | cg17888985 |
| 6379 | cg09344348 |
| 6380 | cg27450668 |
| 6381 | cg25677261 |
| 6382 | cg03377939 |
| 6383 | cg19314470 |
| 6384 | cg02305687 |
| 6385 | cg16568809 |
| 6386 | cg21916566 |
| 6387 | cg09141266 |
| 6388 | cg25871008 |
| 6389 | cg06310633 |
| 6390 | cg24908198 |
| 6391 | cg25839195 |
| 6392 | cg03126946 |
| 6393 | cg17274057 |
| 6394 | cg11363527 |
| 6395 | cg21687740 |
| 6396 | cg23106378 |
| 6397 | cg24694665 |
| 6398 | cg18786280 |
| 6399 | cg14269708 |
| 6400 | cg20543544 |
| 6401 | cg10781582 |
| 6402 | cg11806439 |
| 6403 | cg15984300 |
| 6404 | cg27342781 |
| 6405 | cg26780125 |
| 6406 | cg24902111 |
| 6407 | cg08211704 |
| 6408 | cg11229273 |
| 6409 | cg15258711 |
| 6410 | cg16389901 |
| 6411 | cg25224232 |
| 6412 | cg07970422 |
| 6413 | cg06570075 |
| 6414 | cg07339783 |
| 6415 | cg16402714 |
| 6416 | cg26151206 |
| 6417 | cg11382082 |
| 6418 | cg24965248 |
| 6419 | cg06902607 |
| 6420 | cg13577556 |
| 6421 | cg15333426 |
| 6422 | cg18697632 |
| 6423 | cg25841279 |
| 6424 | cg00397739 |
| 6425 | cg01090659 |
| 6426 | cg15559674 |
| 6427 | cg11449128 |
| 6428 | cg23535784 |
| 6429 | cg18206481 |
| 6430 | cg11391335 |
| 6431 | cg00348467 |
| 6432 | cg24255988 |
| 6433 | cg10546817 |
| 6434 | cg06587623 |
| 6435 | cg25000021 |
| 6436 | cg09223687 |
| 6437 | cg11011512 |
| 6438 | cg05824261 |
| 6439 | cg03234732 |
| 6440 | cg08892491 |
| 6441 | cg07499673 |
| 6442 | cg27483694 |
| 6443 | cg05783290 |
| 6444 | cg23128495 |
| 6445 | cg00914028 |
| 6446 | cg12230728 |
| 6447 | cg00724936 |
| 6448 | cg02187406 |
| 6449 | cg01549576 |
| 6450 | cg21592059 |
| 6451 | cg06721393 |
| 6452 | cg19871235 |
| 6453 | cg21528204 |
| 6454 | cg17849733 |
| 6455 | cg14497346 |
| 6456 | cg11168293 |
| 6457 | cg05927190 |
| 6458 | cg15054508 |
| 6459 | cg24929925 |
| 6460 | cg00498796 |
| 6461 | cg07008943 |
| 6462 | cg23389408 |
| 6463 | cg23226914 |
| 6464 | cg02557335 |
| 6465 | cg05473257 |
| 6466 | cg11319592 |
| 6467 | cg07416237 |
| 6468 | cg02607723 |
| 6469 | cg06213468 |
| 6470 | cg03111560 |
| 6471 | cg13862711 |
| 6472 | cg26661922 |
| 6473 | cg10045024 |
| 6474 | cg20171396 |
| 6475 | cg01558931 |
| 6476 | cg12680929 |
| 6477 | cg16236157 |
| 6478 | cg00508023 |
| 6479 | cg22352078 |
| 6480 | cg27048095 |
| 6481 | cg09653626 |
| 6482 | cg07388969 |
| 6483 | cg03670452 |
| 6484 | cg12223498 |
| 6485 | cg06152865 |
| 6486 | cg27659440 |
| 6487 | cg07572451 |
| 6488 | cg23760687 |
| 6489 | cg19846314 |
| 6490 | cg24553170 |
| 6491 | cg08436419 |
| 6492 | cg08006672 |
| 6493 | cg12355360 |
| 6494 | cg02188185 |
| 6495 | cg12323168 |
| 6496 | cg07795766 |
| 6497 | cg07190553 |
| 6498 | cg10049789 |
| 6499 | cg13973659 |
| 6500 | cg17466278 |
| 6501 | cg01157024 |
| 6502 | cg05610000 |
| 6503 | cg00149213 |
| 6504 | cg19893585 |
| 6505 | cg04679781 |
| 6506 | cg26464082 |
| 6507 | cg27299033 |
| 6508 | cg17109053 |
| 6509 | cg04669853 |
| 6510 | cg01932691 |
| 6511 | cg00931874 |
| 6512 | cg14780297 |
| 6513 | cg01237056 |
| 6514 | cg06607997 |
| 6515 | cg21982950 |
| 6516 | cg03652298 |
| 6517 | cg25504717 |
| 6518 | cg13773631 |
| 6519 | cg00149716 |
| 6520 | cg03958979 |
| 6521 | cg23230830 |
| 6522 | cg00085493 |
| 6523 | cg00843506 |
| 6524 | cg23510258 |
| 6525 | cg11284147 |
| 6526 | cg03388789 |
| 6527 | cg09066883 |
| 6528 | cg09828411 |
| 6529 | cg09782560 |
| 6530 | cg24038545 |
| 6531 | cg12342675 |
| 6532 | cg20387429 |
| 6533 | cg07133197 |
| 6534 | cg03826704 |
| 6535 | cg10905391 |
| 6536 | cg26942432 |
| 6537 | cg19139509 |
| 6538 | cg20443254 |
| 6539 | cg10598353 |
| 6540 | cg09857555 |
| 6541 | cg10699397 |
| 6542 | cg09577651 |
| 6543 | cg22987766 |
| 6544 | cg26474549 |
| 6545 | cg18692678 |
| 6546 | cg16808912 |
| 6547 | cg25066845 |
| 6548 | cg25316749 |
| 6549 | cg07345999 |
| 6550 | cg19387132 |
| 6551 | cg15787128 |
| 6552 | cg15492857 |
| 6553 | cg06021088 |
| 6554 | cg19723436 |
| 6555 | cg27313589 |
| 6556 | cg00074365 |
| 6557 | cg06500727 |
| 6558 | cg07690222 |
| 6559 | cg26446133 |
| 6560 | cg17403699 |
| 6561 | cg06635722 |
| 6562 | cg17031543 |
| 6563 | cg12751521 |
| 6564 | cg03975755 |
| 6565 | cg04962621 |
| 6566 | cg26573786 |
| 6567 | cg06302690 |
| 6568 | cg23238440 |
| 6569 | cg08055746 |
| 6570 | cg09010107 |
| 6571 | cg03335246 |
| 6572 | cg07751528 |
| 6573 | cg17218270 |
| 6574 | cg07106394 |
| 6575 | cg04821375 |
| 6576 | cg08038696 |
| 6577 | cg02722637 |
| 6578 | cg06779449 |
| 6579 | cg13231954 |
| 6580 | cg09854615 |
| 6581 | cg23473694 |
| 6582 | cg27142059 |
| 6583 | cg03646740 |
| 6584 | cg18267049 |
| 6585 | cg02735333 |
| 6586 | cg09249152 |
| 6587 | cg20675009 |
| 6588 | cg16470716 |
| 6589 | cg19263868 |
| 6590 | cg26340916 |
| 6591 | cg01085576 |
| 6592 | cg24527252 |
| 6593 | cg08999742 |
| 6594 | cg23612849 |
| 6595 | cg04922803 |
| 6596 | cg11312740 |
| 6597 | cg14453201 |
| 6598 | cg04477293 |
| 6599 | cg03209871 |
| 6600 | cg03733219 |
| 6601 | cg12948059 |
| 6602 | cg10272461 |
| 6603 | cg04716147 |
| 6604 | cg24428372 |
| 6605 | cg00073460 |
| 6606 | cg11479503 |
| 6607 | cg27183726 |
| 6608 | cg20911165 |
| 6609 | cg16498409 |
| 6610 | cg00426822 |
| 6611 | cg18553979 |
| 6612 | cg09776463 |
| 6613 | cg06935531 |
| 6614 | cg14479798 |
| 6615 | cg13341240 |
| 6616 | cg10104252 |
| 6617 | cg02637643 |
| 6618 | cg26379759 |
| 6619 | cg19013695 |
| 6620 | cg10165543 |
| 6621 | cg05363903 |
| 6622 | cg00093057 |
| 6623 | cg10068478 |
| 6624 | cg26276327 |
| 6625 | cg24373369 |
| 6626 | cg17171407 |
| 6627 | cg18990042 |
| 6628 | cg26664583 |
| 6629 | cg10201685 |
| 6630 | cg08641084 |
| 6631 | cg00299943 |
| 6632 | cg10920646 |
| 6633 | cg22826333 |
| 6634 | cg11617974 |
| 6635 | cg26225794 |
| 6636 | cg05706898 |
| 6637 | cg21261158 |
| 6638 | cg03337057 |
| 6639 | cg14972362 |
| 6640 | cg25938039 |
| 6641 | cg12083476 |
| 6642 | cg17000480 |
| 6643 | cg00599530 |
| 6644 | cg20070323 |
| 6645 | cg17968795 |
| 6646 | cg11253122 |
| 6647 | cg08360726 |
| 6648 | cg22397254 |
| 6649 | cg06495631 |
| 6650 | cg23676369 |
| 6651 | cg03352600 |
| 6652 | cg06850491 |
| 6653 | cg27529515 |
| 6654 | cg17809353 |
| 6655 | cg19728345 |
| 6656 | cg18939596 |
| 6657 | cg00084338 |
| 6658 | cg02484490 |
| 6659 | cg03556068 |
| 6660 | cg06482830 |
| 6661 | cg26779905 |
| 6662 | cg15733165 |
| 6663 | cg16431531 |
| 6664 | cg14866032 |
| 6665 | cg24015747 |
| 6666 | cg17959970 |
| 6667 | cg12788878 |
| 6668 | cg13605024 |
| 6669 | cg07021052 |
| 6670 | cg24623243 |
| 6671 | cg01461856 |
| 6672 | cg04566826 |
| 6673 | cg04948906 |
| 6674 | cg21413492 |
| 6675 | cg26327666 |
| 6676 | cg04337594 |
| 6677 | cg02072400 |
| 6678 | cg22050426 |
| 6679 | cg01562537 |
| 6680 | cg04456238 |
| 6681 | cg16408865 |
| 6682 | cg06558483 |
| 6683 | cg09148948 |
| 6684 | cg10655572 |
| 6685 | cg22580054 |
| 6686 | cg21123833 |
| 6687 | cg26736200 |
| 6688 | cg06585766 |
| 6689 | cg14443519 |
| 6690 | cg03293788 |
| 6691 | cg03104083 |
| 6692 | cg27462519 |
| 6693 | cg17744293 |
| 6694 | cg25946646 |
| 6695 | cg01610165 |
| 6696 | cg10129493 |
| 6697 | cg04500819 |
| 6698 | cg05001848 |
| 6699 | cg09687907 |
| 6700 | cg17780246 |
| 6701 | cg23616139 |
| 6702 | cg21580687 |
| 6703 | cg04805166 |
| 6704 | cg25597390 |
| 6705 | cg20167959 |
| 6706 | cg01409832 |
| 6707 | cg09029791 |
| 6708 | cg08259514 |
| 6709 | cg21006791 |
| 6710 | cg18654812 |
| 6711 | cg19634902 |
| 6712 | cg26308909 |
| 6713 | cg16720809 |
| 6714 | cg23098661 |
| 6715 | cg22122068 |
| 6716 | cg09981233 |
| 6717 | cg13885155 |
| 6718 | cg21016266 |
| 6719 | cg10907249 |
| 6720 | cg12306161 |
| 6721 | cg06411879 |
| 6722 | cg07790279 |
| 6723 | cg09466887 |
| 6724 | cg25027788 |
| 6725 | cg05349667 |
| 6726 | cg02490736 |
| 6727 | cg16050897 |
| 6728 | cg08341874 |
| 6729 | cg21052932 |
| 6730 | cg08536446 |
| 6731 | cg14879165 |
| 6732 | cg26502666 |
| 6733 | cg15544177 |
| 6734 | cg21005607 |
| 6735 | cg07281747 |
| 6736 | cg12326725 |
| 6737 | cg09850478 |
| 6738 | cg13254852 |
| 6739 | cg08153172 |
| 6740 | cg21704843 |
| 6741 | cg05460318 |
| 6742 | cg16696400 |
| 6743 | cg03465499 |
| 6744 | cg01959123 |
| 6745 | cg03865101 |
| 6746 | cg07225966 |
| 6747 | cg24711888 |
| 6748 | cg15886873 |
| 6749 | cg06341027 |
| 6750 | cg07745671 |
| 6751 | cg05136737 |
| 6752 | cg13032272 |
| 6753 | cg27377632 |
| 6754 | cg21699381 |
| 6755 | cg16396933 |
| 6756 | cg26111295 |
| 6757 | cg21430685 |
| 6758 | cg05163491 |
| 6759 | cg07028396 |
| 6760 | cg06085985 |
| 6761 | cg13998092 |
| 6762 | cg15050051 |
| 6763 | cg23991388 |
| 6764 | cg19654061 |
| 6765 | cg01855070 |
| 6766 | cg07770222 |
| 6767 | cg12324144 |
| 6768 | cg04305469 |
| 6769 | cg26640901 |
| 6770 | cg17507671 |
| 6771 | cg14082127 |
| 6772 | cg07698196 |
| 6773 | cg04557383 |
| 6774 | cg07971156 |
| 6775 | cg00346205 |
| 6776 | cg25308173 |
| 6777 | cg04173864 |
| 6778 | cg02005600 |
| 6779 | cg22879476 |
| 6780 | cg22052566 |
| 6781 | cg24303858 |
| 6782 | cg03600259 |
| 6783 | cg27009374 |
| 6784 | cg17118836 |
| 6785 | cg22053945 |
| 6786 | cg02095589 |
| 6787 | cg27507261 |
| 6788 | cg13345558 |
| 6789 | cg22860375 |
| 6790 | cg06660395 |
| 6791 | cg14205321 |
| 6792 | cg08775428 |
| 6793 | cg07782794 |
| 6794 | cg00245150 |
| 6795 | cg17131553 |
| 6796 | cg05914697 |
| 6797 | cg07618409 |
| 6798 | cg13234863 |
| 6799 | cg10375078 |
| 6800 | cg10574365 |
| 6801 | cg07930539 |
| 6802 | cg15190451 |
| 6803 | cg00450560 |
| 6804 | cg20920097 |
| 6805 | cg16622958 |
| 6806 | cg05686633 |
| 6807 | cg17478979 |
| 6808 | cg15291685 |
| 6809 | cg23802491 |
| 6810 | cg23370725 |
| 6811 | cg24867279 |
| 6812 | cg27556492 |
| 6813 | cg09350880 |
| 6814 | cg09852221 |
| 6815 | cg02722204 |
| 6816 | cg00531453 |
| 6817 | cg04895345 |
| 6818 | cg24164933 |
| 6819 | cg07533511 |
| 6820 | cg14606431 |
| 6821 | cg23957595 |
| 6822 | cg13757506 |
| 6823 | cg13447080 |
| 6824 | cg26957187 |
| 6825 | cg16775752 |
| 6826 | cg23415203 |
| 6827 | cg05250932 |
| 6828 | cg09168797 |
| 6829 | cg15810184 |
| 6830 | cg27044931 |
| 6831 | cg26209990 |
| 6832 | cg14425687 |
| 6833 | cg17402060 |
| 6834 | cg27272235 |
| 6835 | cg22913231 |
| 6836 | cg27031099 |
| 6837 | cg01554606 |
| 6838 | cg13465470 |
| 6839 | cg10791035 |
| 6840 | cg24810550 |
| 6841 | cg26955482 |
| 6842 | cg11070274 |
| 6843 | cg24537772 |
| 6844 | cg25292745 |
| 6845 | cg04464316 |
| 6846 | cg24426691 |
| 6847 | cg14432910 |
| 6848 | cg17215468 |
| 6849 | cg25908778 |
| 6850 | cg03871124 |
| 6851 | cg14032261 |
| 6852 | cg18144593 |
| 6853 | cg03990139 |
| 6854 | cg11724511 |
| 6855 | cg22969524 |
| 6856 | cg05374271 |
| 6857 | cg10529891 |
| 6858 | cg25264948 |
| 6859 | cg18203961 |
| 6860 | cg01371660 |
| 6861 | cg13273037 |
| 6862 | cg02758612 |
| 6863 | cg19515857 |
| 6864 | cg11641787 |
| 6865 | cg13871708 |
| 6866 | cg24247132 |
| 6867 | cg17977679 |
| 6868 | cg13309747 |
| 6869 | cg16776065 |
| 6870 | cg26486972 |
| 6871 | cg20170271 |
| 6872 | cg18197392 |
| 6873 | cg01538731 |
| 6874 | cg25720371 |
| 6875 | cg14827832 |
| 6876 | cg00677697 |
| 6877 | cg18206764 |
| 6878 | cg05320671 |
| 6879 | cg21937462 |
| 6880 | cg21542643 |
| 6881 | cg00734931 |
| 6882 | cg11225074 |
| 6883 | cg12790145 |
| 6884 | cg23535171 |
| 6885 | cg22254413 |
| 6886 | cg11878113 |
| 6887 | cg25460753 |
| 6888 | cg26169991 |
| 6889 | cg23082229 |
| 6890 | cg25909396 |
| 6891 | cg26004810 |
| 6892 | cg18366169 |
| 6893 | cg10599156 |
| 6894 | cg09505426 |
| 6895 | cg20640499 |
| 6896 | cg11125332 |
| 6897 | cg06731639 |
| 6898 | cg04876069 |
| 6899 | cg20627652 |
| 6900 | cg00871381 |
| 6901 | cg21057507 |
| 6902 | cg01830463 |
| 6903 | cg17226349 |
| 6904 | cg22920655 |
| 6905 | cg27046573 |
| 6906 | cg14300707 |
| 6907 | cg07954863 |
| 6908 | cg11017106 |
| 6909 | cg04776727 |
| 6910 | cg06278337 |
| 6911 | cg00757404 |
| 6912 | cg16023943 |
| 6913 | cg05687091 |
| 6914 | cg15204734 |
| 6915 | cg19236493 |
| 6916 | cg26868800 |
| 6917 | cg16617723 |
| 6918 | cg04036593 |
| 6919 | cg07126115 |
| 6920 | cg04913265 |
| 6921 | cg25485560 |
| 6922 | cg07804408 |
| 6923 | cg22896107 |
| 6924 | cg08860954 |
| 6925 | cg06430772 |
| 6926 | cg19423978 |
| 6927 | cg12505170 |
| 6928 | cg26986681 |
| 6929 | cg05558615 |
| 6930 | cg02721000 |
| 6931 | cg14909201 |
| 6932 | cg27270264 |
| 6933 | cg10461784 |
| 6934 | cg15752514 |
| 6935 | cg12492885 |
| 6936 | cg19438746 |
| 6937 | cg27444751 |
| 6938 | cg14939821 |
| 6939 | cg13568432 |
| 6940 | cg08253164 |
| 6941 | cg17612001 |
| 6942 | cg08633584 |
| 6943 | cg23725149 |
| 6944 | cg09978441 |
| 6945 | cg21567756 |
| 6946 | cg15367247 |
| 6947 | cg10429608 |
| 6948 | cg17753365 |
| 6949 | cg26766064 |
| 6950 | cg19597498 |
| 6951 | cg13485290 |
| 6952 | cg07107105 |
| 6953 | cg20804418 |
| 6954 | cg07751331 |
| 6955 | cg01330408 |
| 6956 | cg25422780 |
| 6957 | cg14885748 |
| 6958 | cg11117637 |
| 6959 | cg01881308 |
| 6960 | cg22758340 |
| 6961 | cg25063515 |
| 6962 | cg08329137 |
| 6963 | cg16246488 |
| 6964 | cg17862993 |
| 6965 | cg06264984 |
| 6966 | cg26218577 |
| 6967 | cg04748223 |
| 6968 | cg10156846 |
| 6969 | cg17881200 |
| 6970 | cg11597131 |
| 6971 | cg24033122 |
| 6972 | cg24940576 |
| 6973 | cg00657094 |
| 6974 | cg16902945 |
| 6975 | cg14222093 |
| 6976 | cg21678211 |
| 6977 | cg00834581 |
| 6978 | cg01300914 |
| 6979 | cg13982454 |
| 6980 | cg19693071 |
| 6981 | cg17504591 |
| 6982 | cg05903736 |
| 6983 | cg22601285 |
| 6984 | cg23404248 |
| 6985 | cg12314682 |
| 6986 | cg24086675 |
| 6987 | cg03127558 |
| 6988 | cg21608953 |
| 6989 | cg16077929 |
| 6990 | cg14834554 |
| 6991 | cg09845489 |
| 6992 | cg13939978 |
| 6993 | cg08005120 |
| 6994 | cg03287574 |
| 6995 | cg20061654 |
| 6996 | cg23567627 |
| 6997 | cg16725974 |
| 6998 | cg06766732 |
| 6999 | cg08994060 |
| 7000 | cg01481989 |
| 7001 | cg16411361 |
| 7002 | cg19121352 |
| 7003 | cg17690919 |
| 7004 | cg11685510 |
| 7005 | cg11809342 |
| 7006 | cg19509393 |
| 7007 | cg01765416 |
| 7008 | cg09836827 |
| 7009 | cg02464901 |
| 7010 | cg17578639 |
| 7011 | cg04560029 |
| 7012 | cg07312357 |
| 7013 | cg26472118 |
| 7014 | cg11008003 |
| 7015 | cg14431361 |
| 7016 | cg27160726 |
| 7017 | cg06664095 |
| 7018 | cg00689225 |
| 7019 | cg13899142 |
| 7020 | cg26648031 |
| 7021 | cg26991112 |
| 7022 | cg12455762 |
| 7023 | cg21354353 |
| 7024 | cg13415928 |
| 7025 | cg26704293 |
| 7026 | cg16552822 |
| 7027 | cg07180212 |
| 7028 | cg06614044 |
| 7029 | cg12884532 |
| 7030 | cg00871371 |
| 7031 | cg17654660 |
| 7032 | cg26851153 |
| 7033 | cg18428265 |
| 7034 | cg27593341 |
| 7035 | cg00284129 |
| 7036 | cg17143179 |
| 7037 | cg07962783 |
| 7038 | cg03883256 |
| 7039 | cg24009731 |
| 7040 | cg04421582 |
| 7041 | cg06246435 |
| 7042 | cg14921884 |
| 7043 | cg00636427 |
| 7044 | cg24792289 |
| 7045 | cg19899806 |
| 7046 | cg15070011 |
| 7047 | cg10152131 |
| 7048 | cg04515200 |
| 7049 | cg09108394 |
| 7050 | cg24633253 |
| 7051 | cg20301127 |
| 7052 | cg24364737 |
| 7053 | cg04888708 |
| 7054 | cg25987744 |
| 7055 | cg03585778 |
| 7056 | cg05349016 |
| 7057 | cg00289503 |
| 7058 | cg02822788 |
| 7059 | cg26550194 |
| 7060 | cg17661717 |
| 7061 | cg05797501 |
| 7062 | cg04116385 |
| 7063 | cg10381888 |
| 7064 | cg14768206 |
| 7065 | cg15567360 |
| 7066 | cg08668199 |
| 7067 | cg04058516 |
| 7068 | cg25568114 |
| 7069 | cg02470808 |
| 7070 | cg15105326 |
| 7071 | cg00804096 |
| 7072 | cg20544605 |
| 7073 | cg00398420 |
| 7074 | cg03723629 |
| 7075 | cg14363604 |
| 7076 | cg00208504 |
| 7077 | cg27254482 |
| 7078 | cg08533783 |
| 7079 | cg09211763 |
| 7080 | cg08839808 |
| 7081 | cg00301120 |
| 7082 | cg03531754 |
| 7083 | cg11530334 |
| 7084 | cg00264136 |
| 7085 | cg15505276 |
| 7086 | cg05820087 |
| 7087 | cg17860198 |
| 7088 | cg08918879 |
| 7089 | cg05895618 |
| 7090 | cg25164649 |
| 7091 | cg09072630 |
| 7092 | cg01081346 |
| 7093 | cg04765848 |
| 7094 | cg06578825 |
| 7095 | cg25823085 |
| 7096 | cg21883059 |
| 7097 | cg12869334 |
| 7098 | cg01469212 |
| 7099 | cg26203582 |
| 7100 | cg10133876 |
| 7101 | cg07104417 |
| 7102 | cg01549428 |
| 7103 | cg25959149 |
| 7104 | cg05075921 |
| 7105 | cg17405117 |
| 7106 | cg17922359 |
| 7107 | cg09057517 |
| 7108 | cg05313542 |
| 7109 | cg22510337 |
| 7110 | cg17267532 |
| 7111 | cg15341132 |
| 7112 | cg25612698 |
| 7113 | cg08447324 |
| 7114 | cg24363020 |
| 7115 | cg24506320 |
| 7116 | cg12510708 |
| 7117 | cg06366587 |
| 7118 | cg05539265 |
| 7119 | cg13710662 |
| 7120 | cg10077239 |
| 7121 | cg03001305 |
| 7122 | cg01349792 |
| 7123 | cg26816907 |
| 7124 | cg21644740 |
| 7125 | cg18947360 |
| 7126 | cg22645045 |
| 7127 | cg22702205 |
| 7128 | cg17485870 |
| 7129 | cg20709110 |
| 7130 | cg18128536 |
| 7131 | cg18190798 |
| 7132 | cg03192897 |
| 7133 | cg02836478 |
| 7134 | cg14127046 |
| 7135 | cg16719409 |
| 7136 | cg21298174 |
| 7137 | cg18342026 |
| 7138 | cg17372327 |
| 7139 | cg06431877 |
| 7140 | cg02571055 |
| 7141 | cg06644428 |
| 7142 | cg25941963 |
| 7143 | cg03695871 |
| 7144 | cg25101863 |
| 7145 | cg24724664 |
| 7146 | cg26967792 |
| 7147 | cg22141235 |
| 7148 | cg15876676 |
| 7149 | cg27546969 |
| 7150 | cg22365178 |
| 7151 | cg15007963 |
| 7152 | cg10202461 |
| 7153 | cg19228034 |
| 7154 | cg17984293 |
| 7155 | cg14726637 |
| 7156 | cg11427534 |
| 7157 | cg11476866 |
| 7158 | cg20690125 |
| 7159 | cg01743841 |
| 7160 | cg09377696 |
| 7161 | cg20857738 |
| 7162 | cg01213920 |
| 7163 | cg26427044 |
| 7164 | cg18048071 |
| 7165 | cg25527090 |
| 7166 | cg07807210 |
| 7167 | cg11825059 |
| 7168 | cg11139878 |
| 7169 | cg01434160 |
| 7170 | cg24722777 |
| 7171 | cg06850787 |
| 7172 | cg22524864 |
| 7173 | cg19471574 |
| 7174 | cg00004667 |
| 7175 | cg02201490 |
| 7176 | cg17445666 |
| 7177 | cg13547672 |
| 7178 | cg05905176 |
| 7179 | cg22277749 |
| 7180 | cg05063999 |
| 7181 | cg04896126 |
| 7182 | cg07442408 |
| 7183 | cg11914795 |
| 7184 | cg09505809 |
| 7185 | cg23602092 |
| 7186 | cg07617394 |
| 7187 | cg03791955 |
| 7188 | cg05025046 |
| 7189 | cg14560110 |
| 7190 | cg23632875 |
| 7191 | cg10134799 |
| 7192 | cg12281151 |
| 7193 | cg01184930 |
| 7194 | cg00879791 |
| 7195 | cg05355757 |
| 7196 | cg21862992 |
| 7197 | cg18304342 |
| 7198 | cg11101345 |
| 7199 | cg21538684 |
| 7200 | cg02186140 |
| 7201 | cg22843712 |
| 7202 | cg09797356 |
| 7203 | cg16718276 |
| 7204 | cg10766876 |
| 7205 | cg06506352 |
| 7206 | cg18291703 |
| 7207 | cg15710198 |
| 7208 | cg16382969 |
| 7209 | cg12262378 |
| 7210 | cg23737190 |
| 7211 | cg21576525 |
| 7212 | cg10978355 |
| 7213 | cg01524697 |
| 7214 | cg01933515 |
| 7215 | cg08548559 |
| 7216 | cg04138706 |
| 7217 | cg07175536 |
| 7218 | cg03080336 |
| 7219 | cg18973024 |
| 7220 | cg00189137 |
| 7221 | cg03490228 |
| 7222 | cg04821520 |
| 7223 | cg27359566 |
| 7224 | cg01002857 |
| 7225 | cg13211181 |
| 7226 | cg16512885 |
| 7227 | cg19159365 |
| 7228 | cg08908244 |
| 7229 | cg03701930 |
| 7230 | cg05882929 |
| 7231 | cg02779288 |
| 7232 | cg08263647 |
| 7233 | cg23262274 |
| 7234 | cg20879720 |
| 7235 | cg08656816 |
| 7236 | cg15133098 |
| 7237 | cg02304233 |
| 7238 | cg22261694 |
| 7239 | cg27405791 |
| 7240 | cg01759870 |
| 7241 | cg13251533 |
| 7242 | cg05148335 |
| 7243 | cg07604401 |
| 7244 | cg06675877 |
| 7245 | cg04569855 |
| 7246 | cg19943781 |
| 7247 | cg24516901 |
| 7248 | cg17837528 |
| 7249 | cg04397987 |
| 7250 | cg19442201 |
| 7251 | cg02661079 |
| 7252 | cg17372657 |
| 7253 | cg05360958 |
| 7254 | cg12157673 |
| 7255 | cg25826463 |
| 7256 | cg05961809 |
| 7257 | cg02200107 |
| 7258 | cg25712127 |
| 7259 | cg25986774 |
| 7260 | cg14136535 |
| 7261 | cg24437715 |
| 7262 | cg13426490 |
| 7263 | cg01745789 |
| 7264 | cg12020853 |
| 7265 | cg18416936 |
| 7266 | cg18972013 |
| 7267 | cg00160981 |
| 7268 | cg06710672 |
| 7269 | cg19505136 |
| 7270 | cg05340269 |
| 7271 | cg27219182 |
| 7272 | cg22995692 |
| 7273 | cg21133153 |
| 7274 | cg06061081 |
| 7275 | cg18038544 |
| 7276 | cg06647693 |
| 7277 | cg17196051 |
| 7278 | cg04839131 |
| 7279 | cg21411812 |
| 7280 | cg08891882 |
| 7281 | cg10812338 |
| 7282 | cg15977594 |
| 7283 | cg17744933 |
| 7284 | cg03469869 |
| 7285 | cg08535604 |
| 7286 | cg05757448 |
| 7287 | cg22183049 |
| 7288 | cg17069396 |
| 7289 | cg19714985 |
| 7290 | cg21248554 |
| 7291 | cg14783703 |
| 7292 | cg23166857 |
| 7293 | cg24598141 |
| 7294 | cg22537334 |
| 7295 | cg04894218 |
| 7296 | cg01033356 |
| 7297 | cg24102749 |
| 7298 | cg07952254 |
| 7299 | cg24607575 |
| 7300 | cg23008606 |
| 7301 | cg11990630 |
| 7302 | cg01856692 |
| 7303 | cg04943129 |
| 7304 | cg10791406 |
| 7305 | cg05633523 |
| 7306 | cg03263673 |
| 7307 | cg24446178 |
| 7308 | cg03315557 |
| 7309 | cg16409409 |
| 7310 | cg22378427 |
| 7311 | cg22681709 |
| 7312 | cg02670718 |
| 7313 | cg17579232 |
| 7314 | cg02023167 |
| 7315 | cg09645574 |
| 7316 | cg14729962 |
| 7317 | cg11558570 |
| 7318 | cg11643132 |
| 7319 | cg07064544 |
| 7320 | cg11840740 |
| 7321 | cg15921351 |
| 7322 | cg06593940 |
| 7323 | cg00695781 |
| 7324 | cg27128311 |
| 7325 | cg05795002 |
| 7326 | cg18341098 |
| 7327 | cg17327423 |
| 7328 | cg05908492 |
| 7329 | cg26882164 |
| 7330 | cg21042242 |
| 7331 | cg24042923 |
| 7332 | cg11706624 |
| 7333 | cg12679760 |
| 7334 | cg17478810 |
| 7335 | cg06137588 |
| 7336 | cg10972873 |
| 7337 | cg02769145 |
| 7338 | cg11468953 |
| 7339 | cg24831125 |
| 7340 | cg17727125 |
| 7341 | cg12212591 |
| 7342 | cg10963218 |
| 7343 | cg21600346 |
| 7344 | cg05626226 |
| 7345 | cg10146203 |
| 7346 | cg16921285 |
| 7347 | cg01132469 |
| 7348 | cg25612362 |
| 7349 | cg06277329 |
| 7350 | cg18145070 |
| 7351 | cg16246713 |
| 7352 | cg25102614 |
| 7353 | cg02009700 |
| 7354 | cg12144797 |
| 7355 | cg23994296 |
| 7356 | cg07931189 |
| 7357 | cg09933458 |
| 7358 | cg12327989 |
| 7359 | cg09017941 |
| 7360 | cg20698113 |
| 7361 | cg26987660 |
| 7362 | cg19057304 |
| 7363 | cg11218383 |
| 7364 | cg02637318 |
| 7365 | cg08602008 |
| 7366 | cg18999636 |
| 7367 | cg13273340 |
| 7368 | cg01861131 |
| 7369 | cg11852643 |
| 7370 | cg14193097 |
| 7371 | cg05384646 |
| 7372 | cg13769834 |
| 7373 | cg11229864 |
| 7374 | cg24702069 |
| 7375 | cg15509430 |
| 7376 | cg26154808 |
| 7377 | cg10749662 |
| 7378 | cg27179160 |
| 7379 | cg01803928 |
| 7380 | cg05493580 |
| 7381 | cg02554050 |
| 7382 | cg23281529 |
| 7383 | cg25659364 |
| 7384 | cg02305723 |
| 7385 | cg04845582 |
| 7386 | cg06922248 |
| 7387 | cg12762862 |
| 7388 | cg04971763 |
| 7389 | cg26656258 |
| 7390 | cg13132440 |
| 7391 | cg19539667 |
| 7392 | cg16902746 |
| 7393 | cg07329024 |
| 7394 | cg13777759 |
| 7395 | cg24890104 |
| 7396 | cg25837978 |
| 7397 | cg01942845 |
| 7398 | cg23163283 |
| 7399 | cg09332960 |
| 7400 | cg14800136 |
| 7401 | cg25047618 |
| 7402 | cg18021626 |
| 7403 | cg11040546 |
| 7404 | cg02909446 |
| 7405 | cg22937649 |
| 7406 | cg04026169 |
| 7407 | cg11335335 |
| 7408 | cg08293102 |
| 7409 | cg09281528 |
| 7410 | cg09558425 |
| 7411 | cg08271804 |
| 7412 | cg12513880 |
| 7413 | cg20275928 |
| 7414 | cg13578966 |
| 7415 | cg04960536 |
| 7416 | cg18541042 |
| 7417 | cg10218212 |
| 7418 | cg23777233 |
| 7419 | cg08551019 |
| 7420 | cg22086461 |
| 7421 | cg00272622 |
| 7422 | cg19070894 |
| 7423 | cg16851482 |
| 7424 | cg10825151 |
| 7425 | cg08127053 |
| 7426 | cg24037375 |
| 7427 | cg11704005 |
| 7428 | cg21425062 |
| 7429 | cg13923807 |
| 7430 | cg03176205 |
| 7431 | cg11315449 |
| 7432 | cg23097006 |
| 7433 | cg13020664 |
| 7434 | cg04393532 |
| 7435 | cg13781963 |
| 7436 | cg17107156 |
| 7437 | cg23516261 |
| 7438 | cg05702597 |
| 7439 | cg05904364 |
| 7440 | cg08068532 |
| 7441 | cg14648920 |
| 7442 | cg07911233 |
| 7443 | cg24126967 |
| 7444 | cg26654770 |
| 7445 | cg17706091 |
| 7446 | cg24356441 |
| 7447 | cg25646531 |
| 7448 | cg23297742 |
| 7449 | cg19907796 |
| 7450 | cg11012412 |
| 7451 | cg07181030 |
| 7452 | cg16156273 |
| 7453 | cg02988673 |
| 7454 | cg19575753 |
| 7455 | cg03452630 |
| 7456 | cg22603280 |
| 7457 | cg27015792 |
| 7458 | cg26109791 |
| 7459 | cg08554603 |
| 7460 | cg06346266 |
| 7461 | cg26318111 |
| 7462 | cg05341932 |
| 7463 | cg14210607 |
| 7464 | cg17422692 |
| 7465 | cg25790164 |
| 7466 | cg12458763 |
| 7467 | cg05069228 |
| 7468 | cg26554505 |
| 7469 | cg03606772 |
| 7470 | cg18049300 |
| 7471 | cg07891627 |
| 7472 | cg22302675 |
| 7473 | cg08944940 |
| 7474 | cg06774142 |
| 7475 | cg00573835 |
| 7476 | cg11841894 |
| 7477 | cg08942527 |
| 7478 | cg16446543 |
| 7479 | cg14186187 |
| 7480 | cg01233487 |
| 7481 | cg23286992 |
| 7482 | cg19938199 |
| 7483 | cg20381115 |
| 7484 | cg27229613 |
| 7485 | cg08766170 |
| 7486 | cg08224920 |
| 7487 | cg25110204 |
| 7488 | cg17316760 |
| 7489 | cg09371284 |
| 7490 | cg17853216 |
| 7491 | cg21212076 |
| 7492 | cg20368294 |
| 7493 | cg02866177 |
| 7494 | cg11501313 |
| 7495 | cg00372774 |
| 7496 | cg17904897 |
| 7497 | cg19258425 |
| 7498 | cg15033098 |
| 7499 | cg19466818 |
| 7500 | cg20111803 |
| 7501 | cg07323825 |
| 7502 | cg07202552 |
| 7503 | cg07318417 |
| 7504 | cg24593764 |
| 7505 | cg06421678 |
| 7506 | cg27299814 |
| 7507 | cg19882915 |
| 7508 | cg19780308 |
| 7509 | cg06511343 |
| 7510 | cg16317457 |
| 7511 | cg18401367 |
| 7512 | cg08134504 |
| 7513 | cg15768901 |
| 7514 | cg00653041 |
| 7515 | cg25179016 |
| 7516 | cg06154432 |
| 7517 | cg18886943 |
| 7518 | cg18602350 |
| 7519 | cg11549065 |
| 7520 | cg19303908 |
| 7521 | cg02124383 |
| 7522 | cg03485608 |
| 7523 | cg17853378 |
| 7524 | cg01386185 |
| 7525 | cg16340422 |
| 7526 | cg16277218 |
| 7527 | cg12645202 |
| 7528 | cg09615056 |
| 7529 | cg02267483 |
| 7530 | cg26189827 |
| 7531 | cg03330485 |
| 7532 | cg25531179 |
| 7533 | cg11861507 |
| 7534 | cg13407169 |
| 7535 | cg07337793 |
| 7536 | cg24813323 |
| 7537 | cg05385282 |
| 7538 | cg05668703 |
| 7539 | cg08919659 |
| 7540 | cg09474229 |
| 7541 | cg15599832 |
| 7542 | cg01514552 |
| 7543 | cg07660236 |
| 7544 | cg27514038 |
| 7545 | cg15890774 |
| 7546 | cg21852589 |
| 7547 | cg00386413 |
| 7548 | cg07501816 |
| 7549 | cg21754876 |
| 7550 | cg05424126 |
| 7551 | cg19090871 |
| 7552 | cg24000528 |
| 7553 | cg05156901 |
| 7554 | cg27645488 |
| 7555 | cg16794506 |
| 7556 | cg04700814 |
| 7557 | cg22728904 |
| 7558 | cg03548412 |
| 7559 | cg11177698 |
| 7560 | cg10881749 |
| 7561 | cg08535149 |
| 7562 | cg26301167 |
| 7563 | cg05421423 |
| 7564 | cg11563844 |
| 7565 | cg17666539 |
| 7566 | cg14879279 |
| 7567 | cg02693033 |
| 7568 | cg08545632 |
| 7569 | cg10344369 |
| 7570 | cg23601597 |
| 7571 | cg08409173 |
| 7572 | cg11517269 |
| 7573 | cg25267982 |
| 7574 | cg05048624 |
| 7575 | cg03003335 |
| 7576 | cg11406388 |
| 7577 | cg06370132 |
| 7578 | cg21602882 |
| 7579 | cg07900121 |
| 7580 | cg20132513 |
| 7581 | cg04747102 |
| 7582 | cg11089489 |
| 7583 | cg01207337 |
| 7584 | cg19542849 |
| 7585 | cg04018325 |
| 7586 | cg10833066 |
| 7587 | cg17501823 |
| 7588 | cg17677490 |
| 7589 | cg10614768 |
| 7590 | cg01270206 |
| 7591 | cg12402265 |
| 7592 | cg16705086 |
| 7593 | cg00151680 |
| 7594 | cg15716210 |
| 7595 | cg13007207 |
| 7596 | cg08983883 |
| 7597 | cg05628616 |
| 7598 | cg23109530 |
| 7599 | cg05650586 |
| 7600 | cg06905162 |
| 7601 | cg16949251 |
| 7602 | cg20434856 |
| 7603 | cg05992832 |
| 7604 | cg12651914 |
| 7605 | cg13006509 |
| 7606 | cg24023258 |
| 7607 | cg08071282 |
| 7608 | cg01642579 |
| 7609 | cg20912169 |
| 7610 | cg09135656 |
| 7611 | cg08678154 |
| 7612 | cg04845368 |
| 7613 | cg14187844 |
| 7614 | cg13580419 |
| 7615 | cg02262553 |
| 7616 | cg14159452 |
| 7617 | cg19144974 |
| 7618 | cg05275229 |
| 7619 | cg17177779 |
| 7620 | cg27488824 |
| 7621 | cg04545015 |
| 7622 | cg11410682 |
| 7623 | cg10888601 |
| 7624 | cg22593405 |
| 7625 | cg25882887 |
| 7626 | cg03252775 |
| 7627 | cg22583350 |
| 7628 | cg02805130 |
| 7629 | cg01266390 |
| 7630 | cg12869830 |
| 7631 | cg08832220 |
| 7632 | cg15037823 |
| 7633 | cg17412989 |
| 7634 | cg08489874 |
| 7635 | cg10665321 |
| 7636 | cg20991420 |
| 7637 | cg09840667 |
| 7638 | cg21093192 |
| 7639 | cg25260564 |
| 7640 | cg23251701 |
| 7641 | cg26155802 |
| 7642 | cg11212178 |
| 7643 | cg23097878 |
| 7644 | cg00740718 |
| 7645 | cg04299200 |
| 7646 | cg27502912 |
| 7647 | cg21186900 |
| 7648 | cg23066449 |
| 7649 | cg21335376 |
| 7650 | cg04464434 |
| 7651 | cg23267550 |
| 7652 | cg09450024 |
| 7653 | cg02132284 |
| 7654 | cg00278547 |
| 7655 | cg17513925 |
| 7656 | cg07645761 |
| 7657 | cg21593432 |
| 7658 | cg12817908 |
| 7659 | cg20260841 |
| 7660 | cg18620306 |
| 7661 | cg14020052 |
| 7662 | cg05272349 |
| 7663 | cg12654412 |
| 7664 | cg06055764 |
| 7665 | cg14803762 |
| 7666 | cg06825512 |
| 7667 | cg26701242 |
| 7668 | cg12465710 |
| 7669 | cg02718176 |
| 7670 | cg15842366 |
| 7671 | cg16135695 |
| 7672 | cg12320986 |
| 7673 | cg14129735 |
| 7674 | cg16496571 |
| 7675 | cg08974518 |
| 7676 | cg21007342 |
| 7677 | cg06889108 |
| 7678 | cg04087084 |
| 7679 | cg17355385 |
| 7680 | cg05685892 |
| 7681 | cg19143209 |
| 7682 | cg27094173 |
| 7683 | cg04038089 |
| 7684 | cg19788186 |
| 7685 | cg16622920 |
| 7686 | cg26890354 |
| 7687 | cg15712559 |
| 7688 | cg20174472 |
| 7689 | cg04666830 |
| 7690 | cg22311344 |
| 7691 | cg27573991 |
| 7692 | cg25028129 |
| 7693 | cg14983838 |
| 7694 | cg07569464 |
| 7695 | cg13038685 |
| 7696 | cg18751141 |
| 7697 | cg16585234 |
| 7698 | cg02148796 |
| 7699 | cg08750504 |
| 7700 | cg07547492 |
| 7701 | cg08901157 |
| 7702 | cg07060278 |
| 7703 | cg22911687 |
| 7704 | cg18778450 |
| 7705 | cg06921803 |
| 7706 | cg26160218 |
| 7707 | cg09129536 |
| 7708 | cg09817162 |
| 7709 | cg07976695 |
| 7710 | cg12369258 |
| 7711 | cg08683278 |
| 7712 | cg09885664 |
| 7713 | cg14162417 |
| 7714 | cg14976050 |
| 7715 | cg27490286 |
| 7716 | cg16426764 |
| 7717 | cg10909678 |
| 7718 | cg07661965 |
| 7719 | cg14402213 |
| 7720 | cg23233404 |
| 7721 | cg04796653 |
| 7722 | cg01756827 |
| 7723 | cg23144722 |
| 7724 | cg14989616 |
| 7725 | cg22545206 |
| 7726 | cg15084269 |
| 7727 | cg06055086 |
| 7728 | cg05639088 |
| 7729 | cg15733309 |
| 7730 | cg09479286 |
| 7731 | cg11367159 |
| 7732 | cg19016652 |
| 7733 | cg22050877 |
| 7734 | cg19904058 |
| 7735 | cg21237687 |
| 7736 | cg03191504 |
| 7737 | cg27021034 |
| 7738 | cg03795847 |
| 7739 | cg10257302 |
| 7740 | cg10432947 |
| 7741 | cg15516629 |
| 7742 | cg08035416 |
| 7743 | cg26336738 |
| 7744 | cg02148426 |
| 7745 | cg03083189 |
| 7746 | cg05667379 |
| 7747 | cg06104132 |
| 7748 | cg06364487 |
| 7749 | cg14462534 |
| 7750 | cg25094891 |
| 7751 | cg06086634 |
| 7752 | cg08594554 |
| 7753 | cg25866895 |
| 7754 | cg12586756 |
| 7755 | cg07810733 |
| 7756 | cg03482866 |
| 7757 | cg06147368 |
| 7758 | cg08520746 |
| 7759 | cg09061684 |
| 7760 | cg12708994 |
| 7761 | cg04371336 |
| 7762 | cg06995548 |
| 7763 | cg12581627 |
| 7764 | cg26818401 |
| 7765 | cg22523878 |
| 7766 | cg20288617 |
| 7767 | cg07974307 |
| 7768 | cg27444700 |
| 7769 | cg09235983 |
| 7770 | cg04118610 |
| 7771 | cg14341131 |
| 7772 | cg22868237 |
| 7773 | cg04938315 |
| 7774 | cg17105014 |
| 7775 | cg27589676 |
| 7776 | cg20697025 |
| 7777 | cg10400998 |
| 7778 | cg14947218 |
| 7779 | cg12839142 |
| 7780 | cg00320414 |
| 7781 | cg17672861 |
| 7782 | cg13052638 |
| 7783 | cg19470379 |
| 7784 | cg01083633 |
| 7785 | cg04903019 |
| 7786 | cg11944462 |
| 7787 | cg05656374 |
| 7788 | cg14093127 |
| 7789 | cg06290715 |
| 7790 | cg17098103 |
| 7791 | cg02300154 |
| 7792 | cg12802403 |
| 7793 | cg26907578 |
| 7794 | cg12303813 |
| 7795 | cg13366057 |
| 7796 | cg04917160 |
| 7797 | cg25655489 |
| 7798 | cg04031201 |
| 7799 | cg14300943 |
| 7800 | cg00644823 |
| 7801 | cg06789686 |
| 7802 | cg09988242 |
| 7803 | cg00650809 |
| 7804 | cg07294578 |
| 7805 | cg25636629 |
| 7806 | cg19815544 |
| 7807 | cg15771369 |
| 7808 | cg25765881 |
| 7809 | cg15991466 |
| 7810 | cg00298324 |
| 7811 | cg02468320 |
| 7812 | cg26058226 |
| 7813 | cg19656415 |
| 7814 | cg23766254 |
| 7815 | cg11138429 |
| 7816 | cg01699443 |
| 7817 | cg07249274 |
| 7818 | cg16961816 |
| 7819 | cg25873104 |
| 7820 | cg09495909 |
| 7821 | cg00650925 |
| 7822 | cg12088314 |
| 7823 | cg11189648 |
| 7824 | cg16668986 |
| 7825 | cg22985204 |
| 7826 | cg10196532 |
| 7827 | cg03868473 |
| 7828 | cg25944011 |
| 7829 | cg00268086 |
| 7830 | cg08733159 |
| 7831 | cg23945334 |
| 7832 | cg01656007 |
| 7833 | cg23331653 |
| 7834 | cg03758710 |
| 7835 | cg18451373 |
| 7836 | cg12937133 |
| 7837 | cg08506528 |
| 7838 | cg14383658 |
| 7839 | cg13337721 |
| 7840 | cg02236847 |
| 7841 | cg13799504 |
| 7842 | cg01856865 |
| 7843 | cg12835689 |
| 7844 | cg12082371 |
| 7845 | cg25067702 |
| 7846 | cg15873301 |
| 7847 | cg21346043 |
| 7848 | cg25116600 |
| 7849 | cg10545616 |
| 7850 | cg18699526 |
| 7851 | cg17364089 |
| 7852 | cg17799614 |
| 7853 | cg22796353 |
| 7854 | cg23537887 |
| 7855 | cg10212985 |
| 7856 | cg27128939 |
| 7857 | cg26733301 |
| 7858 | cg02653010 |
| 7859 | cg02167021 |
| 7860 | cg02964065 |
| 7861 | cg00030290 |
| 7862 | cg11171705 |
| 7863 | cg26645082 |
| 7864 | cg23816136 |
| 7865 | cg12648537 |
| 7866 | cg14080048 |
| 7867 | cg20140333 |
| 7868 | cg13510360 |
| 7869 | cg07107859 |
| 7870 | cg21542078 |
| 7871 | cg01354632 |
| 7872 | cg05715076 |
| 7873 | cg14399060 |
| 7874 | cg24202817 |
| 7875 | cg16431978 |
| 7876 | cg15299510 |
| 7877 | cg20704005 |
| 7878 | cg10169653 |
| 7879 | cg06977347 |
| 7880 | cg14624451 |
| 7881 | cg16655346 |
| 7882 | cg23916522 |
| 7883 | cg06500337 |
| 7884 | cg23581129 |
| 7885 | cg01493297 |
| 7886 | cg25940248 |
| 7887 | cg08543278 |
| 7888 | cg02182594 |
| 7889 | cg00156118 |
| 7890 | cg10239665 |
| 7891 | cg14533523 |
| 7892 | cg15375772 |
| 7893 | cg23844865 |
| 7894 | cg02558539 |
| 7895 | cg17191109 |
| 7896 | cg24296452 |
| 7897 | cg04003327 |
| 7898 | cg18248145 |
| 7899 | cg15810386 |
| 7900 | cg11447377 |
| 7901 | cg12439011 |
| 7902 | cg14219918 |
| 7903 | cg04176169 |
| 7904 | cg07732735 |
| 7905 | cg26886599 |
| 7906 | cg21342345 |
| 7907 | cg04697454 |
| 7908 | cg03208974 |
| 7909 | cg07768107 |
| 7910 | cg02286533 |
| 7911 | cg26552431 |
| 7912 | cg13985817 |
| 7913 | cg12302701 |
| 7914 | cg15773296 |
| 7915 | cg14606858 |
| 7916 | cg02346790 |
| 7917 | cg05387353 |
| 7918 | cg04841983 |
| 7919 | cg23112821 |
| 7920 | cg25674938 |
| 7921 | cg08004278 |
| 7922 | cg02189685 |
| 7923 | cg06990439 |
| 7924 | cg00530671 |
| 7925 | cg09538156 |
| 7926 | cg22585786 |
| 7927 | cg01730970 |
| 7928 | cg09572195 |
| 7929 | cg08729243 |
| 7930 | cg13413488 |
| 7931 | cg26767059 |
| 7932 | cg05731594 |
| 7933 | cg24390913 |
| 7934 | cg04551318 |
| 7935 | cg11357800 |
| 7936 | cg03353849 |
| 7937 | cg00247998 |
| 7938 | cg18813159 |
| 7939 | cg15414930 |
| 7940 | cg04797359 |
| 7941 | cg03472888 |
| 7942 | cg18918321 |
| 7943 | cg15899738 |
| 7944 | cg15200243 |
| 7945 | cg18349911 |
| 7946 | cg15839431 |
| 7947 | cg14029686 |
| 7948 | cg19239041 |
| 7949 | cg21321626 |
| 7950 | cg10771262 |
| 7951 | cg24249248 |
| 7952 | cg03305585 |
| 7953 | cg21827912 |
| 7954 | cg26187205 |
| 7955 | cg12246177 |
| 7956 | cg24061197 |
| 7957 | cg16349534 |
| 7958 | cg00617875 |
| 7959 | cg09012532 |
| 7960 | cg13703437 |
| 7961 | cg07742368 |
| 7962 | cg08819802 |
| 7963 | cg23078268 |
| 7964 | cg05999317 |
| 7965 | cg02696327 |
| 7966 | cg06432889 |
| 7967 | cg14515812 |
| 7968 | cg17841267 |
| 7969 | cg13906860 |
| 7970 | cg25992382 |
| 7971 | cg23462772 |
| 7972 | cg17716457 |
| 7973 | cg13843993 |
| 7974 | cg19299265 |
| 7975 | cg17177536 |
| 7976 | cg22475974 |
| 7977 | cg03679755 |
| 7978 | cg23255942 |
| 7979 | cg12853970 |
| 7980 | cg07863545 |
| 7981 | cg21908542 |
| 7982 | cg15669600 |
| 7983 | cg18151030 |
| 7984 | cg11319389 |
| 7985 | cg11681041 |
| 7986 | cg11363999 |
| 7987 | cg13947534 |
| 7988 | cg20619987 |
| 7989 | cg02712669 |
| 7990 | cg22363614 |
| 7991 | cg24652615 |
| 7992 | cg07514618 |
| 7993 | cg10890106 |
| 7994 | cg16146501 |
| 7995 | cg02593440 |
| 7996 | cg10454246 |
| 7997 | cg08584759 |
| 7998 | cg22024479 |
| 7999 | cg16311302 |
| 8000 | cg18637486 |
| 8001 | cg05794070 |
| 8002 | cg17828445 |
| 8003 | cg27452619 |
| 8004 | cg14839558 |
| 8005 | cg20239071 |
| 8006 | cg12541454 |
| 8007 | cg00545801 |
| 8008 | cg01780345 |
| 8009 | cg16414472 |
| 8010 | cg06076690 |
| 8011 | cg21320455 |
| 8012 | cg21936552 |
| 8013 | cg06441265 |
| 8014 | cg18800754 |
| 8015 | cg11565786 |
| 8016 | cg18660345 |
| 8017 | cg00313094 |
| 8018 | cg10232759 |
| 8019 | cg13731808 |
| 8020 | cg01997101 |
| 8021 | cg11891591 |
| 8022 | cg27413008 |
| 8023 | cg01218047 |
| 8024 | cg09966085 |
| 8025 | cg08065657 |
| 8026 | cg01039355 |
| 8027 | cg06862136 |
| 8028 | cg01856536 |
| 8029 | cg19966787 |
| 8030 | cg25805864 |
| 8031 | cg20220755 |
| 8032 | cg23094620 |
| 8033 | cg07045155 |
| 8034 | cg09772792 |
| 8035 | cg04506894 |
| 8036 | cg18248753 |
| 8037 | cg23854860 |
| 8038 | cg25441466 |
| 8039 | cg21833714 |
| 8040 | cg22676516 |
| 8041 | cg17632750 |
| 8042 | cg04160427 |
| 8043 | cg08881680 |
| 8044 | cg03429763 |
| 8045 | cg03232465 |
| 8046 | cg23316648 |
| 8047 | cg23275390 |
| 8048 | cg23378736 |
| 8049 | cg15622158 |
| 8050 | cg17812972 |
| 8051 | cg22285110 |
| 8052 | cg08195415 |
| 8053 | cg11333375 |
| 8054 | cg23176316 |
| 8055 | cg15072091 |
| 8056 | cg06436488 |
| 8057 | cg04723534 |
| 8058 | cg02028114 |
| 8059 | cg16201883 |
| 8060 | cg08598454 |
| 8061 | cg22804236 |
| 8062 | cg15005368 |
| 8063 | cg17109725 |
| 8064 | cg09218557 |
| 8065 | cg19505458 |
| 8066 | cg13663931 |
| 8067 | cg13115502 |
| 8068 | cg11382133 |
| 8069 | cg10709593 |
| 8070 | cg16167060 |
| 8071 | cg09718037 |
| 8072 | cg17543239 |
| 8073 | cg16016281 |
| 8074 | cg08959039 |
| 8075 | cg20245362 |
| 8076 | cg08512167 |
| 8077 | cg24307499 |
| 8078 | cg23360154 |
| 8079 | cg16810626 |
| 8080 | cg09232069 |
| 8081 | cg00497735 |
| 8082 | cg00500789 |
| 8083 | cg03196097 |
| 8084 | cg08994342 |
| 8085 | cg24396605 |
| 8086 | cg07355157 |
| 8087 | cg05344372 |
| 8088 | cg10552807 |
| 8089 | cg16209643 |
| 8090 | cg22560937 |
| 8091 | cg19228287 |
| 8092 | cg08164191 |
| 8093 | cg20675391 |
| 8094 | cg02923485 |
| 8095 | cg18703361 |
| 8096 | cg12884378 |
| 8097 | cg06079082 |
| 8098 | cg01948202 |
| 8099 | cg06026136 |
| 8100 | cg25254074 |
| 8101 | cg15961693 |
| 8102 | cg09714458 |
| 8103 | cg12018718 |
| 8104 | cg27360210 |
| 8105 | cg09217258 |
| 8106 | cg14842777 |
| 8107 | cg10249243 |
| 8108 | cg02729344 |
| 8109 | cg09659684 |
| 8110 | cg11719881 |
| 8111 | cg23035341 |
| 8112 | cg13708055 |
| 8113 | cg23579185 |
| 8114 | cg22835712 |
| 8115 | cg09117778 |
| 8116 | cg12284090 |
| 8117 | cg04008252 |
| 8118 | cg19788371 |
| 8119 | cg10134527 |
| 8120 | cg24724587 |
| 8121 | cg24749559 |
| 8122 | cg13967811 |
| 8123 | cg10685856 |
| 8124 | cg02077648 |
| 8125 | cg13052768 |
| 8126 | cg05155812 |
| 8127 | cg24796720 |
| 8128 | cg27065146 |
| 8129 | cg12638731 |
| 8130 | cg26495303 |
| 8131 | cg05911659 |
| 8132 | cg13180165 |
| 8133 | cg03930209 |
| 8134 | cg05525812 |
| 8135 | cg00085434 |
| 8136 | cg08660562 |
| 8137 | cg11728295 |
| 8138 | cg08466034 |
| 8139 | cg09437808 |
| 8140 | cg02423618 |
| 8141 | cg21535253 |
| 8142 | cg01333011 |
| 8143 | cg13058534 |
| 8144 | cg15503752 |
| 8145 | cg16897942 |
| 8146 | cg06899274 |
| 8147 | cg20928945 |
| 8148 | cg04332433 |
| 8149 | cg18313762 |
| 8150 | cg21838487 |
| 8151 | cg17962575 |
| 8152 | cg13235447 |
| 8153 | cg09354154 |
| 8154 | cg21988119 |
| 8155 | cg21132420 |
| 8156 | cg10172675 |
| 8157 | cg14356919 |
| 8158 | cg25087341 |
| 8159 | cg01868869 |
| 8160 | cg04153838 |
| 8161 | cg15819924 |
| 8162 | cg14966648 |
| 8163 | cg13100060 |
| 8164 | cg20249071 |
| 8165 | cg02841912 |
| 8166 | cg14099579 |
| 8167 | cg16322121 |
| 8168 | cg09615411 |
| 8169 | cg03975694 |
| 8170 | cg23013854 |
| 8171 | cg27208307 |
| 8172 | cg01616200 |
| 8173 | cg15460899 |
| 8174 | cg21033965 |
| 8175 | cg09484938 |
| 8176 | cg10984625 |
| 8177 | cg24422618 |
| 8178 | cg02445968 |
| 8179 | cg14215472 |
| 8180 | cg27243955 |
| 8181 | cg06583111 |
| 8182 | cg22513901 |
| 8183 | cg02176715 |
| 8184 | cg08108750 |
| 8185 | cg01605435 |
| 8186 | cg00745693 |
| 8187 | cg08143343 |
| 8188 | cg13083037 |
| 8189 | cg12405136 |
| 8190 | cg10781513 |
| 8191 | cg08301981 |
| 8192 | cg17090934 |
| 8193 | cg02876871 |
| 8194 | cg00181834 |
| 8195 | cg13597851 |
| 8196 | cg01830154 |
| 8197 | cg13315147 |
| 8198 | cg06157941 |
| 8199 | cg17907003 |
| 8200 | cg24170700 |
| 8201 | cg09569989 |
| 8202 | cg18362821 |
| 8203 | cg07331286 |
| 8204 | cg22997113 |
| 8205 | cg26792900 |
| 8206 | cg14754556 |
| 8207 | cg03871267 |
| 8208 | cg19520927 |
| 8209 | cg14317414 |
| 8210 | cg04545585 |
| 8211 | cg23612626 |
| 8212 | cg14524975 |
| 8213 | cg04759104 |
| 8214 | cg24252907 |
| 8215 | cg08478672 |
| 8216 | cg11602997 |
| 8217 | cg13055709 |
| 8218 | cg18819619 |
| 8219 | cg10471825 |
| 8220 | cg00468228 |
| 8221 | cg27521071 |
| 8222 | cg17598923 |
| 8223 | cg24557305 |
| 8224 | cg19474865 |
| 8225 | cg25562417 |
| 8226 | cg26319551 |
| 8227 | cg26857315 |
| 8228 | cg07484910 |
| 8229 | cg07319528 |
| 8230 | cg26910675 |
| 8231 | cg14907603 |
| 8232 | cg13284285 |
| 8233 | cg08853123 |
| 8234 | cg04810284 |
| 8235 | cg15742063 |
| 8236 | cg16301894 |
| 8237 | cg15026265 |
| 8238 | cg12904098 |
| 8239 | cg21538389 |
| 8240 | cg18884865 |
| 8241 | cg26099834 |
| 8242 | cg12266591 |
| 8243 | cg05054998 |
| 8244 | cg00510437 |
| 8245 | cg15814717 |
| 8246 | cg07209719 |
| 8247 | cg03327325 |
| 8248 | cg11749828 |
| 8249 | cg05021643 |
| 8250 | cg08696107 |
| 8251 | cg05190930 |
| 8252 | cg08271229 |
| 8253 | cg14318759 |
| 8254 | cg08089183 |
| 8255 | cg05557582 |
| 8256 | cg05976283 |
| 8257 | cg03694486 |
| 8258 | cg20600379 |
| 8259 | cg12619509 |
| 8260 | cg16820615 |
| 8261 | cg08734647 |
| 8262 | cg19550574 |
| 8263 | cg01612158 |
| 8264 | cg12959840 |
| 8265 | cg02626395 |
| 8266 | cg26394257 |
| 8267 | cg07393032 |
| 8268 | cg09940032 |
| 8269 | cg15348720 |
| 8270 | cg03179435 |
| 8271 | cg08481633 |
| 8272 | cg04376808 |
| 8273 | cg27382744 |
| 8274 | cg16511524 |
| 8275 | cg23923562 |
| 8276 | cg13033202 |
| 8277 | cg02704949 |
| 8278 | cg20008800 |
| 8279 | cg26891236 |
| 8280 | cg14585242 |
| 8281 | cg18896948 |
| 8282 | cg27154217 |
| 8283 | cg21144941 |
| 8284 | cg01963633 |
| 8285 | cg05047156 |
| 8286 | cg22928768 |
| 8287 | cg22189626 |
| 8288 | cg18749015 |
| 8289 | cg09526758 |
| 8290 | cg08241360 |
| 8291 | cg00598009 |
| 8292 | cg10507482 |
| 8293 | cg13997937 |
| 8294 | cg05131483 |
| 8295 | cg11350586 |
| 8296 | cg19301897 |
| 8297 | cg16329721 |
| 8298 | cg03454775 |
| 8299 | cg03716161 |
| 8300 | cg22635935 |
| 8301 | cg00510842 |
| 8302 | cg23870749 |
| 8303 | cg26817877 |
| 8304 | cg06032302 |
| 8305 | cg19108485 |
| 8306 | cg09586924 |
| 8307 | cg16406790 |
| 8308 | cg24146288 |
| 8309 | cg09467607 |
| 8310 | cg23879743 |
| 8311 | cg20293725 |
| 8312 | cg26323755 |
| 8313 | cg07857469 |
| 8314 | cg04406111 |
| 8315 | cg25257677 |
| 8316 | cg05343811 |
| 8317 | cg05547895 |
| 8318 | cg06756047 |
| 8319 | cg04382643 |
| 8320 | cg27319216 |
| 8321 | cg00832546 |
| 8322 | cg22063139 |
| 8323 | cg09610558 |
| 8324 | cg10657950 |
| 8325 | cg12984948 |
| 8326 | cg23698632 |
| 8327 | cg07911080 |
| 8328 | cg11078128 |
| 8329 | cg18424208 |
| 8330 | cg23999621 |
| 8331 | cg23331981 |
| 8332 | cg14498423 |
| 8333 | cg16641320 |
| 8334 | cg26751588 |
| 8335 | cg26879421 |
| 8336 | cg07137382 |
| 8337 | cg09449447 |
| 8338 | cg20944964 |
| 8339 | cg11173447 |
| 8340 | cg27501534 |
| 8341 | cg03030267 |
| 8342 | cg15528441 |
| 8343 | cg16887940 |
| 8344 | cg14186135 |
| 8345 | cg24096902 |
| 8346 | cg15635633 |
| 8347 | cg17671383 |
| 8348 | cg27393610 |
| 8349 | cg08323777 |
| 8350 | cg05306775 |
| 8351 | cg15183772 |
| 8352 | cg24068348 |
| 8353 | cg00763961 |
| 8354 | cg24819607 |
| 8355 | cg05029815 |
| 8356 | cg03224418 |
| 8357 | cg11795066 |
| 8358 | cg19064493 |
| 8359 | cg22738686 |
| 8360 | cg21639713 |
| 8361 | cg08515640 |
| 8362 | cg20255370 |
| 8363 | cg06071058 |
| 8364 | cg09056327 |
| 8365 | cg26656751 |
| 8366 | cg24754223 |
| 8367 | cg04276301 |
| 8368 | cg10800464 |
| 8369 | cg20734448 |
| 8370 | cg08068800 |
| 8371 | cg17202948 |
| 8372 | cg05767426 |
| 8373 | cg04267152 |
| 8374 | cg09163412 |
| 8375 | cg15519237 |
| 8376 | cg21004896 |
| 8377 | cg17445936 |
| 8378 | cg06760830 |
| 8379 | cg13615592 |
| 8380 | cg23334662 |
| 8381 | cg00546915 |
| 8382 | cg17278327 |
| 8383 | cg15358372 |
| 8384 | cg00519537 |
| 8385 | cg23211065 |
| 8386 | cg02876611 |
| 8387 | cg16043223 |
| 8388 | cg16898460 |
| 8389 | cg02970714 |
| 8390 | cg02273889 |
| 8391 | cg27629882 |
| 8392 | cg04398180 |
| 8393 | cg06458557 |
| 8394 | cg16252308 |
| 8395 | cg20431748 |
| 8396 | cg02480675 |
| 8397 | cg00434886 |
| 8398 | cg13629815 |
| 8399 | cg09016797 |
| 8400 | cg19567689 |
| 8401 | cg10384919 |
| 8402 | cg15425253 |
| 8403 | cg08870976 |
| 8404 | cg05249959 |
| 8405 | cg21583284 |
| 8406 | cg18257840 |
| 8407 | cg08371490 |
| 8408 | cg13074203 |
| 8409 | cg06724812 |
| 8410 | cg22109795 |
| 8411 | cg16519399 |
| 8412 | cg10438439 |
| 8413 | cg10769937 |
| 8414 | cg21005510 |
| 8415 | cg17707805 |
| 8416 | cg06809965 |
| 8417 | cg20238855 |
| 8418 | cg01524278 |
| 8419 | cg03652686 |
| 8420 | cg24437523 |
| 8421 | cg27071397 |
| 8422 | cg08671488 |
| 8423 | cg19580937 |
| 8424 | cg26986226 |
| 8425 | cg15268136 |
| 8426 | cg02274103 |
| 8427 | cg20729184 |
| 8428 | cg12624215 |
| 8429 | cg23710751 |
| 8430 | cg08701183 |
| 8431 | cg17086320 |
| 8432 | cg26664137 |
| 8433 | cg17102034 |
| 8434 | cg03652711 |
| 8435 | cg11991398 |
| 8436 | cg04988103 |
| 8437 | cg15952457 |
| 8438 | cg08521684 |
| 8439 | cg10714639 |
| 8440 | cg19342952 |
| 8441 | cg06349450 |
| 8442 | cg10528387 |
| 8443 | cg09609106 |
| 8444 | cg02694810 |
| 8445 | cg11962868 |
| 8446 | cg02798909 |
| 8447 | cg13859764 |
| 8448 | cg20765408 |
| 8449 | cg03643077 |
| 8450 | cg18772231 |
| 8451 | cg22087540 |
| 8452 | cg08966188 |
| 8453 | cg24363820 |
| 8454 | cg14760797 |
| 8455 | cg19827182 |
| 8456 | cg01554723 |
| 8457 | cg04980805 |
| 8458 | cg03081173 |
| 8459 | cg26360792 |
| 8460 | cg18037826 |
| 8461 | cg25146488 |
| 8462 | cg08289525 |
| 8463 | cg06733479 |
| 8464 | cg07902369 |
| 8465 | cg14002061 |
| 8466 | cg05437294 |
| 8467 | cg00549475 |
| 8468 | cg18186343 |
| 8469 | cg08659179 |
| 8470 | cg06482428 |
| 8471 | cg17465304 |
| 8472 | cg20499714 |
| 8473 | cg17413439 |
| 8474 | cg27649971 |
| 8475 | cg24314587 |
| 8476 | cg19506623 |
| 8477 | cg01172872 |
| 8478 | cg23243378 |
| 8479 | cg09375033 |
| 8480 | cg11408937 |
| 8481 | cg02805410 |
| 8482 | cg23634401 |
| 8483 | cg09596674 |
| 8484 | cg20712569 |
| 8485 | cg04581187 |
| 8486 | cg20512501 |
| 8487 | cg22431025 |
| 8488 | cg15868235 |
| 8489 | cg03128011 |
| 8490 | cg03626285 |
| 8491 | cg24263062 |
| 8492 | cg01351601 |
| 8493 | cg23986620 |
| 8494 | cg07873325 |
| 8495 | cg24361808 |
| 8496 | cg11385488 |
| 8497 | cg04075738 |
| 8498 | cg20267775 |
| 8499 | cg05452406 |
| 8500 | cg15880120 |
| 8501 | cg16201792 |
| 8502 | cg11015059 |
| 8503 | cg10486834 |
| 8504 | cg10098273 |
| 8505 | cg08299063 |
| 8506 | cg16126820 |
| 8507 | cg15565057 |
| 8508 | cg07124867 |
| 8509 | cg11198895 |
| 8510 | cg18331519 |
| 8511 | cg24935463 |
| 8512 | cg03895593 |
| 8513 | cg13912714 |
| 8514 | cg25534756 |
| 8515 | cg26632665 |
| 8516 | cg01305625 |
| 8517 | cg18791099 |
| 8518 | cg16797699 |
| 8519 | cg24320234 |
| 8520 | cg13339825 |
| 8521 | cg23887948 |
| 8522 | cg15952487 |
| 8523 | cg01501312 |
| 8524 | cg05170513 |
| 8525 | cg20744756 |
| 8526 | cg14833891 |
| 8527 | cg20176142 |
| 8528 | cg05682942 |
| 8529 | cg08497772 |
| 8530 | cg24236953 |
| 8531 | cg13160891 |
| 8532 | cg24469464 |
| 8533 | cg09021980 |
| 8534 | cg12876356 |
| 8535 | cg08135406 |
| 8536 | cg18527248 |
| 8537 | cg20130085 |
| 8538 | cg06741530 |
| 8539 | cg12496433 |
| 8540 | cg18149485 |
| 8541 | cg16732650 |
| 8542 | cg23668907 |
| 8543 | cg24519156 |
| 8544 | cg08013100 |
| 8545 | cg17722675 |
| 8546 | cg11166453 |
| 8547 | cg07366916 |
| 8548 | cg15912086 |
| 8549 | cg04745032 |
| 8550 | cg22910462 |
| 8551 | cg19240678 |
| 8552 | cg20218614 |
| 8553 | cg02158090 |
| 8554 | cg18258224 |
| 8555 | cg24902269 |
| 8556 | cg16714154 |
| 8557 | cg09447778 |
| 8558 | cg12097883 |
| 8559 | cg01678454 |
| 8560 | cg23302214 |
| 8561 | cg20580524 |
| 8562 | cg22452170 |
| 8563 | cg17589597 |
| 8564 | cg02233915 |
| 8565 | cg12080909 |
| 8566 | cg17045314 |
| 8567 | cg06996254 |
| 8568 | cg26808297 |
| 8569 | cg06906406 |
| 8570 | cg13484553 |
| 8571 | cg24848724 |
| 8572 | cg05554000 |
| 8573 | cg18517680 |
| 8574 | cg20314884 |
| 8575 | cg25830530 |
| 8576 | cg05349089 |
| 8577 | cg23829273 |
| 8578 | cg07561747 |
| 8579 | cg24463519 |
| 8580 | cg18052511 |
| 8581 | cg18161890 |
| 8582 | cg18239246 |
| 8583 | cg11686737 |
| 8584 | cg20972453 |
| 8585 | cg25916993 |
| 8586 | cg04695732 |
| 8587 | cg04612444 |
| 8588 | cg09719491 |
| 8589 | cg27282397 |
| 8590 | cg25984601 |
| 8591 | cg26277620 |
| 8592 | cg03238075 |
| 8593 | cg19953427 |
| 8594 | cg13854271 |
| 8595 | cg24527008 |
| 8596 | cg05760765 |
| 8597 | cg07471943 |
| 8598 | cg06316219 |
| 8599 | cg07020596 |
| 8600 | cg18044572 |
| 8601 | cg07079560 |
| 8602 | cg01965552 |
| 8603 | cg27302190 |
| 8604 | cg12058875 |
| 8605 | cg07384961 |
| 8606 | cg04070007 |
| 8607 | cg08878896 |
| 8608 | cg18960289 |
| 8609 | cg23677426 |
| 8610 | cg06034127 |
| 8611 | cg06587806 |
| 8612 | cg18137308 |
| 8613 | cg10084770 |
| 8614 | cg07400146 |
| 8615 | cg20058937 |
| 8616 | cg18435832 |
| 8617 | cg26882827 |
| 8618 | cg08887327 |
| 8619 | cg16404064 |
| 8620 | cg15152819 |
| 8621 | cg02383368 |
| 8622 | cg23193277 |
| 8623 | cg08388413 |
| 8624 | cg26692003 |
| 8625 | cg12113981 |
| 8626 | cg00009196 |
| 8627 | cg13019868 |
| 8628 | cg06834998 |
| 8629 | cg08465795 |
| 8630 | cg00871487 |
| 8631 | cg01511718 |
| 8632 | cg23892192 |
| 8633 | cg24847621 |
| 8634 | cg27237541 |
| 8635 | cg01019587 |
| 8636 | cg18193764 |
| 8637 | cg26031054 |
| 8638 | cg22590775 |
| 8639 | cg05256053 |
| 8640 | cg01043196 |
| 8641 | cg18720973 |
| 8642 | cg27174787 |
| 8643 | cg05449136 |
| 8644 | cg12872830 |
| 8645 | cg16702194 |
| 8646 | cg26292116 |
| 8647 | cg15015122 |
| 8648 | cg24285545 |
| 8649 | cg25812921 |
| 8650 | cg14356799 |
| 8651 | cg14083707 |
| 8652 | cg06900585 |
| 8653 | cg09970569 |
| 8654 | cg14451276 |
| 8655 | cg08187501 |
| 8656 | cg18692149 |
| 8657 | cg07679285 |
| 8658 | cg00221382 |
| 8659 | cg02971209 |
| 8660 | cg04203238 |
| 8661 | cg13349967 |
| 8662 | cg25651505 |
| 8663 | cg04478133 |
| 8664 | cg14523221 |
| 8665 | cg09646983 |
| 8666 | cg16083498 |
| 8667 | cg20273531 |
| 8668 | cg23808568 |
| 8669 | cg00316839 |
| 8670 | cg05690233 |
| 8671 | cg01092804 |
| 8672 | cg07452465 |
| 8673 | cg22446491 |
| 8674 | cg26349275 |
| 8675 | cg06199077 |
| 8676 | cg13916762 |
| 8677 | cg08374056 |
| 8678 | cg21156511 |
| 8679 | cg07403865 |
| 8680 | cg18001313 |
| 8681 | cg10175795 |
| 8682 | cg07696699 |
| 8683 | cg03252549 |
| 8684 | cg26673516 |
| 8685 | cg05583717 |
| 8686 | cg05473536 |
| 8687 | cg15601264 |
| 8688 | cg13763876 |
| 8689 | cg13337095 |
| 8690 | cg09791621 |
| 8691 | cg11984750 |
| 8692 | cg19071976 |
| 8693 | cg13606025 |
| 8694 | cg20315150 |
| 8695 | cg25267485 |
| 8696 | cg22596998 |
| 8697 | cg00653429 |
| 8698 | cg02482566 |
| 8699 | cg20157753 |
| 8700 | cg25902889 |
| 8701 | cg03423979 |
| 8702 | cg11153375 |
| 8703 | cg24404878 |
| 8704 | cg01639705 |
| 8705 | cg03714522 |
| 8706 | cg26990023 |
| 8707 | cg23201527 |
| 8708 | cg01026660 |
| 8709 | cg08719608 |
| 8710 | cg10099601 |
| 8711 | cg10704762 |
| 8712 | cg21715435 |
| 8713 | cg16723556 |
| 8714 | cg18771300 |
| 8715 | cg16163419 |
| 8716 | cg08751438 |
| 8717 | cg05925497 |
| 8718 | cg06122825 |
| 8719 | cg03402369 |
| 8720 | cg04295612 |
| 8721 | cg02957042 |
| 8722 | cg06473177 |
| 8723 | cg13079094 |
| 8724 | cg25661275 |
| 8725 | cg19961228 |
| 8726 | cg26266824 |
| 8727 | cg01178680 |
| 8728 | cg13262909 |
| 8729 | cg03211362 |
| 8730 | cg02079700 |
| 8731 | cg22209584 |
| 8732 | cg25973282 |
| 8733 | cg07068406 |
| 8734 | cg24549659 |
| 8735 | cg05790451 |
| 8736 | cg00939432 |
| 8737 | cg23027387 |
| 8738 | cg21898046 |
| 8739 | cg05576795 |
| 8740 | cg22363971 |
| 8741 | cg07972946 |
| 8742 | cg26785693 |
| 8743 | cg04867415 |
| 8744 | cg18897632 |
| 8745 | cg16793775 |
| 8746 | cg05388185 |
| 8747 | cg17246606 |
| 8748 | cg26522946 |
| 8749 | cg09400472 |
| 8750 | cg09134668 |
| 8751 | cg05555990 |
| 8752 | cg01845996 |
| 8753 | cg13303497 |
| 8754 | cg15231462 |
| 8755 | cg20951255 |
| 8756 | cg07107756 |
| 8757 | cg13374701 |
| 8758 | cg15367892 |
| 8759 | cg06627334 |
| 8760 | cg18597989 |
| 8761 | cg27522733 |
| 8762 | cg08656837 |
| 8763 | cg09854734 |
| 8764 | cg20482832 |
| 8765 | cg25203351 |
| 8766 | cg06246668 |
| 8767 | cg15549873 |
| 8768 | cg05222671 |
| 8769 | cg17679453 |
| 8770 | cg25652742 |
| 8771 | cg11067829 |
| 8772 | cg11859489 |
| 8773 | cg12103461 |
| 8774 | cg00942017 |
| 8775 | cg09062218 |
| 8776 | cg03354487 |
| 8777 | cg02622000 |
| 8778 | cg16073862 |
| 8779 | cg17335199 |
| 8780 | cg03252055 |
| 8781 | cg04254487 |
| 8782 | cg03528150 |
| 8783 | cg24569492 |
| 8784 | cg09506600 |
| 8785 | cg01589129 |
| 8786 | cg17046150 |
| 8787 | cg05591178 |
| 8788 | cg24381587 |
| 8789 | cg11017086 |
| 8790 | cg03744440 |
| 8791 | cg02182021 |
| 8792 | cg12689670 |
| 8793 | cg22506548 |
| 8794 | cg14322760 |
| 8795 | cg10129408 |
| 8796 | cg04110224 |
| 8797 | cg07899896 |
| 8798 | cg06108056 |
| 8799 | cg16891726 |
| 8800 | cg09078948 |
| 8801 | cg10638734 |
| 8802 | cg21506443 |
| 8803 | cg10788408 |
| 8804 | cg26856289 |
| 8805 | cg11674602 |
| 8806 | cg10828284 |
| 8807 | cg03169867 |
| 8808 | cg26865747 |
| 8809 | cg00847221 |
| 8810 | cg23063311 |
| 8811 | cg24010336 |
| 8812 | cg23997434 |
| 8813 | cg07820966 |
| 8814 | cg02785578 |
| 8815 | cg13026754 |
| 8816 | cg25278353 |
| 8817 | cg12295068 |
| 8818 | cg19926659 |
| 8819 | cg25999604 |
| 8820 | cg03745372 |
| 8821 | cg08554627 |
| 8822 | cg19307528 |
| 8823 | cg20477005 |
| 8824 | cg07074666 |
| 8825 | cg17361163 |
| 8826 | cg22473097 |
| 8827 | cg12298135 |
| 8828 | cg27423627 |
| 8829 | cg22322562 |
| 8830 | cg05130816 |
| 8831 | cg08051713 |
| 8832 | cg00334782 |
| 8833 | cg01865923 |
| 8834 | cg16416659 |
| 8835 | cg23230557 |
| 8836 | cg04237822 |
| 8837 | cg15354823 |
| 8838 | cg00023363 |
| 8839 | cg26456404 |
| 8840 | cg10504000 |
| 8841 | cg00565113 |
| 8842 | cg09994891 |
| 8843 | cg02643468 |
| 8844 | cg26315509 |
| 8845 | cg26997923 |
| 8846 | cg25940746 |
| 8847 | cg13387498 |
| 8848 | cg25690897 |
| 8849 | cg07839457 |
| 8850 | cg17006281 |
| 8851 | cg23224142 |
| 8852 | cg09038770 |
| 8853 | cg10631530 |
| 8854 | cg13419925 |
| 8855 | cg00274965 |
| 8856 | cg03051384 |
| 8857 | cg11183935 |
| 8858 | cg27554954 |
| 8859 | cg24141882 |
| 8860 | cg02919615 |
| 8861 | cg07361505 |
| 8862 | cg08742262 |
| 8863 | cg19593490 |
| 8864 | cg09862148 |
| 8865 | cg17419312 |
| 8866 | cg15258617 |
| 8867 | cg18558220 |
| 8868 | cg04191236 |
| 8869 | cg16815122 |
| 8870 | cg01332054 |
| 8871 | cg17597406 |
| 8872 | cg20557454 |
| 8873 | cg24699146 |
| 8874 | cg11132821 |
| 8875 | cg23051267 |
| 8876 | cg17005395 |
| 8877 | cg16041062 |
| 8878 | cg16293892 |
| 8879 | cg01121969 |
| 8880 | cg06637517 |
| 8881 | cg02605601 |
| 8882 | cg11107966 |
| 8883 | cg10951117 |
| 8884 | cg25137711 |
| 8885 | cg26438409 |
| 8886 | cg17266126 |
| 8887 | cg25497399 |
| 8888 | cg09511697 |
| 8889 | cg02443355 |
| 8890 | cg20914572 |
| 8891 | cg06895640 |
| 8892 | cg22045878 |
| 8893 | cg19551589 |
| 8894 | cg16423499 |
| 8895 | cg04322894 |
| 8896 | cg05249946 |
| 8897 | cg08845721 |
| 8898 | cg11949060 |
| 8899 | cg13067553 |
| 8900 | cg04989070 |
| 8901 | cg02873991 |
| 8902 | cg09086039 |
| 8903 | cg23729341 |
| 8904 | cg11285912 |
| 8905 | cg03172568 |
| 8906 | cg25865108 |
| 8907 | cg04460945 |
| 8908 | cg22812030 |
| 8909 | cg10632505 |
| 8910 | cg15397564 |
| 8911 | cg14899504 |
| 8912 | cg19589415 |
| 8913 | cg19248602 |
| 8914 | cg07214406 |
| 8915 | cg09851465 |
| 8916 | cg23325413 |
| 8917 | cg12802570 |
| 8918 | cg10810804 |
| 8919 | cg04480116 |
| 8920 | cg07343027 |
| 8921 | cg12315311 |
| 8922 | cg26817546 |
| 8923 | cg03237218 |
| 8924 | cg26036831 |
| 8925 | cg04394271 |
| 8926 | cg24553673 |
| 8927 | cg19195279 |
| 8928 | cg13941436 |
| 8929 | cg00560277 |
| 8930 | cg08857479 |
| 8931 | cg11344675 |
| 8932 | cg00731785 |
| 8933 | cg14803440 |
| 8934 | cg05089968 |
| 8935 | cg07180656 |
| 8936 | cg20400592 |
| 8937 | cg18963032 |
| 8938 | cg14289863 |
| 8939 | cg26505970 |
| 8940 | cg00598226 |
| 8941 | cg13613044 |
| 8942 | cg20824939 |
| 8943 | cg19021588 |
| 8944 | cg23989004 |
| 8945 | cg17982102 |
| 8946 | cg07636650 |
| 8947 | cg09250389 |
| 8948 | cg06922269 |
| 8949 | cg11930400 |
| 8950 | cg00579036 |
| 8951 | cg05901462 |
| 8952 | cg26333652 |
| 8953 | cg04650789 |
| 8954 | cg03003291 |
| 8955 | cg00997079 |
| 8956 | cg13268590 |
| 8957 | cg25157827 |
| 8958 | cg18720151 |
| 8959 | cg05774127 |
| 8960 | cg18633230 |
| 8961 | cg23823660 |
| 8962 | cg05466065 |
| 8963 | cg07838035 |
| 8964 | cg06710648 |
| 8965 | cg03334072 |
| 8966 | cg21206335 |
| 8967 | cg27584190 |
| 8968 | cg01042284 |
| 8969 | cg02229382 |
| 8970 | cg07894334 |
| 8971 | cg06320828 |
| 8972 | cg22378817 |
| 8973 | cg12173550 |
| 8974 | cg08918576 |
| 8975 | cg16597989 |
| 8976 | cg16216706 |
| 8977 | cg11256607 |
| 8978 | cg12303215 |
| 8979 | cg24867502 |
| 8980 | cg22166728 |
| 8981 | cg00866429 |
| 8982 | cg00812216 |
| 8983 | cg00697301 |
| 8984 | cg19253914 |
| 8985 | cg00930430 |
| 8986 | cg01663277 |
| 8987 | cg12025076 |
| 8988 | cg06991300 |
| 8989 | cg14595618 |
| 8990 | cg08551551 |
| 8991 | cg09759446 |
| 8992 | cg08378790 |
| 8993 | cg16761581 |
| 8994 | cg00977403 |
| 8995 | cg18496131 |
| 8996 | cg15593269 |
| 8997 | cg23962746 |
| 8998 | cg17329035 |
| 8999 | cg05749408 |
| 9000 | cg25489840 |
| 9001 | cg12775181 |
| 9002 | cg20372230 |
| 9003 | cg05541910 |
| 9004 | cg16484042 |
| 9005 | cg08467793 |
| 9006 | cg03689146 |
| 9007 | cg19373474 |
| 9008 | cg09570214 |
| 9009 | cg03753191 |
| 9010 | cg03405639 |
| 9011 | cg15504747 |
| 9012 | cg08023170 |
| 9013 | cg14048880 |
| 9014 | cg16738646 |
| 9015 | cg22463915 |
| 9016 | cg04627747 |
| 9017 | cg25843866 |
| 9018 | cg26673040 |
| 9019 | cg20950812 |
| 9020 | cg00250572 |
| 9021 | cg21002542 |
| 9022 | cg20547759 |
| 9023 | cg07719492 |
| 9024 | cg12537394 |
| 9025 | cg00290607 |
| 9026 | cg11094565 |
| 9027 | cg00083072 |
| 9028 | cg09029124 |
| 9029 | cg26092983 |
| 9030 | cg26821362 |
| 9031 | cg12011427 |
| 9032 | cg14236242 |
| 9033 | cg25628433 |
| 9034 | cg09469566 |
| 9035 | cg10737524 |
| 9036 | cg03544495 |
| 9037 | cg26660800 |
| 9038 | cg25456368 |
| 9039 | cg14584935 |
| 9040 | cg06713675 |
| 9041 | cg08895590 |
| 9042 | cg17586885 |
| 9043 | cg06970510 |
| 9044 | cg12520664 |
| 9045 | cg07946387 |
| 9046 | cg25507958 |
| 9047 | cg03461641 |
| 9048 | cg16359643 |
| 9049 | cg25367075 |
| 9050 | cg21442419 |
| 9051 | cg03701853 |
| 9052 | cg05922752 |
| 9053 | cg16681647 |
| 9054 | cg13794588 |
| 9055 | cg13123656 |
| 9056 | cg25909535 |
| 9057 | cg06219624 |
| 9058 | cg21048162 |
| 9059 | cg22598426 |
| 9060 | cg07207789 |
| 9061 | cg17890913 |
| 9062 | cg23678954 |
| 9063 | cg16902078 |
| 9064 | cg12231018 |
| 9065 | cg14377594 |
| 9066 | cg03811558 |
| 9067 | cg07277017 |
| 9068 | cg21511981 |
| 9069 | cg06812844 |
| 9070 | cg08076266 |
| 9071 | cg16685257 |
| 9072 | cg05093535 |
| 9073 | cg24746938 |
| 9074 | cg01949520 |
| 9075 | cg23380514 |
| 9076 | cg08619722 |
| 9077 | cg14524387 |
| 9078 | cg07855242 |
| 9079 | cg12137220 |
| 9080 | cg13524586 |
| 9081 | cg23453435 |
| 9082 | cg16227623 |
| 9083 | cg00861100 |
| 9084 | cg06831414 |
| 9085 | cg16686951 |
| 9086 | cg21242356 |
| 9087 | cg16593554 |
| 9088 | cg18034577 |
| 9089 | cg24397470 |
| 9090 | cg08488841 |
| 9091 | cg03107393 |
| 9092 | cg07368199 |
| 9093 | cg04596071 |
| 9094 | cg18669241 |
| 9095 | cg25104107 |
| 9096 | cg21636685 |
| 9097 | cg09652929 |
| 9098 | cg09645818 |
| 9099 | cg02142972 |
| 9100 | cg01615050 |
| 9101 | cg03186975 |
| 9102 | cg15225091 |
| 9103 | cg22541735 |
| 9104 | cg26647549 |
| 9105 | cg02210151 |
| 9106 | cg15500865 |
| 9107 | cg15925374 |
| 9108 | cg26932938 |
| 9109 | cg00397324 |
| 9110 | cg19746337 |
| 9111 | cg07384439 |
| 9112 | cg26373716 |
| 9113 | cg25040796 |
| 9114 | cg07909165 |
| 9115 | cg17540105 |
| 9116 | cg19603134 |
| 9117 | cg11255590 |
| 9118 | cg26850117 |
| 9119 | cg14947478 |
| 9120 | cg02854396 |
| 9121 | cg13318241 |
| 9122 | cg24539848 |
| 9123 | cg12593223 |
| 9124 | cg22727960 |
| 9125 | cg16646662 |
| 9126 | cg06650563 |
| 9127 | cg18867250 |
| 9128 | cg09439511 |
| 9129 | cg08468599 |
| 9130 | cg08863939 |
| 9131 | cg00773902 |
| 9132 | cg16614631 |
| 9133 | cg26532075 |
| 9134 | cg00816396 |
| 9135 | cg05701418 |
| 9136 | cg03827304 |
| 9137 | cg25731655 |
| 9138 | cg16479957 |
| 9139 | cg05811542 |
| 9140 | cg25314729 |
| 9141 | cg06571218 |
| 9142 | cg18693822 |
| 9143 | cg14963724 |
| 9144 | cg23806923 |
| 9145 | cg21435375 |
| 9146 | cg17751510 |
| 9147 | cg03961510 |
| 9148 | cg26644885 |
| 9149 | cg13182563 |
| 9150 | cg03266686 |
| 9151 | cg24418920 |
| 9152 | cg13506288 |
| 9153 | cg16784507 |
| 9154 | cg20876538 |
| 9155 | cg00648532 |
| 9156 | cg01230360 |
| 9157 | cg20343048 |
| 9158 | cg10654584 |
| 9159 | cg12700039 |
| 9160 | cg04704198 |
| 9161 | cg13883479 |
| 9162 | cg03919488 |
| 9163 | cg19908556 |
| 9164 | cg08818829 |
| 9165 | cg21273275 |
| 9166 | cg03228132 |
| 9167 | cg25286045 |
| 9168 | cg20100031 |
| 9169 | cg15441686 |
| 9170 | cg19763195 |
| 9171 | cg04148163 |
| 9172 | cg15574442 |
| 9173 | cg07908679 |
| 9174 | cg26697286 |
| 9175 | cg12232903 |
| 9176 | cg13320146 |
| 9177 | cg04609163 |
| 9178 | cg00562089 |
| 9179 | cg23768894 |
| 9180 | cg26581481 |
| 9181 | cg06374918 |
| 9182 | cg06746829 |
| 9183 | cg22323230 |
| 9184 | cg06951727 |
| 9185 | cg06428175 |
| 9186 | cg16891431 |
| 9187 | cg15204487 |
| 9188 | cg20472283 |
| 9189 | cg15621427 |
| 9190 | cg24680492 |
| 9191 | cg19619387 |
| 9192 | cg15399008 |
| 9193 | cg25614875 |
| 9194 | cg11305249 |
| 9195 | cg19215266 |
| 9196 | cg05197142 |
| 9197 | cg25891559 |
| 9198 | cg25163647 |
| 9199 | cg18206027 |
| 9200 | cg10210094 |
| 9201 | cg17054708 |
| 9202 | cg08118309 |
| 9203 | cg01234420 |
| 9204 | cg15339505 |
| 9205 | cg00568910 |
| 9206 | cg22153994 |
| 9207 | cg19169932 |
| 9208 | cg20113080 |
| 9209 | cg07163057 |
| 9210 | cg08981777 |
| 9211 | cg18260532 |
| 9212 | cg15340776 |
| 9213 | cg08401837 |
| 9214 | cg19432886 |
| 9215 | cg24926465 |
| 9216 | cg02962508 |
| 9217 | cg16696923 |
| 9218 | cg02016407 |
| 9219 | cg08290240 |
| 9220 | cg01943874 |
| 9221 | cg02200642 |
| 9222 | cg10474136 |
| 9223 | cg21737434 |
| 9224 | cg18776945 |
| 9225 | cg09918581 |
| 9226 | cg07157030 |
| 9227 | cg19706320 |
| 9228 | cg13823666 |
| 9229 | cg24233194 |
| 9230 | cg15046693 |
| 9231 | cg25103417 |
| 9232 | cg05860723 |
| 9233 | cg05376754 |
| 9234 | cg08792272 |
| 9235 | cg05350800 |
| 9236 | cg21884568 |
| 9237 | cg02996583 |
| 9238 | cg17285458 |
| 9239 | cg21988207 |
| 9240 | cg22894301 |
| 9241 | cg06236450 |
| 9242 | cg26644715 |
| 9243 | cg03025981 |
| 9244 | cg19534681 |
| 9245 | cg21835874 |
| 9246 | cg05229598 |
| 9247 | cg20299703 |
| 9248 | cg00864293 |
| 9249 | cg05321594 |
| 9250 | cg02976210 |
| 9251 | cg15274245 |
| 9252 | cg13289769 |
| 9253 | cg09011401 |
| 9254 | cg16308270 |
| 9255 | cg26818197 |
| 9256 | cg02848750 |
| 9257 | cg12858577 |
| 9258 | cg10405645 |
| 9259 | cg24624505 |
| 9260 | cg20458338 |
| 9261 | cg21034580 |
| 9262 | cg08275159 |
| 9263 | cg02558992 |
| 9264 | cg05861567 |
| 9265 | cg07796897 |
| 9266 | cg04150136 |
| 9267 | cg24170085 |
| 9268 | cg14706266 |
| 9269 | cg06795419 |
| 9270 | cg14766884 |
| 9271 | cg07607752 |
| 9272 | cg20011134 |
| 9273 | cg22289837 |
| 9274 | cg03033453 |
| 9275 | cg14161589 |
| 9276 | cg21899520 |
| 9277 | cg12148678 |
| 9278 | cg14925764 |
| 9279 | cg18912530 |
| 9280 | cg04594452 |
| 9281 | cg01687558 |
| 9282 | cg07957294 |
| 9283 | cg19642470 |
| 9284 | cg23922743 |
| 9285 | cg14148016 |
| 9286 | cg04561901 |
| 9287 | cg25411977 |
| 9288 | cg27604968 |
| 9289 | cg07812250 |
| 9290 | cg23995914 |
| 9291 | cg22174405 |
| 9292 | cg11021822 |
| 9293 | cg05528872 |
| 9294 | cg06748470 |
| 9295 | cg07149296 |
| 9296 | cg06203364 |
| 9297 | cg02769477 |
| 9298 | cg08662052 |
| 9299 | cg02255488 |
| 9300 | cg25879553 |
| 9301 | cg00548268 |
| 9302 | cg02951847 |
| 9303 | cg22007578 |
| 9304 | cg11201168 |
| 9305 | cg22213247 |
| 9306 | cg03565081 |
| 9307 | cg02446647 |
| 9308 | cg04791291 |
| 9309 | cg25069807 |
| 9310 | cg19819715 |
| 9311 | cg27596890 |
| 9312 | cg19023977 |
| 9313 | cg16828023 |
| 9314 | cg08120226 |
| 9315 | cg11305072 |
| 9316 | cg01315465 |
| 9317 | cg16764061 |
| 9318 | cg18282899 |
| 9319 | cg08549390 |
| 9320 | cg07803108 |
| 9321 | cg11146185 |
| 9322 | cg15331332 |
| 9323 | cg04685101 |
| 9324 | cg02161026 |
| 9325 | cg07734161 |
| 9326 | cg03432955 |
| 9327 | cg02462252 |
| 9328 | cg05904016 |
| 9329 | cg10157902 |
| 9330 | cg19422019 |
| 9331 | cg21031775 |
| 9332 | cg26238800 |
| 9333 | cg17816479 |
| 9334 | cg22976313 |
| 9335 | cg00013688 |
| 9336 | cg06170283 |
| 9337 | cg16935947 |
| 9338 | cg07130623 |
| 9339 | cg02320151 |
| 9340 | cg10706013 |
| 9341 | cg03244009 |
| 9342 | cg03388266 |
| 9343 | cg25258843 |
| 9344 | cg00753835 |
| 9345 | cg06246114 |
| 9346 | cg27659953 |
| 9347 | cg00433917 |
| 9348 | cg24608474 |
| 9349 | cg18189005 |
| 9350 | cg11961243 |
| 9351 | cg22915373 |
| 9352 | cg25045942 |
| 9353 | cg13677990 |
| 9354 | cg22891447 |
| 9355 | cg25308597 |
| 9356 | cg23885809 |
| 9357 | cg05835599 |
| 9358 | cg24583250 |
| 9359 | cg18826274 |
| 9360 | cg17347253 |
| 9361 | cg03171465 |
| 9362 | cg05128414 |
| 9363 | cg00044372 |
| 9364 | cg05886811 |
| 9365 | cg01253087 |
| 9366 | cg04064828 |
| 9367 | cg19279379 |
| 9368 | cg03845718 |
| 9369 | cg12763838 |
| 9370 | cg15802673 |
| 9371 | cg12995043 |
| 9372 | cg03310874 |
| 9373 | cg16949001 |
| 9374 | cg27571747 |
| 9375 | cg25951430 |
| 9376 | cg15010254 |
| 9377 | cg25123470 |
| 9378 | cg08393937 |
| 9379 | cg11661281 |
| 9380 | cg06584478 |
| 9381 | cg04827341 |
| 9382 | cg09473613 |
| 9383 | cg10131972 |
| 9384 | cg16247085 |
| 9385 | cg17929951 |
| 9386 | cg10040951 |
| 9387 | cg05813818 |
| 9388 | cg17949727 |
| 9389 | cg10062196 |
| 9390 | cg23302884 |
| 9391 | cg16124267 |
| 9392 | cg00656603 |
| 9393 | cg20636351 |
| 9394 | cg25762282 |
| 9395 | cg07718431 |
| 9396 | cg13723879 |
| 9397 | cg01187156 |
| 9398 | cg08238215 |
| 9399 | cg10517293 |
| 9400 | cg15811371 |
| 9401 | cg09965661 |
| 9402 | cg17874684 |
| 9403 | cg00044103 |
| 9404 | cg01552711 |
| 9405 | cg17551544 |
| 9406 | cg20875821 |
| 9407 | cg03040092 |
| 9408 | cg14388901 |
| 9409 | cg27534599 |
| 9410 | cg10455196 |
| 9411 | cg26677584 |
| 9412 | cg14774445 |
| 9413 | cg02640497 |
| 9414 | cg03115048 |
| 9415 | cg25704036 |
| 9416 | cg13690590 |
| 9417 | cg22484737 |
| 9418 | cg07793760 |
| 9419 | cg15876825 |
| 9420 | cg11323506 |
| 9421 | cg02303747 |
| 9422 | cg13944018 |
| 9423 | cg04672418 |
| 9424 | cg06267617 |
| 9425 | cg12793116 |
| 9426 | cg26843612 |
| 9427 | cg17775490 |
| 9428 | cg08608240 |
| 9429 | cg02049672 |
| 9430 | cg23454003 |
| 9431 | cg14972576 |
| 9432 | cg23807718 |
| 9433 | cg00231528 |
| 9434 | cg23777599 |
| 9435 | cg16230533 |
| 9436 | cg11589880 |
| 9437 | cg12394010 |
| 9438 | cg13583895 |
| 9439 | cg26031036 |
| 9440 | cg11926119 |
| 9441 | cg17071301 |
| 9442 | cg20304287 |
| 9443 | cg13168042 |
| 9444 | cg21517055 |
| 9445 | cg17763354 |
| 9446 | cg03503516 |
| 9447 | cg15415136 |
| 9448 | cg00926400 |
| 9449 | cg17741014 |
| 9450 | cg02112154 |
| 9451 | cg01814130 |
| 9452 | cg23336695 |
| 9453 | cg03738444 |
| 9454 | cg24440302 |
| 9455 | cg19563307 |
| 9456 | cg07304315 |
| 9457 | cg02898293 |
| 9458 | cg25841380 |
| 9459 | cg19930657 |
| 9460 | cg17945962 |
| 9461 | cg18297745 |
| 9462 | cg02764206 |
| 9463 | cg17772140 |
| 9464 | cg25436886 |
| 9465 | cg21440166 |
| 9466 | cg01435643 |
| 9467 | cg24629311 |
| 9468 | cg19886777 |
| 9469 | cg18628732 |
| 9470 | cg00128324 |
| 9471 | cg10431713 |
| 9472 | cg14936520 |
| 9473 | cg09941713 |
| 9474 | cg02139586 |
| 9475 | cg15221245 |
| 9476 | cg20814095 |
| 9477 | cg12251227 |
| 9478 | cg10439246 |
| 9479 | cg11716106 |
| 9480 | cg16217445 |
| 9481 | cg27113326 |
| 9482 | cg14444715 |
| 9483 | cg19241878 |
| 9484 | cg02542684 |
| 9485 | cg23266773 |
| 9486 | cg06423211 |
| 9487 | cg24376689 |
| 9488 | cg14315019 |
| 9489 | cg12695122 |
| 9490 | cg19590717 |
| 9491 | cg23920642 |
| 9492 | cg04995095 |
| 9493 | cg26020982 |
| 9494 | cg13322237 |
| 9495 | cg02964385 |
| 9496 | cg02293118 |
| 9497 | cg22076873 |
| 9498 | cg10985909 |
| 9499 | cg18147098 |
| 9500 | cg14049075 |
| 9501 | cg14744898 |
| 9502 | cg18499684 |
| 9503 | cg02723395 |
| 9504 | cg04004590 |
| 9505 | cg02390801 |
| 9506 | cg14356843 |
| 9507 | cg06652313 |
| 9508 | cg05164933 |
| 9509 | cg11584098 |
| 9510 | cg15174692 |
| 9511 | cg12310411 |
| 9512 | cg12469902 |
| 9513 | cg07990390 |
| 9514 | cg00154543 |
| 9515 | cg18336854 |
| 9516 | cg04554158 |
| 9517 | cg06570432 |
| 9518 | cg13707002 |
| 9519 | cg11955276 |
| 9520 | cg15886317 |
| 9521 | cg26193427 |
| 9522 | cg17236709 |
| 9523 | cg18431100 |
| 9524 | cg10039504 |
| 9525 | cg20293689 |
| 9526 | cg00370106 |
| 9527 | cg05405978 |
| 9528 | cg14375499 |
| 9529 | cg17197030 |
| 9530 | cg08489309 |
| 9531 | cg13944085 |
| 9532 | cg18531916 |
| 9533 | cg00498211 |
| 9534 | cg04933914 |
| 9535 | cg01611777 |
| 9536 | cg23994292 |
| 9537 | cg11149163 |
| 9538 | cg21238257 |
| 9539 | cg17573913 |
| 9540 | cg02139338 |
| 9541 | cg00674675 |
| 9542 | cg13099276 |
| 9543 | cg02743146 |
| 9544 | cg06271970 |
| 9545 | cg22685816 |
| 9546 | cg01102201 |
| 9547 | cg15449956 |
| 9548 | cg20601028 |
| 9549 | cg18753646 |
| 9550 | cg09176441 |
| 9551 | cg03687532 |
| 9552 | cg07881032 |
| 9553 | cg01166876 |
| 9554 | cg07179753 |
| 9555 | cg27610561 |
| 9556 | cg18462631 |
| 9557 | cg02453828 |
| 9558 | cg11444332 |
| 9559 | cg00772991 |
| 9560 | cg18322693 |
| 9561 | cg10767223 |
| 9562 | cg16749456 |
| 9563 | cg27181315 |
| 9564 | cg10802101 |
| 9565 | cg07214466 |
| 9566 | cg04673465 |
| 9567 | cg05932992 |
| 9568 | cg02461114 |
| 9569 | cg06371967 |
| 9570 | cg00684125 |
| 9571 | cg14629771 |
| 9572 | cg03043804 |
| 9573 | cg26681628 |
| 9574 | cg14839340 |
| 9575 | cg07100050 |
| 9576 | cg05089465 |
| 9577 | cg07935902 |
| 9578 | cg23824183 |
| 9579 | cg22860740 |
| 9580 | cg21827277 |
| 9581 | cg12150256 |
| 9582 | cg04670206 |
| 9583 | cg26360697 |
| 9584 | cg02712700 |
| 9585 | cg00548429 |
| 9586 | cg14607332 |
| 9587 | cg03739434 |
| 9588 | cg01642550 |
| 9589 | cg03368099 |
| 9590 | cg03427322 |
| 9591 | cg13209113 |
| 9592 | cg21919809 |
| 9593 | cg00823843 |
| 9594 | cg06287897 |
| 9595 | cg20577027 |
| 9596 | cg26627769 |
| 9597 | cg14297707 |
| 9598 | cg09003833 |
| 9599 | cg19087116 |
| 9600 | cg20950217 |
| 9601 | cg00084153 |
| 9602 | cg02701080 |
| 9603 | cg00142402 |
| 9604 | cg23564931 |
| 9605 | cg15909951 |
| 9606 | cg09498572 |
| 9607 | cg01181307 |
| 9608 | cg27410158 |
| 9609 | cg13629304 |
| 9610 | cg16450097 |
| 9611 | cg19634247 |
| 9612 | cg23369564 |
| 9613 | cg08641881 |
| 9614 | cg14368211 |
| 9615 | cg17422603 |
| 9616 | cg06155710 |
| 9617 | cg06368300 |
| 9618 | cg25553170 |
| 9619 | cg08732352 |
| 9620 | cg16246347 |
| 9621 | cg12743970 |
| 9622 | cg21871091 |
| 9623 | cg14532892 |
| 9624 | cg27417609 |
| 9625 | cg08111922 |
| 9626 | cg10836779 |
| 9627 | cg24545495 |
| 9628 | cg03672274 |
| 9629 | cg01460202 |
| 9630 | cg20109867 |
| 9631 | cg24454158 |
| 9632 | cg07875146 |
| 9633 | cg10897384 |
| 9634 | cg03819785 |
| 9635 | cg10213542 |
| 9636 | cg04180046 |
| 9637 | cg13871948 |
| 9638 | cg24271393 |
| 9639 | cg16362027 |
| 9640 | cg12515103 |
| 9641 | cg23916360 |
| 9642 | cg24693436 |
| 9643 | cg09288218 |
| 9644 | cg15417547 |
| 9645 | cg22570405 |
| 9646 | cg11074044 |
| 9647 | cg24998347 |
| 9648 | cg23120013 |
| 9649 | cg12816995 |
| 9650 | cg22928082 |
| 9651 | cg05704547 |
| 9652 | cg21103949 |
| 9653 | cg20899781 |
| 9654 | cg09424398 |
| 9655 | cg02770672 |
| 9656 | cg26269162 |
| 9657 | cg25261468 |
| 9658 | cg02747950 |
| 9659 | cg08886711 |
| 9660 | cg13185878 |
| 9661 | cg19636861 |
| 9662 | cg09294573 |
| 9663 | cg27519981 |
| 9664 | cg26185508 |
| 9665 | cg26674160 |
| 9666 | cg09626521 |
| 9667 | cg14794762 |
| 9668 | cg02361878 |
| 9669 | cg13684305 |
| 9670 | cg00310940 |
| 9671 | cg15226275 |
| 9672 | cg22758895 |
| 9673 | cg08511485 |
| 9674 | cg06935979 |
| 9675 | cg17312152 |
| 9676 | cg12559119 |
| 9677 | cg12127282 |
| 9678 | cg02611744 |
| 9679 | cg25687358 |
| 9680 | cg09834465 |
| 9681 | cg09668627 |
| 9682 | cg05846378 |
| 9683 | cg12431052 |
| 9684 | cg06681768 |
| 9685 | cg26314722 |
| 9686 | cg17012682 |
| 9687 | cg14016170 |
| 9688 | cg24954070 |
| 9689 | cg00867317 |
| 9690 | cg27630316 |
| 9691 | cg19420069 |
| 9692 | cg14215970 |
| 9693 | cg05794117 |
| 9694 | cg21380590 |
| 9695 | cg01612247 |
| 9696 | cg07524540 |
| 9697 | cg23856718 |
| 9698 | cg06960461 |
| 9699 | cg10106388 |
| 9700 | cg23933241 |
| 9701 | cg04602516 |
| 9702 | cg08365738 |
| 9703 | cg14280181 |
| 9704 | cg22080940 |
| 9705 | cg26149485 |
| 9706 | cg24610630 |
| 9707 | cg08833509 |
| 9708 | cg01456969 |
| 9709 | cg27024844 |
| 9710 | cg15025103 |
| 9711 | cg02668655 |
| 9712 | cg03706283 |
| 9713 | cg05274680 |
| 9714 | cg00283535 |
| 9715 | cg17786845 |
| 9716 | cg05950113 |
| 9717 | cg18651858 |
| 9718 | cg06728274 |
| 9719 | cg10014986 |
| 9720 | cg15051463 |
| 9721 | cg14854532 |
| 9722 | cg18397998 |
| 9723 | cg18876826 |
| 9724 | cg04124281 |
| 9725 | cg15586699 |
| 9726 | cg01704698 |
| 9727 | cg05035508 |
| 9728 | cg27177158 |
| 9729 | cg19944763 |
| 9730 | cg10093938 |
| 9731 | cg25301532 |
| 9732 | cg01937819 |
| 9733 | cg16152136 |
| 9734 | cg13671732 |
| 9735 | cg04384543 |
| 9736 | cg24452451 |
| 9737 | cg22337491 |
| 9738 | cg24269657 |
| 9739 | cg13209171 |
| 9740 | cg22088344 |
| 9741 | cg10848640 |
| 9742 | cg02483701 |
| 9743 | cg12542842 |
| 9744 | cg19537443 |
| 9745 | cg11015185 |
| 9746 | cg19795817 |
| 9747 | cg16049690 |
| 9748 | cg22749859 |
| 9749 | cg22161303 |
| 9750 | cg14738806 |
| 9751 | cg21386573 |
| 9752 | cg14755019 |
| 9753 | cg10622551 |
| 9754 | cg26221442 |
| 9755 | cg01205267 |
| 9756 | cg12736877 |
| 9757 | cg18731860 |
| 9758 | cg02619037 |
| 9759 | cg03231735 |
| 9760 | cg26573707 |
| 9761 | cg03345116 |
| 9762 | cg11489945 |
| 9763 | cg07073801 |
| 9764 | cg19859957 |
| 9765 | cg18413131 |
| 9766 | cg03081691 |
| 9767 | cg24528392 |
| 9768 | cg17637937 |
| 9769 | cg00043076 |
| 9770 | cg20000866 |
| 9771 | cg26932455 |
| 9772 | cg21154229 |
| 9773 | cg10625097 |
| 9774 | cg13786031 |
| 9775 | cg14081744 |
| 9776 | cg25360385 |
| 9777 | cg14736058 |
| 9778 | cg08340737 |
| 9779 | cg08474478 |
| 9780 | cg02677609 |
| 9781 | cg15108521 |
| 9782 | cg14512563 |
| 9783 | cg22999892 |
| 9784 | cg05178333 |
| 9785 | cg14259717 |
| 9786 | cg26418881 |
| 9787 | cg21550639 |
| 9788 | cg19492190 |
| 9789 | cg14417948 |
| 9790 | cg17028595 |
| 9791 | cg16134349 |
| 9792 | cg26164879 |
| 9793 | cg09508730 |
| 9794 | cg21560076 |
| 9795 | cg00329080 |
| 9796 | cg18923721 |
| 9797 | cg02200032 |
| 9798 | cg21890448 |
| 9799 | cg11787167 |
| 9800 | cg06348275 |
| 9801 | cg14675598 |
| 9802 | cg09802076 |
| 9803 | cg13416517 |
| 9804 | cg09409865 |
| 9805 | cg09024069 |
| 9806 | cg14317881 |
| 9807 | cg09771102 |
| 9808 | cg03531140 |
| 9809 | cg18538872 |
| 9810 | cg15758896 |
| 9811 | cg06759874 |
| 9812 | cg16288880 |
| 9813 | cg04101019 |
| 9814 | cg10935362 |
| 9815 | cg01646508 |
| 9816 | cg27543201 |
| 9817 | cg20111217 |
| 9818 | cg06251304 |
| 9819 | cg07633666 |
| 9820 | cg23223933 |
| 9821 | cg15607161 |
| 9822 | cg04198914 |
| 9823 | cg07403350 |
| 9824 | cg07086492 |
| 9825 | cg26603183 |
| 9826 | cg12033692 |
| 9827 | cg10670256 |
| 9828 | cg25786036 |
| 9829 | cg18222853 |
| 9830 | cg07836142 |
| 9831 | cg03662858 |
| 9832 | cg22328208 |
| 9833 | cg25264268 |
| 9834 | cg17109198 |
| 9835 | cg04361443 |
| 9836 | cg09803321 |
| 9837 | cg25731211 |
| 9838 | cg14130459 |
| 9839 | cg16784985 |
| 9840 | cg27243353 |
| 9841 | cg14276466 |
| 9842 | cg07186962 |
| 9843 | cg17256364 |
| 9844 | cg25012795 |
| 9845 | cg09409539 |
| 9846 | cg06430753 |
| 9847 | cg20727798 |
| 9848 | cg05279229 |
| 9849 | cg06215984 |
| 9850 | cg09906813 |
| 9851 | cg15159083 |
| 9852 | cg04470072 |
| 9853 | cg03217690 |
| 9854 | cg03845745 |
| 9855 | cg06570358 |
| 9856 | cg19635805 |
| 9857 | cg06036947 |
| 9858 | cg15457934 |
| 9859 | cg14076594 |
| 9860 | cg11902641 |
| 9861 | cg26354493 |
| 9862 | cg10800095 |
| 9863 | cg00399069 |
| 9864 | cg22720745 |
| 9865 | cg00340179 |
| 9866 | cg11999598 |
| 9867 | cg25195673 |
| 9868 | cg19507206 |
| 9869 | cg12293184 |
| 9870 | cg16548605 |
| 9871 | cg14565151 |
| 9872 | cg17720153 |
| 9873 | cg27633782 |
| 9874 | cg08743681 |
| 9875 | cg27093637 |
| 9876 | cg06379192 |
| 9877 | cg23854572 |
| 9878 | cg11200797 |
| 9879 | cg01566186 |
| 9880 | cg20904489 |
| 9881 | cg14812524 |
| 9882 | cg12887033 |
| 9883 | cg27336392 |
| 9884 | cg21488633 |
| 9885 | cg24796998 |
| 9886 | cg24458712 |
| 9887 | cg24880736 |
| 9888 | cg00508855 |
| 9889 | cg01759218 |
| 9890 | cg05845403 |
| 9891 | cg26152017 |
| 9892 | cg24418762 |
| 9893 | cg12280471 |
| 9894 | cg15589812 |
| 9895 | cg03773198 |
| 9896 | cg04932937 |
| 9897 | cg23346933 |
| 9898 | cg06902067 |
| 9899 | cg00249374 |
| 9900 | cg19519874 |
| 9901 | cg12211397 |
| 9902 | cg24780509 |
| 9903 | cg06443533 |
| 9904 | cg00132255 |
| 9905 | cg17516809 |
| 9906 | cg27307087 |
| 9907 | cg04340651 |
| 9908 | cg22418837 |
| 9909 | cg11752250 |
| 9910 | cg22139335 |
| 9911 | cg25600446 |
| 9912 | cg12671030 |
| 9913 | cg09881191 |
| 9914 | cg17659636 |
| 9915 | cg14508791 |
| 9916 | cg00035636 |
| 9917 | cg14385362 |
| 9918 | cg16496782 |
| 9919 | cg12226009 |
| 9920 | cg16294013 |
| 9921 | cg05200811 |
| 9922 | cg21817858 |
| 9923 | cg09440559 |
| 9924 | cg21072040 |
| 9925 | cg08629884 |
| 9926 | cg01342115 |
| 9927 | cg15007983 |
| 9928 | cg01986081 |
| 9929 | cg21281463 |
| 9930 | cg14864895 |
| 9931 | cg07293736 |
| 9932 | cg24591770 |
| 9933 | cg17113932 |
| 9934 | cg06976770 |
| 9935 | cg08126875 |
| 9936 | cg12672189 |
| 9937 | cg02546690 |
| 9938 | cg06007201 |
| 9939 | cg07548236 |
| 9940 | cg17669536 |
| 9941 | cg03022836 |
| 9942 | cg12859507 |
| 9943 | cg08297393 |
| 9944 | cg17477052 |
| 9945 | cg04569877 |
| 9946 | cg02075958 |
| 9947 | cg01923037 |
| 9948 | cg10005305 |
| 9949 | cg03379552 |
| 9950 | cg11100157 |
| 9951 | cg22549540 |
| 9952 | cg22253309 |
| 9953 | cg26248066 |
| 9954 | cg11636765 |
| 9955 | cg13406893 |
| 9956 | cg25749512 |
| 9957 | cg18563970 |
| 9958 | cg27140633 |
| 9959 | cg13962718 |
| 9960 | cg06617202 |
| 9961 | cg00624885 |
| 9962 | cg21640221 |
| 9963 | cg07520506 |
| 9964 | cg00331518 |
| 9965 | cg10725542 |
| 9966 | cg05983231 |
| 9967 | cg18611360 |
| 9968 | cg01907512 |
| 9969 | cg15989981 |
| 9970 | cg20537629 |
| 9971 | cg20873866 |
| 9972 | cg26348414 |
| 9973 | cg21434376 |
| 9974 | cg12960401 |
| 9975 | cg06531475 |
| 9976 | cg13933262 |
| 9977 | cg06238004 |
| 9978 | cg10580960 |
| 9979 | cg14549634 |
| 9980 | cg14021398 |
| 9981 | cg13285894 |
| 9982 | cg16952928 |
| 9983 | cg04485799 |
| 9984 | cg00126638 |
| 9985 | cg07187576 |
| 9986 | cg11879188 |
| 9987 | cg19711100 |
| 9988 | cg01234697 |
| 9989 | cg10894156 |
| 9990 | cg20732869 |
| 9991 | cg22329579 |
| 9992 | cg18163442 |
| 9993 | cg22914993 |
| 9994 | cg07415610 |
| 9995 | cg09842118 |
| 9996 | cg17360854 |
| 9997 | cg02586483 |
| 9998 | cg22472941 |
| 9999 | cg26284520 |
| 10000 | cg22003274 |
| 10001 | cg12900231 |
| 10002 | cg20399509 |
| 10003 | cg14827440 |
| 10004 | cg02287214 |
| 10005 | cg26589036 |
| 10006 | cg08417873 |
| 10007 | cg17833169 |
| 10008 | cg22434730 |
| 10009 | cg21331770 |
| 10010 | cg07369084 |
| 10011 | cg05024003 |
| 10012 | cg18618815 |
| 10013 | cg09504320 |
| 10014 | cg02671171 |
| 10015 | cg03991300 |
| 10016 | cg06800829 |
| 10017 | cg16018995 |
| 10018 | cg08453458 |
| 10019 | cg08553467 |
| 10020 | cg13937462 |
| 10021 | cg12407708 |
| 10022 | cg09307985 |
| 10023 | cg14433849 |
| 10024 | cg12516720 |
| 10025 | cg23275355 |
| 10026 | cg04091682 |
| 10027 | cg01116137 |
| 10028 | cg10925265 |
| 10029 | cg20977794 |
| 10030 | cg15628724 |
| 10031 | cg23611697 |
| 10032 | cg12492105 |
| 10033 | cg23741939 |
| 10034 | cg01860091 |
| 10035 | cg22932313 |
| 10036 | cg22846582 |
| 10037 | cg12325700 |
| 10038 | cg17180237 |
| 10039 | cg14368930 |
| 10040 | cg10138882 |
| 10041 | cg12176682 |
| 10042 | cg02770628 |
| 10043 | cg05290820 |
| 10044 | cg26175448 |
| 10045 | cg18846139 |
| 10046 | cg15975980 |
| 10047 | cg17364913 |
| 10048 | cg00376544 |
| 10049 | cg04471251 |
| 10050 | cg04997467 |
| 10051 | cg18161670 |
| 10052 | cg24970360 |
| 10053 | cg02032558 |
| 10054 | cg06382345 |
| 10055 | cg01570297 |
| 10056 | cg17457029 |
| 10057 | cg02086457 |
| 10058 | cg05579187 |
| 10059 | cg07533345 |
| 10060 | cg13257436 |
| 10061 | cg17887794 |
| 10062 | cg06975325 |
| 10063 | cg21468426 |
| 10064 | cg12072740 |
| 10065 | cg27123859 |
| 10066 | cg22238977 |
| 10067 | cg01649219 |
| 10068 | cg14124310 |
| 10069 | cg18768862 |
| 10070 | cg00015801 |
| 10071 | cg00991875 |
| 10072 | cg00892742 |
| 10073 | cg21262776 |
| 10074 | cg11723960 |
| 10075 | cg17136885 |
| 10076 | cg14159342 |
| 10077 | cg21160679 |
| 10078 | cg02387779 |
| 10079 | cg02501779 |
| 10080 | cg27102378 |
| 10081 | cg07054641 |
| 10082 | cg25164391 |
| 10083 | cg12660963 |
| 10084 | cg25176297 |
| 10085 | cg25721648 |
| 10086 | cg01423773 |
| 10087 | cg14685822 |
| 10088 | cg22774340 |
| 10089 | cg26195482 |
| 10090 | cg11792470 |
| 10091 | cg01476710 |
| 10092 | cg04453829 |
| 10093 | cg17328839 |
| 10094 | cg02387660 |
| 10095 | cg08674813 |
| 10096 | cg00885904 |
| 10097 | cg15471388 |
| 10098 | cg27621870 |
| 10099 | cg00319692 |
| 10100 | cg22730004 |
| 10101 | cg01403748 |
| 10102 | cg23982489 |
| 10103 | cg05509190 |
| 10104 | cg19103219 |
| 10105 | cg17720013 |
| 10106 | cg14699932 |
| 10107 | cg00513205 |
| 10108 | cg07661899 |
| 10109 | cg07035299 |
| 10110 | cg27278032 |
| 10111 | cg25497484 |
| 10112 | cg15260465 |
| 10113 | cg15436123 |
| 10114 | cg20801476 |
| 10115 | cg01359822 |
| 10116 | cg16622403 |
| 10117 | cg03734133 |
| 10118 | cg09284949 |
| 10119 | cg20983265 |
| 10120 | cg10376731 |
| 10121 | cg06095304 |
| 10122 | cg17509563 |
| 10123 | cg01782798 |
| 10124 | cg01246599 |
| 10125 | cg12964457 |
| 10126 | cg26518391 |
| 10127 | cg01618938 |
| 10128 | cg12510530 |
| 10129 | cg00916884 |
| 10130 | cg03490971 |
| 10131 | cg13939462 |
| 10132 | cg21193909 |
| 10133 | cg26876834 |
| 10134 | cg08946144 |
| 10135 | cg17416793 |
| 10136 | cg00093095 |
| 10137 | cg17738861 |
| 10138 | cg13840922 |
| 10139 | cg09656934 |
| 10140 | cg03685439 |
| 10141 | cg19220282 |
| 10142 | cg13071333 |
| 10143 | cg12541935 |
| 10144 | cg05408203 |
| 10145 | cg05909365 |
| 10146 | cg09249159 |
| 10147 | cg19569526 |
| 10148 | cg24527560 |
| 10149 | cg01397065 |
| 10150 | cg14097517 |
| 10151 | cg26735544 |
| 10152 | cg01368217 |
| 10153 | cg09117588 |
| 10154 | cg23552088 |
| 10155 | cg09432376 |
| 10156 | cg11242444 |
| 10157 | cg22655956 |
| 10158 | cg22708902 |
| 10159 | cg00717259 |
| 10160 | cg27217253 |
| 10161 | cg06934762 |
| 10162 | cg07545846 |
| 10163 | cg10775231 |
| 10164 | cg08247564 |
| 10165 | cg01906944 |
| 10166 | cg25258033 |
| 10167 | cg17221226 |
| 10168 | cg08128768 |
| 10169 | cg12193201 |
| 10170 | cg03760483 |
| 10171 | cg18294631 |
| 10172 | cg00610508 |
| 10173 | cg09622651 |
| 10174 | cg16551520 |
| 10175 | cg09503816 |
| 10176 | cg26172504 |
| 10177 | cg00548285 |
| 10178 | cg06667094 |
| 10179 | cg12676421 |
| 10180 | cg17334468 |
| 10181 | cg23739048 |
| 10182 | cg27527924 |
| 10183 | cg25290307 |
| 10184 | cg15404483 |
| 10185 | cg24651312 |
| 10186 | cg25835611 |
| 10187 | cg25433586 |
| 10188 | cg06893785 |
| 10189 | cg24574793 |
| 10190 | cg19928703 |
| 10191 | cg00599564 |
| 10192 | cg02938414 |
| 10193 | cg04992771 |
| 10194 | cg12840873 |
| 10195 | cg08618219 |
| 10196 | cg10138206 |
| 10197 | cg03859590 |
| 10198 | cg03615426 |
| 10199 | cg19166896 |
| 10200 | cg20940250 |
| 10201 | cg07014416 |
| 10202 | cg22602379 |
| 10203 | cg04308185 |
| 10204 | cg08508845 |
| 10205 | cg24694549 |
| 10206 | cg11139691 |
| 10207 | cg05495790 |
| 10208 | cg25025437 |
| 10209 | cg26820032 |
| 10210 | cg14854358 |
| 10211 | cg02733241 |
| 10212 | cg06052688 |
| 10213 | cg00998686 |
| 10214 | cg18877506 |
| 10215 | cg14316629 |
| 10216 | cg19081101 |
| 10217 | cg19594772 |
| 10218 | cg04173760 |
| 10219 | cg18779693 |
| 10220 | cg24094897 |
| 10221 | cg08912468 |
| 10222 | cg11992241 |
| 10223 | cg18625627 |
| 10224 | cg02612332 |
| 10225 | cg24702341 |
| 10226 | cg02423065 |
| 10227 | cg15442338 |
| 10228 | cg17511472 |
| 10229 | cg13274692 |
| 10230 | cg22363368 |
| 10231 | cg03953088 |
| 10232 | cg04598121 |
| 10233 | cg00569091 |
| 10234 | cg10602248 |
| 10235 | cg07638857 |
| 10236 | cg27035886 |
| 10237 | cg06818207 |
| 10238 | cg26407715 |
| 10239 | cg01881182 |
| 10240 | cg06554744 |
| 10241 | cg25261151 |
| 10242 | cg26262055 |
| 10243 | cg03052301 |
| 10244 | cg14251134 |
| 10245 | cg23942526 |
| 10246 | cg27518156 |
| 10247 | cg15332782 |
| 10248 | cg04067940 |
| 10249 | cg03882612 |
| 10250 | cg17758878 |
| 10251 | cg01531398 |
| 10252 | cg02140979 |
| 10253 | cg09912824 |
| 10254 | cg02647408 |
| 10255 | cg21188664 |
| 10256 | cg08940075 |
| 10257 | cg27628707 |
| 10258 | cg24721964 |
| 10259 | cg11710851 |
| 10260 | cg00809267 |
| 10261 | cg11312495 |
| 10262 | cg06130683 |
| 10263 | cg08879910 |
| 10264 | cg00147244 |
| 10265 | cg03094242 |
| 10266 | cg09199562 |
| 10267 | cg13802583 |
| 10268 | cg11034978 |
| 10269 | cg18238078 |
| 10270 | cg18384219 |
| 10271 | cg04094178 |
| 10272 | cg24729364 |
| 10273 | cg05701235 |
| 10274 | cg15132183 |
| 10275 | cg01560057 |
| 10276 | cg13266435 |
| 10277 | cg13683361 |
| 10278 | cg11387901 |
| 10279 | cg16854130 |
| 10280 | cg24710048 |
| 10281 | cg10977795 |
| 10282 | cg05454501 |
| 10283 | cg10362475 |
| 10284 | cg07108648 |
| 10285 | cg13068215 |
| 10286 | cg11440486 |
| 10287 | cg06398335 |
| 10288 | cg03560743 |
| 10289 | cg20421623 |
| 10290 | cg05681383 |
| 10291 | cg19698137 |
| 10292 | cg02415051 |
| 10293 | cg15087683 |
| 10294 | cg12533689 |
| 10295 | cg14277923 |
| 10296 | cg12428229 |
| 10297 | cg06398236 |
| 10298 | cg17421060 |
| 10299 | cg10372121 |
| 10300 | cg02999390 |
| 10301 | cg24642151 |
| 10302 | cg26935976 |
| 10303 | cg14864148 |
| 10304 | cg06686895 |
| 10305 | cg10426234 |
| 10306 | cg20806725 |
| 10307 | cg05651243 |
| 10308 | cg06784539 |
| 10309 | cg24276374 |
| 10310 | cg22948672 |
| 10311 | cg06498272 |
| 10312 | cg10069904 |
| 10313 | cg16047144 |
| 10314 | cg23276120 |
| 10315 | cg19059954 |
| 10316 | cg14729368 |
| 10317 | cg23438553 |
| 10318 | cg04074319 |
| 10319 | cg11218842 |
| 10320 | cg04494873 |
| 10321 | cg04910921 |
| 10322 | cg06091596 |
| 10323 | cg25075375 |
| 10324 | cg21447477 |
| 10325 | cg09306332 |
| 10326 | cg14666946 |
| 10327 | cg12751644 |
| 10328 | cg16596440 |
| 10329 | cg01739354 |
| 10330 | cg18002862 |
| 10331 | cg02024445 |
| 10332 | cg11241851 |
| 10333 | cg06137852 |
| 10334 | cg06347083 |
| 10335 | cg17334453 |
| 10336 | cg23702727 |
| 10337 | cg14634607 |
| 10338 | cg24796554 |
| 10339 | cg27328431 |
| 10340 | cg11743829 |
| 10341 | cg07520163 |
| 10342 | cg12435074 |
| 10343 | cg13841463 |
| 10344 | cg11945895 |
| 10345 | cg16602747 |
| 10346 | cg16688899 |
| 10347 | cg15198751 |
| 10348 | cg06041067 |
| 10349 | cg10088359 |
| 10350 | cg09224452 |
| 10351 | cg12881363 |
| 10352 | cg16121384 |
| 10353 | cg03502601 |
| 10354 | cg12552289 |
| 10355 | cg11514511 |
| 10356 | cg09187505 |
| 10357 | cg21733531 |
| 10358 | cg02246055 |
| 10359 | cg20623690 |
| 10360 | cg24339704 |
| 10361 | cg02205443 |
| 10362 | cg26986629 |
| 10363 | cg09391860 |
| 10364 | cg17444561 |
| 10365 | cg25954397 |
| 10366 | cg23709898 |
| 10367 | cg00682096 |
| 10368 | cg14891200 |
| 10369 | cg18989909 |
| 10370 | cg24236935 |
| 10371 | cg05860426 |
| 10372 | cg19245011 |
| 10373 | cg13372562 |
| 10374 | cg17163168 |
| 10375 | cg26683025 |
| 10376 | cg16224936 |
| 10377 | cg18935475 |
| 10378 | cg00736299 |
| 10379 | cg09535437 |
| 10380 | cg03727673 |
| 10381 | cg16465280 |
| 10382 | cg04548162 |
| 10383 | cg27435133 |
| 10384 | cg15794228 |
| 10385 | cg02970458 |
| 10386 | cg16096646 |
| 10387 | cg22697962 |
| 10388 | cg13368877 |
| 10389 | cg15511286 |
| 10390 | cg15545733 |
| 10391 | cg21940220 |
| 10392 | cg07673807 |
| 10393 | cg11391665 |
| 10394 | cg10572054 |
| 10395 | cg25206536 |
| 10396 | cg09236126 |
| 10397 | cg13050240 |
| 10398 | cg03131126 |
| 10399 | cg09827833 |
| 10400 | cg00777546 |
| 10401 | cg12004787 |
| 10402 | cg03363478 |
| 10403 | cg23459573 |
| 10404 | cg11879548 |
| 10405 | cg01778935 |
| 10406 | cg16008620 |
| 10407 | cg00142106 |
| 10408 | cg22063847 |
| 10409 | cg17758575 |
| 10410 | cg17249170 |
| 10411 | cg21270847 |
| 10412 | cg03242265 |
| 10413 | cg24229701 |
| 10414 | cg25665749 |
| 10415 | cg01613464 |
| 10416 | cg05188959 |
| 10417 | cg02397368 |
| 10418 | cg25761931 |
| 10419 | cg03206359 |
| 10420 | cg19668696 |
| 10421 | cg15655676 |
| 10422 | cg13407715 |
| 10423 | cg13261883 |
| 10424 | cg08333283 |
| 10425 | cg01256665 |
| 10426 | cg07267547 |
| 10427 | cg04763290 |
| 10428 | cg04233669 |
| 10429 | cg14229540 |
| 10430 | cg25840608 |
| 10431 | cg12796285 |
| 10432 | cg07094049 |
| 10433 | cg17523555 |
| 10434 | cg14534279 |
| 10435 | cg15086607 |
| 10436 | cg16616467 |
| 10437 | cg15510895 |
| 10438 | cg22409100 |
| 10439 | cg00014467 |
| 10440 | cg20051117 |
| 10441 | cg04340430 |
| 10442 | cg19791409 |
| 10443 | cg14946913 |
| 10444 | cg25526173 |
| 10445 | cg19412791 |
| 10446 | cg00467292 |
| 10447 | cg00184457 |
| 10448 | cg09002358 |
| 10449 | cg03053738 |
| 10450 | cg16228323 |
| 10451 | cg10012273 |
| 10452 | cg25005368 |
| 10453 | cg00259083 |
| 10454 | cg19922825 |
| 10455 | cg24005407 |
| 10456 | cg24025012 |
| 10457 | cg05142520 |
| 10458 | cg06301550 |
| 10459 | cg13687570 |
| 10460 | cg23012609 |
| 10461 | cg16541998 |
| 10462 | cg00186815 |
| 10463 | cg01281904 |
| 10464 | cg12600184 |
| 10465 | cg13679679 |
| 10466 | cg20716202 |
| 10467 | cg00616172 |
| 10468 | cg27375331 |
| 10469 | cg27286626 |
| 10470 | cg06158471 |
| 10471 | cg16867817 |
| 10472 | cg12664173 |
| 10473 | cg10407744 |
| 10474 | cg08754124 |
| 10475 | cg07137108 |
| 10476 | cg16886987 |
| 10477 | cg25773163 |
| 10478 | cg08091806 |
| 10479 | cg12655260 |
| 10480 | cg21865657 |
| 10481 | cg23921113 |
| 10482 | cg15910690 |
| 10483 | cg03158949 |
| 10484 | cg06049452 |
| 10485 | cg21539981 |
| 10486 | cg06161566 |
| 10487 | cg13992954 |
| 10488 | cg27181471 |
| 10489 | cg07425204 |
| 10490 | cg26715042 |
| 10491 | cg06384827 |
| 10492 | cg05632088 |
| 10493 | cg06868115 |
| 10494 | cg07787398 |
| 10495 | cg02868178 |
| 10496 | cg13918518 |
| 10497 | cg14612428 |
| 10498 | cg18785598 |
| 10499 | cg06223162 |
| 10500 | cg17927824 |
| 10501 | cg06318062 |
| 10502 | cg07442907 |
| 10503 | cg19997773 |
| 10504 | cg03751727 |
| 10505 | cg06101324 |
| 10506 | cg01890120 |
| 10507 | cg21482658 |
| 10508 | cg22711111 |
| 10509 | cg04772025 |
| 10510 | cg02071172 |
| 10511 | cg08792812 |
| 10512 | cg26425321 |
| 10513 | cg00242936 |
| 10514 | cg03926784 |
| 10515 | cg03468072 |
| 10516 | cg07120993 |
| 10517 | cg18856386 |
| 10518 | cg11588903 |
| 10519 | cg07085167 |
| 10520 | cg00182893 |
| 10521 | cg02924459 |
| 10522 | cg01219924 |
| 10523 | cg17419888 |
| 10524 | cg18527241 |
| 10525 | cg06856720 |
| 10526 | cg24516362 |
| 10527 | cg20852250 |
| 10528 | cg01373444 |
| 10529 | cg05070262 |
| 10530 | cg21130103 |
| 10531 | cg24434242 |
| 10532 | cg02028297 |
| 10533 | cg03337918 |
| 10534 | cg13931925 |
| 10535 | cg18266383 |
| 10536 | cg00406225 |
| 10537 | cg09734796 |
| 10538 | cg15977864 |
| 10539 | cg13813366 |
| 10540 | cg06872172 |
| 10541 | cg20779757 |
| 10542 | cg08704195 |
| 10543 | cg16981703 |
| 10544 | cg15248119 |
| 10545 | cg08105471 |
| 10546 | cg13294852 |
| 10547 | cg02425511 |
| 10548 | cg18622950 |
| 10549 | cg24199006 |
| 10550 | cg26694831 |
| 10551 | cg16582036 |
| 10552 | cg26086477 |
| 10553 | cg22372129 |
| 10554 | cg04454088 |
| 10555 | cg07046120 |
| 10556 | cg07164791 |
| 10557 | cg25759916 |
| 10558 | cg01268515 |
| 10559 | cg03602119 |
| 10560 | cg10333824 |
| 10561 | cg07700233 |
| 10562 | cg19024700 |
| 10563 | cg20086047 |
| 10564 | cg05447343 |
| 10565 | cg02375166 |
| 10566 | cg06552160 |
| 10567 | cg02646092 |
| 10568 | cg22290782 |
| 10569 | cg02487331 |
| 10570 | cg02303505 |
| 10571 | cg09328921 |
| 10572 | cg12905571 |
| 10573 | cg03906031 |
| 10574 | cg23012855 |
| 10575 | cg25046720 |
| 10576 | cg04790662 |
| 10577 | cg03570035 |
| 10578 | cg16661866 |
| 10579 | cg15924102 |
| 10580 | cg14554371 |
| 10581 | cg22334665 |
| 10582 | cg25961432 |
| 10583 | cg19784907 |
| 10584 | cg10955566 |
| 10585 | cg14911521 |
| 10586 | cg19047660 |
| 10587 | cg00593773 |
| 10588 | cg14859916 |
| 10589 | cg05060175 |
| 10590 | cg25123887 |
| 10591 | cg27261597 |
| 10592 | cg23741906 |
| 10593 | cg08206970 |
| 10594 | cg23346658 |
| 10595 | cg21423387 |
| 10596 | cg05075025 |
| 10597 | cg19823985 |
| 10598 | cg02138322 |
| 10599 | cg14156498 |
| 10600 | cg19718309 |
| 10601 | cg05906144 |
| 10602 | cg10122477 |
| 10603 | cg08757828 |
| 10604 | cg13636014 |
| 10605 | cg10205045 |
| 10606 | cg01231543 |
| 10607 | cg17279839 |
| 10608 | cg27312406 |
| 10609 | cg00378999 |
| 10610 | cg01616586 |
| 10611 | cg13453168 |
| 10612 | cg15295132 |
| 10613 | cg13839160 |
| 10614 | cg24241726 |
| 10615 | cg12899919 |
| 10616 | cg03532904 |
| 10617 | cg13330559 |
| 10618 | cg18825594 |
| 10619 | cg25902939 |
| 10620 | cg14294215 |
| 10621 | cg06496648 |
| 10622 | cg23001785 |
| 10623 | cg09578379 |
| 10624 | cg14938419 |
| 10625 | cg18581998 |
| 10626 | cg18241942 |
| 10627 | cg25451194 |
| 10628 | cg17426192 |
| 10629 | cg22542786 |
| 10630 | cg20959701 |
| 10631 | cg07015749 |
| 10632 | cg24507760 |
| 10633 | cg15501526 |
| 10634 | cg14342235 |
| 10635 | cg26555052 |
| 10636 | cg21399057 |
| 10637 | cg13687924 |
| 10638 | cg23208513 |
| 10639 | cg15917410 |
| 10640 | cg14552508 |
| 10641 | cg21392341 |
| 10642 | cg04763057 |
| 10643 | cg27175850 |
| 10644 | cg20445630 |
| 10645 | cg06211872 |
| 10646 | cg00670891 |
| 10647 | cg04482075 |
| 10648 | cg07356486 |
| 10649 | cg07396452 |
| 10650 | cg07206531 |
| 10651 | cg12781700 |
| 10652 | cg13374297 |
| 10653 | cg16655778 |
| 10654 | cg25396518 |
| 10655 | cg09789721 |
| 10656 | cg08288561 |
| 10657 | cg06286328 |
| 10658 | cg25686513 |
| 10659 | cg13417158 |
| 10660 | cg11269599 |
| 10661 | cg09546550 |
| 10662 | cg10158249 |
| 10663 | cg02346141 |
| 10664 | cg21174688 |
| 10665 | cg26985528 |
| 10666 | cg08564971 |
| 10667 | cg12943885 |
| 10668 | cg04791901 |
| 10669 | cg05176195 |
| 10670 | cg13744613 |
| 10671 | cg14377922 |
| 10672 | cg07307752 |
| 10673 | cg08772003 |
| 10674 | cg13940693 |
| 10675 | cg18759732 |
| 10676 | cg26562566 |
| 10677 | cg27556496 |
| 10678 | cg00510507 |
| 10679 | cg04992191 |
| 10680 | cg13133747 |
| 10681 | cg19552380 |
| 10682 | cg13536080 |
| 10683 | cg25563479 |
| 10684 | cg19071490 |
| 10685 | cg07950857 |
| 10686 | cg19543867 |
| 10687 | cg11909574 |
| 10688 | cg08496086 |
| 10689 | cg01987516 |
| 10690 | cg00619561 |
| 10691 | cg08996805 |
| 10692 | cg27232494 |
| 10693 | cg07382129 |
| 10694 | cg14724304 |
| 10695 | cg02914097 |
| 10696 | cg05520409 |
| 10697 | cg04436877 |
| 10698 | cg17288288 |
| 10699 | cg25244238 |
| 10700 | cg01295399 |
| 10701 | cg14515614 |
| 10702 | cg22576378 |
| 10703 | cg05615996 |
| 10704 | cg00913360 |
| 10705 | cg06634862 |
| 10706 | cg03655136 |
| 10707 | cg24368865 |
| 10708 | cg00960700 |
| 10709 | cg22625519 |
| 10710 | cg13603551 |
| 10711 | cg25990128 |
| 10712 | cg12031524 |
| 10713 | cg04221461 |
| 10714 | cg27139067 |
| 10715 | cg21695704 |
| 10716 | cg21529533 |
| 10717 | cg00611702 |
| 10718 | cg07224726 |
| 10719 | cg15939514 |
| 10720 | cg03848597 |
| 10721 | cg26264232 |
| 10722 | cg00605342 |
| 10723 | cg14524126 |
| 10724 | cg20555562 |
| 10725 | cg10034027 |
| 10726 | cg18780284 |
| 10727 | cg05111772 |
| 10728 | cg26951440 |
| 10729 | cg15641364 |
| 10730 | cg10327428 |
| 10731 | cg00729699 |
| 10732 | cg00819417 |
| 10733 | cg16842331 |
| 10734 | cg18346412 |
| 10735 | cg09848508 |
| 10736 | cg18566313 |
| 10737 | cg10334283 |
| 10738 | cg03079917 |
| 10739 | cg05284589 |
| 10740 | cg02490034 |
| 10741 | cg15393025 |
| 10742 | cg15310800 |
| 10743 | cg23173402 |
| 10744 | cg12993960 |
| 10745 | cg27253301 |
| 10746 | cg10143960 |
| 10747 | cg08791851 |
| 10748 | cg10117831 |
| 10749 | cg23151014 |
| 10750 | cg08887429 |
| 10751 | cg04350515 |
| 10752 | cg10474862 |
| 10753 | cg20070026 |
| 10754 | cg06894334 |
| 10755 | cg21428710 |
| 10756 | cg16429839 |
| 10757 | cg14104842 |
| 10758 | cg04194678 |
| 10759 | cg17082371 |
| 10760 | cg07074042 |
| 10761 | cg17848838 |
| 10762 | cg09959817 |
| 10763 | cg18005828 |
| 10764 | cg10260470 |
| 10765 | cg04679530 |
| 10766 | cg13047217 |
| 10767 | cg02324432 |
| 10768 | cg00448563 |
| 10769 | cg23922806 |
| 10770 | cg09379489 |
| 10771 | cg15832029 |
| 10772 | cg00380574 |
| 10773 | cg03791616 |
| 10774 | cg09189780 |
| 10775 | cg25940557 |
| 10776 | cg02780919 |
| 10777 | cg11887376 |
| 10778 | cg06292624 |
| 10779 | cg00191458 |
| 10780 | cg12121162 |
| 10781 | cg24229750 |
| 10782 | cg20485607 |
| 10783 | cg21850819 |
| 10784 | cg26298967 |
| 10785 | cg26875374 |
| 10786 | cg26391794 |
| 10787 | cg01585571 |
| 10788 | cg06265020 |
| 10789 | cg07305472 |
| 10790 | cg19684982 |
| 10791 | cg21042336 |
| 10792 | cg22251513 |
| 10793 | cg01840020 |
| 10794 | cg01585743 |
| 10795 | cg25433496 |
| 10796 | cg21940923 |
| 10797 | cg17872886 |
| 10798 | cg15334372 |
| 10799 | cg20584011 |
| 10800 | cg21438861 |
| 10801 | cg06882607 |
| 10802 | cg16210324 |
| 10803 | cg00547103 |
| 10804 | cg25586361 |
| 10805 | cg01507147 |
| 10806 | cg03400849 |
| 10807 | cg05726195 |
| 10808 | cg07033669 |
| 10809 | cg22871653 |
| 10810 | cg13522891 |
| 10811 | cg07079219 |
| 10812 | cg02476190 |
| 10813 | cg10769521 |
| 10814 | cg18449021 |
| 10815 | cg12919873 |
| 10816 | cg19049754 |
| 10817 | cg00054047 |
| 10818 | cg23240888 |
| 10819 | cg07363270 |
| 10820 | cg07891033 |
| 10821 | cg05896524 |
| 10822 | cg15159324 |
| 10823 | cg14371439 |
| 10824 | cg04484995 |
| 10825 | cg21410109 |
| 10826 | cg02730865 |
| 10827 | cg14641472 |
| 10828 | cg25080229 |
| 10829 | cg15023686 |
| 10830 | cg26779770 |
| 10831 | cg05843219 |
| 10832 | cg23690350 |
| 10833 | cg12865285 |
| 10834 | cg21848673 |
| 10835 | cg14127663 |
| 10836 | cg22810423 |
| 10837 | cg02709139 |
| 10838 | cg06702484 |
| 10839 | cg08619842 |
| 10840 | cg09688374 |
| 10841 | cg02207052 |
| 10842 | cg25700077 |
| 10843 | cg17432762 |
| 10844 | cg17829574 |
| 10845 | cg01151886 |
| 10846 | cg08245083 |
| 10847 | cg23696611 |
| 10848 | cg08801586 |
| 10849 | cg05644508 |
| 10850 | cg11935354 |
| 10851 | cg01055105 |
| 10852 | cg21100638 |
| 10853 | cg18813444 |
| 10854 | cg25400396 |
| 10855 | cg22697684 |
| 10856 | cg02219206 |
| 10857 | cg03962527 |
| 10858 | cg08191899 |
| 10859 | cg10672164 |
| 10860 | cg23102277 |
| 10861 | cg21424703 |
| 10862 | cg00322003 |
| 10863 | cg27560327 |
| 10864 | cg14947564 |
| 10865 | cg24018520 |
| 10866 | cg10095811 |
| 10867 | cg17438030 |
| 10868 | cg21373145 |
| 10869 | cg22085157 |
| 10870 | cg04122498 |
| 10871 | cg19784903 |
| 10872 | cg26549601 |
| 10873 | cg05066153 |
| 10874 | cg14949292 |
| 10875 | cg27189803 |
| 10876 | cg11257595 |
| 10877 | cg09238801 |
| 10878 | cg25164886 |
| 10879 | cg19053046 |
| 10880 | cg14286208 |
| 10881 | cg24390932 |
| 10882 | cg01883635 |
| 10883 | cg21653641 |
| 10884 | cg06573787 |
| 10885 | cg11577089 |
| 10886 | cg17645652 |
| 10887 | cg13363969 |
| 10888 | cg26168907 |
| 10889 | cg10967110 |
| 10890 | cg15480998 |
| 10891 | cg02113995 |
| 10892 | cg16565901 |
| 10893 | cg26661727 |
| 10894 | cg12806797 |
| 10895 | cg18021927 |
| 10896 | cg18810376 |
| 10897 | cg04400594 |
| 10898 | cg06948629 |
| 10899 | cg15921887 |
| 10900 | cg04101806 |
| 10901 | cg14683528 |
| 10902 | cg06771795 |
| 10903 | cg03978389 |
| 10904 | cg18911718 |
| 10905 | cg10163895 |
| 10906 | cg07578772 |
| 10907 | cg06434634 |
| 10908 | cg16474696 |
| 10909 | cg08715884 |
| 10910 | cg27083604 |
| 10911 | cg18055288 |
| 10912 | cg13021857 |
| 10913 | cg09025432 |
| 10914 | cg06239554 |
| 10915 | cg05756094 |
| 10916 | cg24563540 |
| 10917 | cg17848763 |
| 10918 | cg04088433 |
| 10919 | cg01697026 |
| 10920 | cg21189527 |
| 10921 | cg16487097 |
| 10922 | cg04074945 |
| 10923 | cg20525279 |
| 10924 | cg22680020 |
| 10925 | cg22950102 |
| 10926 | cg11107278 |
| 10927 | cg05931898 |
| 10928 | cg16565031 |
| 10929 | cg07192082 |
| 10930 | cg22616525 |
| 10931 | cg21306988 |
| 10932 | cg06935608 |
| 10933 | cg17758205 |
| 10934 | cg02478603 |
| 10935 | cg05683586 |
| 10936 | cg10123377 |
| 10937 | cg21893210 |
| 10938 | cg10038802 |
| 10939 | cg25660691 |
| 10940 | cg27525686 |
| 10941 | cg10039268 |
| 10942 | cg12787323 |
| 10943 | cg18127159 |
| 10944 | cg00758467 |
| 10945 | cg10479554 |
| 10946 | cg05033952 |
| 10947 | cg08659594 |
| 10948 | cg01464718 |
| 10949 | cg26244981 |
| 10950 | cg25818214 |
| 10951 | cg20087093 |
| 10952 | cg20266220 |
| 10953 | cg07138437 |
| 10954 | cg23441359 |
| 10955 | cg03054795 |
| 10956 | cg03355213 |
| 10957 | cg06001716 |
| 10958 | cg01289541 |
| 10959 | cg20869226 |
| 10960 | cg00284369 |
| 10961 | cg27580048 |
| 10962 | cg03678939 |
| 10963 | cg01558242 |
| 10964 | cg05265936 |
| 10965 | cg18222500 |
| 10966 | cg20971474 |
| 10967 | cg02897667 |
| 10968 | cg25470758 |
| 10969 | cg06353655 |
| 10970 | cg20327845 |
| 10971 | cg01761236 |
| 10972 | cg23155467 |
| 10973 | cg00515457 |
| 10974 | cg23325335 |
| 10975 | cg13772742 |
| 10976 | cg24116870 |
| 10977 | cg14764459 |
| 10978 | cg05735955 |
| 10979 | cg20403644 |
| 10980 | cg02910054 |
| 10981 | cg14898177 |
| 10982 | cg11610350 |
| 10983 | cg01335127 |
| 10984 | cg05700079 |
| 10985 | cg08242633 |
| 10986 | cg07142010 |
| 10987 | cg08972081 |
| 10988 | cg01037823 |
| 10989 | cg26420159 |
| 10990 | cg02491905 |
| 10991 | cg24411961 |
| 10992 | cg04610432 |
| 10993 | cg16188287 |
| 10994 | cg06500792 |
| 10995 | cg23081604 |
| 10996 | cg12516875 |
| 10997 | cg14441475 |
| 10998 | cg06289566 |
| 10999 | cg23403099 |
| 11000 | cg14213394 |
| 11001 | cg09849302 |
| 11002 | cg14139082 |
| 11003 | cg02821342 |
| 11004 | cg16714096 |
| 11005 | cg16295725 |
| 11006 | cg04156606 |
| 11007 | cg26423139 |
| 11008 | cg19679397 |
| 11009 | cg21761881 |
| 11010 | cg11538242 |
| 11011 | cg10307907 |
| 11012 | cg08794478 |
| 11013 | cg13485564 |
| 11014 | cg01149239 |
| 11015 | cg04052934 |
| 11016 | cg12738913 |
| 11017 | cg27289770 |
| 11018 | cg12502176 |
| 11019 | cg16288117 |
| 11020 | cg18218772 |
| 11021 | cg04862208 |
| 11022 | cg22618164 |
| 11023 | cg17049418 |
| 11024 | cg13253887 |
| 11025 | cg05635388 |
| 11026 | cg09074223 |
| 11027 | cg13858580 |
| 11028 | cg05599870 |
| 11029 | cg05189127 |
| 11030 | cg23221723 |
| 11031 | cg05144285 |
| 11032 | cg09206774 |
| 11033 | cg13502125 |
| 11034 | cg24622143 |
| 11035 | cg26426938 |
| 11036 | cg23946364 |
| 11037 | cg02969415 |
| 11038 | cg19529551 |
| 11039 | cg20167366 |
| 11040 | cg27223250 |
| 11041 | cg14228940 |
| 11042 | cg05823693 |
| 11043 | cg04497684 |
| 11044 | cg02859215 |
| 11045 | cg04082016 |
| 11046 | cg25547072 |
| 11047 | cg26845297 |
| 11048 | cg08012354 |
| 11049 | cg11665263 |
| 11050 | cg06301449 |
| 11051 | cg26668675 |
| 11052 | cg06630425 |
| 11053 | cg18112087 |
| 11054 | cg06212263 |
| 11055 | cg24151087 |
| 11056 | cg11679293 |
| 11057 | cg18883951 |
| 11058 | cg03601372 |
| 11059 | cg03506028 |
| 11060 | cg19247032 |
| 11061 | cg00152946 |
| 11062 | cg24800728 |
| 11063 | cg22362864 |
| 11064 | cg16274899 |
| 11065 | cg01464703 |
| 11066 | cg17414318 |
| 11067 | cg09781552 |
| 11068 | cg23788201 |
| 11069 | cg24971112 |
| 11070 | cg22595230 |
| 11071 | cg17882867 |
| 11072 | cg16929393 |
| 11073 | cg07404485 |
| 11074 | cg12279968 |
| 11075 | cg09695996 |
| 11076 | cg17520020 |
| 11077 | cg01971393 |
| 11078 | cg01303685 |
| 11079 | cg23127089 |
| 11080 | cg00249383 |
| 11081 | cg06968405 |
| 11082 | cg14116050 |
| 11083 | cg26451415 |
| 11084 | cg02877371 |
| 11085 | cg11284842 |
| 11086 | cg15105996 |
| 11087 | cg16741524 |
| 11088 | cg18687621 |
| 11089 | cg22284043 |
| 11090 | cg05250832 |
| 11091 | cg27108281 |
| 11092 | cg10258107 |
| 11093 | cg19969083 |
| 11094 | cg04990202 |
| 11095 | cg22699361 |
| 11096 | cg11350063 |
| 11097 | cg17509039 |
| 11098 | cg07660671 |
| 11099 | cg22807449 |
| 11100 | cg22713640 |
| 11101 | cg22588854 |
| 11102 | cg23688719 |
| 11103 | cg19742089 |
| 11104 | cg05997362 |
| 11105 | cg16597280 |
| 11106 | cg21183256 |
| 11107 | cg26127836 |
| 11108 | cg21584518 |
| 11109 | cg27478313 |
| 11110 | cg00063174 |
| 11111 | cg07673230 |
| 11112 | cg03853332 |
| 11113 | cg17023856 |
| 11114 | cg23447233 |
| 11115 | cg04216046 |
| 11116 | cg16049439 |
| 11117 | cg18065767 |
| 11118 | cg10946742 |
| 11119 | cg13300939 |
| 11120 | cg13944838 |
| 11121 | cg16058274 |
| 11122 | cg03630088 |
| 11123 | cg07675337 |
| 11124 | cg23768003 |
| 11125 | cg09626867 |
| 11126 | cg20295248 |
| 11127 | cg21924767 |
| 11128 | cg24350475 |
| 11129 | cg14586941 |
| 11130 | cg19255307 |
| 11131 | cg07997333 |
| 11132 | cg04773652 |
| 11133 | cg06236138 |
| 11134 | cg23934295 |
| 11135 | cg23345570 |
| 11136 | cg26276233 |
| 11137 | cg08514195 |
| 11138 | cg18226972 |
| 11139 | cg03086235 |
| 11140 | cg13299762 |
| 11141 | cg23289172 |
| 11142 | cg11561701 |
| 11143 | cg19120053 |
| 11144 | cg05276360 |
| 11145 | cg03281661 |
| 11146 | cg07871612 |
| 11147 | cg07143470 |
| 11148 | cg07525471 |
| 11149 | cg15427676 |
| 11150 | cg09206294 |
| 11151 | cg12680346 |
| 11152 | cg14645095 |
| 11153 | cg19942459 |
| 11154 | cg01902605 |
| 11155 | cg27212023 |
| 11156 | cg22212217 |
| 11157 | cg00362854 |
| 11158 | cg00012529 |
| 11159 | cg09739024 |
| 11160 | cg02721065 |
| 11161 | cg10184431 |
| 11162 | cg20995928 |
| 11163 | cg01172183 |
| 11164 | cg20674490 |
| 11165 | cg04910877 |
| 11166 | cg00088688 |
| 11167 | cg02090877 |
| 11168 | cg25685062 |
| 11169 | cg10426163 |
| 11170 | cg21747310 |
| 11171 | cg16777557 |
| 11172 | cg11001890 |
| 11173 | cg20187236 |
| 11174 | cg18361892 |
| 11175 | cg26787863 |
| 11176 | cg22696984 |
| 11177 | cg19261727 |
| 11178 | cg06022811 |
| 11179 | cg11135967 |
| 11180 | cg04864807 |
| 11181 | cg15963552 |
| 11182 | cg01819555 |
| 11183 | cg25222129 |
| 11184 | cg12836958 |
| 11185 | cg14161159 |
| 11186 | cg17792666 |
| 11187 | cg13602831 |
| 11188 | cg03639671 |
| 11189 | cg02689640 |
| 11190 | cg16903782 |
| 11191 | cg02623200 |
| 11192 | cg18318878 |
| 11193 | cg18187729 |
| 11194 | cg27151321 |
| 11195 | cg23773075 |
| 11196 | cg23870050 |
| 11197 | cg13690280 |
| 11198 | cg11044033 |
| 11199 | cg04378708 |
| 11200 | cg25950769 |
| 11201 | cg05114861 |
| 11202 | cg22994438 |
| 11203 | cg21300895 |
| 11204 | cg16597737 |
| 11205 | cg18815343 |
| 11206 | cg18447131 |
| 11207 | cg16703312 |
| 11208 | cg09859507 |
| 11209 | cg00175395 |
| 11210 | cg00903577 |
| 11211 | cg09515953 |
| 11212 | cg02390954 |
| 11213 | cg25256099 |
| 11214 | cg08532185 |
| 11215 | cg17842918 |
| 11216 | cg20922371 |
| 11217 | cg16199894 |
| 11218 | cg04505023 |
| 11219 | cg08174191 |
| 11220 | cg04900982 |
| 11221 | cg13426079 |
| 11222 | cg25951210 |
| 11223 | cg17509516 |
| 11224 | cg09186101 |
| 11225 | cg15985905 |
| 11226 | cg27614742 |
| 11227 | cg19982971 |
| 11228 | cg16951984 |
| 11229 | cg10514580 |
| 11230 | cg15335436 |
| 11231 | cg24500821 |
| 11232 | cg00524561 |
| 11233 | cg17154174 |
| 11234 | cg18587302 |
| 11235 | cg22543892 |
| 11236 | cg24476331 |
| 11237 | cg19776793 |
| 11238 | cg19428722 |
| 11239 | cg19626253 |
| 11240 | cg14094040 |
| 11241 | cg01572891 |
| 11242 | cg24696728 |
| 11243 | cg06661232 |
| 11244 | cg08546933 |
| 11245 | cg27479162 |
| 11246 | cg13889415 |
| 11247 | cg03134882 |
| 11248 | cg14659008 |
| 11249 | cg18867923 |
| 11250 | cg26987645 |
| 11251 | cg03492184 |
| 11252 | cg11246222 |
| 11253 | cg26588076 |
| 11254 | cg10821127 |
| 11255 | cg16809858 |
| 11256 | cg27388703 |
| 11257 | cg23758822 |
| 11258 | cg18382353 |
| 11259 | cg25818763 |
| 11260 | cg17715478 |
| 11261 | cg00644536 |
| 11262 | cg23689491 |
| 11263 | cg24601344 |
| 11264 | cg17911788 |
| 11265 | cg15964514 |
| 11266 | cg04411245 |
| 11267 | cg16018106 |
| 11268 | cg10432569 |
| 11269 | cg23693781 |
| 11270 | cg25199357 |
| 11271 | cg09464583 |
| 11272 | cg19107578 |
| 11273 | cg24263958 |
| 11274 | cg00122628 |
| 11275 | cg22428969 |
| 11276 | cg25368948 |
| 11277 | cg09992827 |
| 11278 | cg18503195 |
| 11279 | cg01778994 |
| 11280 | cg12733438 |
| 11281 | cg21412973 |
| 11282 | cg21632158 |
| 11283 | cg06988187 |
| 11284 | cg24013909 |
| 11285 | cg25161899 |
| 11286 | cg15086132 |
| 11287 | cg14074117 |
| 11288 | cg12962263 |
| 11289 | cg25082710 |
| 11290 | cg21536086 |
| 11291 | cg13572907 |
| 11292 | cg08862479 |
| 11293 | cg26288821 |
| 11294 | cg02966415 |
| 11295 | cg16368146 |
| 11296 | cg06738242 |
| 11297 | cg11164506 |
| 11298 | cg02049629 |
| 11299 | cg02560447 |
| 11300 | cg01321673 |
| 11301 | cg04025675 |
| 11302 | cg06919524 |
| 11303 | cg07100532 |
| 11304 | cg06710981 |
| 11305 | cg07949597 |
| 11306 | cg11177839 |
| 11307 | cg09951720 |
| 11308 | cg10915440 |
| 11309 | cg04839706 |
| 11310 | cg13000497 |
| 11311 | cg22292345 |
| 11312 | cg00587922 |
| 11313 | cg08130323 |
| 11314 | cg26355796 |
| 11315 | cg20834081 |
| 11316 | cg01552731 |
| 11317 | cg23590705 |
| 11318 | cg24564731 |
| 11319 | cg05796699 |
| 11320 | cg05859267 |
| 11321 | cg00103785 |
| 11322 | cg01168735 |
| 11323 | cg14472069 |
| 11324 | cg21515692 |
| 11325 | cg18045512 |
| 11326 | cg08666638 |
| 11327 | cg06912623 |
| 11328 | cg09200468 |
| 11329 | cg18480996 |
| 11330 | cg10683263 |
| 11331 | cg20417856 |
| 11332 | cg20848988 |
| 11333 | cg14315508 |
| 11334 | cg24044501 |
| 11335 | cg23009355 |
| 11336 | cg00452882 |
| 11337 | cg16391973 |
| 11338 | cg16805094 |
| 11339 | cg15097000 |
| 11340 | cg14970222 |
| 11341 | cg10886276 |
| 11342 | cg02105856 |
| 11343 | cg03597510 |
| 11344 | cg11737757 |
| 11345 | cg04679515 |
| 11346 | cg23549328 |
| 11347 | cg24278234 |
| 11348 | cg04690464 |
| 11349 | cg14438265 |
| 11350 | cg00423453 |
| 11351 | cg06856402 |
| 11352 | cg15112355 |
| 11353 | cg26361173 |
| 11354 | cg05654673 |
| 11355 | cg15998725 |
| 11356 | cg08436738 |
| 11357 | cg03501666 |
| 11358 | cg18688062 |
| 11359 | cg00210249 |
| 11360 | cg15370982 |
| 11361 | cg19624029 |
| 11362 | cg08082355 |
| 11363 | cg19766489 |
| 11364 | cg18631905 |
| 11365 | cg11909513 |
| 11366 | cg21693060 |
| 11367 | cg24255149 |
| 11368 | cg05721476 |
| 11369 | cg20215257 |
| 11370 | cg21614073 |
| 11371 | cg06178055 |
| 11372 | cg21761184 |
| 11373 | cg15698171 |
| 11374 | cg16828484 |
| 11375 | cg08767838 |
| 11376 | cg16474118 |
| 11377 | cg24448003 |
| 11378 | cg21754524 |
| 11379 | cg10084644 |
| 11380 | cg16977630 |
| 11381 | cg16344648 |
| 11382 | cg14902082 |
| 11383 | cg06099439 |
| 11384 | cg04128669 |
| 11385 | cg23324403 |
| 11386 | cg24993742 |
| 11387 | cg22318155 |
| 11388 | cg03543319 |
| 11389 | cg05239310 |
| 11390 | cg12224131 |
| 11391 | cg24842760 |
| 11392 | cg15412718 |
| 11393 | cg08465346 |
| 11394 | cg10236778 |
| 11395 | cg05955301 |
| 11396 | cg03623178 |
| 11397 | cg00940577 |
| 11398 | cg10298052 |
| 11399 | cg05888914 |
| 11400 | cg05484949 |
| 11401 | cg15609387 |
| 11402 | cg01858712 |
| 11403 | cg27226098 |
| 11404 | cg15303917 |
| 11405 | cg25794730 |
| 11406 | cg19597170 |
| 11407 | cg04353304 |
| 11408 | cg09775272 |
| 11409 | cg02964254 |
| 11410 | cg05430997 |
| 11411 | cg21003190 |
| 11412 | cg14671764 |
| 11413 | cg26878182 |
| 11414 | cg08840298 |
| 11415 | cg21446782 |
| 11416 | cg07235577 |
| 11417 | cg26090355 |
| 11418 | cg08930672 |
| 11419 | cg00354542 |
| 11420 | cg12016809 |
| 11421 | cg08436588 |
| 11422 | cg18578631 |
| 11423 | cg16239783 |
| 11424 | cg10257870 |
| 11425 | cg05194726 |
| 11426 | cg15961105 |
| 11427 | cg04820659 |
| 11428 | cg18890556 |
| 11429 | cg05736435 |
| 11430 | cg12267637 |
| 11431 | cg05225684 |
| 11432 | cg05490366 |
| 11433 | cg18339598 |
| 11434 | cg04187516 |
| 11435 | cg27246147 |
| 11436 | cg06542022 |
| 11437 | cg18127823 |
| 11438 | cg13962111 |
| 11439 | cg16794862 |
| 11440 | cg05306472 |
| 11441 | cg02531934 |
| 11442 | cg03593550 |
| 11443 | cg10503007 |
| 11444 | cg06664357 |
| 11445 | cg17397631 |
| 11446 | cg11025877 |
| 11447 | cg07654934 |
| 11448 | cg12797990 |
| 11449 | cg04781580 |
| 11450 | cg11409510 |
| 11451 | cg14842600 |
| 11452 | cg02403137 |
| 11453 | cg02227529 |
| 11454 | cg18020065 |
| 11455 | cg02095219 |
| 11456 | cg23313665 |
| 11457 | cg10344006 |
| 11458 | cg09167301 |
| 11459 | cg19972706 |
| 11460 | cg02184632 |
| 11461 | cg04313756 |
| 11462 | cg21005155 |
| 11463 | cg17579949 |
| 11464 | cg04660147 |
| 11465 | cg23457705 |
| 11466 | cg21767750 |
| 11467 | cg02238178 |
| 11468 | cg13581859 |
| 11469 | cg02092102 |
| 11470 | cg26169783 |
| 11471 | cg08362102 |
| 11472 | cg21433297 |
| 11473 | cg24471924 |
| 11474 | cg09776772 |
| 11475 | cg13831575 |
| 11476 | cg08799816 |
| 11477 | cg08740967 |
| 11478 | cg12161228 |
| 11479 | cg11926656 |
| 11480 | cg00595982 |
| 11481 | cg27250318 |
| 11482 | cg23595710 |
| 11483 | cg19464574 |
| 11484 | cg24741158 |
| 11485 | cg01085125 |
| 11486 | cg20579054 |
| 11487 | cg16144576 |
| 11488 | cg26472714 |
| 11489 | cg05184588 |
| 11490 | cg13219142 |
| 11491 | cg00991504 |
| 11492 | cg14767360 |
| 11493 | cg06419239 |
| 11494 | cg19012965 |
| 11495 | cg26403843 |
| 11496 | cg09158821 |
| 11497 | cg24663003 |
| 11498 | cg22237668 |
| 11499 | cg17049575 |
| 11500 | cg09984303 |
| 11501 | cg22559398 |
| 11502 | cg03502979 |
| 11503 | cg27040186 |
| 11504 | cg05174890 |
| 11505 | cg25855856 |
| 11506 | cg09153953 |
| 11507 | cg07505964 |
| 11508 | cg24573175 |
| 11509 | cg09577607 |
| 11510 | cg24170465 |
| 11511 | cg25547902 |
| 11512 | cg19457556 |
| 11513 | cg12938014 |
| 11514 | cg10921916 |
| 11515 | cg21489194 |
| 11516 | cg14918082 |
| 11517 | cg20529645 |
| 11518 | cg08476055 |
| 11519 | cg13626246 |
| 11520 | cg21567337 |
| 11521 | cg25653659 |
| 11522 | cg07979034 |
| 11523 | cg06401227 |
| 11524 | cg12726120 |
| 11525 | cg11706865 |
| 11526 | cg05370094 |
| 11527 | cg07286060 |
| 11528 | cg00577935 |
| 11529 | cg22489931 |
| 11530 | cg13922451 |
| 11531 | cg00142072 |
| 11532 | cg04681810 |
| 11533 | cg11541409 |
| 11534 | cg25885684 |
| 11535 | cg20116199 |
| 11536 | cg11034122 |
| 11537 | cg12306853 |
| 11538 | cg16103990 |
| 11539 | cg11603365 |
| 11540 | cg01227133 |
| 11541 | cg06768670 |
| 11542 | cg17169998 |
| 11543 | cg17748724 |
| 11544 | cg12078522 |
| 11545 | cg06591973 |
| 11546 | cg01605075 |
| 11547 | cg08371556 |
| 11548 | cg00841035 |
| 11549 | cg05166028 |
| 11550 | cg11396910 |
| 11551 | cg06202614 |
| 11552 | cg06523799 |
| 11553 | cg19621723 |
| 11554 | cg20900938 |
| 11555 | cg06965174 |
| 11556 | cg22032508 |
| 11557 | cg02158486 |
| 11558 | cg18701055 |
| 11559 | cg03313607 |
| 11560 | cg20165429 |
| 11561 | cg24795274 |
| 11562 | cg10709246 |
| 11563 | cg24002887 |
| 11564 | cg24226148 |
| 11565 | cg07323141 |
| 11566 | cg11358405 |
| 11567 | cg27550372 |
| 11568 | cg07425348 |
| 11569 | cg01247690 |
| 11570 | cg18519905 |
| 11571 | cg25087163 |
| 11572 | cg13503564 |
| 11573 | cg10961186 |
| 11574 | cg23826341 |
| 11575 | cg21745165 |
| 11576 | cg14688905 |
| 11577 | cg15455864 |
| 11578 | cg10664343 |
| 11579 | cg20780394 |
| 11580 | cg17542385 |
| 11581 | cg26916966 |
| 11582 | cg06610944 |
| 11583 | cg14758875 |
| 11584 | cg12828654 |
| 11585 | cg10585661 |
| 11586 | cg27417153 |
| 11587 | cg00151370 |
| 11588 | cg05139114 |
| 11589 | cg26962579 |
| 11590 | cg03022891 |
| 11591 | cg02297974 |
| 11592 | cg23829076 |
| 11593 | cg25329325 |
| 11594 | cg11009362 |
| 11595 | cg22330763 |
| 11596 | cg15976388 |
| 11597 | cg24173753 |
| 11598 | cg14360799 |
| 11599 | cg11525020 |
| 11600 | cg01109219 |
| 11601 | cg08110272 |
| 11602 | cg21472642 |
| 11603 | cg19389884 |
| 11604 | cg08335620 |
| 11605 | cg01153946 |
| 11606 | cg07024531 |
| 11607 | cg17256922 |
| 11608 | cg17592802 |
| 11609 | cg12812107 |
| 11610 | cg17710947 |
| 11611 | cg00216277 |
| 11612 | cg13205937 |
| 11613 | cg20495738 |
| 11614 | cg08871354 |
| 11615 | cg16680159 |
| 11616 | cg01258001 |
| 11617 | cg11875358 |
| 11618 | cg24707486 |
| 11619 | cg05917419 |
| 11620 | cg20305489 |
| 11621 | cg17135225 |
| 11622 | cg09154639 |
| 11623 | cg04550737 |
| 11624 | cg02837600 |
| 11625 | cg20447730 |
| 11626 | cg16520815 |
| 11627 | cg06465011 |
| 11628 | cg18431856 |
| 11629 | cg25113237 |
| 11630 | cg15868523 |
| 11631 | cg14454571 |
| 11632 | cg11439596 |
| 11633 | cg15971010 |
| 11634 | cg23695072 |
| 11635 | cg14071650 |
| 11636 | cg08291902 |
| 11637 | cg03654488 |
| 11638 | cg10680621 |
| 11639 | cg16506036 |
| 11640 | cg10334750 |
| 11641 | cg25492585 |
| 11642 | cg01048287 |
| 11643 | cg09347176 |
| 11644 | cg12258179 |
| 11645 | cg19434183 |
| 11646 | cg19415514 |
| 11647 | cg13787135 |
| 11648 | cg02885454 |
| 11649 | cg07371504 |
| 11650 | cg11262850 |
| 11651 | cg08041736 |
| 11652 | cg26313341 |
| 11653 | cg12337525 |
| 11654 | cg18654238 |
| 11655 | cg04408104 |
| 11656 | cg22214746 |
| 11657 | cg25052156 |
| 11658 | cg24382698 |
| 11659 | cg03458344 |
| 11660 | cg16183736 |
| 11661 | cg05439421 |
| 11662 | cg10406295 |
| 11663 | cg13002740 |
| 11664 | cg26119806 |
| 11665 | cg00604900 |
| 11666 | cg19651757 |
| 11667 | cg09809434 |
| 11668 | cg10880006 |
| 11669 | cg12166711 |
| 11670 | cg04488758 |
| 11671 | cg01345320 |
| 11672 | cg14887505 |
| 11673 | cg06157862 |
| 11674 | cg17481703 |
| 11675 | cg01462607 |
| 11676 | cg16151651 |
| 11677 | cg14332120 |
| 11678 | cg04050824 |
| 11679 | cg01840183 |
| 11680 | cg14943664 |
| 11681 | cg03465894 |
| 11682 | cg23427404 |
| 11683 | cg01635668 |
| 11684 | cg10842913 |
| 11685 | cg12283398 |
| 11686 | cg23332989 |
| 11687 | cg11285496 |
| 11688 | cg11553865 |
| 11689 | cg16705273 |
| 11690 | cg18153651 |
| 11691 | cg13254302 |
| 11692 | cg09197075 |
| 11693 | cg06494592 |
| 11694 | cg07769790 |
| 11695 | cg07333030 |
| 11696 | cg17944326 |
| 11697 | cg14469826 |
| 11698 | cg01252585 |
| 11699 | cg12236088 |
| 11700 | cg11170686 |
| 11701 | cg05810129 |
| 11702 | cg26640984 |
| 11703 | cg08687322 |
| 11704 | cg13996453 |
| 11705 | cg03075307 |
| 11706 | cg18242593 |
| 11707 | cg02223330 |
| 11708 | cg19156875 |
| 11709 | cg24179734 |
| 11710 | cg08190450 |
| 11711 | cg20336809 |
| 11712 | cg19475020 |
| 11713 | cg18087672 |
| 11714 | cg19463022 |
| 11715 | cg09739607 |
| 11716 | cg07150342 |
| 11717 | cg00070899 |
| 11718 | cg05199127 |
| 11719 | cg00794911 |
| 11720 | cg23184252 |
| 11721 | cg12246863 |
| 11722 | cg10834218 |
| 11723 | cg00067824 |
| 11724 | cg09062114 |
| 11725 | cg23916780 |
| 11726 | cg04340928 |
| 11727 | cg22878054 |
| 11728 | cg03324638 |
| 11729 | cg24284534 |
| 11730 | cg07938359 |
| 11731 | cg13269555 |
| 11732 | cg07651658 |
| 11733 | cg00285317 |
| 11734 | cg04364695 |
| 11735 | cg03274046 |
| 11736 | cg22171081 |
| 11737 | cg12587618 |
| 11738 | cg04057339 |
| 11739 | cg09757158 |
| 11740 | cg11676189 |
| 11741 | cg20822579 |
| 11742 | cg12886391 |
| 11743 | cg14347274 |
| 11744 | cg00169856 |
| 11745 | cg21642103 |
| 11746 | cg25076597 |
| 11747 | cg18037117 |
| 11748 | cg17466795 |
| 11749 | cg11792771 |
| 11750 | cg12890133 |
| 11751 | cg23268102 |
| 11752 | cg20190718 |
| 11753 | cg20790520 |
| 11754 | cg27008646 |
| 11755 | cg16390932 |
| 11756 | cg11503875 |
| 11757 | cg06610988 |
| 11758 | cg10060191 |
| 11759 | cg02101812 |
| 11760 | cg15713972 |
| 11761 | cg26452556 |
| 11762 | cg15795580 |
| 11763 | cg14225031 |
| 11764 | cg23017915 |
| 11765 | cg02036521 |
| 11766 | cg01627669 |
| 11767 | cg03161190 |
| 11768 | cg23639308 |
| 11769 | cg10574494 |
| 11770 | cg09190470 |
| 11771 | cg09734183 |
| 11772 | cg27627246 |
| 11773 | cg12942894 |
| 11774 | cg11490944 |
| 11775 | cg01969769 |
| 11776 | cg27646850 |
| 11777 | cg18756476 |
| 11778 | cg24653772 |
| 11779 | cg16670376 |
| 11780 | cg21497935 |
| 11781 | cg11772355 |
| 11782 | cg25575735 |
| 11783 | cg05640560 |
| 11784 | cg15612266 |
| 11785 | cg13407335 |
| 11786 | cg11125805 |
| 11787 | cg14617041 |
| 11788 | cg24200165 |
| 11789 | cg24933157 |
| 11790 | cg13802714 |
| 11791 | cg21963544 |
| 11792 | cg03068446 |
| 11793 | cg11057205 |
| 11794 | cg13241889 |
| 11795 | cg21728107 |
| 11796 | cg06394677 |
| 11797 | cg00948749 |
| 11798 | cg11226105 |
| 11799 | cg01618102 |
| 11800 | cg09632946 |
| 11801 | cg20857694 |
| 11802 | cg04843067 |
| 11803 | cg04681600 |
| 11804 | cg13255282 |
| 11805 | cg16479166 |
| 11806 | cg07504457 |
| 11807 | cg20656525 |
| 11808 | cg00729644 |
| 11809 | cg12880878 |
| 11810 | cg03378725 |
| 11811 | cg17349544 |
| 11812 | cg11042561 |
| 11813 | cg07639026 |
| 11814 | cg13098432 |
| 11815 | cg02821895 |
| 11816 | cg25494045 |
| 11817 | cg05614627 |
| 11818 | cg01412970 |
| 11819 | cg10780090 |
| 11820 | cg27314116 |
| 11821 | cg26384993 |
| 11822 | cg21766976 |
| 11823 | cg08506931 |
| 11824 | cg27240317 |
| 11825 | cg24217948 |
| 11826 | cg27118929 |
| 11827 | cg02603747 |
| 11828 | cg00049664 |
| 11829 | cg07838230 |
| 11830 | cg19920417 |
| 11831 | cg21728073 |
| 11832 | cg06132069 |
| 11833 | cg01779765 |
| 11834 | cg17610800 |
| 11835 | cg25034991 |
| 11836 | cg12681986 |
| 11837 | cg08687088 |
| 11838 | cg16927164 |
| 11839 | cg14924160 |
| 11840 | cg20744362 |
| 11841 | cg27183818 |
| 11842 | cg22775635 |
| 11843 | cg19135362 |
| 11844 | cg05477834 |
| 11845 | cg20246851 |
| 11846 | cg16995768 |
| 11847 | cg19786983 |
| 11848 | cg05696950 |
| 11849 | cg24501381 |
| 11850 | cg08810107 |
| 11851 | cg03494428 |
| 11852 | cg02108675 |
| 11853 | cg20994734 |
| 11854 | cg15824056 |
| 11855 | cg27315958 |
| 11856 | cg13878646 |
| 11857 | cg10723259 |
| 11858 | cg24875017 |
| 11859 | cg00235755 |
| 11860 | cg17355668 |
| 11861 | cg10448227 |
| 11862 | cg05499367 |
| 11863 | cg06354695 |
| 11864 | cg24128731 |
| 11865 | cg05842420 |
| 11866 | cg12854143 |
| 11867 | cg24041118 |
| 11868 | cg22624568 |
| 11869 | cg09122208 |
| 11870 | cg27197651 |
| 11871 | cg13457971 |
| 11872 | cg09859040 |
| 11873 | cg23495279 |
| 11874 | cg26357344 |
| 11875 | cg04816699 |
| 11876 | cg18143392 |
| 11877 | cg00996827 |
| 11878 | cg20314763 |
| 11879 | cg11811897 |
| 11880 | cg00108027 |
| 11881 | cg22680668 |
| 11882 | cg22334681 |
| 11883 | cg25165608 |
| 11884 | cg22734513 |
| 11885 | cg01722936 |
| 11886 | cg13632531 |
| 11887 | cg20289045 |
| 11888 | cg15661018 |
| 11889 | cg04539301 |
| 11890 | cg07678518 |
| 11891 | cg17702388 |
| 11892 | cg23174662 |
| 11893 | cg16839129 |
| 11894 | cg07470207 |
| 11895 | cg07081203 |
| 11896 | cg11187522 |
| 11897 | cg02423433 |
| 11898 | cg07429192 |
| 11899 | cg08309809 |
| 11900 | cg03690861 |
| 11901 | cg08977866 |
| 11902 | cg08498156 |
| 11903 | cg21438541 |
| 11904 | cg14966782 |
| 11905 | cg15833353 |
| 11906 | cg16932094 |
| 11907 | cg06479793 |
| 11908 | cg00120840 |
| 11909 | cg02943290 |
| 11910 | cg05377824 |
| 11911 | cg03469862 |
| 11912 | cg01587052 |
| 11913 | cg15380539 |
| 11914 | cg16950129 |
| 11915 | cg16137851 |
| 11916 | cg19216056 |
| 11917 | cg19213407 |
| 11918 | cg09929763 |
| 11919 | cg11613741 |
| 11920 | cg18113094 |
| 11921 | cg26484108 |
| 11922 | cg19922435 |
| 11923 | cg05412137 |
| 11924 | cg12161349 |
| 11925 | cg02698622 |
| 11926 | cg07475127 |
| 11927 | cg23460961 |
| 11928 | cg23454797 |
| 11929 | cg17192247 |
| 11930 | cg03616827 |
| 11931 | cg02999013 |
| 11932 | cg01744822 |
| 11933 | cg09661228 |
| 11934 | cg11070989 |
| 11935 | cg26469483 |
| 11936 | cg09493975 |
| 11937 | cg12740098 |
| 11938 | cg19901403 |
| 11939 | cg04835733 |
| 11940 | cg05047713 |
| 11941 | cg14058535 |
| 11942 | cg04430024 |
| 11943 | cg14087168 |
| 11944 | cg07343861 |
| 11945 | cg14074970 |
| 11946 | cg06078227 |
| 11947 | cg13215552 |
| 11948 | cg06310816 |
| 11949 | cg09964873 |
| 11950 | cg26145873 |
| 11951 | cg08693080 |
| 11952 | cg05085127 |
| 11953 | cg15892620 |
| 11954 | cg19592194 |
| 11955 | cg05373263 |
| 11956 | cg16563367 |
| 11957 | cg08924203 |
| 11958 | cg25949445 |
| 11959 | cg13672525 |
| 11960 | cg07911961 |
| 11961 | cg21324731 |
| 11962 | cg20707907 |
| 11963 | cg26312807 |
| 11964 | cg14917135 |
| 11965 | cg22547540 |
| 11966 | cg12968732 |
| 11967 | cg06537391 |
| 11968 | cg15013136 |
| 11969 | cg27235148 |
| 11970 | cg00184826 |
| 11971 | cg03221483 |
| 11972 | cg22053159 |
| 11973 | cg15527134 |
| 11974 | cg20468815 |
| 11975 | cg06059810 |
| 11976 | cg08313547 |
| 11977 | cg08140389 |
| 11978 | cg26014113 |
| 11979 | cg21222002 |
| 11980 | cg09273298 |
| 11981 | cg05344026 |
| 11982 | cg04691502 |
| 11983 | cg08412316 |
| 11984 | cg13181327 |
| 11985 | cg16171484 |
| 11986 | cg03689265 |
| 11987 | cg18548864 |
| 11988 | cg21090075 |
| 11989 | cg15205204 |
| 11990 | cg07047280 |
| 11991 | cg08203012 |
| 11992 | cg25008858 |
| 11993 | cg27196974 |
| 11994 | cg09716613 |
| 11995 | cg16794961 |
| 11996 | cg16596295 |
| 11997 | cg08380850 |
| 11998 | cg08669096 |
| 11999 | cg22116670 |
| 12000 | cg22992730 |
| 12001 | cg12958020 |
| 12002 | cg14742445 |
| 12003 | cg17034398 |
| 12004 | cg08897054 |
| 12005 | cg02921122 |
| 12006 | cg05540472 |
| 12007 | cg09530156 |
| 12008 | cg13554231 |
| 12009 | cg16907110 |
| 12010 | cg00584840 |
| 12011 | cg08574978 |
| 12012 | cg00760014 |
| 12013 | cg02109699 |
| 12014 | cg17657898 |
| 12015 | cg22895815 |
| 12016 | cg21531300 |
| 12017 | cg19617611 |
| 12018 | cg14832449 |
| 12019 | cg00998744 |
| 12020 | cg24878207 |
| 12021 | cg03473745 |
| 12022 | cg24169812 |
| 12023 | cg23947928 |
| 12024 | cg12036303 |
| 12025 | cg03733207 |
| 12026 | cg23596287 |
| 12027 | cg00952822 |
| 12028 | cg16425799 |
| 12029 | cg04012681 |
| 12030 | cg15616257 |
| 12031 | cg04303500 |
| 12032 | cg10736660 |
| 12033 | cg03180835 |
| 12034 | cg13015534 |
| 12035 | cg24764848 |
| 12036 | cg14079645 |
| 12037 | cg06936155 |
| 12038 | cg10836101 |
| 12039 | cg24169486 |
| 12040 | cg15524363 |
| 12041 | cg17330048 |
| 12042 | cg03963219 |
| 12043 | cg03103463 |
| 12044 | cg27254970 |
| 12045 | cg10312195 |
| 12046 | cg22899559 |
| 12047 | cg07424912 |
| 12048 | cg18217622 |
| 12049 | cg02101773 |
| 12050 | cg17180284 |
| 12051 | cg03092680 |
| 12052 | cg23831143 |
| 12053 | cg13523713 |
| 12054 | cg00522018 |
| 12055 | cg18565539 |
| 12056 | cg10989634 |
| 12057 | cg05737530 |
| 12058 | cg00273198 |
| 12059 | cg09065629 |
| 12060 | cg21858526 |
| 12061 | cg18888403 |
| 12062 | cg04218710 |
| 12063 | cg19889626 |
| 12064 | cg19852390 |
| 12065 | cg21261608 |
| 12066 | cg16718562 |
| 12067 | cg26503073 |
| 12068 | cg06478886 |
| 12069 | cg19593741 |
| 12070 | cg14207589 |
| 12071 | cg22377043 |
| 12072 | cg02120433 |
| 12073 | cg02516577 |
| 12074 | cg26728709 |
| 12075 | cg07630041 |
| 12076 | cg20882551 |
| 12077 | cg03513146 |
| 12078 | cg20810848 |
| 12079 | cg26513786 |
| 12080 | cg12512804 |
| 12081 | cg02881516 |
| 12082 | cg18959279 |
| 12083 | cg18064631 |
| 12084 | cg21193577 |
| 12085 | cg00876532 |
| 12086 | cg18050420 |
| 12087 | cg14903940 |
| 12088 | cg22953865 |
| 12089 | cg19661478 |
| 12090 | cg04285666 |
| 12091 | cg17158750 |
| 12092 | cg16495772 |
| 12093 | cg12379611 |
| 12094 | cg01485039 |
| 12095 | cg05670715 |
| 12096 | cg11381759 |
| 12097 | cg21708130 |
| 12098 | cg00356131 |
| 12099 | cg27415655 |
| 12100 | cg18925980 |
| 12101 | cg07598481 |
| 12102 | cg21239227 |
| 12103 | cg13756965 |
| 12104 | cg10807101 |
| 12105 | cg07245057 |
| 12106 | cg09358376 |
| 12107 | cg07809108 |
| 12108 | cg01170129 |
| 12109 | cg21621782 |
| 12110 | cg17465132 |
| 12111 | cg15530916 |
| 12112 | cg02669584 |
| 12113 | cg14240646 |
| 12114 | cg12646227 |
| 12115 | cg10902717 |
| 12116 | cg13625026 |
| 12117 | cg07171609 |
| 12118 | cg19498112 |
| 12119 | cg06373268 |
| 12120 | cg18403478 |
| 12121 | cg15557878 |
| 12122 | cg19015997 |
| 12123 | cg13618599 |
| 12124 | cg07362492 |
| 12125 | cg25968713 |
| 12126 | cg12594793 |
| 12127 | cg04140854 |
| 12128 | cg12682133 |
| 12129 | cg24884543 |
| 12130 | cg21476494 |
| 12131 | cg09473315 |
| 12132 | cg01288114 |
| 12133 | cg08160592 |
| 12134 | cg12798245 |
| 12135 | cg26099164 |
| 12136 | cg00009737 |
| 12137 | cg00213192 |
| 12138 | cg00904576 |
| 12139 | cg20725021 |
| 12140 | cg03464366 |
| 12141 | cg10379472 |
| 12142 | cg23796357 |
| 12143 | cg11431604 |
| 12144 | cg04655941 |
| 12145 | cg21319527 |
| 12146 | cg11610703 |
| 12147 | cg13832817 |
| 12148 | cg09627874 |
| 12149 | cg24698785 |
| 12150 | cg02500883 |
| 12151 | cg15460348 |
| 12152 | cg01427471 |
| 12153 | cg02541592 |
| 12154 | cg02077084 |
| 12155 | cg16067929 |
| 12156 | cg11439126 |
| 12157 | cg11024728 |
| 12158 | cg18735473 |
| 12159 | cg15659713 |
| 12160 | cg15684231 |
| 12161 | cg01549227 |
| 12162 | cg07484739 |
| 12163 | cg05763918 |
| 12164 | cg11703632 |
| 12165 | cg25449484 |
| 12166 | cg10580110 |
| 12167 | cg23363372 |
| 12168 | cg26899625 |
| 12169 | cg13046608 |
| 12170 | cg07229001 |
| 12171 | cg00134295 |
| 12172 | cg07071491 |
| 12173 | cg18540673 |
| 12174 | cg08396985 |
| 12175 | cg07461715 |
| 12176 | cg07890104 |
| 12177 | cg20331795 |
| 12178 | cg17804112 |
| 12179 | cg04890478 |
| 12180 | cg20923122 |
| 12181 | cg18077387 |
| 12182 | cg20427312 |
| 12183 | cg06863994 |
| 12184 | cg04855271 |
| 12185 | cg00275126 |
| 12186 | cg03537184 |
| 12187 | cg09114162 |
| 12188 | cg04839207 |
| 12189 | cg06599209 |
| 12190 | cg01973778 |
| 12191 | cg23651088 |
| 12192 | cg16553705 |
| 12193 | cg05557444 |
| 12194 | cg13151010 |
| 12195 | cg14026238 |
| 12196 | cg10038618 |
| 12197 | cg15431659 |
| 12198 | cg25360734 |
| 12199 | cg27396102 |
| 12200 | cg10068034 |
| 12201 | cg00761069 |
| 12202 | cg26150142 |
| 12203 | cg22825261 |
| 12204 | cg08360511 |
| 12205 | cg13478228 |
| 12206 | cg01484075 |
| 12207 | cg01486574 |
| 12208 | cg07549302 |
| 12209 | cg02100011 |
| 12210 | cg09259466 |
| 12211 | cg00666014 |
| 12212 | cg14255238 |
| 12213 | cg02479022 |
| 12214 | cg20779404 |
| 12215 | cg10613567 |
| 12216 | cg07991343 |
| 12217 | cg01962146 |
| 12218 | cg11112161 |
| 12219 | cg18881380 |
| 12220 | cg06649410 |
| 12221 | cg17765387 |
| 12222 | cg25706149 |
| 12223 | cg23165899 |
| 12224 | cg12485147 |
| 12225 | cg19856127 |
| 12226 | cg14637111 |
| 12227 | cg03874476 |
| 12228 | cg02595823 |
| 12229 | cg01583485 |
| 12230 | cg17463698 |
| 12231 | cg24334512 |
| 12232 | cg22621169 |
| 12233 | cg01755541 |
| 12234 | cg13470069 |
| 12235 | cg12984893 |
| 12236 | cg21551253 |
| 12237 | cg26837800 |
| 12238 | cg21351647 |
| 12239 | cg15948428 |
| 12240 | cg20853591 |
| 12241 | cg07836815 |
| 12242 | cg09477019 |
| 12243 | cg18679976 |
| 12244 | cg05105260 |
| 12245 | cg18564052 |
| 12246 | cg00035348 |
| 12247 | cg12113251 |
| 12248 | cg01940753 |
| 12249 | cg10433327 |
| 12250 | cg07928365 |
| 12251 | cg18739977 |
| 12252 | cg17456644 |
| 12253 | cg06785055 |
| 12254 | cg06878994 |
| 12255 | cg14652095 |
| 12256 | cg05132739 |
| 12257 | cg01564380 |
| 12258 | cg23659289 |
| 12259 | cg26338105 |
| 12260 | cg25082074 |
| 12261 | cg08667670 |
| 12262 | cg00524220 |
| 12263 | cg05026964 |
| 12264 | cg25317338 |
| 12265 | cg04669107 |
| 12266 | cg14609015 |
| 12267 | cg15949805 |
| 12268 | cg00806704 |
| 12269 | cg17464766 |
| 12270 | cg22484012 |
| 12271 | cg05596156 |
| 12272 | cg12278959 |
| 12273 | cg03009030 |
| 12274 | cg25328184 |
| 12275 | cg21464565 |
| 12276 | cg17484699 |
| 12277 | cg15817199 |
| 12278 | cg03058273 |
| 12279 | cg19412964 |
| 12280 | cg10465765 |
| 12281 | cg26622285 |
| 12282 | cg03247066 |
| 12283 | cg04485391 |
| 12284 | cg14753161 |
| 12285 | cg02004499 |
| 12286 | cg10811045 |
| 12287 | cg11140265 |
| 12288 | cg07823711 |
| 12289 | cg13345683 |
| 12290 | cg11216632 |
| 12291 | cg19111519 |
| 12292 | cg01858698 |
| 12293 | cg01086974 |
| 12294 | cg10143603 |
| 12295 | cg17258228 |
| 12296 | cg13827224 |
| 12297 | cg18584071 |
| 12298 | cg03465652 |
| 12299 | cg02403198 |
| 12300 | cg24469729 |
| 12301 | cg20778547 |
| 12302 | cg24433043 |
| 12303 | cg17429236 |
| 12304 | cg08311935 |
| 12305 | cg14087045 |
| 12306 | cg16248584 |
| 12307 | cg06376701 |
| 12308 | cg16097708 |
| 12309 | cg09642854 |
| 12310 | cg07465009 |
| 12311 | cg04552784 |
| 12312 | cg11701486 |
| 12313 | cg17872867 |
| 12314 | cg19960420 |
| 12315 | cg08034070 |
| 12316 | cg07778635 |
| 12317 | cg19669385 |
| 12318 | cg08657492 |
| 12319 | cg10894527 |
| 12320 | cg00980620 |
| 12321 | cg23525910 |
| 12322 | cg15029475 |
| 12323 | cg12311882 |
| 12324 | cg07609007 |
| 12325 | cg19152802 |
| 12326 | cg03339910 |
| 12327 | cg01871127 |
| 12328 | cg05369926 |
| 12329 | cg13588483 |
| 12330 | cg10140454 |
| 12331 | cg04415270 |
| 12332 | cg24457562 |
| 12333 | cg21672572 |
| 12334 | cg18199264 |
| 12335 | cg26746037 |
| 12336 | cg05244766 |
| 12337 | cg12042187 |
| 12338 | cg09146471 |
| 12339 | cg17052756 |
| 12340 | cg17250160 |
| 12341 | cg11846905 |
| 12342 | cg02280693 |
| 12343 | cg20510474 |
| 12344 | cg22888055 |
| 12345 | cg17569124 |
| 12346 | cg04166469 |
| 12347 | cg20554943 |
| 12348 | cg18164611 |
| 12349 | cg19010490 |
| 12350 | cg00481350 |
| 12351 | cg00023919 |
| 12352 | cg06281169 |
| 12353 | cg12583553 |
| 12354 | cg04415535 |
| 12355 | cg14078244 |
| 12356 | cg16374627 |
| 12357 | cg01166989 |
| 12358 | cg15273860 |
| 12359 | cg09189898 |
| 12360 | cg00208218 |
| 12361 | cg09109411 |
| 12362 | cg14985989 |
| 12363 | cg24127305 |
| 12364 | cg08445992 |
| 12365 | cg15737787 |
| 12366 | cg02845204 |
| 12367 | cg26643133 |
| 12368 | cg08296601 |
| 12369 | cg11374703 |
| 12370 | cg02228185 |
| 12371 | cg24408216 |
| 12372 | cg20170028 |
| 12373 | cg20303075 |
| 12374 | cg22531183 |
| 12375 | cg12774946 |
| 12376 | cg19546575 |
| 12377 | cg11342331 |
| 12378 | cg09218957 |
| 12379 | cg14492842 |
| 12380 | cg08226958 |
| 12381 | cg07703495 |
| 12382 | cg03552265 |
| 12383 | cg03517120 |
| 12384 | cg19536401 |
| 12385 | cg20705893 |
| 12386 | cg07571734 |
| 12387 | cg03466406 |
| 12388 | cg20095851 |
| 12389 | cg27459803 |
| 12390 | cg14376836 |
| 12391 | cg16037106 |
| 12392 | cg10714356 |
| 12393 | cg27049827 |
| 12394 | cg08461949 |
| 12395 | cg26674848 |
| 12396 | cg10120652 |
| 12397 | cg26581982 |
| 12398 | cg25462095 |
| 12399 | cg05375685 |
| 12400 | cg23708337 |
| 12401 | cg03628310 |
| 12402 | cg25305636 |
| 12403 | cg26091181 |
| 12404 | cg26534477 |
| 12405 | cg09584225 |
| 12406 | cg19980771 |
| 12407 | cg12303521 |
| 12408 | cg27422872 |
| 12409 | cg23784549 |
| 12410 | cg24724630 |
| 12411 | cg01201024 |
| 12412 | cg18324707 |
| 12413 | cg17242803 |
| 12414 | cg09087222 |
| 12415 | cg12846778 |
| 12416 | cg22055815 |
| 12417 | cg27139827 |
| 12418 | cg07426546 |
| 12419 | cg04958236 |
| 12420 | cg25834518 |
| 12421 | cg07388899 |
| 12422 | cg22242842 |
| 12423 | cg05498007 |
| 12424 | cg01438090 |
| 12425 | cg21380024 |
| 12426 | cg21646955 |
| 12427 | cg16877615 |
| 12428 | cg26330155 |
| 12429 | cg18078545 |
| 12430 | cg17338146 |
| 12431 | cg23405152 |
| 12432 | cg22736323 |
| 12433 | cg07260003 |
| 12434 | cg07918620 |
| 12435 | cg15671083 |
| 12436 | cg02695653 |
| 12437 | cg04524478 |
| 12438 | cg12885388 |
| 12439 | cg01272629 |
| 12440 | cg25130925 |
| 12441 | cg19219933 |
| 12442 | cg19423247 |
| 12443 | cg26882438 |
| 12444 | cg08539965 |
| 12445 | cg04269043 |
| 12446 | cg20767344 |
| 12447 | cg00058522 |
| 12448 | cg08561048 |
| 12449 | cg27532919 |
| 12450 | cg24083583 |
| 12451 | cg08641278 |
| 12452 | cg15021280 |
| 12453 | cg04245026 |
| 12454 | cg20710709 |
| 12455 | cg11726661 |
| 12456 | cg12655939 |
| 12457 | cg24538352 |
| 12458 | cg01375176 |
| 12459 | cg23854395 |
| 12460 | cg13453589 |
| 12461 | cg23596886 |
| 12462 | cg08975641 |
| 12463 | cg09858078 |
| 12464 | cg18440821 |
| 12465 | cg05924882 |
| 12466 | cg03952672 |
| 12467 | cg14891022 |
| 12468 | cg06769759 |
| 12469 | cg13612317 |
| 12470 | cg04697262 |
| 12471 | cg21413867 |
| 12472 | cg04471919 |
| 12473 | cg03581383 |
| 12474 | cg26136497 |
| 12475 | cg22566500 |
| 12476 | cg04833713 |
| 12477 | cg25323302 |
| 12478 | cg05032887 |
| 12479 | cg04293460 |
| 12480 | cg22392592 |
| 12481 | cg03953755 |
| 12482 | cg16594542 |
| 12483 | cg01584202 |
| 12484 | cg25017620 |
| 12485 | cg15066194 |
| 12486 | cg26004759 |
| 12487 | cg24155952 |
| 12488 | cg20464902 |
| 12489 | cg21554715 |
| 12490 | cg25192768 |
| 12491 | cg04921656 |
| 12492 | cg05587038 |
| 12493 | cg18315637 |
| 12494 | cg13609534 |
| 12495 | cg24683609 |
| 12496 | cg23910243 |
| 12497 | cg16081325 |
| 12498 | cg21818791 |
| 12499 | cg03469054 |
| 12500 | cg02524236 |
| 12501 | cg15658676 |
| 12502 | cg20605886 |
| 12503 | cg14653988 |
| 12504 | cg15723222 |
| 12505 | cg23233200 |
| 12506 | cg23003225 |
| 12507 | cg27478242 |
| 12508 | cg12465817 |
| 12509 | cg19690403 |
| 12510 | cg14568830 |
| 12511 | cg01006126 |
| 12512 | cg13392051 |
| 12513 | cg27397893 |
| 12514 | cg17321329 |
| 12515 | cg08322244 |
| 12516 | cg00660513 |
| 12517 | cg24791663 |
| 12518 | cg12233129 |
| 12519 | cg06051734 |
| 12520 | cg10897311 |
| 12521 | cg26173375 |
| 12522 | cg16417374 |
| 12523 | cg05019577 |
| 12524 | cg13359344 |
| 12525 | cg07774982 |
| 12526 | cg25324585 |
| 12527 | cg22930859 |
| 12528 | cg03595348 |
| 12529 | cg25047768 |
| 12530 | cg25770851 |
| 12531 | cg25612391 |
| 12532 | cg02010763 |
| 12533 | cg12162189 |
| 12534 | cg03623449 |
| 12535 | cg04550371 |
| 12536 | cg09594039 |
| 12537 | cg11098984 |
| 12538 | cg09584521 |
| 12539 | cg20845437 |
| 12540 | cg19889856 |
| 12541 | cg11394988 |
| 12542 | cg26428069 |
| 12543 | cg19891452 |
| 12544 | cg06319398 |
| 12545 | cg22539471 |
| 12546 | cg24724832 |
| 12547 | cg03995300 |
| 12548 | cg27084781 |
| 12549 | cg19759671 |
| 12550 | cg03340242 |
| 12551 | cg05952163 |
| 12552 | cg16971128 |
| 12553 | cg23279335 |
| 12554 | cg10390536 |
| 12555 | cg06526095 |
| 12556 | cg02987555 |
| 12557 | cg21215767 |
| 12558 | cg07578119 |
| 12559 | cg16220260 |
| 12560 | cg17433604 |
| 12561 | cg19871388 |
| 12562 | cg20752373 |
| 12563 | cg10655333 |
| 12564 | cg13563023 |
| 12565 | cg05624577 |
| 12566 | cg00807608 |
| 12567 | cg00312027 |
| 12568 | cg20430047 |
| 12569 | cg17878320 |
| 12570 | cg09544050 |
| 12571 | cg03004860 |
| 12572 | cg18207558 |
| 12573 | cg22292753 |
| 12574 | cg00849491 |
| 12575 | cg13588735 |
| 12576 | cg04375046 |
| 12577 | cg10591346 |
| 12578 | cg05950610 |
| 12579 | cg00291004 |
| 12580 | cg15274877 |
| 12581 | cg18074184 |
| 12582 | cg17196675 |
| 12583 | cg17624196 |
| 12584 | cg06019613 |
| 12585 | cg02331830 |
| 12586 | cg18611122 |
| 12587 | cg25136495 |
| 12588 | cg23689227 |
| 12589 | cg26170244 |
| 12590 | cg08016257 |
| 12591 | cg22613854 |
| 12592 | cg07898974 |
| 12593 | cg10186366 |
| 12594 | cg09864183 |
| 12595 | cg12626821 |
| 12596 | cg04137911 |
| 12597 | cg06166523 |
| 12598 | cg18388998 |
| 12599 | cg20721433 |
| 12600 | cg16452865 |
| 12601 | cg17551620 |
| 12602 | cg21461082 |
| 12603 | cg22977892 |
| 12604 | cg08743428 |
| 12605 | cg24137913 |
| 12606 | cg00769356 |
| 12607 | cg12686055 |
| 12608 | cg07572773 |
| 12609 | cg15262515 |
| 12610 | cg09412538 |
| 12611 | cg09574909 |
| 12612 | cg11076306 |
| 12613 | cg00674807 |
| 12614 | cg11097954 |
| 12615 | cg16423289 |
| 12616 | cg26854869 |
| 12617 | cg01880236 |
| 12618 | cg19272348 |
| 12619 | cg12016314 |
| 12620 | cg04237608 |
| 12621 | cg00173659 |
| 12622 | cg21321877 |
| 12623 | cg13911501 |
| 12624 | cg12106976 |
| 12625 | cg21917740 |
| 12626 | cg01318761 |
| 12627 | cg14760149 |
| 12628 | cg20385489 |
| 12629 | cg00789213 |
| 12630 | cg10180297 |
| 12631 | cg07963437 |
| 12632 | cg09979431 |
| 12633 | cg15569947 |
| 12634 | cg17397592 |
| 12635 | cg20421482 |
| 12636 | cg05955251 |
| 12637 | cg14636261 |
| 12638 | cg02258414 |
| 12639 | cg10593047 |
| 12640 | cg23418334 |
| 12641 | cg21761885 |
| 12642 | cg13076095 |
| 12643 | cg01430795 |
| 12644 | cg20059982 |
| 12645 | cg03605686 |
| 12646 | cg00318631 |
| 12647 | cg15780361 |
| 12648 | cg14002385 |
| 12649 | cg23647271 |
| 12650 | cg27082285 |
| 12651 | cg12594341 |
| 12652 | cg18264486 |
| 12653 | cg03782070 |
| 12654 | cg20017382 |
| 12655 | cg02350790 |
| 12656 | cg15308037 |
| 12657 | cg06239064 |
| 12658 | cg22693423 |
| 12659 | cg13444533 |
| 12660 | cg12008896 |
| 12661 | cg18834050 |
| 12662 | cg07490273 |
| 12663 | cg13774536 |
| 12664 | cg07959747 |
| 12665 | cg10693587 |
| 12666 | cg06420764 |
| 12667 | cg17473782 |
| 12668 | cg07894352 |
| 12669 | cg27567314 |
| 12670 | cg24699745 |
| 12671 | cg13252700 |
| 12672 | cg14397696 |
| 12673 | cg16171227 |
| 12674 | cg08816563 |
| 12675 | cg02871968 |
| 12676 | cg26145442 |
| 12677 | cg00174508 |
| 12678 | cg12548176 |
| 12679 | cg07251788 |
| 12680 | cg23683588 |
| 12681 | cg05775160 |
| 12682 | cg03348461 |
| 12683 | cg07947016 |
| 12684 | cg14842108 |
| 12685 | cg27158659 |
| 12686 | cg14668811 |
| 12687 | cg17687433 |
| 12688 | cg19162970 |
| 12689 | cg24301620 |
| 12690 | cg04236980 |
| 12691 | cg14289228 |
| 12692 | cg10721220 |
| 12693 | cg23400715 |
| 12694 | cg22022067 |
| 12695 | cg08666831 |
| 12696 | cg02827075 |
| 12697 | cg17659212 |
| 12698 | cg13428978 |
| 12699 | cg11369811 |
| 12700 | cg17912041 |
| 12701 | cg24868015 |
| 12702 | cg21556726 |
| 12703 | cg06050456 |
| 12704 | cg21593690 |
| 12705 | cg14126466 |
| 12706 | cg09415825 |
| 12707 | cg14952312 |
| 12708 | cg09327655 |
| 12709 | cg21145387 |
| 12710 | cg18073938 |
| 12711 | cg19741660 |
| 12712 | cg27330542 |
| 12713 | cg05115902 |
| 12714 | cg09773881 |
| 12715 | cg17945153 |
| 12716 | cg22708657 |
| 12717 | cg01982088 |
| 12718 | cg08178072 |
| 12719 | cg27027151 |
| 12720 | cg07034255 |
| 12721 | cg18107094 |
| 12722 | cg26128135 |
| 12723 | cg13867638 |
| 12724 | cg19935757 |
| 12725 | cg23138440 |
| 12726 | cg13418230 |
| 12727 | cg19916129 |
| 12728 | cg14970802 |
| 12729 | cg08113064 |
| 12730 | cg05144672 |
| 12731 | cg18912728 |
| 12732 | cg19286782 |
| 12733 | cg19597382 |
| 12734 | cg04843461 |
| 12735 | cg16336586 |
| 12736 | cg03264751 |
| 12737 | cg14130249 |
| 12738 | cg22524026 |
| 12739 | cg01188674 |
| 12740 | cg02884181 |
| 12741 | cg00592428 |
| 12742 | cg09980470 |
| 12743 | cg26663490 |
| 12744 | cg25132257 |
| 12745 | cg08401703 |
| 12746 | cg24279897 |
| 12747 | cg13301003 |
| 12748 | cg27129881 |
| 12749 | cg22381128 |
| 12750 | cg04217778 |
| 12751 | cg01843823 |
| 12752 | cg01153077 |
| 12753 | cg09785958 |
| 12754 | cg25313074 |
| 12755 | cg01704924 |
| 12756 | cg26121389 |
| 12757 | cg00767058 |
| 12758 | cg01298990 |
| 12759 | cg15496864 |
| 12760 | cg05631447 |
| 12761 | cg05719140 |
| 12762 | cg17055322 |
| 12763 | cg13906823 |
| 12764 | cg06869505 |
| 12765 | cg05483505 |
| 12766 | cg07894089 |
| 12767 | cg26472922 |
| 12768 | cg16359550 |
| 12769 | cg08456859 |
| 12770 | cg10752315 |
| 12771 | cg27656728 |
| 12772 | cg18853199 |
| 12773 | cg27355724 |
| 12774 | cg04492926 |
| 12775 | cg05782224 |
| 12776 | cg14383544 |
| 12777 | cg00297721 |
| 12778 | cg16337763 |
| 12779 | cg04220116 |
| 12780 | cg08209379 |
| 12781 | cg27300125 |
| 12782 | cg04144673 |
| 12783 | cg09577367 |
| 12784 | cg13718275 |
| 12785 | cg09356672 |
| 12786 | cg06626315 |
| 12787 | cg27170427 |
| 12788 | cg21917089 |
| 12789 | cg01269718 |
| 12790 | cg19828775 |
| 12791 | cg15910301 |
| 12792 | cg26584734 |
| 12793 | cg13599248 |
| 12794 | cg19712474 |
| 12795 | cg17918326 |
| 12796 | cg20245444 |
| 12797 | cg01742187 |
| 12798 | cg03969260 |
| 12799 | cg17665699 |
| 12800 | cg21434132 |
| 12801 | cg14093252 |
| 12802 | cg02359771 |
| 12803 | cg05501426 |
| 12804 | cg24606065 |
| 12805 | cg26768406 |
| 12806 | cg02428209 |
| 12807 | cg24313303 |
| 12808 | cg07713288 |
| 12809 | cg18708986 |
| 12810 | cg15946224 |
| 12811 | cg17348479 |
| 12812 | cg21173144 |
| 12813 | cg17966344 |
| 12814 | cg08777304 |
| 12815 | cg24783610 |
| 12816 | cg21451712 |
| 12817 | cg07132423 |
| 12818 | cg20354552 |
| 12819 | cg18565026 |
| 12820 | cg07217824 |
| 12821 | cg06593715 |
| 12822 | cg21786289 |
| 12823 | cg14781516 |
| 12824 | cg05437476 |
| 12825 | cg11589211 |
| 12826 | cg13925975 |
| 12827 | cg19417613 |
| 12828 | cg14881381 |
| 12829 | cg08451822 |
| 12830 | cg17355742 |
| 12831 | cg19914189 |
| 12832 | cg15567016 |
| 12833 | cg13685506 |
| 12834 | cg02421985 |
| 12835 | cg22862205 |
| 12836 | cg25362585 |
| 12837 | cg02410504 |
| 12838 | cg11174640 |
| 12839 | cg00677295 |
| 12840 | cg15818715 |
| 12841 | cg07880109 |
| 12842 | cg06142351 |
| 12843 | cg12811617 |
| 12844 | cg08523384 |
| 12845 | cg26066724 |
| 12846 | cg06062604 |
| 12847 | cg00437411 |
| 12848 | cg11288670 |
| 12849 | cg07135660 |
| 12850 | cg16091981 |
| 12851 | cg15001389 |
| 12852 | cg12058478 |
| 12853 | cg25275090 |
| 12854 | cg19416832 |
| 12855 | cg18091225 |
| 12856 | cg11124790 |
| 12857 | cg10719363 |
| 12858 | cg08760670 |
| 12859 | cg18445418 |
| 12860 | cg00359521 |
| 12861 | cg23833461 |
| 12862 | cg07526974 |
| 12863 | cg01751280 |
| 12864 | cg08892158 |
| 12865 | cg11217960 |
| 12866 | cg01668014 |
| 12867 | cg26992028 |
| 12868 | cg22282161 |
| 12869 | cg15044486 |
| 12870 | cg11354393 |
| 12871 | cg21828507 |
| 12872 | cg26407924 |
| 12873 | cg03988700 |
| 12874 | cg22872906 |
| 12875 | cg12157377 |
| 12876 | cg19660531 |
| 12877 | cg17334359 |
| 12878 | cg08631528 |
| 12879 | cg01491926 |
| 12880 | cg11148820 |
| 12881 | cg08383160 |
| 12882 | cg18696218 |
| 12883 | cg18892778 |
| 12884 | cg09153398 |
| 12885 | cg14880894 |
| 12886 | cg07104639 |
| 12887 | cg26918812 |
| 12888 | cg20598347 |
| 12889 | cg21241151 |
| 12890 | cg14437045 |
| 12891 | cg07057989 |
| 12892 | cg25697538 |
| 12893 | cg03307717 |
| 12894 | cg10477704 |
| 12895 | cg26620655 |
| 12896 | cg17842879 |
| 12897 | cg08036309 |
| 12898 | cg12081643 |
| 12899 | cg19908611 |
| 12900 | cg01310137 |
| 12901 | cg11562153 |
| 12902 | cg05294813 |
| 12903 | cg00778084 |
| 12904 | cg05885936 |
| 12905 | cg01094920 |
| 12906 | cg11199014 |
| 12907 | cg26715908 |
| 12908 | cg12233487 |
| 12909 | cg24369989 |
| 12910 | cg17081914 |
| 12911 | cg27032427 |
| 12912 | cg05995260 |
| 12913 | cg02005619 |
| 12914 | cg21575526 |
| 12915 | cg18969008 |
| 12916 | cg11928275 |
| 12917 | cg18475252 |
| 12918 | cg20922821 |
| 12919 | cg15122439 |
| 12920 | cg11958373 |
| 12921 | cg16319142 |
| 12922 | cg10828175 |
| 12923 | cg18587137 |
| 12924 | cg07235076 |
| 12925 | cg26292078 |
| 12926 | cg26621770 |
| 12927 | cg04098790 |
| 12928 | cg05142081 |
| 12929 | cg21442983 |
| 12930 | cg21048812 |
| 12931 | cg19490598 |
| 12932 | cg15723716 |
| 12933 | cg05896714 |
| 12934 | cg14940396 |
| 12935 | cg03325664 |
| 12936 | cg05475210 |
| 12937 | cg09491120 |
| 12938 | cg06103624 |
| 12939 | cg08461840 |
| 12940 | cg06361606 |
| 12941 | cg19595242 |
| 12942 | cg25646708 |
| 12943 | cg26365969 |
| 12944 | cg19098465 |
| 12945 | cg19696794 |
| 12946 | cg09450085 |
| 12947 | cg03803086 |
| 12948 | cg21908557 |
| 12949 | cg23904955 |
| 12950 | cg02741226 |
| 12951 | cg20805367 |
| 12952 | cg05846633 |
| 12953 | cg14152068 |
| 12954 | cg15672877 |
| 12955 | cg22462010 |
| 12956 | cg00714377 |
| 12957 | cg03707183 |
| 12958 | cg19019345 |
| 12959 | cg24126361 |
| 12960 | cg22558680 |
| 12961 | cg07322667 |
| 12962 | cg03267697 |
| 12963 | cg21298056 |
| 12964 | cg18345635 |
| 12965 | cg00484803 |
| 12966 | cg09664215 |
| 12967 | cg20331074 |
| 12968 | cg05642923 |
| 12969 | cg14114133 |
| 12970 | cg16835712 |
| 12971 | cg16032065 |
| 12972 | cg23504246 |
| 12973 | cg04535100 |
| 12974 | cg06420033 |
| 12975 | cg15358152 |
| 12976 | cg25198340 |
| 12977 | cg06569139 |
| 12978 | cg03111156 |
| 12979 | cg08790138 |
| 12980 | cg19858820 |
| 12981 | cg16108121 |
| 12982 | cg17609204 |
| 12983 | cg21845457 |
| 12984 | cg04407652 |
| 12985 | cg12483572 |
| 12986 | cg26189303 |
| 12987 | cg07651551 |
| 12988 | cg25850808 |
| 12989 | cg01567634 |
| 12990 | cg18183817 |
| 12991 | cg06219570 |
| 12992 | cg08128418 |
| 12993 | cg15692788 |
| 12994 | cg15440891 |
| 12995 | cg03135687 |
| 12996 | cg14624145 |
| 12997 | cg23989344 |
| 12998 | cg13475822 |
| 12999 | cg04388919 |
| 13000 | cg10962524 |
| 13001 | cg06119162 |
| 13002 | cg13917968 |
| 13003 | cg19928955 |
| 13004 | cg06598836 |
| 13005 | cg16251671 |
| 13006 | cg27261397 |
| 13007 | cg02019333 |
| 13008 | cg01320433 |
| 13009 | cg18668449 |
| 13010 | cg09929187 |
| 13011 | cg02961394 |
| 13012 | cg18417941 |
| 13013 | cg02104952 |
| 13014 | cg26477488 |
| 13015 | cg09874472 |
| 13016 | cg03359095 |
| 13017 | cg07268332 |
| 13018 | cg24421410 |
| 13019 | cg20040976 |
| 13020 | cg26219680 |
| 13021 | cg11747867 |
| 13022 | cg12850078 |
| 13023 | cg11801481 |
| 13024 | cg27074174 |
| 13025 | cg02825483 |
| 13026 | cg10279314 |
| 13027 | cg16910464 |
| 13028 | cg16362232 |
| 13029 | cg11904978 |
| 13030 | cg26108171 |
| 13031 | cg26589785 |
| 13032 | cg11605972 |
| 13033 | cg15625672 |
| 13034 | cg25318211 |
| 13035 | cg00645405 |
| 13036 | cg15545253 |
| 13037 | cg08062407 |
| 13038 | cg03383916 |
| 13039 | cg12824496 |
| 13040 | cg20819445 |
| 13041 | cg22892437 |
| 13042 | cg26528000 |
| 13043 | cg05834845 |
| 13044 | cg08423755 |
| 13045 | cg04181861 |
| 13046 | cg27552679 |
| 13047 | cg27480054 |
| 13048 | cg09287190 |
| 13049 | cg12461735 |
| 13050 | cg05295536 |
| 13051 | cg12090729 |
| 13052 | cg22883889 |
| 13053 | cg05304729 |
| 13054 | cg11218561 |
| 13055 | cg20770435 |
| 13056 | cg13859958 |
| 13057 | cg16196175 |
| 13058 | cg02031362 |
| 13059 | cg12076765 |
| 13060 | cg19215671 |
| 13061 | cg21192344 |
| 13062 | cg21284772 |
| 13063 | cg16467082 |
| 13064 | cg01676578 |
| 13065 | cg05488369 |
| 13066 | cg19513940 |
| 13067 | cg01485549 |
| 13068 | cg22832557 |
| 13069 | cg21838488 |
| 13070 | cg26062476 |
| 13071 | cg17143376 |
| 13072 | cg19360311 |
| 13073 | cg10321156 |
| 13074 | cg18376497 |
| 13075 | cg05884310 |
| 13076 | cg19359797 |
| 13077 | cg27119030 |
| 13078 | cg02873315 |
| 13079 | cg15282709 |
| 13080 | cg22463409 |
| 13081 | cg06435440 |
| 13082 | cg10936796 |
| 13083 | cg26126371 |
| 13084 | cg19683073 |
| 13085 | cg13110654 |
| 13086 | cg25830612 |
| 13087 | cg02431972 |
| 13088 | cg02658990 |
| 13089 | cg15864926 |
| 13090 | cg00883565 |
| 13091 | cg24224909 |
| 13092 | cg27481559 |
| 13093 | cg07676709 |
| 13094 | cg22196855 |
| 13095 | cg14284257 |
| 13096 | cg16985770 |
| 13097 | cg25944351 |
| 13098 | cg14236118 |
| 13099 | cg01431057 |
| 13100 | cg14315444 |
| 13101 | cg07712330 |
| 13102 | cg09164395 |
| 13103 | cg13583523 |
| 13104 | cg27091631 |
| 13105 | cg13044052 |
| 13106 | cg26469387 |
| 13107 | cg22111118 |
| 13108 | cg09630121 |
| 13109 | cg22731513 |
| 13110 | cg00050482 |
| 13111 | cg00492070 |
| 13112 | cg26351826 |
| 13113 | cg06349780 |
| 13114 | cg27282264 |
| 13115 | cg20308437 |
| 13116 | cg07743451 |
| 13117 | cg16014076 |
| 13118 | cg13944407 |
| 13119 | cg27452471 |
| 13120 | cg00659878 |
| 13121 | cg07018760 |
| 13122 | cg05313794 |
| 13123 | cg16079681 |
| 13124 | cg20067407 |
| 13125 | cg17284844 |
| 13126 | cg06027542 |
| 13127 | cg10338518 |
| 13128 | cg16218009 |
| 13129 | cg23947376 |
| 13130 | cg18518916 |
| 13131 | cg07515196 |
| 13132 | cg16772405 |
| 13133 | cg04972566 |
| 13134 | cg07810884 |
| 13135 | cg10985202 |
| 13136 | cg14468013 |
| 13137 | cg24664346 |
| 13138 | cg23010585 |
| 13139 | cg00776293 |
| 13140 | cg08662619 |
| 13141 | cg11472631 |
| 13142 | cg05319144 |
| 13143 | cg11953254 |
| 13144 | cg06052200 |
| 13145 | cg21846220 |
| 13146 | cg26961166 |
| 13147 | cg04065767 |
| 13148 | cg11186282 |
| 13149 | cg23719713 |
| 13150 | cg17254383 |
| 13151 | cg15424989 |
| 13152 | cg02502786 |
| 13153 | cg17942851 |
| 13154 | cg13398488 |
| 13155 | cg09980803 |
| 13156 | cg06101851 |
| 13157 | cg07725712 |
| 13158 | cg16134082 |
| 13159 | cg21748271 |
| 13160 | cg12811942 |
| 13161 | cg08293368 |
| 13162 | cg07964219 |
| 13163 | cg26171695 |
| 13164 | cg25026992 |
| 13165 | cg25556502 |
| 13166 | cg17829936 |
| 13167 | cg08160063 |
| 13168 | cg17809945 |
| 13169 | cg26975460 |
| 13170 | cg22876086 |
| 13171 | cg04829621 |
| 13172 | cg11350799 |
| 13173 | cg12187747 |
| 13174 | cg10480693 |
| 13175 | cg14721506 |
| 13176 | cg27179172 |
| 13177 | cg23009044 |
| 13178 | cg21475154 |
| 13179 | cg06624036 |
| 13180 | cg24720688 |
| 13181 | cg08461339 |
| 13182 | cg11838471 |
| 13183 | cg00357663 |
| 13184 | cg22081905 |
| 13185 | cg20701461 |
| 13186 | cg12912434 |
| 13187 | cg21515349 |
| 13188 | cg08074000 |
| 13189 | cg15668843 |
| 13190 | cg07495363 |
| 13191 | cg02285102 |
| 13192 | cg14299781 |
| 13193 | cg03589235 |
| 13194 | cg12681948 |
| 13195 | cg22844085 |
| 13196 | cg10244770 |
| 13197 | cg08977725 |
| 13198 | cg06880028 |
| 13199 | cg03163184 |
| 13200 | cg00375608 |
| 13201 | cg13448923 |
| 13202 | cg17435733 |
| 13203 | cg13254480 |
| 13204 | cg06177560 |
| 13205 | cg26600614 |
| 13206 | cg20592017 |
| 13207 | cg27139419 |
| 13208 | cg08152055 |
| 13209 | cg05339793 |
| 13210 | cg08825635 |
| 13211 | cg12305074 |
| 13212 | cg02840890 |
| 13213 | cg13077829 |
| 13214 | cg04439911 |
| 13215 | cg07664183 |
| 13216 | cg12613889 |
| 13217 | cg25093608 |
| 13218 | cg19403014 |
| 13219 | cg12616177 |
| 13220 | cg02311396 |
| 13221 | cg13175780 |
| 13222 | cg26625897 |
| 13223 | cg03832855 |
| 13224 | cg18248023 |
| 13225 | cg03725784 |
| 13226 | cg22972858 |
| 13227 | cg20698667 |
| 13228 | cg15642343 |
| 13229 | cg21890667 |
| 13230 | cg07275979 |
| 13231 | cg14416879 |
| 13232 | cg25482900 |
| 13233 | cg10850215 |
| 13234 | cg19318872 |
| 13235 | cg07361097 |
| 13236 | cg08267789 |
| 13237 | cg02448381 |
| 13238 | cg09046316 |
| 13239 | cg18012012 |
| 13240 | cg27060744 |
| 13241 | cg11333940 |
| 13242 | cg27358936 |
| 13243 | cg16858307 |
| 13244 | cg09981464 |
| 13245 | cg19037373 |
| 13246 | cg01244934 |
| 13247 | cg09096031 |
| 13248 | cg16165165 |
| 13249 | cg09409838 |
| 13250 | cg12965270 |
| 13251 | cg07967246 |
| 13252 | cg00377691 |
| 13253 | cg14508933 |
| 13254 | cg04277677 |
| 13255 | cg03210663 |
| 13256 | cg06194604 |
| 13257 | cg23395553 |
| 13258 | cg19202803 |
| 13259 | cg16289485 |
| 13260 | cg00066854 |
| 13261 | cg20148498 |
| 13262 | cg00821341 |
| 13263 | cg27096043 |
| 13264 | cg00480115 |
| 13265 | cg11039806 |
| 13266 | cg18362003 |
| 13267 | cg01971181 |
| 13268 | cg19876302 |
| 13269 | cg13705241 |
| 13270 | cg17880661 |
| 13271 | cg15539330 |
| 13272 | cg04271938 |
| 13273 | cg26657086 |
| 13274 | cg06169746 |
| 13275 | cg09451320 |
| 13276 | cg10469399 |
| 13277 | cg24104249 |
| 13278 | cg10244340 |
| 13279 | cg03678062 |
| 13280 | cg09879334 |
| 13281 | cg16969116 |
| 13282 | cg26344227 |
| 13283 | cg22805341 |
| 13284 | cg22915785 |
| 13285 | cg04191989 |
| 13286 | cg05877528 |
| 13287 | cg05257196 |
| 13288 | cg04248263 |
| 13289 | cg26929608 |
| 13290 | cg24884214 |
| 13291 | cg04256834 |
| 13292 | cg01593673 |
| 13293 | cg00740479 |
| 13294 | cg19246007 |
| 13295 | cg25069354 |
| 13296 | cg21598751 |
| 13297 | cg17493310 |
| 13298 | cg02699612 |
| 13299 | cg01494994 |
| 13300 | cg26008908 |
| 13301 | cg01063579 |
| 13302 | cg19408398 |
| 13303 | cg00871787 |
| 13304 | cg13264840 |
| 13305 | cg03166835 |
| 13306 | cg13396607 |
| 13307 | cg16454495 |
| 13308 | cg01495714 |
| 13309 | cg22154735 |
| 13310 | cg11445169 |
| 13311 | cg21532801 |
| 13312 | cg07414525 |
| 13313 | cg15917517 |
| 13314 | cg25586990 |
| 13315 | cg26490299 |
| 13316 | cg18277682 |
| 13317 | cg15722977 |
| 13318 | cg03571507 |
| 13319 | cg21533994 |
| 13320 | cg01904793 |
| 13321 | cg20385585 |
| 13322 | cg02122327 |
| 13323 | cg20504084 |
| 13324 | cg04884062 |
| 13325 | cg01627758 |
| 13326 | cg19814116 |
| 13327 | cg12333845 |
| 13328 | cg20082563 |
| 13329 | cg25994907 |
| 13330 | cg24020843 |
| 13331 | cg03044838 |
| 13332 | cg04362989 |
| 13333 | cg03706436 |
| 13334 | cg02312550 |
| 13335 | cg13588599 |
| 13336 | cg24207068 |
| 13337 | cg26240393 |
| 13338 | cg08031982 |
| 13339 | cg20293942 |
| 13340 | cg12816961 |
| 13341 | cg06865026 |
| 13342 | cg23247945 |
| 13343 | cg11362049 |
| 13344 | cg19371800 |
| 13345 | cg19637903 |
| 13346 | cg19803194 |
| 13347 | cg07070348 |
| 13348 | cg16906497 |
| 13349 | cg13433694 |
| 13350 | cg01891736 |
| 13351 | cg19248557 |
| 13352 | cg13493414 |
| 13353 | cg04599026 |
| 13354 | cg05743220 |
| 13355 | cg04426607 |
| 13356 | cg00304185 |
| 13357 | cg24579589 |
| 13358 | cg05230694 |
| 13359 | cg22731432 |
| 13360 | cg23184226 |
| 13361 | cg24765360 |
| 13362 | cg09309117 |
| 13363 | cg22132040 |
| 13364 | cg12685187 |
| 13365 | cg08742575 |
| 13366 | cg17614575 |
| 13367 | cg20589862 |
| 13368 | cg06815976 |
| 13369 | cg05033369 |
| 13370 | cg18196096 |
| 13371 | cg00359633 |
| 13372 | cg01211906 |
| 13373 | cg11536788 |
| 13374 | cg06157991 |
| 13375 | cg15309421 |
| 13376 | cg17155010 |
| 13377 | cg02764093 |
| 13378 | cg17153727 |
| 13379 | cg03315344 |
| 13380 | cg09726198 |
| 13381 | cg25828207 |
| 13382 | cg18092028 |
| 13383 | cg10627955 |
| 13384 | cg19954017 |
| 13385 | cg04084807 |
| 13386 | cg05861420 |
| 13387 | cg08343240 |
| 13388 | cg00012692 |
| 13389 | cg04598224 |
| 13390 | cg21825342 |
| 13391 | cg15713707 |
| 13392 | cg06864398 |
| 13393 | cg04539515 |
| 13394 | cg24726538 |
| 13395 | cg20371046 |
| 13396 | cg06007464 |
| 13397 | cg22743884 |
| 13398 | cg19651184 |
| 13399 | cg03470180 |
| 13400 | cg05007486 |
| 13401 | cg07216436 |
| 13402 | cg19477346 |
| 13403 | cg23192873 |
| 13404 | cg26390944 |
| 13405 | cg12167431 |
| 13406 | cg21809516 |
| 13407 | cg07734149 |
| 13408 | cg17554913 |
| 13409 | cg14169368 |
| 13410 | cg06411730 |
| 13411 | cg06724500 |
| 13412 | cg01515659 |
| 13413 | cg23976600 |
| 13414 | cg07763467 |
| 13415 | cg07134252 |
| 13416 | cg23959206 |
| 13417 | cg00983060 |
| 13418 | cg01103464 |
| 13419 | cg03257387 |
| 13420 | cg18236862 |
| 13421 | cg04002235 |
| 13422 | cg13334579 |
| 13423 | cg26559315 |
| 13424 | cg25331431 |
| 13425 | cg24118251 |
| 13426 | cg21900624 |
| 13427 | cg22791987 |
| 13428 | cg15831060 |
| 13429 | cg00335802 |
| 13430 | cg18355936 |
| 13431 | cg07327358 |
| 13432 | cg15068810 |
| 13433 | cg10595054 |
| 13434 | cg23243355 |
| 13435 | cg14723606 |
| 13436 | cg27380997 |
| 13437 | cg05868813 |
| 13438 | cg09287629 |
| 13439 | cg05884705 |
| 13440 | cg14714797 |
| 13441 | cg03331978 |
| 13442 | cg01892994 |
| 13443 | cg20734611 |
| 13444 | cg21333674 |
| 13445 | cg25835351 |
| 13446 | cg06117891 |
| 13447 | cg14254720 |
| 13448 | cg23646367 |
| 13449 | cg18229049 |
| 13450 | cg03716852 |
| 13451 | cg05947572 |
| 13452 | cg14619099 |
| 13453 | cg05109008 |
| 13454 | cg10440696 |
| 13455 | cg03031839 |
| 13456 | cg20005742 |
| 13457 | cg27206026 |
| 13458 | cg21799168 |
| 13459 | cg19475870 |
| 13460 | cg14996263 |
| 13461 | cg13431037 |
| 13462 | cg05769153 |
| 13463 | cg19169288 |
| 13464 | cg20741105 |
| 13465 | cg12373597 |
| 13466 | cg04493169 |
| 13467 | cg13772160 |
| 13468 | cg06557421 |
| 13469 | cg05758793 |
| 13470 | cg18945630 |
| 13471 | cg09121533 |
| 13472 | cg22598458 |
| 13473 | cg06649645 |
| 13474 | cg04142929 |
| 13475 | cg22708087 |
| 13476 | cg24142603 |
| 13477 | cg01553433 |
| 13478 | cg27608102 |
| 13479 | cg07526904 |
| 13480 | cg14895298 |
| 13481 | cg22373181 |
| 13482 | cg10813539 |
| 13483 | cg19814552 |
| 13484 | cg16966992 |
| 13485 | cg18172495 |
| 13486 | cg14733042 |
| 13487 | cg00364304 |
| 13488 | cg26980034 |
| 13489 | cg10290737 |
| 13490 | cg25818689 |
| 13491 | cg15089370 |
| 13492 | cg23055986 |
| 13493 | cg06300886 |
| 13494 | cg08220243 |
| 13495 | cg27079726 |
| 13496 | cg19614379 |
| 13497 | cg04566037 |
| 13498 | cg25256067 |
| 13499 | cg11774116 |
| 13500 | cg13058321 |
| 13501 | cg23704517 |
| 13502 | cg22401573 |
| 13503 | cg22758454 |
| 13504 | cg16725984 |
| 13505 | cg11171995 |
| 13506 | cg22746337 |
| 13507 | cg10203922 |
| 13508 | cg01318441 |
| 13509 | cg25052523 |
| 13510 | cg04957729 |
| 13511 | cg04493942 |
| 13512 | cg16629695 |
| 13513 | cg01306062 |
| 13514 | cg02729866 |
| 13515 | cg14771419 |
| 13516 | cg25415474 |
| 13517 | cg12728517 |
| 13518 | cg05486647 |
| 13519 | cg05133593 |
| 13520 | cg10320259 |
| 13521 | cg23867923 |
| 13522 | cg00946598 |
| 13523 | cg05425114 |
| 13524 | cg01047555 |
| 13525 | cg15892341 |
| 13526 | cg14167415 |
| 13527 | cg14016757 |
| 13528 | cg24362778 |
| 13529 | cg15618908 |
| 13530 | cg09450907 |
| 13531 | cg00209038 |
| 13532 | cg17954205 |
| 13533 | cg24916768 |
| 13534 | cg18696822 |
| 13535 | cg14784405 |
| 13536 | cg02972749 |
| 13537 | cg04848359 |
| 13538 | cg05164163 |
| 13539 | cg11701819 |
| 13540 | cg27414481 |
| 13541 | cg05168279 |
| 13542 | cg15824094 |
| 13543 | cg26382679 |
| 13544 | cg08522085 |
| 13545 | cg11104088 |
| 13546 | cg17521050 |
| 13547 | cg03582047 |
| 13548 | cg23615393 |
| 13549 | cg21668786 |
| 13550 | cg07453440 |
| 13551 | cg19913677 |
| 13552 | cg26386673 |
| 13553 | cg22349396 |
| 13554 | cg04892677 |
| 13555 | cg14667123 |
| 13556 | cg13338454 |
| 13557 | cg17703000 |
| 13558 | cg20883195 |
| 13559 | cg10482508 |
| 13560 | cg16122424 |
| 13561 | cg02097137 |
| 13562 | cg23115907 |
| 13563 | cg01530154 |
| 13564 | cg16860739 |
| 13565 | cg08040395 |
| 13566 | cg04956653 |
| 13567 | cg22319147 |
| 13568 | cg13264234 |
| 13569 | cg14277161 |
| 13570 | cg21176127 |
| 13571 | cg08201311 |
| 13572 | cg02121529 |
| 13573 | cg24466939 |
| 13574 | cg07212470 |
| 13575 | cg17602126 |
| 13576 | cg21555783 |
| 13577 | cg02055540 |
| 13578 | cg03945763 |
| 13579 | cg16960046 |
| 13580 | cg04129754 |
| 13581 | cg08083082 |
| 13582 | cg13912545 |
| 13583 | cg01239922 |
| 13584 | cg21171335 |
| 13585 | cg14753355 |
| 13586 | cg02887254 |
| 13587 | cg12863924 |
| 13588 | cg09119805 |
| 13589 | cg09973284 |
| 13590 | cg24635943 |
| 13591 | cg07824555 |
| 13592 | cg24154839 |
| 13593 | cg13123591 |
| 13594 | cg07784042 |
| 13595 | cg23007665 |
| 13596 | cg09536368 |
| 13597 | cg16391678 |
| 13598 | cg06291627 |
| 13599 | cg02865101 |
| 13600 | cg19078878 |
| 13601 | cg13203834 |
| 13602 | cg21710377 |
| 13603 | cg04075191 |
| 13604 | cg15473092 |
| 13605 | cg01615382 |
| 13606 | cg00278359 |
| 13607 | cg24093342 |
| 13608 | cg12984178 |
| 13609 | cg22316093 |
| 13610 | cg13809116 |
| 13611 | cg24602417 |
| 13612 | cg13398482 |
| 13613 | cg02578087 |
| 13614 | cg04494534 |
| 13615 | cg16323603 |
| 13616 | cg08455778 |
| 13617 | cg07837433 |
| 13618 | cg26261627 |
| 13619 | cg25216704 |
| 13620 | cg23268208 |
| 13621 | cg14912355 |
| 13622 | cg11970602 |
| 13623 | cg11855643 |
| 13624 | cg21797564 |
| 13625 | cg21405376 |
| 13626 | cg19439043 |
| 13627 | cg15289643 |
| 13628 | cg19579500 |
| 13629 | cg26496902 |
| 13630 | cg17004353 |
| 13631 | cg02227339 |
| 13632 | cg23264234 |
| 13633 | cg16483329 |
| 13634 | cg05315321 |
| 13635 | cg00868131 |
| 13636 | cg20723815 |
| 13637 | cg05240760 |
| 13638 | cg18000029 |
| 13639 | cg10764984 |
| 13640 | cg08959575 |
| 13641 | cg04680436 |
| 13642 | cg09755551 |
| 13643 | cg10972686 |
| 13644 | cg13627968 |
| 13645 | cg27283592 |
| 13646 | cg00642274 |
| 13647 | cg18863087 |
| 13648 | cg25857194 |
| 13649 | cg24059418 |
| 13650 | cg02813126 |
| 13651 | cg08834436 |
| 13652 | cg01887148 |
| 13653 | cg17046586 |
| 13654 | cg07197092 |
| 13655 | cg14520280 |
| 13656 | cg14480530 |
| 13657 | cg24135211 |
| 13658 | cg27175024 |
| 13659 | cg10103850 |
| 13660 | cg06138439 |
| 13661 | cg04844987 |
| 13662 | cg08087868 |
| 13663 | cg19815376 |
| 13664 | cg22072216 |
| 13665 | cg00341843 |
| 13666 | cg15328703 |
| 13667 | cg08913189 |
| 13668 | cg04991214 |
| 13669 | cg07220903 |
| 13670 | cg20737812 |
| 13671 | cg14003022 |
| 13672 | cg11416384 |
| 13673 | cg24730915 |
| 13674 | cg23383832 |
| 13675 | cg11200656 |
| 13676 | cg16608935 |
| 13677 | cg21217540 |
| 13678 | cg14876280 |
| 13679 | cg06107434 |
| 13680 | cg02983592 |
| 13681 | cg10540488 |
| 13682 | cg00229359 |
| 13683 | cg14838248 |
| 13684 | cg22720885 |
| 13685 | cg19075225 |
| 13686 | cg00455331 |
| 13687 | cg08991210 |
| 13688 | cg11026385 |
| 13689 | cg09812070 |
| 13690 | cg22462726 |
| 13691 | cg24882882 |
| 13692 | cg14571491 |
| 13693 | cg05672223 |
| 13694 | cg00667081 |
| 13695 | cg02872491 |
| 13696 | cg13069353 |
| 13697 | cg04367164 |
| 13698 | cg01892656 |
| 13699 | cg13366863 |
| 13700 | cg16204414 |
| 13701 | cg13791131 |
| 13702 | cg20582188 |
| 13703 | cg09127448 |
| 13704 | cg09706574 |
| 13705 | cg16782161 |
| 13706 | cg26554547 |
| 13707 | cg06905679 |
| 13708 | cg01802506 |
| 13709 | cg06679270 |
| 13710 | cg00128482 |
| 13711 | cg23012519 |
| 13712 | cg12164321 |
| 13713 | cg10196558 |
| 13714 | cg26296101 |
| 13715 | cg00224471 |
| 13716 | cg04892836 |
| 13717 | cg16428374 |
| 13718 | cg18054172 |
| 13719 | cg08158233 |
| 13720 | cg25383503 |
| 13721 | cg20940675 |
| 13722 | cg11253416 |
| 13723 | cg13739680 |
| 13724 | cg15985115 |
| 13725 | cg01926238 |
| 13726 | cg01568519 |
| 13727 | cg12419864 |
| 13728 | cg27535677 |
| 13729 | cg04331189 |
| 13730 | cg00207125 |
| 13731 | cg06213635 |
| 13732 | cg08111446 |
| 13733 | cg01119903 |
| 13734 | cg11945668 |
| 13735 | cg08356028 |
| 13736 | cg02187937 |
| 13737 | cg00613284 |
| 13738 | cg20173717 |
| 13739 | cg14853772 |
| 13740 | cg09847528 |
| 13741 | cg13593913 |
| 13742 | cg17185586 |
| 13743 | cg07273304 |
| 13744 | cg19449173 |
| 13745 | cg02241678 |
| 13746 | cg07315010 |
| 13747 | cg07405796 |
| 13748 | cg16609181 |
| 13749 | cg23913077 |
| 13750 | cg03548384 |
| 13751 | cg05569624 |
| 13752 | cg11635197 |
| 13753 | cg03298789 |
| 13754 | cg15173475 |
| 13755 | cg04797681 |
| 13756 | cg14021170 |
| 13757 | cg09198462 |
| 13758 | cg24550149 |
| 13759 | cg03328892 |
| 13760 | cg19288362 |
| 13761 | cg18613786 |
| 13762 | cg20747380 |
| 13763 | cg13154147 |
| 13764 | cg08916985 |
| 13765 | cg10611692 |
| 13766 | cg03530155 |
| 13767 | cg10918326 |
| 13768 | cg16491132 |
| 13769 | cg12695286 |
| 13770 | cg16845363 |
| 13771 | cg06174330 |
| 13772 | cg11301046 |
| 13773 | cg07107661 |
| 13774 | cg00405942 |
| 13775 | cg25016677 |
| 13776 | cg00157932 |
| 13777 | cg00445518 |
| 13778 | cg12964546 |
| 13779 | cg14995262 |
| 13780 | cg20272146 |
| 13781 | cg08697732 |
| 13782 | cg01191259 |
| 13783 | cg22077406 |
| 13784 | cg01621764 |
| 13785 | cg12335110 |
| 13786 | cg18722847 |
| 13787 | cg14483431 |
| 13788 | cg13160181 |
| 13789 | cg25837126 |
| 13790 | cg27459180 |
| 13791 | cg06602545 |
| 13792 | cg23221741 |
| 13793 | cg01512626 |
| 13794 | cg15645203 |
| 13795 | cg00400217 |
| 13796 | cg09796529 |
| 13797 | cg09045979 |
| 13798 | cg15016481 |
| 13799 | cg06333233 |
| 13800 | cg06621744 |
| 13801 | cg08985293 |
| 13802 | cg15951935 |
| 13803 | cg20084184 |
| 13804 | cg24110063 |
| 13805 | cg18375642 |
| 13806 | cg12322235 |
| 13807 | cg11696200 |
| 13808 | cg11558203 |
| 13809 | cg02142792 |
| 13810 | cg05641535 |
| 13811 | cg23230478 |
| 13812 | cg07825931 |
| 13813 | cg06153448 |
| 13814 | cg24895173 |
| 13815 | cg22626169 |
| 13816 | cg18199741 |
| 13817 | cg11297458 |
| 13818 | cg19947214 |
| 13819 | cg23356674 |
| 13820 | cg15795081 |
| 13821 | cg24257776 |
| 13822 | cg14452599 |
| 13823 | cg14621763 |
| 13824 | cg05215127 |
| 13825 | cg25374359 |
| 13826 | cg27479358 |
| 13827 | cg22034189 |
| 13828 | cg15799036 |
| 13829 | cg20016411 |
| 13830 | cg14607011 |
| 13831 | cg17589866 |
| 13832 | cg02540460 |
| 13833 | cg04060873 |
| 13834 | cg17414101 |
| 13835 | cg01110759 |
| 13836 | cg15179566 |
| 13837 | cg20207567 |
| 13838 | cg06201841 |
| 13839 | cg19049502 |
| 13840 | cg24092651 |
| 13841 | cg00953158 |
| 13842 | cg22605919 |
| 13843 | cg01387792 |
| 13844 | cg04036537 |
| 13845 | cg27023360 |
| 13846 | cg20270653 |
| 13847 | cg06866240 |
| 13848 | cg07781364 |
| 13849 | cg19557723 |
| 13850 | cg04166006 |
| 13851 | cg20148850 |
| 13852 | cg15149437 |
| 13853 | cg01373217 |
| 13854 | cg11440915 |
| 13855 | cg15315565 |
| 13856 | cg01682285 |
| 13857 | cg06725662 |
| 13858 | cg26881513 |
| 13859 | cg13567205 |
| 13860 | cg03417681 |
| 13861 | cg12684113 |
| 13862 | cg12760869 |
| 13863 | cg15920942 |
| 13864 | cg23010096 |
| 13865 | cg09941884 |
| 13866 | cg13579267 |
| 13867 | cg19311244 |
| 13868 | cg16555269 |
| 13869 | cg17470942 |
| 13870 | cg13923516 |
| 13871 | cg10662345 |
| 13872 | cg02582963 |
| 13873 | cg11774233 |
| 13874 | cg08516535 |
| 13875 | cg23221603 |
| 13876 | cg03267730 |
| 13877 | cg04363092 |
| 13878 | cg18560936 |
| 13879 | cg11948071 |
| 13880 | cg20029849 |
| 13881 | cg15574509 |
| 13882 | cg23003783 |
| 13883 | cg08971217 |
| 13884 | cg10928544 |
| 13885 | cg23657299 |
| 13886 | cg13354811 |
| 13887 | cg00364287 |
| 13888 | cg02432003 |
| 13889 | cg24828620 |
| 13890 | cg06655810 |
| 13891 | cg12198977 |
| 13892 | cg16396054 |
| 13893 | cg06835459 |
| 13894 | cg09677560 |
| 13895 | cg03989507 |
| 13896 | cg22665096 |
| 13897 | cg05587380 |
| 13898 | cg26125020 |
| 13899 | cg25171969 |
| 13900 | cg19023847 |
| 13901 | cg09059267 |
| 13902 | cg16178091 |
| 13903 | cg08547072 |
| 13904 | cg26083822 |
| 13905 | cg21307295 |
| 13906 | cg03546689 |
| 13907 | cg05634218 |
| 13908 | cg17965318 |
| 13909 | cg14039865 |
| 13910 | cg09595479 |
| 13911 | cg19280586 |
| 13912 | cg14793110 |
| 13913 | cg09352285 |
| 13914 | cg07472704 |
| 13915 | cg20300776 |
| 13916 | cg17663186 |
| 13917 | cg11539857 |
| 13918 | cg26358968 |
| 13919 | cg12761144 |
| 13920 | cg10546210 |
| 13921 | cg05816868 |
| 13922 | cg13620119 |
| 13923 | cg21913319 |
| 13924 | cg14590806 |
| 13925 | cg13293488 |
| 13926 | cg07852628 |
| 13927 | cg16698623 |
| 13928 | cg07837359 |
| 13929 | cg24005842 |
| 13930 | cg06222414 |
| 13931 | cg24587301 |
| 13932 | cg18773807 |
| 13933 | cg26029453 |
| 13934 | cg18750210 |
| 13935 | cg12219334 |
| 13936 | cg21171746 |
| 13937 | cg22061529 |
| 13938 | cg26531023 |
| 13939 | cg16248515 |
| 13940 | cg19219220 |
| 13941 | cg17854471 |
| 13942 | cg25556122 |
| 13943 | cg03436326 |
| 13944 | cg18958580 |
| 13945 | cg05219445 |
| 13946 | cg07898264 |
| 13947 | cg07016546 |
| 13948 | cg17334389 |
| 13949 | cg17828057 |
| 13950 | cg25174412 |
| 13951 | cg05385881 |
| 13952 | cg08701550 |
| 13953 | cg22827300 |
| 13954 | cg26460647 |
| 13955 | cg20514322 |
| 13956 | cg15256219 |
| 13957 | cg19555318 |
| 13958 | cg07412254 |
| 13959 | cg19241220 |
| 13960 | cg22115892 |
| 13961 | cg14684255 |
| 13962 | cg15000813 |
| 13963 | cg08577953 |
| 13964 | cg17458659 |
| 13965 | cg25181651 |
| 13966 | cg15883287 |
| 13967 | cg03474889 |
| 13968 | cg22514682 |
| 13969 | cg13331550 |
| 13970 | cg06099014 |
| 13971 | cg25494270 |
| 13972 | cg00010954 |
| 13973 | cg02742133 |
| 13974 | cg14575739 |
| 13975 | cg25078225 |
| 13976 | cg16831085 |
| 13977 | cg02721536 |
| 13978 | cg20877076 |
| 13979 | cg08971562 |
| 13980 | cg20987924 |
| 13981 | cg14163722 |
| 13982 | cg03399477 |
| 13983 | cg20845664 |
| 13984 | cg07003097 |
| 13985 | cg09437479 |
| 13986 | cg25007283 |
| 13987 | cg26271170 |
| 13988 | cg26979504 |
| 13989 | cg06064945 |
| 13990 | cg05053752 |
| 13991 | cg18796695 |
| 13992 | cg24910887 |
| 13993 | cg16533864 |
| 13994 | cg00293718 |
| 13995 | cg06906827 |
| 13996 | cg02150060 |
| 13997 | cg20152501 |
| 13998 | cg25119077 |
| 13999 | cg07731828 |
| 14000 | cg13235059 |
| 14001 | cg09214412 |
| 14002 | cg07516685 |
| 14003 | cg08196968 |
| 14004 | cg19944656 |
| 14005 | cg03433030 |
| 14006 | cg05526269 |
| 14007 | cg05945782 |
| 14008 | cg04498198 |
| 14009 | cg14200594 |
| 14010 | cg22433759 |
| 14011 | cg16684084 |
| 14012 | cg18301423 |
| 14013 | cg15025140 |
| 14014 | cg04672622 |
| 14015 | cg12035237 |
| 14016 | cg04048259 |
| 14017 | cg09581137 |
| 14018 | cg05812024 |
| 14019 | cg13058214 |
| 14020 | cg19921158 |
| 14021 | cg16984927 |
| 14022 | cg16337430 |
| 14023 | cg22995042 |
| 14024 | cg17516271 |
| 14025 | cg12964261 |
| 14026 | cg02921382 |
| 14027 | cg22876250 |
| 14028 | cg10359304 |
| 14029 | cg14540247 |
| 14030 | cg19192065 |
| 14031 | cg01275038 |
| 14032 | cg11914037 |
| 14033 | cg16331883 |
| 14034 | cg07263322 |
| 14035 | cg18176300 |
| 14036 | cg05089931 |
| 14037 | cg17720118 |
| 14038 | cg10072464 |
| 14039 | cg05472536 |
| 14040 | cg18548879 |
| 14041 | cg07679370 |
| 14042 | cg08829007 |
| 14043 | cg20156679 |
| 14044 | cg27531336 |
| 14045 | cg18353572 |
| 14046 | cg11830461 |
| 14047 | cg14836839 |
| 14048 | cg06382922 |
| 14049 | cg09757250 |
| 14050 | cg03368627 |
| 14051 | cg23975326 |
| 14052 | cg22559034 |
| 14053 | cg01082072 |
| 14054 | cg08108224 |
| 14055 | cg08065240 |
| 14056 | cg12252135 |
| 14057 | cg08531940 |
| 14058 | cg01525816 |
| 14059 | cg01939082 |
| 14060 | cg25863391 |
| 14061 | cg00435273 |
| 14062 | cg25002649 |
| 14063 | cg16612097 |
| 14064 | cg25566301 |
| 14065 | cg18403193 |
| 14066 | cg12824378 |
| 14067 | cg15540985 |
| 14068 | cg02650080 |
| 14069 | cg20003893 |
| 14070 | cg06665890 |
| 14071 | cg05606376 |
| 14072 | cg22680931 |
| 14073 | cg07899016 |
| 14074 | cg07042301 |
| 14075 | cg14781778 |
| 14076 | cg06002638 |
| 14077 | cg06687318 |
| 14078 | cg26426910 |
| 14079 | cg26977633 |
| 14080 | cg07109131 |
| 14081 | cg15711727 |
| 14082 | cg16949914 |
| 14083 | cg11173246 |
| 14084 | cg22444445 |
| 14085 | cg18683500 |
| 14086 | cg22997543 |
| 14087 | cg04221650 |
| 14088 | cg23298862 |
| 14089 | cg03856044 |
| 14090 | cg24694502 |
| 14091 | cg10837229 |
| 14092 | cg07646791 |
| 14093 | cg03839542 |
| 14094 | cg23885932 |
| 14095 | cg01228357 |
| 14096 | cg00612625 |
| 14097 | cg25048987 |
| 14098 | cg14570280 |
| 14099 | cg04159760 |
| 14100 | cg09505846 |
| 14101 | cg09134760 |
| 14102 | cg24187216 |
| 14103 | cg17239236 |
| 14104 | cg19571285 |
| 14105 | cg19295951 |
| 14106 | cg10224245 |
| 14107 | cg20824707 |
| 14108 | cg25316812 |
| 14109 | cg01361990 |
| 14110 | cg05595134 |
| 14111 | cg13090478 |
| 14112 | cg09110374 |
| 14113 | cg24556792 |
| 14114 | cg21336122 |
| 14115 | cg15898536 |
| 14116 | cg23856413 |
| 14117 | cg02603588 |
| 14118 | cg19853612 |
| 14119 | cg07354458 |
| 14120 | cg10816539 |
| 14121 | cg00277753 |
| 14122 | cg07869907 |
| 14123 | cg21387325 |
| 14124 | cg18377670 |
| 14125 | cg08356564 |
| 14126 | cg00935894 |
| 14127 | cg02955354 |
| 14128 | cg03281561 |
| 14129 | cg04554576 |
| 14130 | cg05406609 |
| 14131 | cg13099330 |
| 14132 | cg14883569 |
| 14133 | cg27353899 |
| 14134 | cg19380996 |
| 14135 | cg25823128 |
| 14136 | cg08186275 |
| 14137 | cg13047720 |
| 14138 | cg25142500 |
| 14139 | cg02079273 |
| 14140 | cg26769493 |
| 14141 | cg06505619 |
| 14142 | cg06148145 |
| 14143 | cg01091951 |
| 14144 | cg02730156 |
| 14145 | cg14659346 |
| 14146 | cg00536792 |
| 14147 | cg18560789 |
| 14148 | cg09515862 |
| 14149 | cg02584257 |
| 14150 | cg17294440 |
| 14151 | cg16032841 |
| 14152 | cg08246090 |
| 14153 | cg26342059 |
| 14154 | cg20592766 |
| 14155 | cg01789829 |
| 14156 | cg18944451 |
| 14157 | cg04117693 |
| 14158 | cg18702278 |
| 14159 | cg17527673 |
| 14160 | cg08823209 |
| 14161 | cg23085466 |
| 14162 | cg14584278 |
| 14163 | cg26133164 |
| 14164 | cg05058246 |
| 14165 | cg21835643 |
| 14166 | cg01736680 |
| 14167 | cg24885297 |
| 14168 | cg10850930 |
| 14169 | cg18757077 |
| 14170 | cg00395667 |
| 14171 | cg20447920 |
| 14172 | cg13534503 |
| 14173 | cg27314002 |
| 14174 | cg07377195 |
| 14175 | cg07823169 |
| 14176 | cg04228305 |
| 14177 | cg27583988 |
| 14178 | cg22419134 |
| 14179 | cg23874561 |
| 14180 | cg06530338 |
| 14181 | cg05875719 |
| 14182 | cg13726901 |
| 14183 | cg23933458 |
| 14184 | cg15202934 |
| 14185 | cg12303318 |
| 14186 | cg20633402 |
| 14187 | cg01414065 |
| 14188 | cg06033789 |
| 14189 | cg21929464 |
| 14190 | cg27152903 |
| 14191 | cg06487870 |
| 14192 | cg00553149 |
| 14193 | cg00871610 |
| 14194 | cg20127496 |
| 14195 | cg20507276 |
| 14196 | cg07891904 |
| 14197 | cg16776967 |
| 14198 | cg02532341 |
| 14199 | cg27561907 |
| 14200 | cg26791400 |
| 14201 | cg07908047 |
| 14202 | cg15037541 |
| 14203 | cg14094646 |
| 14204 | cg16325826 |
| 14205 | cg21628877 |
| 14206 | cg25812068 |
| 14207 | cg12386646 |
| 14208 | cg04677723 |
| 14209 | cg02392976 |
| 14210 | cg20376082 |
| 14211 | cg00959309 |
| 14212 | cg02646779 |
| 14213 | cg02616886 |
| 14214 | cg16154810 |
| 14215 | cg10087172 |
| 14216 | cg13673023 |
| 14217 | cg24994863 |
| 14218 | cg14994777 |
| 14219 | cg00746886 |
| 14220 | cg13347284 |
| 14221 | cg13858900 |
| 14222 | cg23858537 |
| 14223 | cg06495547 |
| 14224 | cg23326536 |
| 14225 | cg26281685 |
| 14226 | cg12523066 |
| 14227 | cg23530594 |
| 14228 | cg13637104 |
| 14229 | cg08987380 |
| 14230 | cg14074184 |
| 14231 | cg22048405 |
| 14232 | cg20601412 |
| 14233 | cg05191651 |
| 14234 | cg04223044 |
| 14235 | cg09676794 |
| 14236 | cg02483449 |
| 14237 | cg00867835 |
| 14238 | cg11199066 |
| 14239 | cg03510486 |
| 14240 | cg14373126 |
| 14241 | cg23061396 |
| 14242 | cg24805373 |
| 14243 | cg22040162 |
| 14244 | cg20744387 |
| 14245 | cg07223926 |
| 14246 | cg25914433 |
| 14247 | cg00771642 |
| 14248 | cg21547037 |
| 14249 | cg03332623 |
| 14250 | cg19300549 |
| 14251 | cg03285577 |
| 14252 | cg17357892 |
| 14253 | cg07986019 |
| 14254 | cg11697953 |
| 14255 | cg00479463 |
| 14256 | cg10151281 |
| 14257 | cg03912703 |
| 14258 | cg09159993 |
| 14259 | cg14104035 |
| 14260 | cg04267658 |
| 14261 | cg10905220 |
| 14262 | cg13227481 |
| 14263 | cg11503274 |
| 14264 | cg05665581 |
| 14265 | cg05372113 |
| 14266 | cg01576149 |
| 14267 | cg13313047 |
| 14268 | cg21086329 |
| 14269 | cg02288805 |
| 14270 | cg22977055 |
| 14271 | cg24337025 |
| 14272 | cg01825213 |
| 14273 | cg26040809 |
| 14274 | cg08866991 |
| 14275 | cg04608991 |
| 14276 | cg19788754 |
| 14277 | cg21501207 |
| 14278 | cg00282989 |
| 14279 | cg10846615 |
| 14280 | cg25933645 |
| 14281 | cg14383174 |
| 14282 | cg22967396 |
| 14283 | cg06569266 |
| 14284 | cg10122877 |
| 14285 | cg00017489 |
| 14286 | cg05564182 |
| 14287 | cg25060890 |
| 14288 | cg13842421 |
| 14289 | cg09962952 |
| 14290 | cg06805707 |
| 14291 | cg07131210 |
| 14292 | cg01711961 |
| 14293 | cg03265495 |
| 14294 | cg16627764 |
| 14295 | cg06146973 |
| 14296 | cg07934856 |
| 14297 | cg13304638 |
| 14298 | cg17989736 |
| 14299 | cg12456833 |
| 14300 | cg10935968 |
| 14301 | cg20608085 |
| 14302 | cg02306236 |
| 14303 | cg02794434 |
| 14304 | cg18016034 |
| 14305 | cg05026415 |
| 14306 | cg19478682 |
| 14307 | cg22729821 |
| 14308 | cg16539741 |
| 14309 | cg01396302 |
| 14310 | cg12417938 |
| 14311 | cg04427498 |
| 14312 | cg27610491 |
| 14313 | cg11771151 |
| 14314 | cg00376553 |
| 14315 | cg04300684 |
| 14316 | cg06754312 |
| 14317 | cg12174736 |
| 14318 | cg16627782 |
| 14319 | cg14191454 |
| 14320 | cg20814021 |
| 14321 | cg12512906 |
| 14322 | cg01513078 |
| 14323 | cg25206951 |
| 14324 | cg01315201 |
| 14325 | cg10825315 |
| 14326 | cg16534850 |
| 14327 | cg10866060 |
| 14328 | cg08189993 |
| 14329 | cg10902548 |
| 14330 | cg20530101 |
| 14331 | cg24387367 |
| 14332 | cg12368169 |
| 14333 | cg14992311 |
| 14334 | cg09526712 |
| 14335 | cg14503395 |
| 14336 | cg11979414 |
| 14337 | cg25205705 |
| 14338 | cg27366261 |
| 14339 | cg10502121 |
| 14340 | cg20381843 |
| 14341 | cg04681489 |
| 14342 | cg20707086 |
| 14343 | cg25475729 |
| 14344 | cg07702486 |
| 14345 | cg22780193 |
| 14346 | cg16719560 |
| 14347 | cg20673652 |
| 14348 | cg15990972 |
| 14349 | cg11026647 |
| 14350 | cg10242160 |
| 14351 | cg12232463 |
| 14352 | cg02300143 |
| 14353 | cg20888615 |
| 14354 | cg03468882 |
| 14355 | cg10440639 |
| 14356 | cg01232121 |
| 14357 | cg03103886 |
| 14358 | cg26258454 |
| 14359 | cg08772942 |
| 14360 | cg07370683 |
| 14361 | cg26437845 |
| 14362 | cg20841047 |
| 14363 | cg16798878 |
| 14364 | cg06768010 |
| 14365 | cg09592244 |
| 14366 | cg21962374 |
| 14367 | cg15326863 |
| 14368 | cg27039312 |
| 14369 | cg14062050 |
| 14370 | cg23716141 |
| 14371 | cg01733619 |
| 14372 | cg04832245 |
| 14373 | cg20525449 |
| 14374 | cg20857612 |
| 14375 | cg21918548 |
| 14376 | cg06906869 |
| 14377 | cg26033937 |
| 14378 | cg17939444 |
| 14379 | cg16879993 |
| 14380 | cg18207048 |
| 14381 | cg18740968 |
| 14382 | cg09135986 |
| 14383 | cg13035571 |
| 14384 | cg17943995 |
| 14385 | cg07984515 |
| 14386 | cg08025585 |
| 14387 | cg00583861 |
| 14388 | cg16695258 |
| 14389 | cg16913600 |
| 14390 | cg12774644 |
| 14391 | cg24271538 |
| 14392 | cg06206989 |
| 14393 | cg00637173 |
| 14394 | cg10910400 |
| 14395 | cg19092096 |
| 14396 | cg17649516 |
| 14397 | cg16536718 |
| 14398 | cg12480674 |
| 14399 | cg01322214 |
| 14400 | cg18899220 |
| 14401 | cg01786306 |
| 14402 | cg21837920 |
| 14403 | cg12801917 |
| 14404 | cg00481884 |
| 14405 | cg18603580 |
| 14406 | cg26908959 |
| 14407 | cg00182761 |
| 14408 | cg01797501 |
| 14409 | cg22389491 |
| 14410 | cg00163510 |
| 14411 | cg05303559 |
| 14412 | cg10132287 |
| 14413 | cg00008075 |
| 14414 | cg18478850 |
| 14415 | cg13982366 |
| 14416 | cg24833277 |
| 14417 | cg20660182 |
| 14418 | cg27026942 |
| 14419 | cg09244071 |
| 14420 | cg11681428 |
| 14421 | cg06438204 |
| 14422 | cg14273083 |
| 14423 | cg03614159 |
| 14424 | cg09577324 |
| 14425 | cg03448915 |
| 14426 | cg22753611 |
| 14427 | cg04945312 |
| 14428 | cg11558005 |
| 14429 | cg12309703 |
| 14430 | cg14352412 |
| 14431 | cg05822940 |
| 14432 | cg24639098 |
| 14433 | cg19222405 |
| 14434 | cg16127496 |
| 14435 | cg19632442 |
| 14436 | cg10038185 |
| 14437 | cg00821396 |
| 14438 | cg18675146 |
| 14439 | cg21086113 |
| 14440 | cg11961590 |
| 14441 | cg05027713 |
| 14442 | cg18155700 |
| 14443 | cg12075928 |
| 14444 | cg01513441 |
| 14445 | cg01679017 |
| 14446 | cg02724965 |
| 14447 | cg06878548 |
| 14448 | cg12827852 |
| 14449 | cg27528222 |
| 14450 | cg22251569 |
| 14451 | cg13311440 |
| 14452 | cg02093229 |
| 14453 | cg14780183 |
| 14454 | cg03720762 |
| 14455 | cg08376299 |
| 14456 | cg13688966 |
| 14457 | cg19818890 |
| 14458 | cg06659436 |
| 14459 | cg24652919 |
| 14460 | cg18863237 |
| 14461 | cg07469151 |
| 14462 | cg21579312 |
| 14463 | cg26282761 |
| 14464 | cg18637858 |
| 14465 | cg02101451 |
| 14466 | cg11248078 |
| 14467 | cg02370884 |
| 14468 | cg03793270 |
| 14469 | cg20450471 |
| 14470 | cg05912212 |
| 14471 | cg19278170 |
| 14472 | cg09545871 |
| 14473 | cg05768824 |
| 14474 | cg25741116 |
| 14475 | cg22458304 |
| 14476 | cg20298802 |
| 14477 | cg10807724 |
| 14478 | cg11295724 |
| 14479 | cg10011018 |
| 14480 | cg05357152 |
| 14481 | cg19748424 |
| 14482 | cg20540723 |
| 14483 | cg15710574 |
| 14484 | cg17080227 |
| 14485 | cg05380935 |
| 14486 | cg00327031 |
| 14487 | cg10973622 |
| 14488 | cg15042891 |
| 14489 | cg02569698 |
| 14490 | cg16482344 |
| 14491 | cg19701577 |
| 14492 | cg11135072 |
| 14493 | cg08164046 |
| 14494 | cg27504195 |
| 14495 | cg21890241 |
| 14496 | cg23522522 |
| 14497 | cg15653173 |
| 14498 | cg07919476 |
| 14499 | cg25526519 |
| 14500 | cg01306824 |
| 14501 | cg12017745 |
| 14502 | cg14954309 |
| 14503 | cg10975001 |
| 14504 | cg00949794 |
| 14505 | cg08227282 |
| 14506 | cg19171393 |
| 14507 | cg10246081 |
| 14508 | cg07492937 |
| 14509 | cg00044107 |
| 14510 | cg24974737 |
| 14511 | cg05394783 |
| 14512 | cg03061435 |
| 14513 | cg08439728 |
| 14514 | cg06195354 |
| 14515 | cg26988138 |
| 14516 | cg17652428 |
| 14517 | cg08048268 |
| 14518 | cg12923919 |
| 14519 | cg02088237 |
| 14520 | cg00324161 |
| 14521 | cg24870273 |
| 14522 | cg22586726 |
| 14523 | cg20639646 |
| 14524 | cg10452448 |
| 14525 | cg05001111 |
| 14526 | cg16018377 |
| 14527 | cg07939157 |
| 14528 | cg02539082 |
| 14529 | cg23894309 |
| 14530 | cg12623536 |
| 14531 | cg19124816 |
| 14532 | cg05195559 |
| 14533 | cg03638905 |
| 14534 | cg27076536 |
| 14535 | cg17979827 |
| 14536 | cg11286463 |
| 14537 | cg18058747 |
| 14538 | cg03265111 |
| 14539 | cg07538447 |
| 14540 | cg06138935 |
| 14541 | cg11015251 |
| 14542 | cg24182240 |
| 14543 | cg04423556 |
| 14544 | cg11128956 |
| 14545 | cg16944251 |
| 14546 | cg12324970 |
| 14547 | cg04737185 |
| 14548 | cg05526801 |
| 14549 | cg12574140 |
| 14550 | cg01430863 |
| 14551 | cg18163955 |
| 14552 | cg27005118 |
| 14553 | cg02840179 |
| 14554 | cg11774454 |
| 14555 | cg17583957 |
| 14556 | cg19325331 |
| 14557 | cg17737314 |
| 14558 | cg21643178 |
| 14559 | cg18100581 |
| 14560 | cg26470175 |
| 14561 | cg00330375 |
| 14562 | cg23533513 |
| 14563 | cg22769375 |
| 14564 | cg13259711 |
| 14565 | cg04717802 |
| 14566 | cg25974450 |
| 14567 | cg23686842 |
| 14568 | cg24682910 |
| 14569 | cg15477363 |
| 14570 | cg05718034 |
| 14571 | cg09852604 |
| 14572 | cg20824873 |
| 14573 | cg26266424 |
| 14574 | cg11644454 |
| 14575 | cg06359375 |
| 14576 | cg13644071 |
| 14577 | cg05157360 |
| 14578 | cg22896572 |
| 14579 | cg27311590 |
| 14580 | cg01617990 |
| 14581 | cg23461615 |
| 14582 | cg04245248 |
| 14583 | cg25945642 |
| 14584 | cg08944241 |
| 14585 | cg03749794 |
| 14586 | cg04155451 |
| 14587 | cg03685475 |
| 14588 | cg19321535 |
| 14589 | cg24497732 |
| 14590 | cg21755044 |
| 14591 | cg05705704 |
| 14592 | cg24935598 |
| 14593 | cg13443733 |
| 14594 | cg02415992 |
| 14595 | cg17455757 |
| 14596 | cg00928711 |
| 14597 | cg07816850 |
| 14598 | cg24521781 |
| 14599 | cg24227026 |
| 14600 | cg07872430 |
| 14601 | cg22505766 |
| 14602 | cg21115691 |
| 14603 | cg19885284 |
| 14604 | cg07263598 |
| 14605 | cg00400259 |
| 14606 | cg09558069 |
| 14607 | cg07300063 |
| 14608 | cg01254303 |
| 14609 | cg05357527 |
| 14610 | cg12507643 |
| 14611 | cg15848561 |
| 14612 | cg18567079 |
| 14613 | cg17764549 |
| 14614 | cg17533330 |
| 14615 | cg20196215 |
| 14616 | cg22609017 |
| 14617 | cg02600910 |
| 14618 | cg07998677 |
| 14619 | cg08509901 |
| 14620 | cg22726310 |
| 14621 | cg07632468 |
| 14622 | cg00350735 |
| 14623 | cg00393047 |
| 14624 | cg18492526 |
| 14625 | cg12407258 |
| 14626 | cg27064708 |
| 14627 | cg25752864 |
| 14628 | cg06978604 |
| 14629 | cg07744502 |
| 14630 | cg19379125 |
| 14631 | cg08621778 |
| 14632 | cg13758054 |
| 14633 | cg07942997 |
| 14634 | cg00021892 |
| 14635 | cg02619298 |
| 14636 | cg24806360 |
| 14637 | cg19565305 |
| 14638 | cg11445109 |
| 14639 | cg04445379 |
| 14640 | cg05363005 |
| 14641 | cg19264599 |
| 14642 | cg22705268 |
| 14643 | cg08924449 |
| 14644 | cg05242348 |
| 14645 | cg07836225 |
| 14646 | cg25880324 |
| 14647 | cg08532566 |
| 14648 | cg10885338 |
| 14649 | cg07832143 |
| 14650 | cg22589137 |
| 14651 | cg11787176 |
| 14652 | cg05199761 |
| 14653 | cg24164973 |
| 14654 | cg20802276 |
| 14655 | cg12370224 |
| 14656 | cg03426320 |
| 14657 | cg25517633 |
| 14658 | cg01181898 |
| 14659 | cg10277651 |
| 14660 | cg21262834 |
| 14661 | cg12560128 |
| 14662 | cg04807036 |
| 14663 | cg15858512 |
| 14664 | cg15603793 |
| 14665 | cg04037327 |
| 14666 | cg12521225 |
| 14667 | cg01525847 |
| 14668 | cg18906971 |
| 14669 | cg15445000 |
| 14670 | cg25316769 |
| 14671 | cg25598400 |
| 14672 | cg26661122 |
| 14673 | cg16957890 |
| 14674 | cg06607919 |
| 14675 | cg12797828 |
| 14676 | cg22169777 |
| 14677 | cg17341765 |
| 14678 | cg15900701 |
| 14679 | cg18473137 |
| 14680 | cg04792676 |
| 14681 | cg03689129 |
| 14682 | cg11279967 |
| 14683 | cg11941630 |
| 14684 | cg01719832 |
| 14685 | cg11324957 |
| 14686 | cg24485037 |
| 14687 | cg14561565 |
| 14688 | cg22709362 |
| 14689 | cg21851672 |
| 14690 | cg24625984 |
| 14691 | cg05641292 |
| 14692 | cg23612423 |
| 14693 | cg05076590 |
| 14694 | cg05994135 |
| 14695 | cg06480353 |
| 14696 | cg05018648 |
| 14697 | cg12912344 |
| 14698 | cg11131246 |
| 14699 | cg00044665 |
| 14700 | cg15272762 |
| 14701 | cg14917096 |
| 14702 | cg04738552 |
| 14703 | cg26726768 |
| 14704 | cg03573064 |
| 14705 | cg20811072 |
| 14706 | cg01684256 |
| 14707 | cg03616722 |
| 14708 | cg26639747 |
| 14709 | cg03268860 |
| 14710 | cg01939562 |
| 14711 | cg05081182 |
| 14712 | cg02679214 |
| 14713 | cg14640762 |
| 14714 | cg01263833 |
| 14715 | cg06646708 |
| 14716 | cg24877675 |
| 14717 | cg23206461 |
| 14718 | cg10605603 |
| 14719 | cg22481115 |
| 14720 | cg10761077 |
| 14721 | cg03139377 |
| 14722 | cg12423387 |
| 14723 | cg18172411 |
| 14724 | cg21498490 |
| 14725 | cg06643013 |
| 14726 | cg02411429 |
| 14727 | cg17337233 |
| 14728 | cg27063263 |
| 14729 | cg01994826 |
| 14730 | cg19291518 |
| 14731 | cg16276113 |
| 14732 | cg03315346 |
| 14733 | cg02481842 |
| 14734 | cg14538537 |
| 14735 | cg13524973 |
| 14736 | cg18213256 |
| 14737 | cg05863003 |
| 14738 | cg25818611 |
| 14739 | cg01165402 |
| 14740 | cg23841748 |
| 14741 | cg00812435 |
| 14742 | cg20238678 |
| 14743 | cg02218200 |
| 14744 | cg25767001 |
| 14745 | cg24914149 |
| 14746 | cg02808427 |
| 14747 | cg02063835 |
| 14748 | cg02571968 |
| 14749 | cg02936049 |
| 14750 | cg07594247 |
| 14751 | cg21172322 |
| 14752 | cg14952313 |
| 14753 | cg19601636 |
| 14754 | cg06569117 |
| 14755 | cg00982287 |
| 14756 | cg01566235 |
| 14757 | cg11603443 |
| 14758 | cg03676956 |
| 14759 | cg14924495 |
| 14760 | cg11060956 |
| 14761 | cg26098768 |
| 14762 | cg13622535 |
| 14763 | cg13624602 |
| 14764 | cg14955976 |
| 14765 | cg05176806 |
| 14766 | cg05393286 |
| 14767 | cg20608100 |
| 14768 | cg01281435 |
| 14769 | cg17199367 |
| 14770 | cg09856853 |
| 14771 | cg04551579 |
| 14772 | cg10874111 |
| 14773 | cg14317639 |
| 14774 | cg16033633 |
| 14775 | cg20793897 |
| 14776 | cg04345581 |
| 14777 | cg08439930 |
| 14778 | cg13806029 |
| 14779 | cg07430967 |
| 14780 | cg11132921 |
| 14781 | cg14791737 |
| 14782 | cg15697019 |
| 14783 | cg03804272 |
| 14784 | cg01424997 |
| 14785 | cg18390345 |
| 14786 | cg23532123 |
| 14787 | cg10820485 |
| 14788 | cg03460240 |
| 14789 | cg14184524 |
| 14790 | cg10545366 |
| 14791 | cg10000705 |
| 14792 | cg05980473 |
| 14793 | cg23819204 |
| 14794 | cg26888672 |
| 14795 | cg27500970 |
| 14796 | cg11807903 |
| 14797 | cg14907703 |
| 14798 | cg05908840 |
| 14799 | cg08732526 |
| 14800 | cg03785432 |
| 14801 | cg00567115 |
| 14802 | cg26296894 |
| 14803 | cg21597425 |
| 14804 | cg00665374 |
| 14805 | cg08781015 |
| 14806 | cg27568751 |
| 14807 | cg26721644 |
| 14808 | cg04635543 |
| 14809 | cg04597449 |
| 14810 | cg04661001 |
| 14811 | cg15770813 |
| 14812 | cg07363131 |
| 14813 | cg08815081 |
| 14814 | cg15312943 |
| 14815 | cg07931368 |
| 14816 | cg10351275 |
| 14817 | cg06526688 |
| 14818 | cg08205065 |
| 14819 | cg24193254 |
| 14820 | cg03864211 |
| 14821 | cg05830220 |
| 14822 | cg10696316 |
| 14823 | cg20268658 |
| 14824 | cg10179123 |
| 14825 | cg19174603 |
| 14826 | cg18489675 |
| 14827 | cg01850011 |
| 14828 | cg10331829 |
| 14829 | cg04001333 |
| 14830 | cg10334285 |
| 14831 | cg04119303 |
| 14832 | cg08211068 |
| 14833 | cg24610399 |
| 14834 | cg05204167 |
| 14835 | cg15393941 |
| 14836 | cg03792071 |
| 14837 | cg06510243 |
| 14838 | cg10122097 |
| 14839 | cg17110364 |
| 14840 | cg02786964 |
| 14841 | cg06029792 |
| 14842 | cg17939585 |
| 14843 | cg06535121 |
| 14844 | cg04742578 |
| 14845 | cg11234449 |
| 14846 | cg02888092 |
| 14847 | cg15193638 |
| 14848 | cg13553204 |
| 14849 | cg08407204 |
| 14850 | cg11289039 |
| 14851 | cg16533692 |
| 14852 | cg04426698 |
| 14853 | cg12447100 |
| 14854 | cg14541582 |
| 14855 | cg24766228 |
| 14856 | cg13037738 |
| 14857 | cg23442198 |
| 14858 | cg23821359 |
| 14859 | cg22257105 |
| 14860 | cg08094381 |
| 14861 | cg26060835 |
| 14862 | cg24634568 |
| 14863 | cg20083079 |
| 14864 | cg26460366 |
| 14865 | cg01400685 |
| 14866 | cg19804071 |
| 14867 | cg06921399 |
| 14868 | cg18484136 |
| 14869 | cg04024514 |
| 14870 | cg08856033 |
| 14871 | cg01104230 |
| 14872 | cg08094541 |
| 14873 | cg12343363 |
| 14874 | cg00469540 |
| 14875 | cg00949224 |
| 14876 | cg02739870 |
| 14877 | cg09058974 |
| 14878 | cg08218720 |
| 14879 | cg09026070 |
| 14880 | cg06260709 |
| 14881 | cg27500473 |
| 14882 | cg08004951 |
| 14883 | cg18839637 |
| 14884 | cg03058664 |
| 14885 | cg04215345 |
| 14886 | cg04086018 |
| 14887 | cg15041339 |
| 14888 | cg19297245 |
| 14889 | cg17614598 |
| 14890 | cg09015774 |
| 14891 | cg24238409 |
| 14892 | cg17523053 |
| 14893 | cg08569678 |
| 14894 | cg19319037 |
| 14895 | cg09248044 |
| 14896 | cg11642412 |
| 14897 | cg02121060 |
| 14898 | cg27299406 |
| 14899 | cg21937377 |
| 14900 | cg09881453 |
| 14901 | cg21202178 |
| 14902 | cg05990891 |
| 14903 | cg02825211 |
| 14904 | cg02656594 |
| 14905 | cg10390058 |
| 14906 | cg15651267 |
| 14907 | cg23198699 |
| 14908 | cg06120750 |
| 14909 | cg19644750 |
| 14910 | cg16480239 |
| 14911 | cg03812136 |
| 14912 | cg24365704 |
| 14913 | cg23687322 |
| 14914 | cg24913868 |
| 14915 | cg23960442 |
| 14916 | cg22348755 |
| 14917 | cg20200594 |
| 14918 | cg16985405 |
| 14919 | cg17499156 |
| 14920 | cg26666428 |
| 14921 | cg24697254 |
| 14922 | cg10178836 |
| 14923 | cg20519495 |
| 14924 | cg26024214 |
| 14925 | cg01208318 |
| 14926 | cg10477905 |
| 14927 | cg20149111 |
| 14928 | cg01192889 |
| 14929 | cg26581860 |
| 14930 | cg00375146 |
| 14931 | cg03852267 |
| 14932 | cg15583492 |
| 14933 | cg13849253 |
| 14934 | cg08066376 |
| 14935 | cg17783117 |
| 14936 | cg11752927 |
| 14937 | cg07403367 |
| 14938 | cg24144391 |
| 14939 | cg18352985 |
| 14940 | cg05891765 |
| 14941 | cg03858703 |
| 14942 | cg02391757 |
| 14943 | cg02419321 |
| 14944 | cg01927901 |
| 14945 | cg27114734 |
| 14946 | cg23388881 |
| 14947 | cg03207054 |
| 14948 | cg20147755 |
| 14949 | cg08942800 |
| 14950 | cg16580038 |
| 14951 | cg16864658 |
| 14952 | cg09611679 |
| 14953 | cg04675946 |
| 14954 | cg13641786 |
| 14955 | cg03819136 |
| 14956 | cg04059762 |
| 14957 | cg07388532 |
| 14958 | cg00840597 |
| 14959 | cg14743859 |
| 14960 | cg10588310 |
| 14961 | cg26097051 |
| 14962 | cg15897287 |
| 14963 | cg06023006 |
| 14964 | cg08626439 |
| 14965 | cg22230660 |
| 14966 | cg12622378 |
| 14967 | cg02106719 |
| 14968 | cg25637655 |
| 14969 | cg02196220 |
| 14970 | cg10308833 |
| 14971 | cg21029035 |
| 14972 | cg13391259 |
| 14973 | cg25114873 |
| 14974 | cg04954225 |
| 14975 | cg11875044 |
| 14976 | cg11743670 |
| 14977 | cg12709329 |
| 14978 | cg18091615 |
| 14979 | cg08051321 |
| 14980 | cg21951729 |
| 14981 | cg23350904 |
| 14982 | cg17927488 |
| 14983 | cg22887880 |
| 14984 | cg25533993 |
| 14985 | cg12349623 |
| 14986 | cg17128833 |
| 14987 | cg11304664 |
| 14988 | cg15800151 |
| 14989 | cg05395509 |
| 14990 | cg20353001 |
| 14991 | cg02566391 |
| 14992 | cg24410381 |
| 14993 | cg10530568 |
| 14994 | cg00913495 |
| 14995 | cg03410640 |
| 14996 | cg21335012 |
| 14997 | cg20232723 |
| 14998 | cg08501334 |
| 14999 | cg23526586 |
| 15000 | cg18552439 |
| 15001 | cg23059946 |
| 15002 | cg06365976 |
| 15003 | cg16989822 |
| 15004 | cg09727812 |
| 15005 | cg13533801 |
| 15006 | cg05952379 |
| 15007 | cg01604411 |
| 15008 | cg25306480 |
| 15009 | cg13427598 |
| 15010 | cg23299462 |
| 15011 | cg14202477 |
| 15012 | cg04121938 |
| 15013 | cg10923408 |
| 15014 | cg06343355 |
| 15015 | cg23170410 |
| 15016 | cg17532672 |
| 15017 | cg04055843 |
| 15018 | cg08664021 |
| 15019 | cg13567972 |
| 15020 | cg16795772 |
| 15021 | cg15343023 |
| 15022 | cg18464784 |
| 15023 | cg00805728 |
| 15024 | cg19835525 |
| 15025 | cg06857925 |
| 15026 | cg13926524 |
| 15027 | cg20417000 |
| 15028 | cg06179667 |
| 15029 | cg04602696 |
| 15030 | cg13995010 |
| 15031 | cg01356829 |
| 15032 | cg03981685 |
| 15033 | cg09460013 |
| 15034 | cg10646193 |
| 15035 | cg06507301 |
| 15036 | cg07922719 |
| 15037 | cg10669481 |
| 15038 | cg15051989 |
| 15039 | cg27213238 |
| 15040 | cg05578102 |
| 15041 | cg26368594 |
| 15042 | cg04343927 |
| 15043 | cg15154249 |
| 15044 | cg27547132 |
| 15045 | cg15411403 |
| 15046 | cg06186155 |
| 15047 | cg21110456 |
| 15048 | cg27459367 |
| 15049 | cg15114001 |
| 15050 | cg22785294 |
| 15051 | cg21477262 |
| 15052 | cg20645058 |
| 15053 | cg13970894 |
| 15054 | cg08219069 |
| 15055 | cg15638189 |
| 15056 | cg10526223 |
| 15057 | cg05465666 |
| 15058 | cg14528468 |
| 15059 | cg18715220 |
| 15060 | cg00350353 |
| 15061 | cg22610645 |
| 15062 | cg25350011 |
| 15063 | cg21289795 |
| 15064 | cg16758079 |
| 15065 | cg17099048 |
| 15066 | cg18529726 |
| 15067 | cg00448952 |
| 15068 | cg15464727 |
| 15069 | cg25790232 |
| 15070 | cg16467921 |
| 15071 | cg04093298 |
| 15072 | cg10090732 |
| 15073 | cg01150129 |
| 15074 | cg00002426 |
| 15075 | cg08881864 |
| 15076 | cg01981417 |
| 15077 | cg25428553 |
| 15078 | cg05378938 |
| 15079 | cg22697217 |
| 15080 | cg13981380 |
| 15081 | cg13990355 |
| 15082 | cg21068178 |
| 15083 | cg04789475 |
| 15084 | cg08702717 |
| 15085 | cg08860346 |
| 15086 | cg25330387 |
| 15087 | cg12474394 |
| 15088 | cg19350469 |
| 15089 | cg18117039 |
| 15090 | cg09129971 |
| 15091 | cg21723861 |
| 15092 | cg03432176 |
| 15093 | cg26580869 |
| 15094 | cg03659519 |
| 15095 | cg13337047 |
| 15096 | cg12877039 |
| 15097 | cg19601398 |
| 15098 | cg24407355 |
| 15099 | cg16581308 |
| 15100 | cg12008581 |
| 15101 | cg08799000 |
| 15102 | cg24561297 |
| 15103 | cg22423974 |
| 15104 | cg23210852 |
| 15105 | cg04628369 |
| 15106 | cg09793883 |
| 15107 | cg02649698 |
| 15108 | cg08230215 |
| 15109 | cg24544593 |
| 15110 | cg05864370 |
| 15111 | cg22925650 |
| 15112 | cg03462868 |
| 15113 | cg15325875 |
| 15114 | cg11743503 |
| 15115 | cg04272994 |
| 15116 | cg09056876 |
| 15117 | cg24529771 |
| 15118 | cg09936757 |
| 15119 | cg03889507 |
| 15120 | cg01231183 |
| 15121 | cg17796323 |
| 15122 | cg00860726 |
| 15123 | cg25110734 |
| 15124 | cg26688762 |
| 15125 | cg07151399 |
| 15126 | cg12647497 |
| 15127 | cg11907323 |
| 15128 | cg01928545 |
| 15129 | cg01983682 |
| 15130 | cg13983941 |
| 15131 | cg26782748 |
| 15132 | cg16208622 |
| 15133 | cg11469778 |
| 15134 | cg12897578 |
| 15135 | cg03043243 |
| 15136 | cg26152950 |
| 15137 | cg13764421 |
| 15138 | cg07325427 |
| 15139 | cg09899185 |
| 15140 | cg03680032 |
| 15141 | cg11316904 |
| 15142 | cg07930121 |
| 15143 | cg21400398 |
| 15144 | cg08873424 |
| 15145 | cg05018116 |
| 15146 | cg20564892 |
| 15147 | cg25388528 |
| 15148 | cg04821993 |
| 15149 | cg06999858 |
| 15150 | cg20005705 |
| 15151 | cg23214534 |
| 15152 | cg12365190 |
| 15153 | cg12398528 |
| 15154 | cg01298452 |
| 15155 | cg09453103 |
| 15156 | cg16969368 |
| 15157 | cg18963913 |
| 15158 | cg20797409 |
| 15159 | cg00814985 |
| 15160 | cg04452158 |
| 15161 | cg17461492 |
| 15162 | cg18433728 |
| 15163 | cg18644703 |
| 15164 | cg17244719 |
| 15165 | cg03467725 |
| 15166 | cg04943066 |
| 15167 | cg01467266 |
| 15168 | cg13151291 |
| 15169 | cg11049680 |
| 15170 | cg10934001 |
| 15171 | cg26224664 |
| 15172 | cg20009044 |
| 15173 | cg14176930 |
| 15174 | cg18178197 |
| 15175 | cg11523424 |
| 15176 | cg11460471 |
| 15177 | cg15092219 |
| 15178 | cg19079150 |
| 15179 | cg01468696 |
| 15180 | cg12853675 |
| 15181 | cg19280751 |
| 15182 | cg12323528 |
| 15183 | cg02434121 |
| 15184 | cg20607506 |
| 15185 | cg04671803 |
| 15186 | cg25185014 |
| 15187 | cg01905967 |
| 15188 | cg22489305 |
| 15189 | cg26495711 |
| 15190 | cg12445658 |
| 15191 | cg03198741 |
| 15192 | cg18151540 |
| 15193 | cg17221955 |
| 15194 | cg14753356 |
| 15195 | cg06925304 |
| 15196 | cg13121699 |
| 15197 | cg23287992 |
| 15198 | cg24042578 |
| 15199 | cg25937382 |
| 15200 | cg12357458 |
| 15201 | cg05679108 |
| 15202 | cg03476323 |
| 15203 | cg03029204 |
| 15204 | cg15209988 |
| 15205 | cg25906647 |
| 15206 | cg11204177 |
| 15207 | cg26050864 |
| 15208 | cg22076160 |
| 15209 | cg12718582 |
| 15210 | cg08834350 |
| 15211 | cg07791803 |
| 15212 | cg11959697 |
| 15213 | cg24392274 |
| 15214 | cg25941151 |
| 15215 | cg27365825 |
| 15216 | cg13947929 |
| 15217 | cg16400495 |
| 15218 | cg02462015 |
| 15219 | cg03089483 |
| 15220 | cg02168291 |
| 15221 | cg22246155 |
| 15222 | cg27661209 |
| 15223 | cg19497767 |
| 15224 | cg16818286 |
| 15225 | cg07501125 |
| 15226 | cg00105822 |
| 15227 | cg24127994 |
| 15228 | cg25113360 |
| 15229 | cg09419027 |
| 15230 | cg00669876 |
| 15231 | cg01180547 |
| 15232 | cg01848079 |
| 15233 | cg11405475 |
| 15234 | cg06459104 |
| 15235 | cg23244697 |
| 15236 | cg19241311 |
| 15237 | cg14948757 |
| 15238 | cg16676200 |
| 15239 | cg12339752 |
| 15240 | cg15174906 |
| 15241 | cg25569999 |
| 15242 | cg19618984 |
| 15243 | cg08699074 |
| 15244 | cg08644381 |
| 15245 | cg01262290 |
| 15246 | cg10458446 |
| 15247 | cg20635144 |
| 15248 | cg17693713 |
| 15249 | cg13952840 |
| 15250 | cg03704733 |
| 15251 | cg04955351 |
| 15252 | cg06430465 |
| 15253 | cg12174804 |
| 15254 | cg05487895 |
| 15255 | cg26438215 |
| 15256 | cg18756184 |
| 15257 | cg09814468 |
| 15258 | cg07091758 |
| 15259 | cg11804021 |
| 15260 | cg14448735 |
| 15261 | cg11826862 |
| 15262 | cg14554507 |
| 15263 | cg08610901 |
| 15264 | cg17633736 |
| 15265 | cg07036786 |
| 15266 | cg08874886 |
| 15267 | cg11615461 |
| 15268 | cg15055165 |
| 15269 | cg14251734 |
| 15270 | cg26151999 |
| 15271 | cg13567405 |
| 15272 | cg01445163 |
| 15273 | cg03865213 |
| 15274 | cg00443946 |
| 15275 | cg16297799 |
| 15276 | cg10393598 |
| 15277 | cg13007871 |
| 15278 | cg10258419 |
| 15279 | cg15806329 |
| 15280 | cg00087806 |
| 15281 | cg13478528 |
| 15282 | cg19464563 |
| 15283 | cg19584649 |
| 15284 | cg26862822 |
| 15285 | cg04937416 |
| 15286 | cg22546374 |
| 15287 | cg09167956 |
| 15288 | cg09774589 |
| 15289 | cg17172331 |
| 15290 | cg17119279 |
| 15291 | cg01979223 |
| 15292 | cg23248150 |
| 15293 | cg21450199 |
| 15294 | cg00417929 |
| 15295 | cg08161041 |
| 15296 | cg17122582 |
| 15297 | cg18220786 |
| 15298 | cg10160527 |
| 15299 | cg20731561 |
| 15300 | cg05268444 |
| 15301 | cg07464977 |
| 15302 | cg23311905 |
| 15303 | cg12629485 |
| 15304 | cg05951817 |
| 15305 | cg25390787 |
| 15306 | cg05625284 |
| 15307 | cg00432177 |
| 15308 | cg21085915 |
| 15309 | cg08931835 |
| 15310 | cg18081258 |
| 15311 | cg18734246 |
| 15312 | cg05306310 |
| 15313 | cg07151596 |
| 15314 | cg12441203 |
| 15315 | cg06702891 |
| 15316 | cg19905109 |
| 15317 | cg23762445 |
| 15318 | cg18830687 |
| 15319 | cg22257514 |
| 15320 | cg14807446 |
| 15321 | cg19650983 |
| 15322 | cg26773607 |
| 15323 | cg10682119 |
| 15324 | cg02855850 |
| 15325 | cg05998610 |
| 15326 | cg07123855 |
| 15327 | cg20066782 |
| 15328 | cg15731317 |
| 15329 | cg07045119 |
| 15330 | cg16592274 |
| 15331 | cg04190037 |
| 15332 | cg10987071 |
| 15333 | cg22066168 |
| 15334 | cg20966426 |
| 15335 | cg12061236 |
| 15336 | cg12945444 |
| 15337 | cg20512545 |
| 15338 | cg08549396 |
| 15339 | cg20061010 |
| 15340 | cg08081727 |
| 15341 | cg06615355 |
| 15342 | cg08637946 |
| 15343 | cg14220348 |
| 15344 | cg21770061 |
| 15345 | cg20421058 |
| 15346 | cg24194279 |
| 15347 | cg02057050 |
| 15348 | cg20606000 |
| 15349 | cg26953727 |
| 15350 | cg02216463 |
| 15351 | cg16130408 |
| 15352 | cg15125338 |
| 15353 | cg14317384 |
| 15354 | cg20535653 |
| 15355 | cg08640361 |
| 15356 | cg27212386 |
| 15357 | cg01259220 |
| 15358 | cg09731841 |
| 15359 | cg07257824 |
| 15360 | cg04837697 |
| 15361 | cg15835339 |
| 15362 | cg18560264 |
| 15363 | cg16117799 |
| 15364 | cg08855908 |
| 15365 | cg17672639 |
| 15366 | cg17738010 |
| 15367 | cg00295485 |
| 15368 | cg11697978 |
| 15369 | cg07620230 |
| 15370 | cg04434244 |
| 15371 | cg08758963 |
| 15372 | cg16362592 |
| 15373 | cg16509777 |
| 15374 | cg01107185 |
| 15375 | cg18589526 |
| 15376 | cg11675881 |
| 15377 | cg14637685 |
| 15378 | cg07578108 |
| 15379 | cg10515131 |
| 15380 | cg22550229 |
| 15381 | cg05573550 |
| 15382 | cg16021909 |
| 15383 | cg03491584 |
| 15384 | cg16086730 |
| 15385 | cg25564241 |
| 15386 | cg07659075 |
| 15387 | cg23763735 |
| 15388 | cg23208120 |
| 15389 | cg22277593 |
| 15390 | cg21616755 |
| 15391 | cg07072704 |
| 15392 | cg19444609 |
| 15393 | cg12439163 |
| 15394 | cg13089905 |
| 15395 | cg27127608 |
| 15396 | cg21939759 |
| 15397 | cg00568177 |
| 15398 | cg02546607 |
| 15399 | cg14674796 |
| 15400 | cg10377354 |
| 15401 | cg24920358 |
| 15402 | cg27494164 |
| 15403 | cg13871695 |
| 15404 | cg21681017 |
| 15405 | cg04995717 |
| 15406 | cg26978396 |
| 15407 | cg00643864 |
| 15408 | cg01094891 |
| 15409 | cg10591797 |
| 15410 | cg14202850 |
| 15411 | cg05046589 |
| 15412 | cg10453443 |
| 15413 | cg02005231 |
| 15414 | cg07055397 |
| 15415 | cg14508358 |
| 15416 | cg26815456 |
| 15417 | cg05750047 |
| 15418 | cg13470831 |
| 15419 | cg14313213 |
| 15420 | cg07782603 |
| 15421 | cg16811851 |
| 15422 | cg06395232 |
| 15423 | cg09726239 |
| 15424 | cg21868599 |
| 15425 | cg24093698 |
| 15426 | cg11834233 |
| 15427 | cg20020844 |
| 15428 | cg16155175 |
| 15429 | cg05259303 |
| 15430 | cg15257820 |
| 15431 | cg14698070 |
| 15432 | cg23556939 |
| 15433 | cg06503201 |
| 15434 | cg05546642 |
| 15435 | cg09961381 |
| 15436 | cg00827210 |
| 15437 | cg15747595 |
| 15438 | cg09044574 |
| 15439 | cg08619102 |
| 15440 | cg12476758 |
| 15441 | cg18724891 |
| 15442 | cg01323104 |
| 15443 | cg04145621 |
| 15444 | cg01509725 |
| 15445 | cg01708932 |
| 15446 | cg03675845 |
| 15447 | cg06313447 |
| 15448 | cg09731946 |
| 15449 | cg25471917 |
| 15450 | cg11183307 |
| 15451 | cg25058019 |
| 15452 | cg14489013 |
| 15453 | cg18749404 |
| 15454 | cg10746396 |
| 15455 | cg04315688 |
| 15456 | cg20263853 |
| 15457 | cg25631863 |
| 15458 | cg26088163 |
| 15459 | cg26904005 |
| 15460 | cg11641102 |
| 15461 | cg07849944 |
| 15462 | cg02370562 |
| 15463 | cg08335273 |
| 15464 | cg22660904 |
| 15465 | cg05412410 |
| 15466 | cg06596503 |
| 15467 | cg13622530 |
| 15468 | cg06459049 |
| 15469 | cg18991998 |
| 15470 | cg13244241 |
| 15471 | cg17283327 |
| 15472 | cg03740167 |
| 15473 | cg10634257 |
| 15474 | cg27624684 |
| 15475 | cg02633817 |
| 15476 | cg06724245 |
| 15477 | cg24049888 |
| 15478 | cg27183030 |
| 15479 | cg19912737 |
| 15480 | cg19149020 |
| 15481 | cg11260259 |
| 15482 | cg10473623 |
| 15483 | cg06621452 |
| 15484 | cg26924967 |
| 15485 | cg19448426 |
| 15486 | cg11274314 |
| 15487 | cg19412467 |
| 15488 | cg15846434 |
| 15489 | cg12147346 |
| 15490 | cg17260475 |
| 15491 | cg00737916 |
| 15492 | cg03955589 |
| 15493 | cg06744540 |
| 15494 | cg15654025 |
| 15495 | cg07073529 |
| 15496 | cg23463608 |
| 15497 | cg10498365 |
| 15498 | cg03805182 |
| 15499 | cg23713299 |
| 15500 | cg13201942 |
| 15501 | cg22109370 |
| 15502 | cg17378342 |
| 15503 | cg23357257 |
| 15504 | cg02054776 |
| 15505 | cg02849511 |
| 15506 | cg14454227 |
| 15507 | cg05751542 |
| 15508 | cg09559672 |
| 15509 | cg20068535 |
| 15510 | cg20739013 |
| 15511 | cg17172187 |
| 15512 | cg14376791 |
| 15513 | cg10248878 |
| 15514 | cg07721777 |
| 15515 | cg05137386 |
| 15516 | cg01800986 |
| 15517 | cg10963793 |
| 15518 | cg06934299 |
| 15519 | cg22497094 |
| 15520 | cg00790020 |
| 15521 | cg11091888 |
| 15522 | cg12914966 |
| 15523 | cg14442689 |
| 15524 | cg02280031 |
| 15525 | cg22611274 |
| 15526 | cg15292513 |
| 15527 | cg02424654 |
| 15528 | cg16059405 |
| 15529 | cg12894334 |
| 15530 | cg03444722 |
| 15531 | cg07746943 |
| 15532 | cg14223654 |
| 15533 | cg13485366 |
| 15534 | cg05454848 |
| 15535 | cg24699296 |
| 15536 | cg10779492 |
| 15537 | cg17261676 |
| 15538 | cg03961824 |
| 15539 | cg07189587 |
| 15540 | cg14385834 |
| 15541 | cg13638593 |
| 15542 | cg16170614 |
| 15543 | cg02940362 |
| 15544 | cg18527574 |
| 15545 | cg01264765 |
| 15546 | cg05859572 |
| 15547 | cg16331194 |
| 15548 | cg09611279 |
| 15549 | cg11072645 |
| 15550 | cg25795270 |
| 15551 | cg14009508 |
| 15552 | cg06580966 |
| 15553 | cg06162872 |
| 15554 | cg16261483 |
| 15555 | cg07013148 |
| 15556 | cg04920044 |
| 15557 | cg06635918 |
| 15558 | cg00333679 |
| 15559 | cg07109649 |
| 15560 | cg25174343 |
| 15561 | cg02367723 |
| 15562 | cg25600383 |
| 15563 | cg09424828 |
| 15564 | cg03650063 |
| 15565 | cg24347336 |
| 15566 | cg15476602 |
| 15567 | cg26132493 |
| 15568 | cg03296935 |
| 15569 | cg15797286 |
| 15570 | cg17743056 |
| 15571 | cg11290182 |
| 15572 | cg19046580 |
| 15573 | cg24847993 |
| 15574 | cg27020649 |
| 15575 | cg27097542 |
| 15576 | cg21630608 |
| 15577 | cg00832644 |
| 15578 | cg00388596 |
| 15579 | cg06420834 |
| 15580 | cg09623982 |
| 15581 | cg06373584 |
| 15582 | cg16849440 |
| 15583 | cg19314159 |
| 15584 | cg09868294 |
| 15585 | cg06754764 |
| 15586 | cg16837441 |
| 15587 | cg12848070 |
| 15588 | cg10927178 |
| 15589 | cg06028808 |
| 15590 | cg23310290 |
| 15591 | cg06830360 |
| 15592 | cg11300208 |
| 15593 | cg01126532 |
| 15594 | cg02728634 |
| 15595 | cg14890311 |
| 15596 | cg06824288 |
| 15597 | cg26158194 |
| 15598 | cg12589188 |
| 15599 | cg15182287 |
| 15600 | cg25463863 |
| 15601 | cg16021018 |
| 15602 | cg02616906 |
| 15603 | cg26819695 |
| 15604 | cg05107650 |
| 15605 | cg22507435 |
| 15606 | cg15989926 |
| 15607 | cg01966791 |
| 15608 | cg23034985 |
| 15609 | cg02506716 |
| 15610 | cg06154597 |
| 15611 | cg20071972 |
| 15612 | cg25861699 |
| 15613 | cg22634380 |
| 15614 | cg25532560 |
| 15615 | cg23979832 |
| 15616 | cg19474196 |
| 15617 | cg08534055 |
| 15618 | cg14100191 |
| 15619 | cg01471886 |
| 15620 | cg22268164 |
| 15621 | cg21406402 |
| 15622 | cg09357926 |
| 15623 | cg09438962 |
| 15624 | cg02245566 |
| 15625 | cg18270174 |
| 15626 | cg26531879 |
| 15627 | cg20837800 |
| 15628 | cg14395218 |
| 15629 | cg09749435 |
| 15630 | cg02081019 |
| 15631 | cg11081049 |
| 15632 | cg04353868 |
| 15633 | cg20636352 |
| 15634 | cg11756026 |
| 15635 | cg04027736 |
| 15636 | cg12424391 |
| 15637 | cg05001334 |
| 15638 | cg00230346 |
| 15639 | cg02150090 |
| 15640 | cg05513374 |
| 15641 | cg20615879 |
| 15642 | cg02041805 |
| 15643 | cg04232360 |
| 15644 | cg25801113 |
| 15645 | cg05112555 |
| 15646 | cg10466421 |
| 15647 | cg12145775 |
| 15648 | cg06504116 |
| 15649 | cg08070485 |
| 15650 | cg00551143 |
| 15651 | cg21933165 |
| 15652 | cg24258395 |
| 15653 | cg05735180 |
| 15654 | cg19203173 |
| 15655 | cg07434433 |
| 15656 | cg21691293 |
| 15657 | cg21615663 |
| 15658 | cg08464402 |
| 15659 | cg10580091 |
| 15660 | cg08898775 |
| 15661 | cg08036259 |
| 15662 | cg04387445 |
| 15663 | cg12284854 |
| 15664 | cg01841797 |
| 15665 | cg14969744 |
| 15666 | cg07394242 |
| 15667 | cg06697540 |
| 15668 | cg18314300 |
| 15669 | cg12226028 |
| 15670 | cg19623226 |
| 15671 | cg06744279 |
| 15672 | cg27537199 |
| 15673 | cg14420466 |
| 15674 | cg03251655 |
| 15675 | cg12709009 |
| 15676 | cg01021271 |
| 15677 | cg27055296 |
| 15678 | cg00401972 |
| 15679 | cg09641202 |
| 15680 | cg25968569 |
| 15681 | cg25651608 |
| 15682 | cg18801567 |
| 15683 | cg06082612 |
| 15684 | cg17602428 |
| 15685 | cg05751661 |
| 15686 | cg09676045 |
| 15687 | cg12615761 |
| 15688 | cg19418618 |
| 15689 | cg13374658 |
| 15690 | cg21752624 |
| 15691 | cg09147843 |
| 15692 | cg09927651 |
| 15693 | cg23520270 |
| 15694 | cg05648752 |
| 15695 | cg08994982 |
| 15696 | cg20358011 |
| 15697 | cg05297088 |
| 15698 | cg25544461 |
| 15699 | cg18487535 |
| 15700 | cg18597176 |
| 15701 | cg02949244 |
| 15702 | cg21770322 |
| 15703 | cg16029875 |
| 15704 | cg13012494 |
| 15705 | cg02065165 |
| 15706 | cg26414148 |
| 15707 | cg00178864 |
| 15708 | cg05467819 |
| 15709 | cg15946590 |
| 15710 | cg13968605 |
| 15711 | cg00795830 |
| 15712 | cg12873498 |
| 15713 | cg10231182 |
| 15714 | cg05543030 |
| 15715 | cg11967492 |
| 15716 | cg19209043 |
| 15717 | cg17902219 |
| 15718 | cg17076780 |
| 15719 | cg16869108 |
| 15720 | cg24775496 |
| 15721 | cg12418025 |
| 15722 | cg16811988 |
| 15723 | cg12493255 |
| 15724 | cg22442557 |
| 15725 | cg03814644 |
| 15726 | cg03085949 |
| 15727 | cg05920182 |
| 15728 | cg06842409 |
| 15729 | cg20587945 |
| 15730 | cg15295766 |
| 15731 | cg19985495 |
| 15732 | cg22143184 |
| 15733 | cg00539976 |
| 15734 | cg23841297 |
| 15735 | cg19537435 |
| 15736 | cg10780897 |
| 15737 | cg21683105 |
| 15738 | cg26416745 |
| 15739 | cg03662122 |
| 15740 | cg12448376 |
| 15741 | cg08442706 |
| 15742 | cg08471739 |
| 15743 | cg11862551 |
| 15744 | cg15518684 |
| 15745 | cg18049969 |
| 15746 | cg25555781 |
| 15747 | cg08096750 |
| 15748 | cg13555464 |
| 15749 | cg00624589 |
| 15750 | cg07243452 |
| 15751 | cg22473770 |
| 15752 | cg06550614 |
| 15753 | cg20560873 |
| 15754 | cg24086268 |
| 15755 | cg02556634 |
| 15756 | cg10754187 |
| 15757 | cg26568750 |
| 15758 | cg00761985 |
| 15759 | cg18334631 |
| 15760 | cg10332413 |
| 15761 | cg18327157 |
| 15762 | cg22540135 |
| 15763 | cg07978456 |
| 15764 | cg04466888 |
| 15765 | cg14145842 |
| 15766 | cg24155429 |
| 15767 | cg05584950 |
| 15768 | cg25865229 |
| 15769 | cg24356501 |
| 15770 | cg16493240 |
| 15771 | cg12450149 |
| 15772 | cg02525722 |
| 15773 | cg21663666 |
| 15774 | cg08042220 |
| 15775 | cg08282375 |
| 15776 | cg24718756 |
| 15777 | cg26014538 |
| 15778 | cg00026474 |
| 15779 | cg06812747 |
| 15780 | cg18336215 |
| 15781 | cg11667453 |
| 15782 | cg17842821 |
| 15783 | cg03644701 |
| 15784 | cg11222623 |
| 15785 | cg09041851 |
| 15786 | cg06297677 |
| 15787 | cg14506667 |
| 15788 | cg13229520 |
| 15789 | cg17025555 |
| 15790 | cg10747118 |
| 15791 | cg16789104 |
| 15792 | cg06432342 |
| 15793 | cg23005173 |
| 15794 | cg14096482 |
| 15795 | cg00080125 |
| 15796 | cg20604541 |
| 15797 | cg13445376 |
| 15798 | cg04429994 |
| 15799 | cg04039397 |
| 15800 | cg03702255 |
| 15801 | cg04574459 |
| 15802 | cg05460571 |
| 15803 | cg06547379 |
| 15804 | cg06829584 |
| 15805 | cg15512344 |
| 15806 | cg13278637 |
| 15807 | cg21236631 |
| 15808 | cg13939602 |
| 15809 | cg02742809 |
| 15810 | cg22901017 |
| 15811 | cg17870925 |
| 15812 | cg05037640 |
| 15813 | cg13557231 |
| 15814 | cg20567249 |
| 15815 | cg21775675 |
| 15816 | cg24138433 |
| 15817 | cg02479842 |
| 15818 | cg22128724 |
| 15819 | cg14525492 |
| 15820 | cg00372928 |
| 15821 | cg02631113 |
| 15822 | cg20370576 |
| 15823 | cg00922157 |
| 15824 | cg23453589 |
| 15825 | cg23934484 |
| 15826 | cg10168494 |
| 15827 | cg24354818 |
| 15828 | cg14295102 |
| 15829 | cg05888284 |
| 15830 | cg11560106 |
| 15831 | cg23896586 |
| 15832 | cg01037764 |
| 15833 | cg21735522 |
| 15834 | cg14150115 |
| 15835 | cg07787634 |
| 15836 | cg02259324 |
| 15837 | cg17487719 |
| 15838 | cg00197993 |
| 15839 | cg05414815 |
| 15840 | cg25563198 |
| 15841 | cg20026299 |
| 15842 | cg23092885 |
| 15843 | cg07259245 |
| 15844 | cg03671802 |
| 15845 | cg10097651 |
| 15846 | cg17290170 |
| 15847 | cg16686273 |
| 15848 | cg12615535 |
| 15849 | cg16813697 |
| 15850 | cg02719872 |
| 15851 | cg16983110 |
| 15852 | cg12849688 |
| 15853 | cg08580014 |
| 15854 | cg25971473 |
| 15855 | cg25582189 |
| 15856 | cg19287103 |
| 15857 | cg19080043 |
| 15858 | cg21848482 |
| 15859 | cg12624096 |
| 15860 | cg12947261 |
| 15861 | cg24613691 |
| 15862 | cg06985993 |
| 15863 | cg27147000 |
| 15864 | cg01916237 |
| 15865 | cg02883407 |
| 15866 | cg00491255 |
| 15867 | cg22689633 |
| 15868 | cg14611844 |
| 15869 | cg11931463 |
| 15870 | cg10195169 |
| 15871 | cg04027576 |
| 15872 | cg22026150 |
| 15873 | cg14119936 |
| 15874 | cg00972313 |
| 15875 | cg25315816 |
| 15876 | cg05080322 |
| 15877 | cg02753272 |
| 15878 | cg27060355 |
| 15879 | cg05486260 |
| 15880 | cg09938275 |
| 15881 | cg23347094 |
| 15882 | cg26425132 |
| 15883 | cg06112171 |
| 15884 | cg13785068 |
| 15885 | cg26834680 |
| 15886 | cg03135081 |
| 15887 | cg14738921 |
| 15888 | cg09324608 |
| 15889 | cg24180910 |
| 15890 | cg19467332 |
| 15891 | cg09414535 |
| 15892 | cg22416963 |
| 15893 | cg26241626 |
| 15894 | cg23058319 |
| 15895 | cg05473599 |
| 15896 | cg25988214 |
| 15897 | cg08884571 |
| 15898 | cg05793195 |
| 15899 | cg07733918 |
| 15900 | cg24691891 |
| 15901 | cg05977462 |
| 15902 | cg02753012 |
| 15903 | cg27598340 |
| 15904 | cg05849109 |
| 15905 | cg22697034 |
| 15906 | cg02671204 |
| 15907 | cg23725924 |
| 15908 | cg06895831 |
| 15909 | cg09681449 |
| 15910 | cg10815770 |
| 15911 | cg10030684 |
| 15912 | cg05172784 |
| 15913 | cg25012961 |
| 15914 | cg12138377 |
| 15915 | cg19844799 |
| 15916 | cg23385492 |
| 15917 | cg11848323 |
| 15918 | cg18307783 |
| 15919 | cg14849022 |
| 15920 | cg26219718 |
| 15921 | cg11047257 |
| 15922 | cg07427438 |
| 15923 | cg14126408 |
| 15924 | cg09984479 |
| 15925 | cg09533168 |
| 15926 | cg21671806 |
| 15927 | cg11388619 |
| 15928 | cg15717081 |
| 15929 | cg10893986 |
| 15930 | cg19590598 |
| 15931 | cg17377182 |
| 15932 | cg27007439 |
| 15933 | cg06448284 |
| 15934 | cg12485341 |
| 15935 | cg04212021 |
| 15936 | cg06216853 |
| 15937 | cg10737611 |
| 15938 | cg06978697 |
| 15939 | cg02164615 |
| 15940 | cg09480735 |
| 15941 | cg27003765 |
| 15942 | cg07059846 |
| 15943 | cg16539251 |
| 15944 | cg16721021 |
| 15945 | cg11439633 |
| 15946 | cg08250582 |
| 15947 | cg13094252 |
| 15948 | cg19591003 |
| 15949 | cg26615440 |
| 15950 | cg08203845 |
| 15951 | cg27262821 |
| 15952 | cg03381704 |
| 15953 | cg13818762 |
| 15954 | cg09099830 |
| 15955 | cg24574382 |
| 15956 | cg11196848 |
| 15957 | cg04304705 |
| 15958 | cg17828988 |
| 15959 | cg16151451 |
| 15960 | cg24515883 |
| 15961 | cg04131890 |
| 15962 | cg03546284 |
| 15963 | cg22160612 |
| 15964 | cg21507303 |
| 15965 | cg03486281 |
| 15966 | cg05830480 |
| 15967 | cg19987129 |
| 15968 | cg15156941 |
| 15969 | cg04508804 |
| 15970 | cg11345505 |
| 15971 | cg26362263 |
| 15972 | cg22260973 |
| 15973 | cg08732300 |
| 15974 | cg26670786 |
| 15975 | cg06647007 |
| 15976 | cg13267324 |
| 15977 | cg07012128 |
| 15978 | cg13329242 |
| 15979 | cg00147849 |
| 15980 | cg08289877 |
| 15981 | cg19180304 |
| 15982 | cg21731382 |
| 15983 | cg25903783 |
| 15984 | cg10513852 |
| 15985 | cg26494437 |
| 15986 | cg02846517 |
| 15987 | cg13775220 |
| 15988 | cg11488440 |
| 15989 | cg00897796 |
| 15990 | cg09599986 |
| 15991 | cg20269954 |
| 15992 | cg06179788 |
| 15993 | cg23416621 |
| 15994 | cg12625567 |
| 15995 | cg11896100 |
| 15996 | cg08488177 |
| 15997 | cg12976582 |
| 15998 | cg12039422 |
| 15999 | cg03218323 |
| 16000 | cg07737560 |
| 16001 | cg20216309 |
| 16002 | cg12453766 |
| 16003 | cg05314907 |
| 16004 | cg14289542 |
| 16005 | cg10534938 |
| 16006 | cg21199957 |
| 16007 | cg19280785 |
| 16008 | cg10968815 |
| 16009 | cg14987769 |
| 16010 | cg21859216 |
| 16011 | cg03263706 |
| 16012 | cg26317209 |
| 16013 | cg25497530 |
| 16014 | cg21122225 |
| 16015 | cg06818710 |
| 16016 | cg16861209 |
| 16017 | cg09704166 |
| 16018 | cg18792146 |
| 16019 | cg00486340 |
| 16020 | cg25021325 |
| 16021 | cg09644164 |
| 16022 | cg25436986 |
| 16023 | cg16159940 |
| 16024 | cg25707745 |
| 16025 | cg20872981 |
| 16026 | cg19229557 |
| 16027 | cg26629942 |
| 16028 | cg17578341 |
| 16029 | cg19417324 |
| 16030 | cg15227848 |
| 16031 | cg15586258 |
| 16032 | cg14299455 |
| 16033 | cg07158237 |
| 16034 | cg08960498 |
| 16035 | cg24079152 |
| 16036 | cg13086983 |
| 16037 | cg16368147 |
| 16038 | cg06148480 |
| 16039 | cg04212812 |
| 16040 | cg08181572 |
| 16041 | cg05306109 |
| 16042 | cg11395946 |
| 16043 | cg24578857 |
| 16044 | cg19420671 |
| 16045 | cg14595965 |
| 16046 | cg15859496 |
| 16047 | cg08202165 |
| 16048 | cg17840925 |
| 16049 | cg11509184 |
| 16050 | cg04118119 |
| 16051 | cg09713047 |
| 16052 | cg04781532 |
| 16053 | cg26744460 |
| 16054 | cg01811856 |
| 16055 | cg23715029 |
| 16056 | cg15507901 |
| 16057 | cg18932116 |
| 16058 | cg15075241 |
| 16059 | cg03924164 |
| 16060 | cg23533419 |
| 16061 | cg11389960 |
| 16062 | cg15335104 |
| 16063 | cg10739556 |
| 16064 | cg18530141 |
| 16065 | cg18038269 |
| 16066 | cg00857933 |
| 16067 | cg06904356 |
| 16068 | cg17797106 |
| 16069 | cg07009285 |
| 16070 | cg25629409 |
| 16071 | cg06454619 |
| 16072 | cg25586488 |
| 16073 | cg14203218 |
| 16074 | cg11827190 |
| 16075 | cg24323253 |
| 16076 | cg10004653 |
| 16077 | cg02017812 |
| 16078 | cg02656323 |
| 16079 | cg08041350 |
| 16080 | cg08070419 |
| 16081 | cg15329410 |
| 16082 | cg03971616 |
| 16083 | cg08257579 |
| 16084 | cg01917340 |
| 16085 | cg16704241 |
| 16086 | cg05901977 |
| 16087 | cg06569725 |
| 16088 | cg16733203 |
| 16089 | cg23930856 |
| 16090 | cg02248780 |
| 16091 | cg07803924 |
| 16092 | cg06529371 |
| 16093 | cg26060179 |
| 16094 | cg06916161 |
| 16095 | cg18396357 |
| 16096 | cg20585841 |
| 16097 | cg04886338 |
| 16098 | cg02664575 |
| 16099 | cg16386697 |
| 16100 | cg22172600 |
| 16101 | cg15034333 |
| 16102 | cg04072153 |
| 16103 | cg06060795 |
| 16104 | cg14837607 |
| 16105 | cg14397171 |
| 16106 | cg02691058 |
| 16107 | cg08471800 |
| 16108 | cg19987665 |
| 16109 | cg13032555 |
| 16110 | cg00575186 |
| 16111 | cg26288692 |
| 16112 | cg01558645 |
| 16113 | cg07824155 |
| 16114 | cg27647623 |
| 16115 | cg24541997 |
| 16116 | cg00546153 |
| 16117 | cg14400877 |
| 16118 | cg20964588 |
| 16119 | cg12581967 |
| 16120 | cg22659772 |
| 16121 | cg04322026 |
| 16122 | cg08596773 |
| 16123 | cg08586809 |
| 16124 | cg08141300 |
| 16125 | cg13363416 |
| 16126 | cg01784477 |
| 16127 | cg11828163 |
| 16128 | cg19946742 |
| 16129 | cg24754199 |
| 16130 | cg17063595 |
| 16131 | cg22101188 |
| 16132 | cg06136628 |
| 16133 | cg01965047 |
| 16134 | cg08088989 |
| 16135 | cg12293949 |
| 16136 | cg27158679 |
| 16137 | cg06163994 |
| 16138 | cg02125166 |
| 16139 | cg01635555 |
| 16140 | cg15028548 |
| 16141 | cg20561509 |
| 16142 | cg16751949 |
| 16143 | cg11911620 |
| 16144 | cg08528157 |
| 16145 | cg00874605 |
| 16146 | cg27549878 |
| 16147 | cg05468953 |
| 16148 | cg01910486 |
| 16149 | cg26562921 |
| 16150 | cg02555166 |
| 16151 | cg09306294 |
| 16152 | cg08139247 |
| 16153 | cg19028499 |
| 16154 | cg12955011 |
| 16155 | cg05033220 |
| 16156 | cg16788982 |
| 16157 | cg07606384 |
| 16158 | cg12865615 |
| 16159 | cg11063110 |
| 16160 | cg19278199 |
| 16161 | cg13805884 |
| 16162 | cg01835489 |
| 16163 | cg08594650 |
| 16164 | cg17732038 |
| 16165 | cg19454119 |
| 16166 | cg23748340 |
| 16167 | cg13829089 |
| 16168 | cg12049263 |
| 16169 | cg11725807 |
| 16170 | cg20763401 |
| 16171 | cg22018084 |
| 16172 | cg17667084 |
| 16173 | cg18637476 |
| 16174 | cg06950952 |
| 16175 | cg20800117 |
| 16176 | cg27509642 |
| 16177 | cg13581155 |
| 16178 | cg25050565 |
| 16179 | cg20959920 |
| 16180 | cg05364762 |
| 16181 | cg05461938 |
| 16182 | cg07097417 |
| 16183 | cg17891827 |
| 16184 | cg14813069 |
| 16185 | cg26553501 |
| 16186 | cg15971559 |
| 16187 | cg26239041 |
| 16188 | cg18731055 |
| 16189 | cg17667870 |
| 16190 | cg04027548 |
| 16191 | cg23090897 |
| 16192 | cg16274221 |
| 16193 | cg14992910 |
| 16194 | cg27246321 |
| 16195 | cg07336099 |
| 16196 | cg08872493 |
| 16197 | cg15159503 |
| 16198 | cg13580008 |
| 16199 | cg02763813 |
| 16200 | cg10237978 |
| 16201 | cg24277774 |
| 16202 | cg00066239 |
| 16203 | cg13123331 |
| 16204 | cg04331032 |
| 16205 | cg05070042 |
| 16206 | cg01219553 |
| 16207 | cg18505241 |
| 16208 | cg09563102 |
| 16209 | cg09475293 |
| 16210 | cg02538248 |
| 16211 | cg09163646 |
| 16212 | cg04567669 |
| 16213 | cg13429093 |
| 16214 | cg04953735 |
| 16215 | cg10403239 |
| 16216 | cg21952799 |
| 16217 | cg27160395 |
| 16218 | cg26014699 |
| 16219 | cg07027513 |
| 16220 | cg13636371 |
| 16221 | cg20389954 |
| 16222 | cg25111337 |
| 16223 | cg18603473 |
| 16224 | cg06368617 |
| 16225 | cg11367593 |
| 16226 | cg04462774 |
| 16227 | cg03653026 |
| 16228 | cg27345516 |
| 16229 | cg17176676 |
| 16230 | cg14089067 |
| 16231 | cg17865254 |
| 16232 | cg08122234 |
| 16233 | cg03326552 |
| 16234 | cg14013080 |
| 16235 | cg00901320 |
| 16236 | cg02037013 |
| 16237 | cg20543683 |
| 16238 | cg16793483 |
| 16239 | cg03224259 |
| 16240 | cg25403488 |
| 16241 | cg06985721 |
| 16242 | cg09107344 |
| 16243 | cg15366841 |
| 16244 | cg02587673 |
| 16245 | cg04815410 |
| 16246 | cg26569449 |
| 16247 | cg11086547 |
| 16248 | cg25983849 |
| 16249 | cg02849401 |
| 16250 | cg08643994 |
| 16251 | cg17530152 |
| 16252 | cg25392060 |
| 16253 | cg20945983 |
| 16254 | cg02134798 |
| 16255 | cg15786900 |
| 16256 | cg08782899 |
| 16257 | cg19013223 |
| 16258 | cg02395590 |
| 16259 | cg18444952 |
| 16260 | cg15489935 |
| 16261 | cg07341220 |
| 16262 | cg20424702 |
| 16263 | cg00068391 |
| 16264 | cg22932685 |
| 16265 | cg05394857 |
| 16266 | cg15160664 |
| 16267 | cg10421718 |
| 16268 | cg07416364 |
| 16269 | cg01626681 |
| 16270 | cg17501107 |
| 16271 | cg22933549 |
| 16272 | cg13004635 |
| 16273 | cg10721538 |
| 16274 | cg11433085 |
| 16275 | cg02479497 |
| 16276 | cg10704177 |
| 16277 | cg05213070 |
| 16278 | cg15990629 |
| 16279 | cg17374981 |
| 16280 | cg09266771 |
| 16281 | cg23811789 |
| 16282 | cg03613050 |
| 16283 | cg24113968 |
| 16284 | cg23946903 |
| 16285 | cg12663302 |
| 16286 | cg09578568 |
| 16287 | cg06275182 |
| 16288 | cg00574819 |
| 16289 | cg21864732 |
| 16290 | cg08046818 |
| 16291 | cg16917269 |
| 16292 | cg13808058 |
| 16293 | cg22478544 |
| 16294 | cg03895047 |
| 16295 | cg06933525 |
| 16296 | cg12391665 |
| 16297 | cg25878055 |
| 16298 | cg18815398 |
| 16299 | cg04028950 |
| 16300 | cg06394247 |
| 16301 | cg04836354 |
| 16302 | cg18867200 |
| 16303 | cg20102877 |
| 16304 | cg11649168 |
| 16305 | cg03861143 |
| 16306 | cg01495416 |
| 16307 | cg08510499 |
| 16308 | cg12138678 |
| 16309 | cg14652773 |
| 16310 | cg13945265 |
| 16311 | cg15615645 |
| 16312 | cg03709601 |
| 16313 | cg08840441 |
| 16314 | cg00067702 |
| 16315 | cg23236365 |
| 16316 | cg04232458 |
| 16317 | cg07135401 |
| 16318 | cg12714140 |
| 16319 | cg04288961 |
| 16320 | cg21967713 |
| 16321 | cg13676220 |
| 16322 | cg16247912 |
| 16323 | cg25724842 |
| 16324 | cg07238058 |
| 16325 | cg04002944 |
| 16326 | cg26938749 |
| 16327 | cg09468177 |
| 16328 | cg23640289 |
| 16329 | cg19066520 |
| 16330 | cg22412989 |
| 16331 | cg27373604 |
| 16332 | cg06462449 |
| 16333 | cg07010687 |
| 16334 | cg13671582 |
| 16335 | cg02239754 |
| 16336 | cg13808012 |
| 16337 | cg19593767 |
| 16338 | cg27178717 |
| 16339 | cg18599533 |
| 16340 | cg19871722 |
| 16341 | cg18450270 |
| 16342 | cg02412169 |
| 16343 | cg07125278 |
| 16344 | cg23125776 |
| 16345 | cg08335200 |
| 16346 | cg23182559 |
| 16347 | cg09840141 |
| 16348 | cg09893305 |
| 16349 | cg06012299 |
| 16350 | cg06115614 |
| 16351 | cg17140469 |
| 16352 | cg18769590 |
| 16353 | cg25862444 |
| 16354 | cg09424405 |
| 16355 | cg06722223 |
| 16356 | cg15650664 |
| 16357 | cg08321812 |
| 16358 | cg10432586 |
| 16359 | cg24993713 |
| 16360 | cg23548151 |
| 16361 | cg08893087 |
| 16362 | cg26549675 |
| 16363 | cg24608381 |
| 16364 | cg11491998 |
| 16365 | cg24693153 |
| 16366 | cg01101873 |
| 16367 | cg26496843 |
| 16368 | cg25570190 |
| 16369 | cg22046201 |
| 16370 | cg20028869 |
| 16371 | cg11137113 |
| 16372 | cg03562360 |
| 16373 | cg00021855 |
| 16374 | cg15705865 |
| 16375 | cg11637759 |
| 16376 | cg12494143 |
| 16377 | cg06741367 |
| 16378 | cg26236972 |
| 16379 | cg04952324 |
| 16380 | cg08371186 |
| 16381 | cg25654774 |
| 16382 | cg11391771 |
| 16383 | cg05400219 |
| 16384 | cg15200009 |
| 16385 | cg13655674 |
| 16386 | cg07264216 |
| 16387 | cg07189966 |
| 16388 | cg00920395 |
| 16389 | cg09914288 |
| 16390 | cg07647077 |
| 16391 | cg05727605 |
| 16392 | cg18720577 |
| 16393 | cg00674864 |
| 16394 | cg18559901 |
| 16395 | cg11300147 |
| 16396 | cg23013968 |
| 16397 | cg14631161 |
| 16398 | cg26673240 |
| 16399 | cg01384254 |
| 16400 | cg26426080 |
| 16401 | cg16303742 |
| 16402 | cg23163513 |
| 16403 | cg23800435 |
| 16404 | cg22046408 |
| 16405 | cg14790332 |
| 16406 | cg25065430 |
| 16407 | cg15580417 |
| 16408 | cg06800652 |
| 16409 | cg11182199 |
| 16410 | cg25838726 |
| 16411 | cg19763108 |
| 16412 | cg26920212 |
| 16413 | cg12829045 |
| 16414 | cg18602667 |
| 16415 | cg17768741 |
| 16416 | cg08321382 |
| 16417 | cg04744922 |
| 16418 | cg16568442 |
| 16419 | cg07044115 |
| 16420 | cg13675655 |
| 16421 | cg23797679 |
| 16422 | cg14518658 |
| 16423 | cg10594550 |
| 16424 | cg10067021 |
| 16425 | cg09935124 |
| 16426 | cg05945230 |
| 16427 | cg26211258 |
| 16428 | cg18059362 |
| 16429 | cg08802841 |
| 16430 | cg15542016 |
| 16431 | cg24644830 |
| 16432 | cg10122917 |
| 16433 | cg20155584 |
| 16434 | cg14037769 |
| 16435 | cg24349476 |
| 16436 | cg01268431 |
| 16437 | cg00331421 |
| 16438 | cg14074534 |
| 16439 | cg09788492 |
| 16440 | cg14043822 |
| 16441 | cg26786947 |
| 16442 | cg01502872 |
| 16443 | cg00366716 |
| 16444 | cg27434596 |
| 16445 | cg21821315 |
| 16446 | cg13236934 |
| 16447 | cg03064693 |
| 16448 | cg10122698 |
| 16449 | cg16281758 |
| 16450 | cg06802538 |
| 16451 | cg20852302 |
| 16452 | cg26243334 |
| 16453 | cg16596069 |
| 16454 | cg17783086 |
| 16455 | cg08024169 |
| 16456 | cg04051565 |
| 16457 | cg07175236 |
| 16458 | cg19828220 |
| 16459 | cg04132407 |
| 16460 | cg02636348 |
| 16461 | cg22531018 |
| 16462 | cg00090261 |
| 16463 | cg25546629 |
| 16464 | cg15584466 |
| 16465 | cg08813062 |
| 16466 | cg01519129 |
| 16467 | cg14435498 |
| 16468 | cg07802401 |
| 16469 | cg06666025 |
| 16470 | cg02260363 |
| 16471 | cg09330123 |
| 16472 | cg05602531 |
| 16473 | cg21633105 |
| 16474 | cg09655257 |
| 16475 | cg08733166 |
| 16476 | cg03533858 |
| 16477 | cg08173515 |
| 16478 | cg16169513 |
| 16479 | cg11794751 |
| 16480 | cg16470259 |
| 16481 | cg23404978 |
| 16482 | cg03391854 |
| 16483 | cg22666373 |
| 16484 | cg08165083 |
| 16485 | cg10657823 |
| 16486 | cg16169675 |
| 16487 | cg00811758 |
| 16488 | cg05755378 |
| 16489 | cg07617237 |
| 16490 | cg21848191 |
| 16491 | cg24116665 |
| 16492 | cg00635674 |
| 16493 | cg23823577 |
| 16494 | cg24644367 |
| 16495 | cg16710224 |
| 16496 | cg23452220 |
| 16497 | cg01959155 |
| 16498 | cg12711018 |
| 16499 | cg20110678 |
| 16500 | cg18119255 |
| 16501 | cg09987620 |
| 16502 | cg03873153 |
| 16503 | cg00121039 |
| 16504 | cg25810857 |
| 16505 | cg11401022 |
| 16506 | cg13425960 |
| 16507 | cg23881458 |
| 16508 | cg26412808 |
| 16509 | cg25492837 |
| 16510 | cg20715159 |
| 16511 | cg14605948 |
| 16512 | cg09681660 |
| 16513 | cg20727124 |
| 16514 | cg17394874 |
| 16515 | cg21471707 |
| 16516 | cg24378730 |
| 16517 | cg03394725 |
| 16518 | cg27310267 |
| 16519 | cg22990562 |
| 16520 | cg24571913 |
| 16521 | cg06640997 |
| 16522 | cg16139685 |
| 16523 | cg08430329 |
| 16524 | cg03337475 |
| 16525 | cg01056400 |
| 16526 | cg01112547 |
| 16527 | cg10217889 |
| 16528 | cg26095658 |
| 16529 | cg08234878 |
| 16530 | cg21532636 |
| 16531 | cg13922403 |
| 16532 | cg02510809 |
| 16533 | cg15683080 |
| 16534 | cg03099728 |
| 16535 | cg02576202 |
| 16536 | cg23121993 |
| 16537 | cg11420667 |
| 16538 | cg15244965 |
| 16539 | cg25338320 |
| 16540 | cg05866504 |
| 16541 | cg24186190 |
| 16542 | cg03201427 |
| 16543 | cg18232313 |
| 16544 | cg15279186 |
| 16545 | cg08888763 |
| 16546 | cg10195983 |
| 16547 | cg01730072 |
| 16548 | cg19433225 |
| 16549 | cg20902370 |
| 16550 | cg21803181 |
| 16551 | cg10868445 |
| 16552 | cg09100286 |
| 16553 | cg09948687 |
| 16554 | cg05083507 |
| 16555 | cg18499941 |
| 16556 | cg12982531 |
| 16557 | cg15910469 |
| 16558 | cg14430167 |
| 16559 | cg13750264 |
| 16560 | cg13190849 |
| 16561 | cg24261491 |
| 16562 | cg07433723 |
| 16563 | cg12676322 |
| 16564 | cg07377178 |
| 16565 | cg10285992 |
| 16566 | cg00067676 |
| 16567 | cg18463607 |
| 16568 | cg22922553 |
| 16569 | cg11496508 |
| 16570 | cg09017607 |
| 16571 | cg19753675 |
| 16572 | cg14049295 |
| 16573 | cg15720112 |
| 16574 | cg21541748 |
| 16575 | cg11815057 |
| 16576 | cg11127587 |
| 16577 | cg07609031 |
| 16578 | cg02288969 |
| 16579 | cg03156170 |
| 16580 | cg04159246 |
| 16581 | cg15717205 |
| 16582 | cg10895875 |
| 16583 | cg20387815 |
| 16584 | cg05539750 |
| 16585 | cg19948478 |
| 16586 | cg10386082 |
| 16587 | cg26452056 |
| 16588 | cg08691775 |
| 16589 | cg26280758 |
| 16590 | cg13519834 |
| 16591 | cg05357862 |
| 16592 | cg01397448 |
| 16593 | cg06563450 |
| 16594 | cg05490470 |
| 16595 | cg06459070 |
| 16596 | cg21425842 |
| 16597 | cg16881467 |
| 16598 | cg08706986 |
| 16599 | cg23989336 |
| 16600 | cg11974709 |
| 16601 | cg17709391 |
| 16602 | cg05227405 |
| 16603 | cg23870316 |
| 16604 | cg14393732 |
| 16605 | cg23625162 |
| 16606 | cg23576695 |
| 16607 | cg03992114 |
| 16608 | cg02799905 |
| 16609 | cg02281167 |
| 16610 | cg07951455 |
| 16611 | cg12423553 |
| 16612 | cg08932256 |
| 16613 | cg16196740 |
| 16614 | cg00055771 |
| 16615 | cg11925237 |
| 16616 | cg17655970 |
| 16617 | cg12598645 |
| 16618 | cg22407111 |
| 16619 | cg09998038 |
| 16620 | cg18270687 |
| 16621 | cg00616888 |
| 16622 | cg21251563 |
| 16623 | cg07436615 |
| 16624 | cg00810173 |
| 16625 | cg25449415 |
| 16626 | cg03024212 |
| 16627 | cg16872357 |
| 16628 | cg15469023 |
| 16629 | cg12368260 |
| 16630 | cg19334350 |
| 16631 | cg01898056 |
| 16632 | cg03720605 |
| 16633 | cg23552820 |
| 16634 | cg13318543 |
| 16635 | cg19953487 |
| 16636 | cg21727032 |
| 16637 | cg23632656 |
| 16638 | cg22154446 |
| 16639 | cg14544492 |
| 16640 | cg21194933 |
| 16641 | cg01882249 |
| 16642 | cg25206797 |
| 16643 | cg16028555 |
| 16644 | cg06321213 |
| 16645 | cg03637964 |
| 16646 | cg11190458 |
| 16647 | cg11268178 |
| 16648 | cg15251562 |
| 16649 | cg14503489 |
| 16650 | cg23023797 |
| 16651 | cg15263821 |
| 16652 | cg21123160 |
| 16653 | cg05383931 |
| 16654 | cg25913233 |
| 16655 | cg13782615 |
| 16656 | cg02503796 |
| 16657 | cg23622299 |
| 16658 | cg00423364 |
| 16659 | cg25331571 |
| 16660 | cg15799963 |
| 16661 | cg08015107 |
| 16662 | cg21571060 |
| 16663 | cg26353598 |
| 16664 | cg19920378 |
| 16665 | cg06802709 |
| 16666 | cg23023643 |
| 16667 | cg19739407 |
| 16668 | cg04110585 |
| 16669 | cg20776772 |
| 16670 | cg05210373 |
| 16671 | cg12222949 |
| 16672 | cg03879061 |
| 16673 | cg14727777 |
| 16674 | cg11415369 |
| 16675 | cg23864158 |
| 16676 | cg06210139 |
| 16677 | cg00363357 |
| 16678 | cg17382310 |
| 16679 | cg10296382 |
| 16680 | cg27084175 |
| 16681 | cg23199006 |
| 16682 | cg19016664 |
| 16683 | cg27097489 |
| 16684 | cg03973584 |
| 16685 | cg11555591 |
| 16686 | cg07617759 |
| 16687 | cg18629132 |
| 16688 | cg27311341 |
| 16689 | cg19419556 |
| 16690 | cg01730433 |
| 16691 | cg11745271 |
| 16692 | cg10131286 |
| 16693 | cg15167754 |
| 16694 | cg14988680 |
| 16695 | cg00230120 |
| 16696 | cg12584496 |
| 16697 | cg10672754 |
| 16698 | cg11487379 |
| 16699 | cg24287218 |
| 16700 | cg21913528 |
| 16701 | cg05447100 |
| 16702 | cg23839159 |
| 16703 | cg04082524 |
| 16704 | cg26625863 |
| 16705 | cg17005332 |
| 16706 | cg20442599 |
| 16707 | cg18254930 |
| 16708 | cg10535132 |
| 16709 | cg05313918 |
| 16710 | cg17500986 |
| 16711 | cg18813527 |
| 16712 | cg16658951 |
| 16713 | cg07690882 |
| 16714 | cg15487791 |
| 16715 | cg13815182 |
| 16716 | cg03691144 |
| 16717 | cg07878514 |
| 16718 | cg25585899 |
| 16719 | cg00237391 |
| 16720 | cg21249091 |
| 16721 | cg21885361 |
| 16722 | cg12829360 |
| 16723 | cg19061551 |
| 16724 | cg17796318 |
| 16725 | cg14997592 |
| 16726 | cg04632023 |
| 16727 | cg04784535 |
| 16728 | cg19940063 |
| 16729 | cg06679851 |
| 16730 | cg12505603 |
| 16731 | cg05033729 |
| 16732 | cg13169574 |
| 16733 | cg04304872 |
| 16734 | cg26724312 |
| 16735 | cg01850245 |
| 16736 | cg09384276 |
| 16737 | cg26268968 |
| 16738 | cg16876710 |
| 16739 | cg07364729 |
| 16740 | cg15775921 |
| 16741 | cg07749715 |
| 16742 | cg07804203 |
| 16743 | cg04772948 |
| 16744 | cg05730368 |
| 16745 | cg12601909 |
| 16746 | cg14456470 |
| 16747 | cg22239534 |
| 16748 | cg25902146 |
| 16749 | cg10237252 |
| 16750 | cg07948401 |
| 16751 | cg17043722 |
| 16752 | cg03756721 |
| 16753 | cg02419922 |
| 16754 | cg27654476 |
| 16755 | cg18019314 |
| 16756 | cg26664556 |
| 16757 | cg03181300 |
| 16758 | cg11492043 |
| 16759 | cg04505284 |
| 16760 | cg07644125 |
| 16761 | cg22693849 |
| 16762 | cg06651904 |
| 16763 | cg21854408 |
| 16764 | cg12303240 |
| 16765 | cg22677556 |
| 16766 | cg00933542 |
| 16767 | cg16749242 |
| 16768 | cg07813265 |
| 16769 | cg02776658 |
| 16770 | cg24691398 |
| 16771 | cg02970696 |
| 16772 | cg11469587 |
| 16773 | cg14141340 |
| 16774 | cg00107916 |
| 16775 | cg11957848 |
| 16776 | cg06612016 |
| 16777 | cg14206473 |
| 16778 | cg01391193 |
| 16779 | cg07230652 |
| 16780 | cg12961069 |
| 16781 | cg22232707 |
| 16782 | cg11961495 |
| 16783 | cg01798157 |
| 16784 | cg16832407 |
| 16785 | cg20658081 |
| 16786 | cg00306523 |
| 16787 | cg12145624 |
| 16788 | cg06157318 |
| 16789 | cg18482112 |
| 16790 | cg12749863 |
| 16791 | cg14916909 |
| 16792 | cg14591730 |
| 16793 | cg00188488 |
| 16794 | cg21464975 |
| 16795 | cg03898692 |
| 16796 | cg08665903 |
| 16797 | cg01469715 |
| 16798 | cg18762722 |
| 16799 | cg14385233 |
| 16800 | cg15420468 |
| 16801 | cg18370700 |
| 16802 | cg05435490 |
| 16803 | cg00091302 |
| 16804 | cg20740051 |
| 16805 | cg21212139 |
| 16806 | cg16546017 |
| 16807 | cg07803218 |
| 16808 | cg02796135 |
| 16809 | cg05487507 |
| 16810 | cg01025883 |
| 16811 | cg16733226 |
| 16812 | cg17931356 |
| 16813 | cg21760537 |
| 16814 | cg15451784 |
| 16815 | cg25977206 |
| 16816 | cg12557374 |
| 16817 | cg26815032 |
| 16818 | cg04276413 |
| 16819 | cg26076666 |
| 16820 | cg20972255 |
| 16821 | cg15563591 |
| 16822 | cg10395101 |
| 16823 | cg15878708 |
| 16824 | cg14258285 |
| 16825 | cg01691696 |
| 16826 | cg07315426 |
| 16827 | cg16298547 |
| 16828 | cg20341535 |
| 16829 | cg07805058 |
| 16830 | cg10453602 |
| 16831 | cg10953604 |
| 16832 | cg17284609 |
| 16833 | cg15894129 |
| 16834 | cg10767661 |
| 16835 | cg02801685 |
| 16836 | cg00124045 |
| 16837 | cg17711521 |
| 16838 | cg24214818 |
| 16839 | cg05600609 |
| 16840 | cg13315995 |
| 16841 | cg08719642 |
| 16842 | cg05451388 |
| 16843 | cg12938891 |
| 16844 | cg02158978 |
| 16845 | cg22021934 |
| 16846 | cg26884773 |
| 16847 | cg08482215 |
| 16848 | cg19685719 |
| 16849 | cg24357619 |
| 16850 | cg16265542 |
| 16851 | cg08398567 |
| 16852 | cg04677581 |
| 16853 | cg03388979 |
| 16854 | cg20213491 |
| 16855 | cg02514398 |
| 16856 | cg26895768 |
| 16857 | cg23294090 |
| 16858 | cg18130905 |
| 16859 | cg23601468 |
| 16860 | cg08510234 |
| 16861 | cg10847408 |
| 16862 | cg22317573 |
| 16863 | cg23833951 |
| 16864 | cg06497848 |
| 16865 | cg02072170 |
| 16866 | cg04585311 |
| 16867 | cg21827532 |
| 16868 | cg21643619 |
| 16869 | cg06490751 |
| 16870 | cg04429609 |
| 16871 | cg12731671 |
| 16872 | cg02437406 |
| 16873 | cg27583677 |
| 16874 | cg08527797 |
| 16875 | cg09154528 |
| 16876 | cg20585208 |
| 16877 | cg06976144 |
| 16878 | cg02364236 |
| 16879 | cg07126617 |
| 16880 | cg04909357 |
| 16881 | cg17980032 |
| 16882 | cg02161940 |
| 16883 | cg14054275 |
| 16884 | cg19676181 |
| 16885 | cg26700277 |
| 16886 | cg24158209 |
| 16887 | cg22918656 |
| 16888 | cg15465743 |
| 16889 | cg07033621 |
| 16890 | cg07165167 |
| 16891 | cg03827118 |
| 16892 | cg09455342 |
| 16893 | cg12542656 |
| 16894 | cg24179979 |
| 16895 | cg25318809 |
| 16896 | cg24189381 |
| 16897 | cg14534180 |
| 16898 | cg16198225 |
| 16899 | cg15816817 |
| 16900 | cg05603527 |
| 16901 | cg09133032 |
| 16902 | cg17151172 |
| 16903 | cg21166844 |
| 16904 | cg05572461 |
| 16905 | cg03181248 |
| 16906 | cg12912150 |
| 16907 | cg13755830 |
| 16908 | cg18616220 |
| 16909 | cg02532096 |
| 16910 | cg24819835 |
| 16911 | cg10725229 |
| 16912 | cg25464950 |
| 16913 | cg15323840 |
| 16914 | cg07497659 |
| 16915 | cg04948346 |
| 16916 | cg03255113 |
| 16917 | cg23725208 |
| 16918 | cg22578433 |
| 16919 | cg12356793 |
| 16920 | cg07623256 |
| 16921 | cg13481969 |
| 16922 | cg25645028 |
| 16923 | cg10370591 |
| 16924 | cg25456068 |
| 16925 | cg06300884 |
| 16926 | cg10028884 |
| 16927 | cg03375864 |
| 16928 | cg01820561 |
| 16929 | cg08677047 |
| 16930 | cg11877857 |
| 16931 | cg23012886 |
| 16932 | cg26825850 |
| 16933 | cg18073545 |
| 16934 | cg16118539 |
| 16935 | cg07951355 |
| 16936 | cg07124927 |
| 16937 | cg23958704 |
| 16938 | cg13936230 |
| 16939 | cg18272292 |
| 16940 | cg11958128 |
| 16941 | cg05867304 |
| 16942 | cg13461178 |
| 16943 | cg09131317 |
| 16944 | cg14360907 |
| 16945 | cg02208236 |
| 16946 | cg08471158 |
| 16947 | cg08122151 |
| 16948 | cg25028792 |
| 16949 | cg09905178 |
| 16950 | cg20975297 |
| 16951 | cg17512474 |
| 16952 | cg07388018 |
| 16953 | cg12336537 |
| 16954 | cg06439907 |
| 16955 | cg22768129 |
| 16956 | cg03275396 |
| 16957 | cg07608719 |
| 16958 | cg07632934 |
| 16959 | cg18093120 |
| 16960 | cg01277506 |
| 16961 | cg05004770 |
| 16962 | cg24364120 |
| 16963 | cg25792999 |
| 16964 | cg12724901 |
| 16965 | cg04863982 |
| 16966 | cg09387522 |
| 16967 | cg12761707 |
| 16968 | cg08474826 |
| 16969 | cg06790168 |
| 16970 | cg03950476 |
| 16971 | cg25232236 |
| 16972 | cg05161374 |
| 16973 | cg13716200 |
| 16974 | cg03530994 |
| 16975 | cg11735997 |
| 16976 | cg27027827 |
| 16977 | cg03323597 |
| 16978 | cg13112404 |
| 16979 | cg25357666 |
| 16980 | cg13677341 |
| 16981 | cg18099058 |
| 16982 | cg11088536 |
| 16983 | cg05595517 |
| 16984 | cg00347198 |
| 16985 | cg07204479 |
| 16986 | cg22209929 |
| 16987 | cg13950578 |
| 16988 | cg09974253 |
| 16989 | cg00176231 |
| 16990 | cg13115898 |
| 16991 | cg24605576 |
| 16992 | cg16958658 |
| 16993 | cg06898214 |
| 16994 | cg20250126 |
| 16995 | cg02534163 |
| 16996 | cg13757089 |
| 16997 | cg02541672 |
| 16998 | cg26879644 |
| 16999 | cg10233698 |
| 17000 | cg01906119 |
| 17001 | cg27543040 |
| 17002 | cg15228341 |
| 17003 | cg07986251 |
| 17004 | cg25007705 |
| 17005 | cg07069934 |
| 17006 | cg06972953 |
| 17007 | cg16224812 |
| 17008 | cg13574911 |
| 17009 | cg16259229 |
| 17010 | cg21801277 |
| 17011 | cg24404533 |
| 17012 | cg27256256 |
| 17013 | cg12267166 |
| 17014 | cg02678971 |
| 17015 | cg11099300 |
| 17016 | cg07437284 |
| 17017 | cg00247830 |
| 17018 | cg11932292 |
| 17019 | cg10464968 |
| 17020 | cg07755689 |
| 17021 | cg19157500 |
| 17022 | cg12621285 |
| 17023 | cg12130725 |
| 17024 | cg17476701 |
| 17025 | cg02278646 |
| 17026 | cg22992497 |
| 17027 | cg17015522 |
| 17028 | cg02786313 |
| 17029 | cg25773267 |
| 17030 | cg24605747 |
| 17031 | cg06943988 |
| 17032 | cg11972924 |
| 17033 | cg21325808 |
| 17034 | cg13139972 |
| 17035 | cg01764953 |
| 17036 | cg13488501 |
| 17037 | cg21795544 |
| 17038 | cg12394215 |
| 17039 | cg14868760 |
| 17040 | cg04103896 |
| 17041 | cg13219825 |
| 17042 | cg26033526 |
| 17043 | cg06306558 |
| 17044 | cg19543269 |
| 17045 | cg14112935 |
| 17046 | cg12914509 |
| 17047 | cg13523649 |
| 17048 | cg11132908 |
| 17049 | cg27086356 |
| 17050 | cg17104824 |
| 17051 | cg00158122 |
| 17052 | cg26736929 |
| 17053 | cg09252461 |
| 17054 | cg25447164 |
| 17055 | cg25660390 |
| 17056 | cg21239179 |
| 17057 | cg09231976 |
| 17058 | cg09122838 |
| 17059 | cg26364947 |
| 17060 | cg23411869 |
| 17061 | cg16316923 |
| 17062 | cg00376255 |
| 17063 | cg07024568 |
| 17064 | cg10401537 |
| 17065 | cg13520278 |
| 17066 | cg10237044 |
| 17067 | cg16259985 |
| 17068 | cg24642022 |
| 17069 | cg03198010 |
| 17070 | cg23701971 |
| 17071 | cg05076877 |
| 17072 | cg09966455 |
| 17073 | cg19610529 |
| 17074 | cg03927470 |
| 17075 | cg05961022 |
| 17076 | cg21221540 |
| 17077 | cg25900541 |
| 17078 | cg01966891 |
| 17079 | cg26883837 |
| 17080 | cg22010446 |
| 17081 | cg20630582 |
| 17082 | cg23641117 |
| 17083 | cg11384475 |
| 17084 | cg27159689 |
| 17085 | cg04346189 |
| 17086 | cg03319497 |
| 17087 | cg22384261 |
| 17088 | cg08713159 |
| 17089 | cg05104052 |
| 17090 | cg10220843 |
| 17091 | cg12948621 |
| 17092 | cg00728371 |
| 17093 | cg15415783 |
| 17094 | cg07132068 |
| 17095 | cg05225883 |
| 17096 | cg05420685 |
| 17097 | cg09256941 |
| 17098 | cg06618082 |
| 17099 | cg16004738 |
| 17100 | cg26913248 |
| 17101 | cg18863582 |
| 17102 | cg20703997 |
| 17103 | cg13218710 |
| 17104 | cg22101977 |
| 17105 | cg09107315 |
| 17106 | cg17501574 |
| 17107 | cg00470947 |
| 17108 | cg20113824 |
| 17109 | cg22320399 |
| 17110 | cg19735903 |
| 17111 | cg09901400 |
| 17112 | cg05898470 |
| 17113 | cg07025752 |
| 17114 | cg08491437 |
| 17115 | cg05246613 |
| 17116 | cg18841634 |
| 17117 | cg25236050 |
| 17118 | cg25602543 |
| 17119 | cg02675724 |
| 17120 | cg03560416 |
| 17121 | cg26397549 |
| 17122 | cg09421083 |
| 17123 | cg01654432 |
| 17124 | cg01355382 |
| 17125 | cg23893681 |
| 17126 | cg27197639 |
| 17127 | cg23391090 |
| 17128 | cg24281777 |
| 17129 | cg02626129 |
| 17130 | cg17501395 |
| 17131 | cg24795351 |
| 17132 | cg07429038 |
| 17133 | cg12777862 |
| 17134 | cg02857726 |
| 17135 | cg23706586 |
| 17136 | cg00998782 |
| 17137 | cg16034795 |
| 17138 | cg09753772 |
| 17139 | cg14057562 |
| 17140 | cg04955912 |
| 17141 | cg23651872 |
| 17142 | cg14773178 |
| 17143 | cg12954182 |
| 17144 | cg03321133 |
| 17145 | cg22952142 |
| 17146 | cg11339384 |
| 17147 | cg20786687 |
| 17148 | cg26530319 |
| 17149 | cg11604426 |
| 17150 | cg03231596 |
| 17151 | cg27462398 |
| 17152 | cg07451222 |
| 17153 | cg22685705 |
| 17154 | cg18395531 |
| 17155 | cg11635839 |
| 17156 | cg03091982 |
| 17157 | cg16956773 |
| 17158 | cg24148085 |
| 17159 | cg04911050 |
| 17160 | cg05487307 |
| 17161 | cg14619528 |
| 17162 | cg07161968 |
| 17163 | cg02366189 |
| 17164 | cg11157235 |
| 17165 | cg04396781 |
| 17166 | cg26431550 |
| 17167 | cg27599211 |
| 17168 | cg17208001 |
| 17169 | cg00241745 |
| 17170 | cg18272307 |
| 17171 | cg12504148 |
| 17172 | cg16865965 |
| 17173 | cg22370326 |
| 17174 | cg13185961 |
| 17175 | cg03472063 |
| 17176 | cg23713520 |
| 17177 | cg20824635 |
| 17178 | cg08469646 |
| 17179 | cg03141011 |
| 17180 | cg13065415 |
| 17181 | cg01748991 |
| 17182 | cg14092276 |
| 17183 | cg19362787 |
| 17184 | cg02152068 |
| 17185 | cg04236117 |
| 17186 | cg07316873 |
| 17187 | cg06893288 |
| 17188 | cg06937717 |
| 17189 | cg09435527 |
| 17190 | cg22703520 |
| 17191 | cg11817899 |
| 17192 | cg02696685 |
| 17193 | cg19241424 |
| 17194 | cg18195560 |
| 17195 | cg27082467 |
| 17196 | cg17012248 |
| 17197 | cg05390307 |
| 17198 | cg06620896 |
| 17199 | cg01889070 |
| 17200 | cg01835399 |
| 17201 | cg13080721 |
| 17202 | cg02218644 |
| 17203 | cg05133853 |
| 17204 | cg01235215 |
| 17205 | cg01347842 |
| 17206 | cg27072481 |
| 17207 | cg02631126 |
| 17208 | cg12585232 |
| 17209 | cg05570231 |
| 17210 | cg23744697 |
| 17211 | cg17809377 |
| 17212 | cg00011173 |
| 17213 | cg22490454 |
| 17214 | cg00330581 |
| 17215 | cg10727171 |
| 17216 | cg22410478 |
| 17217 | cg07254165 |
| 17218 | cg06151468 |
| 17219 | cg17863035 |
| 17220 | cg14350325 |
| 17221 | cg12566938 |
| 17222 | cg18049267 |
| 17223 | cg23885371 |
| 17224 | cg09720701 |
| 17225 | cg07511668 |
| 17226 | cg12337480 |
| 17227 | cg17963157 |
| 17228 | cg20651353 |
| 17229 | cg25159064 |
| 17230 | cg00816613 |
| 17231 | cg08774902 |
| 17232 | cg25230743 |
| 17233 | cg01345315 |
| 17234 | cg18561782 |
| 17235 | cg03572617 |
| 17236 | cg14016533 |
| 17237 | cg00555456 |
| 17238 | cg17347063 |
| 17239 | cg22658758 |
| 17240 | cg08071970 |
| 17241 | cg11375297 |
| 17242 | cg12486169 |
| 17243 | cg25750114 |
| 17244 | cg05400732 |
| 17245 | cg17475172 |
| 17246 | cg14428237 |
| 17247 | cg22871175 |
| 17248 | cg09833224 |
| 17249 | cg01471709 |
| 17250 | cg05867210 |
| 17251 | cg12636325 |
| 17252 | cg04849574 |
| 17253 | cg14458815 |
| 17254 | cg11913577 |
| 17255 | cg01537995 |
| 17256 | cg13791558 |
| 17257 | cg03592051 |
| 17258 | cg12149609 |
| 17259 | cg08469326 |
| 17260 | cg04781339 |
| 17261 | cg26191447 |
| 17262 | cg02542748 |
| 17263 | cg16851046 |
| 17264 | cg21335583 |
| 17265 | cg03287339 |
| 17266 | cg15151492 |
| 17267 | cg01413771 |
| 17268 | cg14780466 |
| 17269 | cg20160563 |
| 17270 | cg00499500 |
| 17271 | cg09214243 |
| 17272 | cg20985193 |
| 17273 | cg18898325 |
| 17274 | cg26484896 |
| 17275 | cg27227250 |
| 17276 | cg15877238 |
| 17277 | cg09964012 |
| 17278 | cg02744604 |
| 17279 | cg11722581 |
| 17280 | cg01302228 |
| 17281 | cg07139762 |
| 17282 | cg14169284 |
| 17283 | cg08832418 |
| 17284 | cg03135983 |
| 17285 | cg14816778 |
| 17286 | cg12053816 |
| 17287 | cg26784011 |
| 17288 | cg16656411 |
| 17289 | cg03234839 |
| 17290 | cg02486421 |
| 17291 | cg04344860 |
| 17292 | cg16195332 |
| 17293 | cg19035612 |
| 17294 | cg15815333 |
| 17295 | cg23248083 |
| 17296 | cg23209684 |
| 17297 | cg00656630 |
| 17298 | cg09698471 |
| 17299 | cg07081633 |
| 17300 | cg15058545 |
| 17301 | cg01789926 |
| 17302 | cg13288223 |
| 17303 | cg04371749 |
| 17304 | cg18118442 |
| 17305 | cg22543665 |
| 17306 | cg03877420 |
| 17307 | cg00498129 |
| 17308 | cg07675998 |
| 17309 | cg00091953 |
| 17310 | cg08414403 |
| 17311 | cg03233624 |
| 17312 | cg02580969 |
| 17313 | cg15316817 |
| 17314 | cg02743569 |
| 17315 | cg17474973 |
| 17316 | cg05320842 |
| 17317 | cg15633035 |
| 17318 | cg04061618 |
| 17319 | cg16934374 |
| 17320 | cg00688591 |
| 17321 | cg13332832 |
| 17322 | cg09782833 |
| 17323 | cg19560927 |
| 17324 | cg14952641 |
| 17325 | cg14620572 |
| 17326 | cg09608949 |
| 17327 | cg26400885 |
| 17328 | cg04813697 |
| 17329 | cg12979009 |
| 17330 | cg17201760 |
| 17331 | cg06694446 |
| 17332 | cg06700063 |
| 17333 | cg16334425 |
| 17334 | cg19334622 |
| 17335 | cg23688510 |
| 17336 | cg02555368 |
| 17337 | cg03284866 |
| 17338 | cg20453370 |
| 17339 | cg23333125 |
| 17340 | cg21418575 |
| 17341 | cg11931762 |
| 17342 | cg17122870 |
| 17343 | cg00236991 |
| 17344 | cg00812833 |
| 17345 | cg03488840 |
| 17346 | cg09778927 |
| 17347 | cg15030392 |
| 17348 | cg26968378 |
| 17349 | cg09988805 |
| 17350 | cg07705650 |
| 17351 | cg24477733 |
| 17352 | cg14644378 |
| 17353 | cg08029281 |
| 17354 | cg00283887 |
| 17355 | cg15895526 |
| 17356 | cg25715909 |
| 17357 | cg09454102 |
| 17358 | cg04850211 |
| 17359 | cg25921502 |
| 17360 | cg07436286 |
| 17361 | cg20343567 |
| 17362 | cg04202774 |
| 17363 | cg05207924 |
| 17364 | cg22043544 |
| 17365 | cg08180070 |
| 17366 | cg10507346 |
| 17367 | cg05793240 |
| 17368 | cg25320523 |
| 17369 | cg23158916 |
| 17370 | cg19673155 |
| 17371 | cg09079855 |
| 17372 | cg06271630 |
| 17373 | cg08747153 |
| 17374 | cg04762199 |
| 17375 | cg17221251 |
| 17376 | cg10794973 |
| 17377 | cg22338960 |
| 17378 | cg08812936 |
| 17379 | cg02458062 |
| 17380 | cg03821354 |
| 17381 | cg11591636 |
| 17382 | cg01485767 |
| 17383 | cg04736556 |
| 17384 | cg18771553 |
| 17385 | cg21778193 |
| 17386 | cg22253677 |
| 17387 | cg00015193 |
| 17388 | cg19822343 |
| 17389 | cg02873421 |
| 17390 | cg27618173 |
| 17391 | cg26981268 |
| 17392 | cg02411275 |
| 17393 | cg14701491 |
| 17394 | cg27336368 |
| 17395 | cg13158235 |
| 17396 | cg26861333 |
| 17397 | cg25614726 |
| 17398 | cg21610815 |
| 17399 | cg06098548 |
| 17400 | cg16496507 |
| 17401 | cg24355048 |
| 17402 | cg19076536 |
| 17403 | cg27430561 |
| 17404 | cg23699463 |
| 17405 | cg26985203 |
| 17406 | cg16751778 |
| 17407 | cg22450120 |
| 17408 | cg05379634 |
| 17409 | cg15867307 |
| 17410 | cg14906103 |
| 17411 | cg04636166 |
| 17412 | cg03515479 |
| 17413 | cg26148923 |
| 17414 | cg26394071 |
| 17415 | cg04322202 |
| 17416 | cg26939277 |
| 17417 | cg05477778 |
| 17418 | cg10522607 |
| 17419 | cg00372375 |
| 17420 | cg19514381 |
| 17421 | cg06602086 |
| 17422 | cg23746574 |
| 17423 | cg15192103 |
| 17424 | cg23902686 |
| 17425 | cg11375451 |
| 17426 | cg04508912 |
| 17427 | cg12609829 |
| 17428 | cg26158447 |
| 17429 | cg11626496 |
| 17430 | cg07374666 |
| 17431 | cg02295938 |
| 17432 | cg25148572 |
| 17433 | cg01895259 |
| 17434 | cg06867542 |
| 17435 | cg13440544 |
| 17436 | cg02096296 |
| 17437 | cg25473438 |
| 17438 | cg05976923 |
| 17439 | cg18121824 |
| 17440 | cg15123428 |
| 17441 | cg14783600 |
| 17442 | cg01112258 |
| 17443 | cg04311455 |
| 17444 | cg12544812 |
| 17445 | cg06708315 |
| 17446 | cg16998578 |
| 17447 | cg23983517 |
| 17448 | cg12350592 |
| 17449 | cg25103953 |
| 17450 | cg26213252 |
| 17451 | cg12298697 |
| 17452 | cg21419404 |
| 17453 | cg10460063 |
| 17454 | cg13699771 |
| 17455 | cg01313513 |
| 17456 | cg00346024 |
| 17457 | cg21977522 |
| 17458 | cg07703765 |
| 17459 | cg16478734 |
| 17460 | cg22931087 |
| 17461 | cg25135233 |
| 17462 | cg11599718 |
| 17463 | cg25487775 |
| 17464 | cg02592957 |
| 17465 | cg17168460 |
| 17466 | cg00187557 |
| 17467 | cg16500026 |
| 17468 | cg15370010 |
| 17469 | cg00096536 |
| 17470 | cg05771324 |
| 17471 | cg26643813 |
| 17472 | cg13611686 |
| 17473 | cg12288061 |
| 17474 | cg06636678 |
| 17475 | cg09215260 |
| 17476 | cg12977772 |
| 17477 | cg00713280 |
| 17478 | cg23371434 |
| 17479 | cg00894715 |
| 17480 | cg13296570 |
| 17481 | cg17280705 |
| 17482 | cg11822515 |
| 17483 | cg01092133 |
| 17484 | cg04440361 |
| 17485 | cg20311501 |
| 17486 | cg17178175 |
| 17487 | cg24595093 |
| 17488 | cg10848373 |
| 17489 | cg04679620 |
| 17490 | cg24562906 |
| 17491 | cg14530584 |
| 17492 | cg05904338 |
| 17493 | cg12002745 |
| 17494 | cg03585053 |
| 17495 | cg18466965 |
| 17496 | cg00048759 |
| 17497 | cg01669927 |
| 17498 | cg22304507 |
| 17499 | cg05673214 |
| 17500 | cg19098437 |
| 17501 | cg11032707 |
| 17502 | cg10818566 |
| 17503 | cg03743191 |
| 17504 | cg23907053 |
| 17505 | cg26579986 |
| 17506 | cg20376277 |
| 17507 | cg13102045 |
| 17508 | cg11818092 |
| 17509 | cg05765510 |
| 17510 | cg11059973 |
| 17511 | cg06288489 |
| 17512 | cg05905844 |
| 17513 | cg12763828 |
| 17514 | cg02446073 |
| 17515 | cg06688960 |
| 17516 | cg19067730 |
| 17517 | cg21184439 |
| 17518 | cg14134368 |
| 17519 | cg07047589 |
| 17520 | cg20483671 |
| 17521 | cg11462099 |
| 17522 | cg02883053 |
| 17523 | cg12759288 |
| 17524 | cg20339843 |
| 17525 | cg10379724 |
| 17526 | cg27196715 |
| 17527 | cg14511652 |
| 17528 | cg06265252 |
| 17529 | cg02211983 |
| 17530 | cg23852520 |
| 17531 | cg16185834 |
| 17532 | cg01771019 |
| 17533 | cg17397672 |
| 17534 | cg04428805 |
| 17535 | cg17717993 |
| 17536 | cg11947548 |
| 17537 | cg14747847 |
| 17538 | cg25901651 |
| 17539 | cg15877165 |
| 17540 | cg08122156 |
| 17541 | cg00362812 |
| 17542 | cg08894980 |
| 17543 | cg21995171 |
| 17544 | cg02914589 |
| 17545 | cg14221171 |
| 17546 | cg02456074 |
| 17547 | cg22346570 |
| 17548 | cg13622788 |
| 17549 | cg07950469 |
| 17550 | cg12637676 |
| 17551 | cg08000432 |
| 17552 | cg07612396 |
| 17553 | cg20259011 |
| 17554 | cg09470638 |
| 17555 | cg24187177 |
| 17556 | cg22316451 |
| 17557 | cg02008285 |
| 17558 | cg03112057 |
| 17559 | cg08831252 |
| 17560 | cg09020213 |
| 17561 | cg01709189 |
| 17562 | cg25503169 |
| 17563 | cg01053551 |
| 17564 | cg01731630 |
| 17565 | cg24240763 |
| 17566 | cg23591302 |
| 17567 | cg15964537 |
| 17568 | cg21464258 |
| 17569 | cg10388583 |
| 17570 | cg20650823 |
| 17571 | cg10997203 |
| 17572 | cg10813029 |
| 17573 | cg00432203 |
| 17574 | cg00911794 |
| 17575 | cg06294416 |
| 17576 | cg14015525 |
| 17577 | cg19008097 |
| 17578 | cg10230190 |
| 17579 | cg16364805 |
| 17580 | cg13350917 |
| 17581 | cg11702934 |
| 17582 | cg11230435 |
| 17583 | cg10841124 |
| 17584 | cg18067096 |
| 17585 | cg12858902 |
| 17586 | cg12161599 |
| 17587 | cg06719604 |
| 17588 | cg20593464 |
| 17589 | cg10723962 |
| 17590 | cg01313313 |
| 17591 | cg10056460 |
| 17592 | cg25318962 |
| 17593 | cg20024459 |
| 17594 | cg18371186 |
| 17595 | cg18593842 |
| 17596 | cg06007668 |
| 17597 | cg04078644 |
| 17598 | cg24432675 |
| 17599 | cg04927066 |
| 17600 | cg06716419 |
| 17601 | cg08899182 |
| 17602 | cg13717768 |
| 17603 | cg00133624 |
| 17604 | cg14310109 |
| 17605 | cg07074086 |
| 17606 | cg09938479 |
| 17607 | cg15232708 |
| 17608 | cg10309769 |
| 17609 | cg16178065 |
| 17610 | cg20218073 |
| 17611 | cg14331765 |
| 17612 | cg12749540 |
| 17613 | cg23303246 |
| 17614 | cg12448102 |
| 17615 | cg18883033 |
| 17616 | cg04628802 |
| 17617 | cg24871887 |
| 17618 | cg24636368 |
| 17619 | cg05086444 |
| 17620 | cg07164152 |
| 17621 | cg24773040 |
| 17622 | cg21483999 |
| 17623 | cg22304593 |
| 17624 | cg05408680 |
| 17625 | cg23619936 |
| 17626 | cg03224756 |
| 17627 | cg09141304 |
| 17628 | cg25432735 |
| 17629 | cg17625389 |
| 17630 | cg19519355 |
| 17631 | cg09012269 |
| 17632 | cg08532895 |
| 17633 | cg26127201 |
| 17634 | cg17702692 |
| 17635 | cg10411590 |
| 17636 | cg03143830 |
| 17637 | cg11029191 |
| 17638 | cg16059943 |
| 17639 | cg20820107 |
| 17640 | cg10781276 |
| 17641 | cg18711009 |
| 17642 | cg02952121 |
| 17643 | cg14768157 |
| 17644 | cg05228402 |
| 17645 | cg21886089 |
| 17646 | cg06090895 |
| 17647 | cg06686156 |
| 17648 | cg10809166 |
| 17649 | cg23778943 |
| 17650 | cg19776344 |
| 17651 | cg20454887 |
| 17652 | cg11638298 |
| 17653 | cg06744792 |
| 17654 | cg26180080 |
| 17655 | cg01111006 |
| 17656 | cg04338863 |
| 17657 | cg22871485 |
| 17658 | cg02556436 |
| 17659 | cg22145560 |
| 17660 | cg03261948 |
| 17661 | cg13857210 |
| 17662 | cg15973075 |
| 17663 | cg24449777 |
| 17664 | cg11810135 |
| 17665 | cg13321166 |
| 17666 | cg13213931 |
| 17667 | cg14344157 |
| 17668 | cg23952740 |
| 17669 | cg04836949 |
| 17670 | cg00517605 |
| 17671 | cg13227982 |
| 17672 | cg00947796 |
| 17673 | cg00236467 |
| 17674 | cg23307680 |
| 17675 | cg25953788 |
| 17676 | cg08683644 |
| 17677 | cg07143125 |
| 17678 | cg04377610 |
| 17679 | cg08883204 |
| 17680 | cg01355753 |
| 17681 | cg15853475 |
| 17682 | cg26236192 |
| 17683 | cg03721175 |
| 17684 | cg19235893 |
| 17685 | cg15543199 |
| 17686 | cg20479009 |
| 17687 | cg21781706 |
| 17688 | cg21825111 |
| 17689 | cg07736457 |
| 17690 | cg08540275 |
| 17691 | cg04458053 |
| 17692 | cg05611414 |
| 17693 | cg12250896 |
| 17694 | cg01940429 |
| 17695 | cg06624527 |
| 17696 | cg13819230 |
| 17697 | cg07140888 |
| 17698 | cg24432464 |
| 17699 | cg11822447 |
| 17700 | cg18343292 |
| 17701 | cg13219226 |
| 17702 | cg09496585 |
| 17703 | cg14476095 |
| 17704 | cg18226451 |
| 17705 | cg07360720 |
| 17706 | cg15855900 |
| 17707 | cg20578405 |
| 17708 | cg06303744 |
| 17709 | cg21760404 |
| 17710 | cg07757611 |
| 17711 | cg06996956 |
| 17712 | cg19309256 |
| 17713 | cg19754622 |
| 17714 | cg12748266 |
| 17715 | cg04384208 |
| 17716 | cg04101934 |
| 17717 | cg25278191 |
| 17718 | cg07729608 |
| 17719 | cg05493394 |
| 17720 | cg21721566 |
| 17721 | cg05962042 |
| 17722 | cg16406131 |
| 17723 | cg24529736 |
| 17724 | cg18637832 |
| 17725 | cg02860232 |
| 17726 | cg02930996 |
| 17727 | cg05790077 |
| 17728 | cg23610453 |
| 17729 | cg22775801 |
| 17730 | cg03416521 |
| 17731 | cg03851835 |
| 17732 | cg07483086 |
| 17733 | cg10144554 |
| 17734 | cg06473507 |
| 17735 | cg10851010 |
| 17736 | cg14537825 |
| 17737 | cg15456144 |
| 17738 | cg20721135 |
| 17739 | cg24525238 |
| 17740 | cg26462586 |
| 17741 | cg07178825 |
| 17742 | cg02837876 |
| 17743 | cg20987369 |
| 17744 | cg18831425 |
| 17745 | cg04336859 |
| 17746 | cg12466669 |
| 17747 | cg18509524 |
| 17748 | cg27248029 |
| 17749 | cg01220592 |
| 17750 | cg12711196 |
| 17751 | cg14471540 |
| 17752 | cg02300584 |
| 17753 | cg06246351 |
| 17754 | cg24070814 |
| 17755 | cg01284858 |
| 17756 | cg25131101 |
| 17757 | cg17334762 |
| 17758 | cg06726820 |
| 17759 | cg18950540 |
| 17760 | cg24124740 |
| 17761 | cg00676421 |
| 17762 | cg16686757 |
| 17763 | cg03760308 |
| 17764 | cg09652503 |
| 17765 | cg27052152 |
| 17766 | cg16279861 |
| 17767 | cg02903275 |
| 17768 | cg02348449 |
| 17769 | cg26992963 |
| 17770 | cg22132982 |
| 17771 | cg10221896 |
| 17772 | cg16511396 |
| 17773 | cg01033844 |
| 17774 | cg21289800 |
| 17775 | cg08783300 |
| 17776 | cg00227156 |
| 17777 | cg23414376 |
| 17778 | cg00469380 |
| 17779 | cg09363841 |
| 17780 | cg07292612 |
| 17781 | cg00574379 |
| 17782 | cg25640931 |
| 17783 | cg02536253 |
| 17784 | cg20116936 |
| 17785 | cg16620719 |
| 17786 | cg14937617 |
| 17787 | cg05011554 |
| 17788 | cg02238549 |
| 17789 | cg02936292 |
| 17790 | cg06373648 |
| 17791 | cg24905385 |
| 17792 | cg01393447 |
| 17793 | cg25730047 |
| 17794 | cg26781701 |
| 17795 | cg11540018 |
| 17796 | cg18995275 |
| 17797 | cg14434876 |
| 17798 | cg16334795 |
| 17799 | cg07952789 |
| 17800 | cg21835860 |
| 17801 | cg04801085 |
| 17802 | cg18361375 |
| 17803 | cg03330181 |
| 17804 | cg20262477 |
| 17805 | cg05269287 |
| 17806 | cg06416034 |
| 17807 | cg25227866 |
| 17808 | cg05136264 |
| 17809 | cg03764357 |
| 17810 | cg16123062 |
| 17811 | cg09279927 |
| 17812 | cg05958352 |
| 17813 | cg08903441 |
| 17814 | cg15082473 |
| 17815 | cg21021972 |
| 17816 | cg02192678 |
| 17817 | cg25067242 |
| 17818 | cg19958368 |
| 17819 | cg06594122 |
| 17820 | cg21967134 |
| 17821 | cg04281750 |
| 17822 | cg08302650 |
| 17823 | cg01647693 |
| 17824 | cg09489639 |
| 17825 | cg08141172 |
| 17826 | cg11909908 |
| 17827 | cg03250832 |
| 17828 | cg05245760 |
| 17829 | cg06595206 |
| 17830 | cg04477660 |
| 17831 | cg17227564 |
| 17832 | cg12720965 |
| 17833 | cg25642476 |
| 17834 | cg20360009 |
| 17835 | cg04964857 |
| 17836 | cg06540739 |
| 17837 | cg24206669 |
| 17838 | cg12528056 |
| 17839 | cg27434809 |
| 17840 | cg17367472 |
| 17841 | cg07083848 |
| 17842 | cg11793860 |
| 17843 | cg18077866 |
| 17844 | cg26927807 |
| 17845 | cg23455302 |
| 17846 | cg26043485 |
| 17847 | cg15071463 |
| 17848 | cg03777931 |
| 17849 | cg05813297 |
| 17850 | cg24116965 |
| 17851 | cg02983163 |
| 17852 | cg02826739 |
| 17853 | cg24753998 |
| 17854 | cg08995661 |
| 17855 | cg27145679 |
| 17856 | cg13278267 |
| 17857 | cg17087916 |
| 17858 | cg23108843 |
| 17859 | cg13700089 |
| 17860 | cg27564043 |
| 17861 | cg17866778 |
| 17862 | cg09003373 |
| 17863 | cg03486379 |
| 17864 | cg14960550 |
| 17865 | cg01089579 |
| 17866 | cg18683103 |
| 17867 | cg11795809 |
| 17868 | cg13765278 |
[truncated: 49,031 more chars]
