# Supplementary figures and images for: Deep learning-based classifier for carcinoma of unknown primary using methylation quantitative trait loci
Source: J Neuropathol Exp Neurol. 2024 Nov 28;84(2):147–54. doi: 10.1093/jnen/nlae123 (PMC11747144; doi:10.1093/jnen/nlae123)

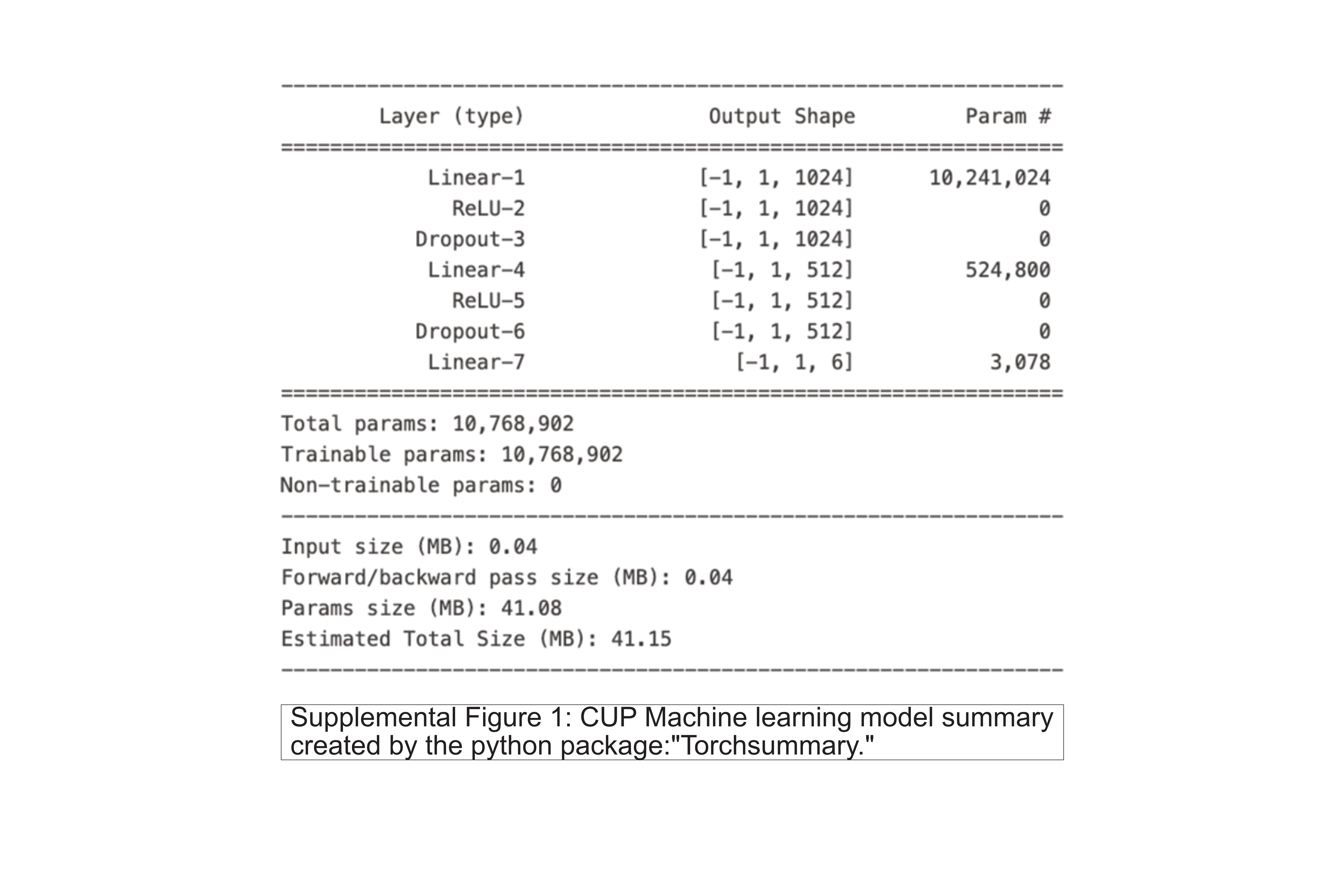

Supplement: nlae123_Supplementary_Data [file nlae123_supplementary_data.zip › nlae123_Supplementary_Data/Supp Figure 1.tiff]
